# Supplementary material for: Integrative analysis of Dupuytren's disease identifies novel risk locus and reveals a shared genetic etiology with BMI
Source: Genet Epidemiol. 2019 May 13;43(6):629–45. doi: 10.1002/gepi.22209 (PMC6699495; doi:10.1002/gepi.22209)
Supplement: Supplementary file 1 — Supporting information [file GEPI-43-629-s001.docx]

### SUPPLEMENTAL FIGURES


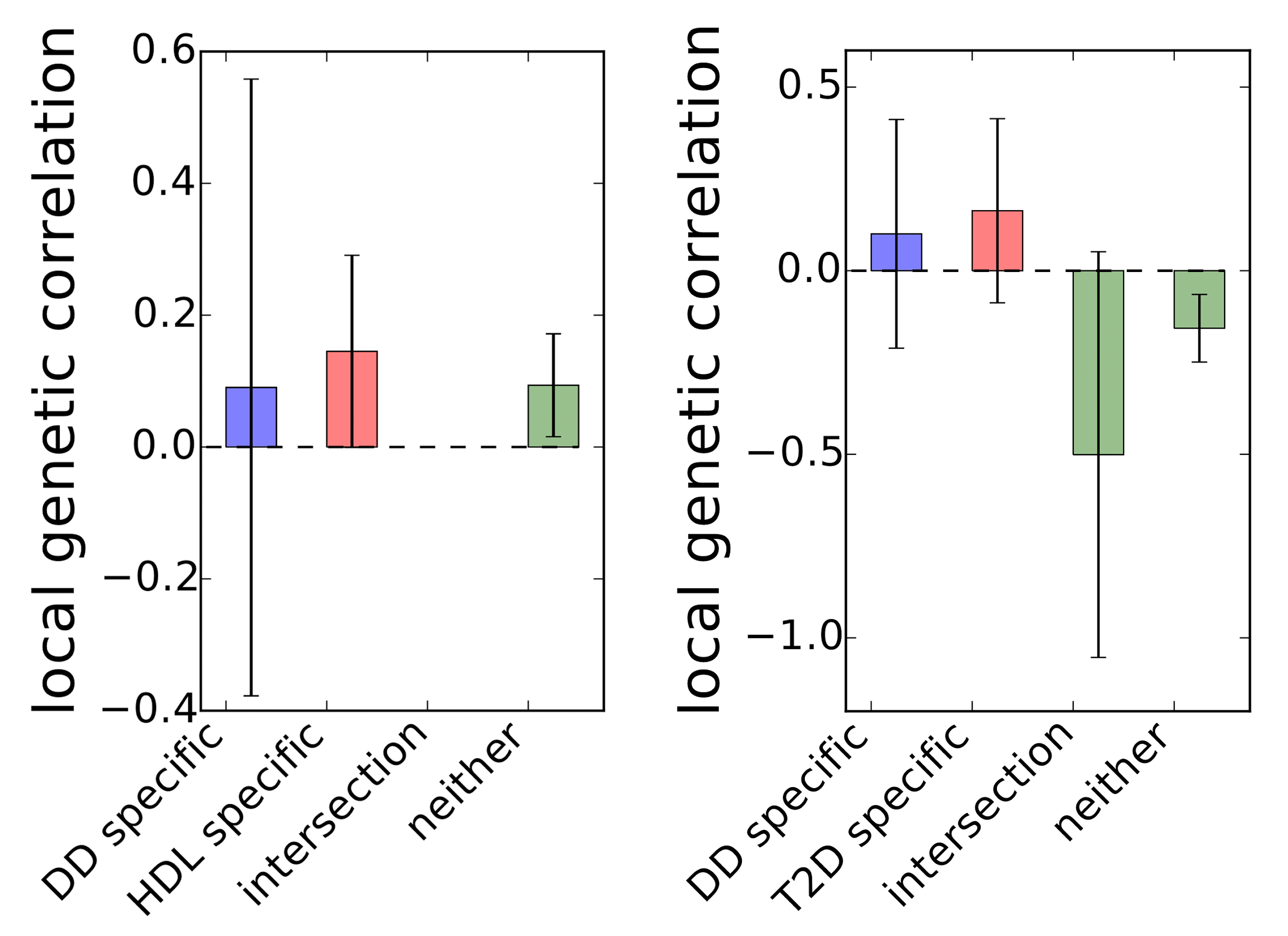


**Figure S1: No evidence for causal relationships of HDL or T2D on DD.** Here we have the genetic correlation for DD and HDL or DD and T2D between four groupings of SNPs: (1) DD specific significant GWAS SNPs, (2) HDL or T2D specific significant GWAS SNPs, (3) significant GWAS SNPs in both DD and HDL or T2D and (4) all non-significant SNPs shared between studies. While there is evidence of some shared genetics from effect of SNPs, the results found here are not consistent with a causal relationship of HDL or T2D on DD. Error bars are defined by the genetic correlation ± 1.96 times the s.e. for each grouping of SNPs.


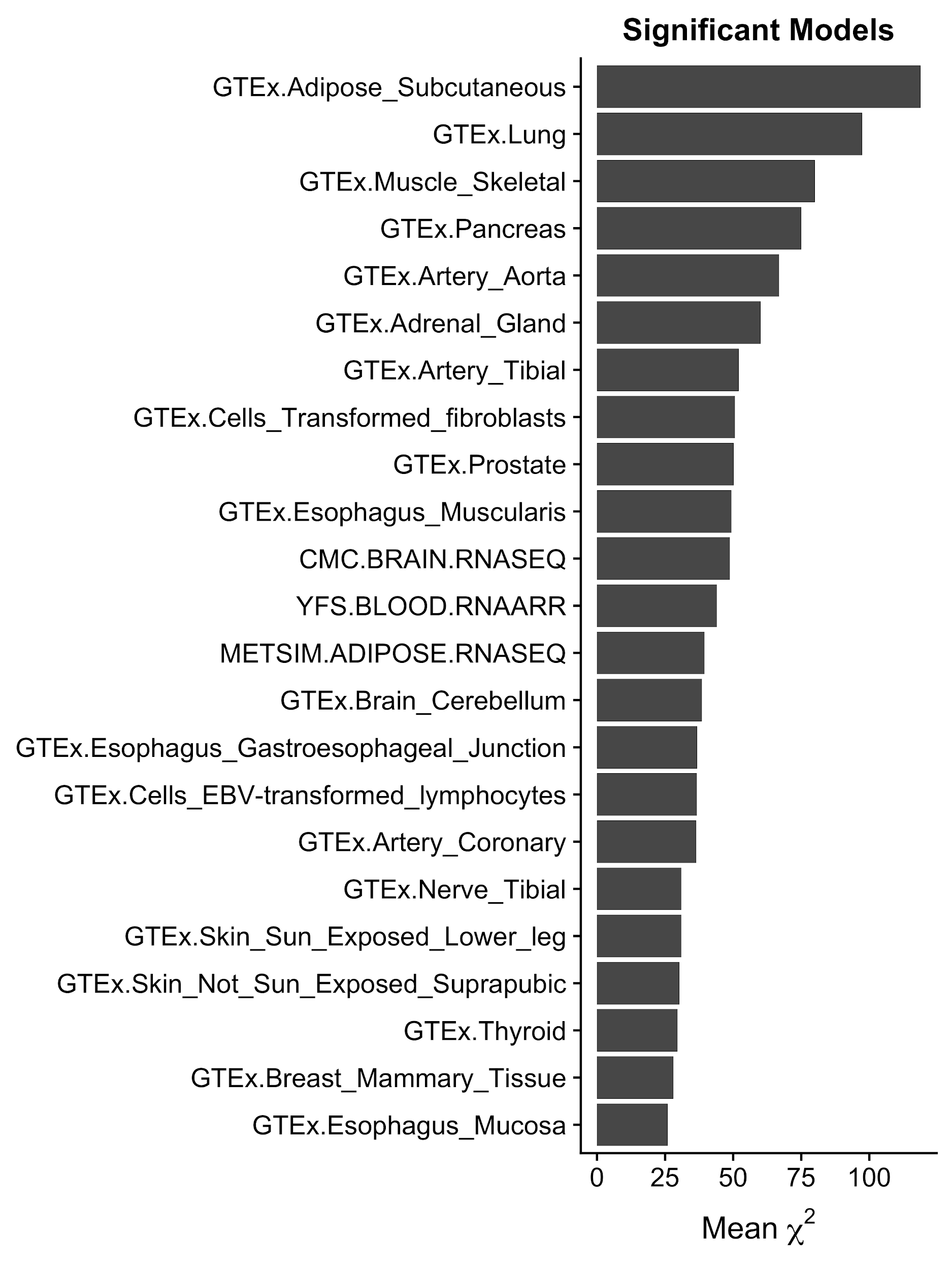


**Figure S2: Mean** $\chi^{\boldsymbol{2}}$ **over the 43 significant tissue-specific gene models from TWAS.** To find the tissue with the most signal from TWAS, we calculated the mean $\chi^{2}$statistic for all significant models within a tissue.
