# Supplementary material for: Integrative analysis of Dupuytren's disease identifies novel risk locus and reveals a shared genetic etiology with BMI
Source: Genet Epidemiol. 2019 May 13;43(6):629–45. doi: 10.1002/gepi.22209 (PMC6699495; doi:10.1002/gepi.22209)
Supplement: Supplementary file 2 — Supporting information [file GEPI-43-629-s002.docx]

### SUPPLEMENTAL TABLES

| Reference Panel | Sample Size | Number of Gene Models | Number of Genes with Significant Association |
| --- | --- | --- | --- |
| GTEx.Adipose_Subcutaneous | 298 | 3492 | 2 |
| GTEx.Adipose_Visceral_Omentum | 185 | 2037 | 0 |
| GTEx.Adrenal_Gland | 126 | 1457 | 1 |
| GTEx.Artery_Aorta | 197 | 2976 | 2 |
| GTEx.Artery_Coronary | 118 | 1183 | 1 |
| GTEx.Artery_Tibial | 285 | 3760 | 3 |
| GTEx.Brain_Caudate_basal_ganglia | 100 | 921 | 0 |
| GTEx.Brain_Cerebellar_Hemisphere | 89 | 1488 | 0 |
| GTEx.Brain_Cerebellum | 103 | 1982 | 2 |
| GTEx.Brain_Cortex | 96 | 1034 | 0 |
| GTEx.Brain_Frontal_Cortex_BA9 | 92 | 924 | 0 |
| GTEx.Brain_Hippocampus | 81 | 525 | 0 |
| GTEx.Brain_Hypothalamus | 81 | 590 | 0 |
| GTEx.Brain_Nucleus_accumbens_basal_ganglia | 93 | 865 | 0 |
| GTEx.Brain_Putamen_basal_ganglia | 82 | 624 | 0 |
| GTEx.Breast_Mammary_Tissue | 183 | 1974 | 1 |
| GTEx.Cells_EBV-transformed_lymphocytes | 114 | 1420 | 1 |
| GTEx.Cells_Transformed_fibroblasts | 272 | 4198 | 4 |
| GTEx.Colon_Sigmoid | 124 | 1460 | 0 |
| GTEx.Colon_Transverse | 169 | 2064 | 0 |
| GTEx.Esophagus_Gastroesophageal_Junction | 127 | 1446 | 1 |
| GTEx.Esophagus_Mucosa | 241 | 3319 | 1 |
| GTEx.Esophagus_Muscularis | 218 | 3230 | 3 |
| GTEx.Heart_Atrial_Appendage | 159 | 1579 | 0 |
| GTEx.Heart_Left_Ventricle | 190 | 1531 | 0 |
| GTEx.Liver | 97 | 717 | 0 |
| GTEx.Lung | 278 | 2872 | 2 |
| GTEx.Muscle_Skeletal | 361 | 2891 | 2 |
| GTEx.Nerve_Tibial | 256 | 4324 | 4 |
| GTEx.Ovary | 85 | 909 | 0 |
| GTEx.Pancreas | 149 | 1632 | 1 |
| GTEx.Pituitary | 87 | 1115 | 0 |
| GTEx.Prostate | 87 | 834 | 1 |
| GTEx.Skin_Not_Sun_Exposed_Suprapubic | 196 | 2460 | 1 |
| GTEx.Skin_Sun_Exposed_Lower_leg | 302 | 3636 | 1 |
| GTEx.Small_Intestine_Terminal_Ileum | 77 | 461 | 0 |
| GTEx.Spleen | 89 | 1410 | 0 |
| GTEx.Stomach | 170 | 1455 | 0 |
| GTEx.Testis | 157 | 3155 | 0 |
| GTEx.Thyroid | 278 | 3997 | 1 |
| GTEx.Uterus | 70 | 566 | 0 |
| GTEx.Vagina | 79 | 635 | 0 |
| GTEx.Whole_Blood | 338 | 1991 | 0 |
| METSIM.ADIPOSE.RNASEQ | 563 | 4609 | 1 |
| YFS.BLOOD.RNAARR | 1264 | 4618 | 4 |
| CMC.BRAIN.RNASEQ | 452 | 5377 | 3 |
| NTR.BLOOD.RNAARR | 1247 | 2404 | 0 |

**Table S1: Tissue reference panels for predicting gene expression in TWAS.**

| Gene | Chr | TSS | TES | Best GWAS SNP | $Z_{GWAS}$ | Reference Tissue Panel | cis-$h_{g}^{2}$ | $P_{TWAS}$ |
| --- | --- | --- | --- | --- | --- | --- | --- | --- |
| PJA2 | 5 | 108,670,409 | 108,745,675 | rs414724 | -6.4 | GTEx.Nerve_Tibial | 0.09 | 1.1E-07 |
| CTD-2587M2.1 | 5 | 108,572,821 | 108,662,070 | rs414724 | -6.4 | METSIM.ADIPOSE.RNASEQ | 0.18 | 3.3E-10 |
| MAN2A1 | 5 | 109,025,066 | 109,205,326 | rs414724 | -6.4 | GTEx.Nerve_Tibial | 0.07 | 2.4E-08 |
| SDK1 | 7 | 3,341,079 | 4,308,631 | rs10264803 | -6.0 | GTEx.Cells_Transformed_fibroblasts | 0.17 | 3.3E-09 |
| SDK1 | 7 | 3,341,079 | 4,308,631 | rs10264803 | -6.0 | GTEx.Esophagus_Muscularis | 0.08 | 6.4E-08 |
| EPDR1 | 7 | 37,960,162 | 37,991,542 | rs17171240 | 14.7 | GTEx.Lung | 0.15 | 6.4E-31 |
| EPDR1 | 7 | 37,960,162 | 37,991,542 | rs17171240 | 14.7 | GTEx.Adipose_Subcutaneous | 0.12 | 5.1E-23 |
| EPDR1 | 7 | 37,960,162 | 37,991,542 | rs17171240 | 14.7 | GTEx.Pancreas | 0.19 | 4.6E-18 |
| EPDR1 | 7 | 37,960,162 | 37,991,542 | rs17171240 | 14.7 | GTEx.Esophagus_Muscularis | 0.35 | 6.6E-14 |
| EPDR1 | 7 | 37,960,162 | 37,991,542 | rs17171240 | 14.7 | YFS.BLOOD.RNAARR | 0.18 | 1.4E-13 |
| EPDR1 | 7 | 37,960,162 | 37,991,542 | rs17171240 | 14.7 | GTEx.Nerve_Tibial | 0.30 | 5.0E-09 |
| EPDR1 | 7 | 37,960,162 | 37,991,542 | rs17171240 | 14.7 | GTEx.Artery_Tibial | 0.27 | 1.5E-08 |
| EPDR1 | 7 | 37,960,162 | 37,991,542 | rs17171240 | 14.7 | GTEx.Thyroid | 0.19 | 5.4E-08 |
| EPDR1 | 7 | 37,960,162 | 37,991,542 | rs17171240 | 14.7 | GTEx.Cells_Transformed_fibroblasts | 0.21 | 1.9E-07 |
| EPDR1 | 7 | 37,960,162 | 37,991,542 | rs17171240 | 14.7 | CMC.BRAIN.RNASEQ | 0.24 | 3.1E-07 |
| TRGC2 | 7 | 38,279,181 | 38,289,173 | rs17171240 | 14.7 | GTEx.Prostate | 0.37 | 1.4E-12 |
| SULF1 | 8 | 70,378,858 | 70,573,147 | rs542288 | 11.8 | GTEx.Artery_Aorta | 0.27 | 4.0E-25 |
| RSPO2 | 8 | 108,911,543 | 109,095,913 | rs612265 | -9.3 | CMC.BRAIN.RNASEQ | 0.12 | 1.2E-08 |
| EIF3E | 8 | 109,213,971 | 109,260,959 | rs612265 | -9.3 | CMC.BRAIN.RNASEQ | 0.07 | 7.6E-21 |
| EIF3E | 8 | 109,213,971 | 109,260,959 | rs612265 | -9.3 | YFS.BLOOD.RNAARR | 0.01 | 1.4E-15 |
| EIF3E | 8 | 109,213,971 | 109,260,959 | rs612265 | -9.3 | GTEx.Brain_Cerebellum | 0.16 | 1.8E-11 |
| EMC2 | 8 | 109,455,852 | 109,499,136 | rs612265 | -9.3 | GTEx.Muscle_Skeletal | 0.05 | 8.9E-12 |
| EMC2 | 8 | 109,455,852 | 109,499,136 | rs612265 | -9.3 | GTEx.Esophagus_Gastroesophageal_Junction | 0.09 | 1.3E-09 |
| EMC2 | 8 | 109,455,852 | 109,499,136 | rs612265 | -9.3 | GTEx.Brain_Cerebellum | 0.29 | 1.6E-08 |
| MRPL52 | 14 | 23,299,091 | 23,304,246 | rs1042704 | 7.3 | YFS.BLOOD.RNAARR | 0.44 | 1.1E-07 |
| NEDD4 | 15 | 56,119,116 | 56,285,944 | rs8032158 | 5.2 | GTEx.Artery_Tibial | 0.18 | 2.6E-07 |
| BCAR1 | 16 | 75,262,927 | 75,301,951 | rs977987 | 5.9 | GTEx.Artery_Aorta | 0.18 | 2.8E-07 |
| BCAR1 | 16 | 75,262,927 | 75,301,951 | rs977987 | 5.9 | GTEx.Esophagus_Mucosa | 0.16 | 3.6E-07 |
| CFDP1 | 16 | 75,327,607 | 75,467,387 | rs977987 | 5.9 | YFS.BLOOD.RNAARR | 0.21 | 5.6E-08 |
| TMEM170A | 16 | 75,480,922 | 75,498,584 | rs977987 | 5.9 | GTEx.Cells_EBV-transformed_lymphocytes | 0.13 | 1.4E-09 |
| TMEM170A | 16 | 75,480,922 | 75,498,584 | rs977987 | 5.9 | GTEx.Skin_Sun_Exposed_Lower_leg | 0.14 | 2.7E-08 |
| TMEM170A | 16 | 75,480,922 | 75,498,584 | rs977987 | 5.9 | GTEx.Skin_Not_Sun_Exposed_Suprapubic | 0.09 | 3.9E-08 |
| TMEM106A* | 17 | 41,363,845 | 41,372,057 | rs4793248 | 4.1 | GTEx.Breast_Mammary_Tissue | 0.12 | 1.2E-07 |
| ATXN10 | 22 | 46,067,677 | 46,241,187 | rs34088184 | 13.8 | GTEx.Cells_Transformed_fibroblasts | 0.17 | 1.7E-07 |
| LINC00899 | 22 | 46,435,786 | 46,440,748 | rs34088184 | 13.8 | GTEx.Adipose_Subcutaneous | 0.17 | 2.8E-32 |
| LINC00899 | 22 | 46,435,786 | 46,440,748 | rs34088184 | 13.8 | GTEx.Muscle_Skeletal | 0.24 | 1.6E-26 |
| LINC00899 | 22 | 46,435,786 | 46,440,748 | rs34088184 | 13.8 | GTEx.Cells_Transformed_fibroblasts | 0.18 | 1.9E-26 |
| LINC00899 | 22 | 46,435,786 | 46,440,748 | rs34088184 | 13.8 | GTEx.Artery_Tibial | 0.30 | 5.6E-23 |
| LINC00899 | 22 | 46,435,786 | 46,440,748 | rs34088184 | 13.8 | GTEx.Esophagus_Muscularis | 0.36 | 2.3E-15 |
| LINC00899 | 22 | 46,435,786 | 46,440,748 | rs34088184 | 13.8 | GTEx.Lung | 0.21 | 5.7E-15 |
| LINC00899 | 22 | 46,435,786 | 46,440,748 | rs34088184 | 13.8 | GTEx.Adrenal_Gland | 0.68 | 9.2E-15 |
| LINC00899 | 22 | 46,435,786 | 46,440,748 | rs34088184 | 13.8 | GTEx.Artery_Coronary | 0.56 | 1.6E-09 |
| LINC00899 | 22 | 46,435,786 | 46,440,748 | rs34088184 | 13.8 | GTEx.Nerve_Tibial | 0.35 | 3.4E-08 |

**Table S2: Significant tissue-specific gene expression models from TWAS.** These are the 43 significant ($P_{TWAS}\leq\frac{0.05}{98147}$) tissue-specific gene models representing 18 unique genes. The novel risk region is from the *TMEM106A* model significant in the GTEx breast mammary tissue, indicated by “*”.

| Reference Tissue Panel | cis-$h_{g}^{2}$ | Best GWAS SNP | $Z_{GWAS}$ | $Z_{TWAS}$ | $P_{TWAS}$ |
| --- | --- | --- | --- | --- | --- |
| GTEx.Artery_Aorta | 0.24 | rs4793248 | 4.12 | 3.42 | 6.2E-04 |
| GTEx.Artery_Coronary | 0.17 | rs4793248 | 4.12 | 4.16 | 3.1E-05 |
| GTEx.Artery_Tibial | 0.16 | rs4793248 | 4.12 | 3.37 | 7.4E-04 |
| GTEx.Breast_Mammary_Tissue | 0.12 | rs4793248 | 4.12 | 5.3 | 1.2E-07 |
| GTEx.Cells_EBV-transformed_lymphocytes | 0.46 | rs4793248 | 4.12 | -3.12 | 0.0018 |
| GTEx.Heart_Atrial_Appendage | 0.08 | rs4793248 | 4.12 | 4.8 | 1.6E-06 |
| GTEx.Nerve_Tibial | 0.15 | rs4793248 | 4.12 | 3.63 | 2.8E-04 |
| GTEx.Pituitary | 0.15 | rs4793248 | 4.12 | 3.76 | 1.7E-04 |
| GTEx.Skin_Not_Sun_Exposed_Suprapubic | 0.07 | rs4793248 | 4.12 | 1.9 | 0.0571 |
| GTEx.Small_Intestine_Terminal_Ileum | 0.15 | rs4793248 | 4.12 | 4.29 | 1.8E-05 |
| GTEx.Vagina | 0.41 | rs4793248 | 4.12 | 1.73 | 0.0845 |
| YFS.BLOOD.RNAARR | 0.02 | rs4793248 | 4.12 | -1.3 | 0.1948 |

**Table S3: All tissue-specific gene models for the *TMEM106A*.** Here we have the 12 different tissues that *TMEM106A* (chr17:41363845-41372057) expression was tested with for association with DD. Only the GTEx breast mammary tissue model was significant.

| Trait | LDSC $\hat{r}_{g}$ | s.e. | ρ-HESS $\hat{r}_{g}$ | s.e. |
| --- | --- | --- | --- | --- |
| BMI | -0.196 | 0.04 | -0.229 | 0.02 |
| HDL | 0.133 | 0.04 | 0.093 | 0.03 |
| TG | -0.139 | 0.04 | -0.109 | 0.03 |
| T2D | -0.182 | 0.05 | -0.112 | 0.04 |

**Table S4: Genetic correlation for four traits with DD using LDSC and ρ-HESS.** Here are the genome-wide genetic correlation estimates from LDSC and ρ-HESS for four traits. Pearson’s correlation between these estimates is 0.94.

| Name | Coefficient | s.e. | $P_{SEG,GE}$ |
| --- | --- | --- | --- |
| A02.835.583.443.800.Synovial.Membrane | 8.64E-08 | 4.07E-08 | 0.01684 |
| A05.360.319.679.256.Cervix.Uteri | 1.10E-07 | 5.65E-08 | 0.02582 |
| Artery_Aorta | 8.15E-08 | 4.20E-08 | 0.02613 |
| Cervix_Endocervix | 1.15E-07 | 5.95E-08 | 0.02703 |
| A03.734.Pancreas | 1.03E-07 | 5.67E-08 | 0.03469 |
| Artery_Tibial | 9.04E-08 | 5.00E-08 | 0.03532 |
| Breast_Mammary_Tissue | 8.53E-08 | 4.87E-08 | 0.03991 |
| Bladder | 7.25E-08 | 4.20E-08 | 0.04224 |
| A05.360.Genitalia | 9.61E-08 | 5.69E-08 | 0.04557 |
| A04.411.Lung | 6.35E-08 | 3.76E-08 | 0.04573 |
| A03.556.249.249.356.668.Colon..Sigmoid | 9.73E-08 | 5.77E-08 | 0.04582 |
| A10.615.789.Serous.Membrane | 8.83E-08 | 5.26E-08 | 0.04669 |
| A15.382.Immune.System | 5.93E-08 | 3.85E-08 | 0.06174 |
| A03.556.124.526.767.Rectum | 9.17E-08 | 6.00E-08 | 0.06319 |
| A07.541.510.110.Aortic.Valve | 6.80E-08 | 4.55E-08 | 0.0677 |
| A05.360.319.Genitalia..Female | 6.34E-08 | 4.41E-08 | 0.07517 |
| A05.810.890.Urinary.Bladder | 8.81E-08 | 6.14E-08 | 0.07573 |
| Cervix_Ectocervix | 7.85E-08 | 5.48E-08 | 0.07577 |
| A11.118.637.Leukocytes | 6.55E-08 | 4.60E-08 | 0.07719 |
| A05.360.319.679.Uterus | 6.92E-08 | 4.88E-08 | 0.07803 |
| Cells_EBV-transformed_lymphocytes | 8.37E-08 | 5.92E-08 | 0.0789 |
| A03.556.875.500.Esophagus | 6.93E-08 | 5.21E-08 | 0.09183 |
| A15.382.812.260.Dendritic.Cells | 4.87E-08 | 3.67E-08 | 0.09221 |
| Vagina | 6.46E-08 | 5.06E-08 | 0.10091 |
| Uterus | 7.07E-08 | 5.65E-08 | 0.10536 |
| Adipose_Subcutaneous | 5.64E-08 | 4.51E-08 | 0.10549 |
| A15.378.316.580.Monocytes | 5.03E-08 | 4.17E-08 | 0.11382 |
| A05.360.319.114.630.Ovary | 7.03E-08 | 5.85E-08 | 0.11476 |
| A03.556.124.684.Intestine..Small | 6.23E-08 | 5.28E-08 | 0.11905 |
| Artery_Coronary | 5.06E-08 | 4.32E-08 | 0.12069 |
| A11.329.171.Chondrocytes | 6.14E-08 | 5.43E-08 | 0.12936 |
| Cells_Transformed_fibroblasts | 5.49E-08 | 4.87E-08 | 0.12975 |
| A15.382.680.Phagocytes | 4.33E-08 | 3.85E-08 | 0.13064 |
| A03.556.249.249.209.Cecum | 6.39E-08 | 5.71E-08 | 0.1316 |
| A11.329.372.600.Macrophages..Alveolar | 3.74E-08 | 3.51E-08 | 0.14366 |
| A15.382.490.555.567.537.Killer.Cells..Natural | 4.77E-08 | 4.69E-08 | 0.15467 |
| A15.145.229.637.555.Leukocytes..Mononuclear | 4.27E-08 | 4.24E-08 | 0.15671 |
| A11.436.294.064.Glucagon.Secreting.Cells | 3.99E-08 | 3.99E-08 | 0.15831 |
| A06.407.Endocrine.Glands | 5.73E-08 | 5.73E-08 | 0.15872 |
| A05.360.444.Genitalia..Male | 3.35E-08 | 3.50E-08 | 0.16938 |
| A11.872.190.Embryonic.Stem.Cells | 4.70E-08 | 4.96E-08 | 0.17134 |
| Minor_Salivary_Gland | 3.25E-08 | 3.45E-08 | 0.17305 |
| A02.165.Cartilage | 3.76E-08 | 4.03E-08 | 0.17532 |
| A06.407.312.Gonads | 5.16E-08 | 5.63E-08 | 0.17995 |
| A06.407.900.Thyroid.Gland | 3.88E-08 | 4.24E-08 | 0.18011 |
| A15.382.812.Mononuclear.Phagocyte.System | 3.72E-08 | 4.17E-08 | 0.18659 |
| A11.872.700.500.Induced.Pluripotent.Stem.Cells | 4.58E-08 | 5.35E-08 | 0.19584 |
| A10.336.707.Prostate | 2.65E-08 | 3.39E-08 | 0.21675 |
| A09.371.729.Retina | 2.82E-08 | 3.64E-08 | 0.21953 |
| Lung | 2.60E-08 | 3.43E-08 | 0.22361 |
| A11.118.637.555.567.562.B.Lymphocytes | 2.88E-08 | 3.82E-08 | 0.22594 |
| Spleen | 3.30E-08 | 4.40E-08 | 0.22674 |
| Nerve_Tibial | 2.66E-08 | 3.67E-08 | 0.23422 |
| A03.556.249.249.356.Colon | 3.88E-08 | 5.56E-08 | 0.24244 |
| A05.360.319.887.Vulva | 3.07E-08 | 4.41E-08 | 0.24361 |
| Adrenal_Gland | 2.86E-08 | 4.16E-08 | 0.24631 |
| A03.734.414.Islets.of.Langerhans | 2.57E-08 | 3.87E-08 | 0.25339 |
| A06.407.071.140.Adrenal.Cortex | 2.36E-08 | 3.70E-08 | 0.26149 |
| A11.382.Endocrine.Cells | 3.10E-08 | 4.91E-08 | 0.2634 |
| Colon_Transverse | 2.14E-08 | 3.42E-08 | 0.2663 |
| A15.382.812.522.Macrophages | 2.24E-08 | 3.60E-08 | 0.26719 |
| Brain_Spinal_cord_(cervical_c-1) | 1.96E-08 | 3.22E-08 | 0.27141 |
| A11.497.497.600.Oocytes | 2.22E-08 | 3.75E-08 | 0.27665 |
| A10.615.Membranes | 2.38E-08 | 4.24E-08 | 0.28736 |
| A05.360.319.679.490.Endometrium | 2.38E-08 | 4.24E-08 | 0.28751 |
| A11.872.190.260.Embryoid.Bodies | 1.98E-08 | 3.71E-08 | 0.29699 |
| A05.810.453.324.Kidney.Cortex | 1.86E-08 | 3.69E-08 | 0.30689 |
| A05.360.319.679.690.Myometrium | 1.82E-08 | 4.06E-08 | 0.32678 |
| Colon_Sigmoid | 3.20E-08 | 7.14E-08 | 0.32697 |
| Thyroid | 1.45E-08 | 3.31E-08 | 0.33089 |
| Ovary | 1.86E-08 | 4.88E-08 | 0.35113 |
| A06.407.312.782.Testis | 1.57E-08 | 4.28E-08 | 0.35661 |
| Prostate | 1.19E-08 | 3.57E-08 | 0.36927 |
| A05.360.490.Germ.Cells | 1.23E-08 | 3.70E-08 | 0.37043 |
| A06.407.071.Adrenal.Glands | 1.18E-08 | 3.78E-08 | 0.37752 |
| A05.810.453.Kidney | 1.25E-08 | 4.07E-08 | 0.37955 |
| A11.436.329.Granulosa.Cells | 1.43E-08 | 4.86E-08 | 0.38414 |
| A03.556.124.Intestines | 1.23E-08 | 4.24E-08 | 0.38575 |
| A03.556.875.Upper.Gastrointestinal.Tract | 1.53E-08 | 5.43E-08 | 0.38891 |
| Small_Intestine_Terminal_Ileum | 9.77E-09 | 3.50E-08 | 0.39014 |
| A10.165.114.830.750.Subcutaneous.Fat | 1.15E-08 | 4.15E-08 | 0.39114 |
| A10.549.400.Lymph.Nodes | 1.03E-08 | 3.88E-08 | 0.39552 |
| Brain_Hypothalamus | 9.15E-09 | 3.86E-08 | 0.40626 |
| Esophagus_Mucosa | 6.64E-09 | 3.32E-08 | 0.42083 |
| Fallopian_Tube | 7.61E-09 | 3.99E-08 | 0.42436 |
| A15.382.490.555.567.Lymphocytes | 5.92E-09 | 4.03E-08 | 0.44171 |
| A08.186.211.132.810.428.200.Cerebellum | 4.45E-09 | 3.15E-08 | 0.44381 |
| A08.186.211.865.428.Metencephalon | 3.68E-09 | 3.17E-08 | 0.45374 |
| A10.615.550.Mucous.Membrane | 4.16E-09 | 4.26E-08 | 0.46116 |
| A08.186.211.730.317.357.Hypothalamus | 3.38E-09 | 4.61E-08 | 0.47073 |
| Adipose_Visceral_(Omentum) | 2.78E-09 | 5.07E-08 | 0.47809 |
| Brain_Substantia_nigra | 5.29E-10 | 4.15E-08 | 0.49491 |
| A11.872.653.Neural.Stem.Cells | -2.62E-10 | 4.08E-08 | 0.50257 |
| Brain_Hippocampus | -1.20E-09 | 3.14E-08 | 0.51527 |
| A10.165.114.830.500.750.Subcutaneous.Fat..Abdominal | -3.13E-09 | 4.10E-08 | 0.53041 |
| A11.872.040.Adult.Stem.Cells | -3.96E-09 | 3.85E-08 | 0.54087 |
| A11.329.629.Osteoblasts | -3.51E-09 | 3.25E-08 | 0.54295 |
| A08.186.211.730.885.287.249.Basal.Ganglia | -4.34E-09 | 3.46E-08 | 0.54999 |
| A11.329.114.Adipocytes | -5.41E-09 | 3.94E-08 | 0.55456 |
| A05.360.319.114.373.Fallopian.Tubes | -5.96E-09 | 4.31E-08 | 0.55507 |
| A10.690.Muscles | -6.75E-09 | 3.63E-08 | 0.57367 |
| A03.556.124.369.Intestinal.Mucosa | -8.02E-09 | 3.86E-08 | 0.58234 |
| A08.186.211.132.Brain.Stem | -7.13E-09 | 3.29E-08 | 0.58578 |
| A15.378.316.Bone.Marrow.Cells | -9.43E-09 | 4.16E-08 | 0.58955 |
| A15.145.300.Fetal.Blood | -1.26E-08 | 4.88E-08 | 0.602 |
| A02.835.583.443.800.800.Synovial.Fluid | -1.15E-08 | 4.27E-08 | 0.6062 |
| A11.118.637.555.567.562.440.Precursor.Cells..B.Lymphoid | -1.08E-08 | 3.98E-08 | 0.60741 |
| Skin_Not_Sun_Exposed_(Suprapubic) | -1.05E-08 | 3.68E-08 | 0.6125 |
| A07.231.114.Arteries | -1.24E-08 | 3.99E-08 | 0.62246 |
| A11.329.Connective.Tissue.Cells | -1.21E-08 | 3.70E-08 | 0.62822 |
| A15.382.490.315.583.Neutrophils | -1.28E-08 | 3.56E-08 | 0.63997 |
| A03.556.249.124.Ileum | -1.58E-08 | 4.24E-08 | 0.64508 |
| A10.549.Lymphoid.Tissue | -1.42E-08 | 3.79E-08 | 0.64567 |
| A15.145.229.Blood.Cells | -1.60E-08 | 4.22E-08 | 0.64726 |
| A15.145.229.188.Blood.Platelets | -1.58E-08 | 3.39E-08 | 0.67893 |
| A15.145.Blood | -2.03E-08 | 4.28E-08 | 0.68296 |
| A08.186.211.464.Limbic.System | -1.92E-08 | 3.94E-08 | 0.68751 |
| A15.382.490.555.567.622.Lymphocytes..Null | -1.88E-08 | 3.82E-08 | 0.68837 |
| A15.145.229.637.555.567.562.725.Plasma.Cells | -1.73E-08 | 3.51E-08 | 0.68922 |
| A11.627.624.249.Monocyte.Macrophage.Precursor.Cells | -1.94E-08 | 3.76E-08 | 0.69698 |
| Esophagus_Gastroesophageal_Junction | -3.08E-08 | 5.87E-08 | 0.70004 |
| A08.186.211.730.317.357.352.435.Hypothalamo.Hypophyseal.System | -2.28E-08 | 4.34E-08 | 0.70047 |
| A10.165.450.300.Cicatrix | -1.69E-08 | 3.18E-08 | 0.70192 |
| A11.872.378.590.635.Granulocyte.Macrophage.Progenitor.Cells | -2.55E-08 | 4.77E-08 | 0.70315 |
| A03.556.Gastrointestinal.Tract | -2.01E-08 | 3.56E-08 | 0.71347 |
| Liver | -1.79E-08 | 3.14E-08 | 0.71542 |
| A05.360.444.492.362.Foreskin | -1.99E-08 | 3.38E-08 | 0.72263 |
| Brain_Cerebellar_Hemisphere | -1.97E-08 | 3.28E-08 | 0.72554 |
| Brain_Amygdala | -1.84E-08 | 3.04E-08 | 0.72776 |
| A03.556.500.760.Salivary.Glands | -2.25E-08 | 3.68E-08 | 0.72946 |
| A10.615.284.473.Chorion | -2.26E-08 | 3.56E-08 | 0.73693 |
| A11.436.348.Hepatocytes | -2.61E-08 | 4.11E-08 | 0.73723 |
| A11.872.Stem.Cells | -2.51E-08 | 3.89E-08 | 0.74059 |
| Muscle_Skeletal | -2.53E-08 | 3.73E-08 | 0.7509 |
| A08.186.211.653.Mesencephalon | -2.36E-08 | 3.47E-08 | 0.75148 |
| A14.724.Pharynx | -2.88E-08 | 4.16E-08 | 0.75565 |
| A14.549.167.Dentition | -3.48E-08 | 4.77E-08 | 0.76677 |
| Skin_Sun_Exposed_(Lower_leg) | -2.66E-08 | 3.61E-08 | 0.76997 |
| A08.186.211.464.405.Hippocampus | -2.45E-08 | 3.12E-08 | 0.78354 |
| A03.556.500.760.464.Parotid.Gland | -2.90E-08 | 3.67E-08 | 0.78543 |
| A14.724.557.Nasopharynx | -3.38E-08 | 4.26E-08 | 0.78619 |
| A03.556.875.875.Stomach | -3.29E-08 | 3.97E-08 | 0.79612 |
| Brain_Frontal_Cortex_(BA9) | -2.59E-08 | 3.11E-08 | 0.79738 |
| Whole_Blood | -3.19E-08 | 3.66E-08 | 0.8088 |
| A08.186.211.730.885.287.249.487.Corpus.Striatum | -2.78E-08 | 3.17E-08 | 0.80965 |
| A10.272.Epithelium | -3.62E-08 | 4.04E-08 | 0.8146 |
| A11.436.Epithelial.Cells | -3.46E-08 | 3.69E-08 | 0.82576 |
| A03.620.Liver | -3.29E-08 | 3.46E-08 | 0.82956 |
| A11.443.Erythroid.Cells | -3.41E-08 | 3.53E-08 | 0.83319 |
| A10.165.450.300.425.Keloid | -3.38E-08 | 3.40E-08 | 0.83999 |
| A17.815.Skin | -3.89E-08 | 3.78E-08 | 0.84784 |
| A11.118.637.555.567.569.200.700.T.Lymphocytes..Regulatory | -4.03E-08 | 3.87E-08 | 0.85113 |
| A15.145.229.637.555.567.569.200.CD4.Positive.T.Lymphocytes | -4.01E-08 | 3.82E-08 | 0.85331 |
| A02.835.232.834.151.Cervical.Vertebrae | -3.84E-08 | 3.63E-08 | 0.85499 |
| A11.118.637.555.567.569.T.Lymphocytes | -4.03E-08 | 3.79E-08 | 0.85638 |
| A11.436.275.Endothelial.Cells | -3.65E-08 | 3.38E-08 | 0.85968 |
| A04.531.520.Nasal.Mucosa | -4.27E-08 | 3.92E-08 | 0.86201 |
| Brain_Cerebellum | -3.09E-08 | 2.82E-08 | 0.864 |
| A15.382.520.604.700.Spleen | -4.37E-08 | 3.98E-08 | 0.86406 |
| A08.186.211.730.885.287.500.670.Parietal.Lobe | -3.92E-08 | 3.56E-08 | 0.86497 |
| Stomach | -3.87E-08 | 3.50E-08 | 0.86558 |
| A08.186.211.730.885.287.500.270.Frontal.Lobe | -3.46E-08 | 3.09E-08 | 0.86892 |
| A08.186.211.730.885.287.500.Cerebral.Cortex | -3.84E-08 | 3.25E-08 | 0.88193 |
| A10.615.550.599.Mouth.Mucosa | -4.48E-08 | 3.74E-08 | 0.88425 |
| A02.633.567.850.Quadriceps.Muscle | -4.03E-08 | 3.25E-08 | 0.89197 |
| A08.186.211.730.317.Diencephalon | -5.08E-08 | 3.97E-08 | 0.89993 |
| A10.690.467.Muscle..Smooth | -4.35E-08 | 3.35E-08 | 0.90319 |
| A08.186.211.730.885.287.500.571.735.Visual.Cortex | -3.91E-08 | 2.95E-08 | 0.90789 |
| Brain_Caudate_(basal_ganglia) | -3.54E-08 | 2.64E-08 | 0.91002 |
| A15.145.846.Serum | -5.42E-08 | 4.01E-08 | 0.91183 |
| Pancreas | -5.09E-08 | 3.55E-08 | 0.92409 |
| A15.382.520.604.800.Palatine.Tonsil | -5.85E-08 | 4.07E-08 | 0.92481 |
| A11.872.378.Hematopoietic.Stem.Cells | -5.05E-08 | 3.50E-08 | 0.92512 |
| Kidney_Cortex | -5.06E-08 | 3.35E-08 | 0.9345 |
| A11.627.340.360.Granulocyte.Precursor.Cells | -5.95E-08 | 3.87E-08 | 0.93809 |
| A11.329.228.Fibroblasts | -5.91E-08 | 3.71E-08 | 0.94425 |
| A11.872.580.Mesenchymal.Stem.Cells | -6.01E-08 | 3.71E-08 | 0.94734 |
| A11.620.520.Myocytes..Smooth.Muscle | -5.85E-08 | 3.56E-08 | 0.94994 |
| A11.329.830.Stromal.Cells | -7.73E-08 | 4.66E-08 | 0.95129 |
| Testis | -5.39E-08 | 3.23E-08 | 0.95278 |
| A08.186.211.464.710.225.Entorhinal.Cortex | -5.46E-08 | 3.20E-08 | 0.95586 |
| A08.186.211.Brain | -5.47E-08 | 3.21E-08 | 0.95595 |
| Brain_Putamen_(basal_ganglia) | -4.56E-08 | 2.66E-08 | 0.95664 |
| A14.549.Mouth | -6.07E-08 | 3.54E-08 | 0.95689 |
| A07.231.908.670.874.Umbilical.Veins | -5.31E-08 | 3.03E-08 | 0.96049 |
| A14.549.167.646.Periodontium | -6.64E-08 | 3.56E-08 | 0.96913 |
| A11.872.378.590.817.Megakaryocyte.Erythroid.Progenitor.Cells | -5.99E-08 | 3.18E-08 | 0.97009 |
| Esophagus_Muscularis | -7.29E-08 | 3.71E-08 | 0.97515 |
| A11.627.635.Myeloid.Progenitor.Cells | -6.98E-08 | 3.54E-08 | 0.97585 |
| Brain_Cortex | -4.89E-08 | 2.45E-08 | 0.97698 |
| A07.231.Blood.Vessels | -7.12E-08 | 3.55E-08 | 0.97745 |
| Brain_Anterior_cingulate_cortex_(BA24) | -5.10E-08 | 2.54E-08 | 0.97752 |
| Pituitary | -5.88E-08 | 2.89E-08 | 0.979 |
| A10.272.497.Epidermis | -7.40E-08 | 3.40E-08 | 0.98512 |
| A09.371.Eye | -6.93E-08 | 3.07E-08 | 0.98798 |
| A07.231.908.Veins | -7.05E-08 | 3.01E-08 | 0.99035 |
| A11.436.397.Keratinocytes | -8.96E-08 | 3.80E-08 | 0.99079 |
| A07.541.Heart | -7.60E-08 | 3.14E-08 | 0.99228 |
| Brain_Nucleus_accumbens_(basal_ganglia) | -6.85E-08 | 2.76E-08 | 0.99353 |
| A07.541.358.100.Atrial.Appendage | -8.61E-08 | 3.23E-08 | 0.99611 |
| A14.549.885.Tongue | -9.69E-08 | 3.54E-08 | 0.99691 |
| A07.541.358.Heart.Atria | -9.08E-08 | 3.24E-08 | 0.99747 |
| A07.541.560.Heart.Ventricles | -8.94E-08 | 3.00E-08 | 0.99854 |
| Heart_Atrial_Appendage | -1.08E-07 | 2.88E-08 | 0.9999 |
| Heart_Left_Ventricle | -1.41E-07 | 2.83E-08 | 1 |

**Table S5: LDSC-SEG results for 205 tissue gene expression annotations.** None were significant after FDR correction (FDR < 0.1).

| Name | Coefficient | s.e. | $P_{SEG,C}$ |
| --- | --- | --- | --- |
| Esoph-Mucosa_ENTEX__H3K4me1 | 9.82E-07 | 3.18E-07 | 0.00102 |
| NHLF_Lung_Fibroblast_Primary_Cells__H3K4me1 | 1.23E-06 | 5.11E-07 | 0.00796 |
| Artery-Tibial_ENTEX__H3K36me3 | 7.70E-07 | 3.20E-07 | 0.00811 |
| NHEK-Epidermal_Keratinocyte_Primary_Cells__H3K27ac | 9.85E-07 | 4.22E-07 | 0.00983 |
| NHDF-Ad_Adult_Dermal_Fibroblast_Primary_Cells__H3K27ac | 1.53E-06 | 6.58E-07 | 0.01018 |
| Duodenum_Smooth_Muscle__H3K36me3 | 9.44E-07 | 4.08E-07 | 0.0104 |
| NHEK-Epidermal_Keratinocyte_Primary_Cells__H3K9ac | 1.74E-06 | 7.52E-07 | 0.01041 |
| NHDF-Ad_Adult_Dermal_Fibroblast_Primary_Cells__DNase | 1.80E-06 | 7.95E-07 | 0.0116 |
| NHEK-Epidermal_Keratinocyte_Primary_Cells__H3K4me1 | 8.54E-07 | 3.78E-07 | 0.01195 |
| NHLF_Lung_Fibroblast_Primary_Cells__DNase | 2.02E-06 | 9.00E-07 | 0.01242 |
| Aorta_ENTEX__H3K27ac | 3.97E-07 | 1.81E-07 | 0.01406 |
| Rectal_Mucosa_Donor_29__H3K36me3 | 8.81E-07 | 4.04E-07 | 0.01462 |
| Aorta_ENTEX__H3K36me3 | 4.96E-07 | 2.28E-07 | 0.01481 |
| Cortex_derived_primary_cultured_neurospheres__H3K4me3 | 2.23E-06 | 1.03E-06 | 0.01535 |
| Fetal_Lung__H3K36me3 | 1.14E-06 | 5.41E-07 | 0.01778 |
| Brain_Hippocampus_Middle__H3K4me3 | 1.51E-06 | 7.25E-07 | 0.01863 |
| Mammary_ENTEX__H3K4me1 | 2.70E-06 | 1.31E-06 | 0.01993 |
| Aorta_ENTEX__H3K4me1 | 4.01E-07 | 1.95E-07 | 0.02011 |
| Rectal_Mucosa_Donor_31__H3K36me3 | 7.43E-07 | 3.81E-07 | 0.02551 |
| Foreskin_Keratinocyte_Primary_Cells_skin02__H3K4me1 | 1.27E-06 | 6.51E-07 | 0.0258 |
| NHLF_Lung_Fibroblast_Primary_Cells__H3K36me3 | 1.28E-06 | 6.71E-07 | 0.02846 |
| Foreskin_Keratinocyte_Primary_Cells_skin02__DNase | 1.21E-06 | 6.38E-07 | 0.02937 |
| Fetal_Muscle_Trunk__H3K36me3 | 7.46E-07 | 3.95E-07 | 0.02945 |
| Foreskin_Keratinocyte_Primary_Cells_skin03__H3K27ac | 8.56E-07 | 4.56E-07 | 0.03026 |
| NHDF-Ad_Adult_Dermal_Fibroblast_Primary_Cells__H3K4me1 | 7.55E-07 | 4.11E-07 | 0.03307 |
| Brain_Anterior_Caudate__H3K4me3 | 1.48E-06 | 8.12E-07 | 0.03408 |
| Nerve-Tibial_ENTEX__H3K36me3 | 5.91E-07 | 3.25E-07 | 0.03439 |
| Foreskin_Keratinocyte_Primary_Cells_skin03__H3K4me1 | 6.16E-07 | 3.40E-07 | 0.03525 |
| Osteoblast_Primary_Cells__H3K36me3 | 6.23E-07 | 3.46E-07 | 0.03596 |
| Artery-Coronary_ENTEX__H3K27ac | 5.88E-07 | 3.28E-07 | 0.03625 |
| Brain_Dorsolateral_Prefrontal_Cortex__H3K4me3 | 2.00E-06 | 1.13E-06 | 0.03891 |
| Esophagus__H3K4me1 | 1.01E-06 | 5.87E-07 | 0.04294 |
| Brain_Cingulate_Gyrus__H3K4me3 | 1.57E-06 | 9.30E-07 | 0.04594 |
| Osteoblast_Primary_Cells__H3K27ac | 6.61E-07 | 3.99E-07 | 0.04891 |
| Lung__H3K27ac | 7.18E-07 | 4.50E-07 | 0.05516 |
| Osteoblast_Primary_Cells__H3K4me3 | 1.58E-06 | 9.90E-07 | 0.05517 |
| Colon_Smooth_Muscle__H3K4me1 | 4.58E-07 | 2.88E-07 | 0.05577 |
| Colonic_Mucosa__H3K4me1 | 8.37E-07 | 5.30E-07 | 0.05715 |
| Prostate_ENTEX__H3K27ac | 6.09E-07 | 3.87E-07 | 0.05785 |
| Primary_hematopoietic_stem_cells_G-CSF-mobilized_Female__H3K4me3 | 1.62E-06 | 1.03E-06 | 0.05815 |
| NHEK-Epidermal_Keratinocyte_Primary_Cells__DNase | 1.19E-06 | 7.68E-07 | 0.0612 |
| Rectal_Smooth_Muscle__H3K4me1 | 6.35E-07 | 4.13E-07 | 0.06198 |
| Fetal_Kidney__H3K36me3 | 1.71E-06 | 1.13E-06 | 0.06485 |
| HMEC_Mammary_Epithelial_Primary_Cells__H3K27ac | 6.70E-07 | 4.46E-07 | 0.06622 |
| NHLF_Lung_Fibroblast_Primary_Cells__H3K27ac | 6.98E-07 | 4.71E-07 | 0.06911 |
| Vagina_ENTEX__H3K27ac | 5.53E-07 | 3.74E-07 | 0.06932 |
| Brain_Substantia_Nigra__H3K4me3 | 1.44E-06 | 9.76E-07 | 0.06955 |
| HMEC_Mammary_Epithelial_Primary_Cells__H3K9ac | 1.29E-06 | 8.80E-07 | 0.07096 |
| Brain_Germinal_Matrix__H3K4me3 | 2.58E-06 | 1.76E-06 | 0.07096 |
| Fetal_Muscle_Trunk__DNase | 9.46E-07 | 6.47E-07 | 0.0719 |
| Fetal_Muscle_Leg__DNase | 9.44E-07 | 6.47E-07 | 0.07231 |
| Osteoblast_Primary_Cells__H3K4me1 | 4.81E-07 | 3.30E-07 | 0.07258 |
| Artery-Tibial_ENTEX__H3K4me1 | 1.23E-06 | 8.44E-07 | 0.07318 |
| Foreskin_Fibroblast_Primary_Cells_skin01__H3K27ac | 4.76E-07 | 3.31E-07 | 0.07533 |
| NHDF-Ad_Adult_Dermal_Fibroblast_Primary_Cells__H3K9ac | 1.64E-06 | 1.15E-06 | 0.0767 |
| Fetal_Brain_Female__H3K4me3 | 1.19E-06 | 8.43E-07 | 0.07815 |
| Fetal_Lung__H3K4me1 | 6.31E-07 | 4.46E-07 | 0.07845 |
| Nerve-Tibial_ENTEX__H3K4me3 | 2.33E-06 | 1.68E-06 | 0.08232 |
| Brain_Angular_Gyrus__H3K9ac | 8.75E-07 | 6.34E-07 | 0.08382 |
| Breast_variant_Human_Mammary_Epithelial_Cells_(vHMEC)__DNase | 1.41E-06 | 1.02E-06 | 0.084 |
| Adipose_Nuclei__H3K36me3 | 4.93E-07 | 3.61E-07 | 0.08576 |
| Aorta__H3K27ac | 6.37E-07 | 4.73E-07 | 0.08912 |
| Breast_variant_Human_Mammary_Epithelial_Cells_(vHMEC)__H3K4me1 | 4.62E-07 | 3.46E-07 | 0.09064 |
| Brain_Inferior_Temporal_Lobe__H3K4me3 | 1.12E-06 | 8.43E-07 | 0.09195 |
| Brain_Inferior_Temporal_Lobe__H3K9ac | 8.07E-07 | 6.08E-07 | 0.09221 |
| Aorta__H3K4me1 | 9.48E-07 | 7.19E-07 | 0.09361 |
| Pancreas__H3K4me3 | 1.43E-06 | 1.09E-06 | 0.09464 |
| Colon-TV_ENTEX__H3K36me3 | 5.24E-07 | 4.02E-07 | 0.09637 |
| NHEK-Epidermal_Keratinocyte_Primary_Cells__H3K4me3 | 1.71E-06 | 1.32E-06 | 0.0989 |
| Artery-Coronary_ENTEX__H3K4me3 | 7.11E-07 | 5.69E-07 | 0.10584 |
| Pancreas__H3K27ac | 1.21E-06 | 9.68E-07 | 0.10628 |
| NHDF-Ad_Adult_Dermal_Fibroblast_Primary_Cells__H3K36me3 | 1.11E-06 | 9.09E-07 | 0.11081 |
| Fetal_Lung__DNase | 1.20E-06 | 9.81E-07 | 0.11121 |
| Pancreatic_Islets__H3K27ac | 1.29E-06 | 1.06E-06 | 0.11148 |
| Foreskin_Fibroblast_Primary_Cells_skin02__H3K27ac | 4.93E-07 | 4.04E-07 | 0.11159 |
| Colon_Smooth_Muscle__H3K36me3 | 5.72E-07 | 4.70E-07 | 0.11206 |
| Rectal_Smooth_Muscle__H3K36me3 | 7.98E-07 | 6.61E-07 | 0.1136 |
| Brain_Angular_Gyrus__H3K4me3 | 1.29E-06 | 1.08E-06 | 0.11537 |
| Vagina_ENTEX__H3K4me3 | 1.53E-06 | 1.28E-06 | 0.11538 |
| Colonic_Mucosa__H3K36me3 | 6.87E-07 | 5.76E-07 | 0.11624 |
| Mammary_ENTEX__H3K4me3 | 1.12E-06 | 9.69E-07 | 0.12359 |
| Duodenum_Mucosa__H3K36me3 | 3.51E-07 | 3.04E-07 | 0.12375 |
| Fetal_Muscle_Leg__H3K27ac | 5.55E-07 | 4.82E-07 | 0.12478 |
| Primary_T_helper_17_cells_PMA-I_stimulated__H3K4me3 | 9.77E-07 | 8.53E-07 | 0.12616 |
| Fetal_Muscle_Trunk__H3K27ac | 5.81E-07 | 5.11E-07 | 0.1278 |
| HMEC_Mammary_Epithelial_Primary_Cells__H3K4me1 | 4.42E-07 | 3.89E-07 | 0.12801 |
| Foreskin_Keratinocyte_Primary_Cells_skin03__H3K4me3 | 8.69E-07 | 7.77E-07 | 0.13168 |
| NHLF_Lung_Fibroblast_Primary_Cells__H3K9ac | 1.61E-06 | 1.47E-06 | 0.1361 |
| Fetal_Brain_Male__H3K4me1 | 2.77E-07 | 2.56E-07 | 0.13967 |
| Placenta_Amnion__H3K36me3 | 4.24E-07 | 3.93E-07 | 0.14027 |
| Primary_T_helper_17_cells_PMA-I_stimulated__H3K27ac | 5.96E-07 | 5.54E-07 | 0.14093 |
| Primary_hematopoietic_stem_cells_G-CSF-mobilized_Female__H3K4me1 | 2.65E-07 | 2.46E-07 | 0.14124 |
| Prostate_ENTEX__H3K4me3 | 2.02E-06 | 1.88E-06 | 0.14181 |
| Foreskin_Keratinocyte_Primary_Cells_skin02__H3K4me3 | 1.24E-06 | 1.17E-06 | 0.14349 |
| Fetal_Brain_Male__H3K36me3 | 1.16E-06 | 1.09E-06 | 0.14349 |
| Nerve-Tibial_ENTEX__H3K4me1 | 2.68E-07 | 2.55E-07 | 0.14723 |
| Primary_hematopoietic_stem_cells_G-CSF-mobilized_Female__H3K27ac | 4.07E-07 | 3.88E-07 | 0.14743 |
| Colonic_Mucosa__H3K9ac | 1.04E-06 | 1.01E-06 | 0.15158 |
| Spleen__H3K4me1 | 3.78E-07 | 3.72E-07 | 0.15451 |
| Pancreas_ENTEX__H3K4me3 | 8.63E-07 | 8.57E-07 | 0.15705 |
| HMEC_Mammary_Epithelial_Primary_Cells__DNase | 8.15E-07 | 8.13E-07 | 0.1582 |
| Uterus_ENTEX__H3K27ac | 2.60E-07 | 2.63E-07 | 0.16139 |
| Brain_Anterior_Caudate__H3K9ac | 5.71E-07 | 5.84E-07 | 0.16418 |
| Brain_Substantia_Nigra__H3K9ac | 6.29E-07 | 6.51E-07 | 0.16704 |
| Stomach_Smooth_Muscle__H3K4me3 | 6.05E-07 | 6.32E-07 | 0.16892 |
| Ovary__H3K4me3 | 2.16E-06 | 2.26E-06 | 0.1694 |
| Fetal_Intestine_Large__H3K36me3 | 3.82E-07 | 4.02E-07 | 0.17078 |
| Breast_variant_Human_Mammary_Epithelial_Cells_(vHMEC)__H3K36me3 | 2.12E-07 | 2.24E-07 | 0.17188 |
| Breast_Myoepithelial_Primary_Cells__H3K4me3 | 1.83E-06 | 1.94E-06 | 0.17317 |
| Primary_hematopoietic_stem_cells_G-CSF-mobilized_Male__H3K36me3 | 2.46E-07 | 2.63E-07 | 0.17437 |
| Aorta_ENTEX__H3K4me3 | 4.72E-07 | 5.05E-07 | 0.17461 |
| Psoas_Muscle__H3K36me3 | 6.76E-07 | 7.31E-07 | 0.17763 |
| Ovary__H3K27ac | 6.42E-07 | 6.95E-07 | 0.17796 |
| Nerve-Tibial_ENTEX__H3K27ac | 1.93E-07 | 2.11E-07 | 0.17955 |
| Ganglion_Eminence_derived_primary_cultured_neurospheres__H3K4me3 | 8.00E-07 | 8.81E-07 | 0.18172 |
| Adrenal_gland_ENTEX__H3K36me3 | 2.79E-07 | 3.13E-07 | 0.18622 |
| Esophagus__H3K27ac | 4.86E-07 | 5.49E-07 | 0.18839 |
| Stomach_ENTEX__H3K4me3 | 9.24E-07 | 1.05E-06 | 0.18859 |
| Heart-Atrial_ENTEX__H3K4me1 | 1.02E-06 | 1.18E-06 | 0.19208 |
| Ganglion_Eminence_derived_primary_cultured_neurospheres__H3K36me3 | 3.41E-07 | 3.95E-07 | 0.19369 |
| Esoph-Muscularis_ENTEX__H3K4me3 | 7.82E-07 | 9.18E-07 | 0.19705 |
| Brain_Cingulate_Gyrus__H3K9ac | 5.32E-07 | 6.25E-07 | 0.19736 |
| Lung__H3K4me1 | 3.89E-07 | 4.64E-07 | 0.20088 |
| Foreskin_Fibroblast_Primary_Cells_skin01__H3K4me3 | 5.14E-07 | 6.16E-07 | 0.20224 |
| Foreskin_Fibroblast_Primary_Cells_skin01__DNase | 5.73E-07 | 6.95E-07 | 0.20476 |
| Sigmoid_Colon__H3K36me3 | 4.03E-07 | 4.89E-07 | 0.20498 |
| HMEC_Mammary_Epithelial_Primary_Cells__H3K4me3 | 1.35E-06 | 1.65E-06 | 0.20738 |
| SI-Term-Ileum_ENTEX__H3K27ac | 2.08E-07 | 2.61E-07 | 0.21272 |
| Primary_hematopoietic_stem_cells_G-CSF-mobilized_Male__H3K4me1 | 2.34E-07 | 2.97E-07 | 0.21577 |
| Colon_Smooth_Muscle__H3K4me3 | 5.67E-07 | 7.35E-07 | 0.22036 |
| Colon-Sigm_ENTEX__H3K4me1 | 1.46E-06 | 1.89E-06 | 0.22074 |
| Colon-TV_ENTEX__H3K4me1 | 6.29E-07 | 8.27E-07 | 0.22332 |
| Adrenal_gland_ENTEX__H3K4me1 | 2.23E-07 | 2.96E-07 | 0.22606 |
| Colon_Smooth_Muscle__H3K27ac | 2.38E-07 | 3.21E-07 | 0.22922 |
| Colon-TV_ENTEX__H3K27ac | 2.12E-07 | 2.86E-07 | 0.23 |
| Stomach_Mucosa__H3K4me3 | 7.44E-07 | 1.02E-06 | 0.23301 |
| Testis_ENTEX__H3K27ac | 1.77E-07 | 2.44E-07 | 0.2342 |
| Placenta__H3K36me3 | 2.42E-07 | 3.34E-07 | 0.23452 |
| Esoph-Mucosa_ENTEX__H3K27ac | 2.84E-07 | 3.93E-07 | 0.23489 |
| Foreskin_Keratinocyte_Primary_Cells_skin03__H3K36me3 | 1.29E-07 | 1.82E-07 | 0.23819 |
| NHEK-Epidermal_Keratinocyte_Primary_Cells__H3K36me3 | 2.56E-07 | 3.67E-07 | 0.24311 |
| Fetal_Muscle_Leg__H3K4me1 | 2.47E-07 | 3.55E-07 | 0.24313 |
| Rectal_Smooth_Muscle__H3K4me3 | 7.42E-07 | 1.09E-06 | 0.24748 |
| Primary_hematopoietic_stem_cells_G-CSF-mobilized_Male__H3K4me3 | 4.80E-07 | 7.07E-07 | 0.24838 |
| Heart-LV_ENTEX__H3K36me3 | 2.34E-07 | 3.46E-07 | 0.24936 |
| Duodenum_Smooth_Muscle__H3K4me1 | 3.13E-07 | 4.73E-07 | 0.25396 |
| Esoph-Mucosa_ENTEX__H3K4me3 | 9.14E-07 | 1.41E-06 | 0.25841 |
| Psoas_Muscle__H3K4me3 | 9.66E-07 | 1.50E-06 | 0.25927 |
| Brain_Dorsolateral_Prefrontal_Cortex__H3K9ac | 5.58E-07 | 9.12E-07 | 0.27024 |
| Rectal_Mucosa_Donor_29__H3K27ac | 2.10E-07 | 3.46E-07 | 0.27144 |
| Foreskin_Fibroblast_Primary_Cells_skin02__H3K4me3 | 3.42E-07 | 5.69E-07 | 0.27363 |
| Liver__H3K4me1 | 1.57E-07 | 2.66E-07 | 0.27764 |
| Primary_monocytes_from_peripheral_blood__H3K27ac | 2.64E-07 | 4.57E-07 | 0.2816 |
| Primary_monocytes_from_peripheral_blood__DNase | 5.32E-07 | 9.32E-07 | 0.28424 |
| Spleen__H3K27ac | 3.00E-07 | 5.48E-07 | 0.29221 |
| Fetal_Muscle_Leg__H3K36me3 | 2.08E-07 | 3.85E-07 | 0.29475 |
| Rectal_Mucosa_Donor_31__H3K4me1 | 1.82E-07 | 3.38E-07 | 0.29532 |
| Brain_Germinal_Matrix__H3K4me1 | 4.30E-07 | 8.04E-07 | 0.29643 |
| Breast_variant_Human_Mammary_Epithelial_Cells_(vHMEC)__H3K4me3 | 6.16E-07 | 1.15E-06 | 0.2968 |
| Lung__H3K4me3 | 7.60E-07 | 1.44E-06 | 0.29929 |
| Brain_Hippocampus_Middle__H3K4me1 | 1.44E-07 | 2.76E-07 | 0.30045 |
| Adipose_Nuclei__H3K4me3 | 2.90E-07 | 5.55E-07 | 0.301 |
| Colonic_Mucosa__H3K27ac | 2.27E-07 | 4.48E-07 | 0.30628 |
| Rectal_Smooth_Muscle__H3K27ac | 1.65E-07 | 3.27E-07 | 0.30677 |
| Foreskin_Keratinocyte_Primary_Cells_skin02__H3K36me3 | 1.79E-07 | 3.72E-07 | 0.31496 |
| Primary_Natural_Killer_cells_from_peripheral_blood__H3K4me3 | 9.69E-07 | 2.04E-06 | 0.3176 |
| Adipose_Nuclei__H3K4me1 | 1.57E-07 | 3.37E-07 | 0.32025 |
| Breast_Myoepithelial_Primary_Cells__H3K36me3 | 1.10E-07 | 2.42E-07 | 0.3242 |
| Brain_Dorsolateral_Prefrontal_Cortex__H3K27ac | 1.61E-07 | 3.75E-07 | 0.33361 |
| Primary_T_helper_17_cells_PMA-I_stimulated__H3K4me1 | 1.47E-07 | 3.48E-07 | 0.33616 |
| HMEC_Mammary_Epithelial_Primary_Cells__H3K36me3 | 2.23E-07 | 5.33E-07 | 0.33783 |
| Pancreatic_Islets__H3K4me3 | 6.15E-07 | 1.48E-06 | 0.3389 |
| Pancreas__H3K4me1 | 1.47E-07 | 3.61E-07 | 0.34183 |
| Stomach_ENTEX__H3K36me3 | 1.09E-07 | 2.73E-07 | 0.34556 |
| Pancreatic_Islets__H3K9ac | 5.40E-07 | 1.36E-06 | 0.34608 |
| Ovary_ENTEX__H3K4me3 | 5.28E-07 | 1.40E-06 | 0.35273 |
| Breast_Myoepithelial_Primary_Cells__H3K9ac | 3.42E-07 | 9.22E-07 | 0.35552 |
| liver_ENTEX__H3K36me3 | 1.33E-07 | 3.71E-07 | 0.35966 |
| Fetal_Brain_Female__H3K4me1 | 1.56E-07 | 4.39E-07 | 0.3611 |
| Primary_T_helper_cells_PMA-I_stimulated__H3K4me3 | 1.99E-07 | 5.61E-07 | 0.36124 |
| Colonic_Mucosa__H3K4me3 | 4.89E-07 | 1.46E-06 | 0.3685 |
| Primary_T_helper_memory_cells_from_peripheral_blood_1__H3K36me3 | 1.65E-07 | 4.99E-07 | 0.37041 |
| Fetal_Stomach__H3K36me3 | 7.79E-08 | 2.36E-07 | 0.37073 |
| Primary_monocytes_from_peripheral_blood__H3K4me1 | 8.41E-08 | 2.57E-07 | 0.37175 |
| Stomach_Smooth_Muscle__H3K36me3 | 1.51E-07 | 4.64E-07 | 0.37222 |
| Foreskin_Melanocyte_Primary_Cells_skin03__H3K36me3 | 7.43E-08 | 2.31E-07 | 0.37367 |
| Small_Intestine__H3K36me3 | 2.73E-07 | 8.72E-07 | 0.37731 |
| Brain_Hippocampus_Middle__H3K27ac | 9.37E-08 | 3.13E-07 | 0.38227 |
| Primary_T_cells_from_peripheral_blood__H3K4me3 | 3.68E-07 | 1.30E-06 | 0.38847 |
| Fetal_Brain_Female__DNase | 2.04E-07 | 7.30E-07 | 0.38971 |
| Primary_hematopoietic_stem_cells_G-CSF-mobilized_Female__DNase | 1.99E-07 | 7.13E-07 | 0.39026 |
| Brain_Angular_Gyrus__H3K27ac | 8.33E-08 | 3.13E-07 | 0.39519 |
| Pancreas__DNase | 2.52E-07 | 9.50E-07 | 0.3954 |
| Aorta__H3K36me3 | 2.95E-07 | 1.13E-06 | 0.39718 |
| Adrenal_gland_ENTEX__H3K27ac | 5.01E-08 | 1.97E-07 | 0.39949 |
| Pancreatic_Islets__H3K4me1 | 1.03E-07 | 4.09E-07 | 0.40085 |
| Stomach_Mucosa__H3K36me3 | 1.73E-07 | 7.04E-07 | 0.40295 |
| Primary_T_regulatory_cells_from_peripheral_blood__H3K4me3 | 1.84E-07 | 7.96E-07 | 0.40887 |
| Foreskin_Fibroblast_Primary_Cells_skin02__H3K4me1 | 6.17E-08 | 2.88E-07 | 0.41512 |
| Fetal_Heart__H3K36me3 | 1.62E-07 | 7.64E-07 | 0.41628 |
| Brain_Substantia_Nigra__H3K4me1 | 6.36E-08 | 3.05E-07 | 0.41729 |
| Pancreas_ENTEX__H3K27ac | 4.35E-08 | 2.10E-07 | 0.41786 |
| Rectal_Mucosa_Donor_29__H3K4me1 | 1.03E-07 | 5.03E-07 | 0.41918 |
| Cortex_derived_primary_cultured_neurospheres__H3K36me3 | 8.04E-08 | 4.14E-07 | 0.42294 |
| Primary_Natural_Killer_cells_from_peripheral_blood__H3K27ac | 1.30E-07 | 6.92E-07 | 0.42525 |
| Spleen_ENTEX__H3K4me3 | 7.54E-08 | 4.09E-07 | 0.42688 |
| Primary_neutrophils_from_peripheral_blood__H3K4me1 | 3.89E-08 | 2.13E-07 | 0.42755 |
| Primary_hematopoietic_stem_cells__H3K36me3 | 9.65E-08 | 5.45E-07 | 0.42972 |
| Liver__H3K27ac | 4.19E-08 | 2.47E-07 | 0.43273 |
| Brain_Inferior_Temporal_Lobe__H3K4me1 | 6.29E-08 | 3.82E-07 | 0.43463 |
| Primary_monocytes_from_peripheral_blood__H3K4me3 | 1.69E-07 | 1.33E-06 | 0.44951 |
| Primary_neutrophils_from_peripheral_blood__H3K4me3 | 5.91E-08 | 5.20E-07 | 0.45481 |
| Duodenum_Smooth_Muscle__H3K4me3 | 5.38E-08 | 5.02E-07 | 0.45731 |
| Thyroid_gland_ENTEX__H3K27ac | 1.81E-08 | 1.72E-07 | 0.45798 |
| Mammary_ENTEX__H3K27ac | 2.63E-08 | 2.75E-07 | 0.46192 |
| Stomach_Smooth_Muscle__H3K4me1 | 2.69E-08 | 3.05E-07 | 0.46485 |
| Foreskin_Fibroblast_Primary_Cells_skin02__DNase | 6.58E-08 | 7.48E-07 | 0.46498 |
| Thyroid_gland_ENTEX__H3K4me1 | 1.80E-08 | 2.05E-07 | 0.465 |
| Breast_Myoepithelial_Primary_Cells__H3K4me1 | 1.36E-08 | 2.07E-07 | 0.47383 |
| Primary_T_helper_memory_cells_from_peripheral_blood_2__H3K36me3 | 3.02E-08 | 5.46E-07 | 0.47793 |
| Thyroid_gland_ENTEX__H3K36me3 | 1.51E-08 | 2.88E-07 | 0.47914 |
| Rectal_Mucosa_Donor_31__H3K9ac | 2.95E-08 | 5.72E-07 | 0.47943 |
| Brain_Anterior_Caudate__H3K27ac | 1.52E-08 | 3.15E-07 | 0.48073 |
| Primary_hematopoietic_stem_cells_G-CSF-mobilized_Female__H3K36me3 | 1.18E-08 | 2.73E-07 | 0.48283 |
| Brain_Cingulate_Gyrus__H3K27ac | 9.64E-09 | 3.13E-07 | 0.48769 |
| Brain_Anterior_Caudate__H3K4me1 | 8.90E-09 | 3.21E-07 | 0.48893 |
| Mammary_ENTEX__H3K36me3 | 4.46E-09 | 3.17E-07 | 0.49439 |
| Primary_T_cells_effector_memory_enriched_from_peripheral_blood__H3K36me3 | 1.78E-09 | 4.69E-07 | 0.49849 |
| Primary_monocytes_from_peripheral_blood__H3K36me3 | -1.79E-11 | 1.99E-07 | 0.50004 |
| Primary_T_cells_effector_memory_enriched_from_peripheral_blood__H3K27ac | -5.75E-09 | 5.10E-07 | 0.50449 |
| Brain_Dorsolateral_Prefrontal_Cortex__H3K4me1 | -4.36E-09 | 3.55E-07 | 0.5049 |
| liver_ENTEX__H3K4me1 | -1.22E-08 | 9.09E-07 | 0.50535 |
| Adipose_Nuclei__H3K9ac | -1.65E-08 | 1.00E-06 | 0.50655 |
| Stomach_Mucosa__H3K4me1 | -3.81E-09 | 2.13E-07 | 0.50715 |
| Lung_ENTEX__H3K4me1 | -5.35E-09 | 2.78E-07 | 0.50767 |
| Fetal_Muscle_Trunk__H3K4me1 | -8.71E-09 | 2.94E-07 | 0.5118 |
| Lung_ENTEX__H3K4me3 | -4.07E-08 | 1.16E-06 | 0.51403 |
| Primary_T_helper_memory_cells_from_peripheral_blood_1__H3K27ac | -2.79E-08 | 5.28E-07 | 0.52103 |
| Uterus_ENTEX__H3K4me3 | -9.00E-08 | 1.56E-06 | 0.52299 |
| Fetal_Intestine_Small__H3K36me3 | -1.58E-08 | 2.71E-07 | 0.52319 |
| Brain_Substantia_Nigra__H3K36me3 | -3.89E-08 | 6.54E-07 | 0.5237 |
| Pancreas_ENTEX__H3K4me1 | -1.23E-08 | 2.01E-07 | 0.52437 |
| Brain_Cingulate_Gyrus__H3K4me1 | -2.12E-08 | 3.06E-07 | 0.52762 |
| Foreskin_Fibroblast_Primary_Cells_skin01__H3K4me1 | -1.61E-08 | 2.20E-07 | 0.52918 |
| Primary_Natural_Killer_cells_from_peripheral_blood__H3K4me1 | -2.47E-08 | 3.15E-07 | 0.53125 |
| Brain_Substantia_Nigra__H3K27ac | -2.37E-08 | 2.57E-07 | 0.53677 |
| Brain_Germinal_Matrix__H3K36me3 | -4.00E-08 | 4.27E-07 | 0.53734 |
| Fetal_Intestine_Large__H3K27ac | -3.90E-08 | 3.78E-07 | 0.54108 |
| Stomach_Smooth_Muscle__H3K27ac | -2.97E-08 | 2.71E-07 | 0.5436 |
| Fetal_Lung__H3K9ac | -1.24E-07 | 1.08E-06 | 0.54576 |
| Primary_T_cells_effector_memory_enriched_from_peripheral_blood__H3K4me3 | -1.15E-07 | 9.22E-07 | 0.54957 |
| Foreskin_Fibroblast_Primary_Cells_skin02__H3K36me3 | -3.56E-08 | 2.76E-07 | 0.55145 |
| Lung_ENTEX__H3K27ac | -3.18E-08 | 2.38E-07 | 0.55307 |
| Adipose_Nuclei__H3K27ac | -3.75E-08 | 2.79E-07 | 0.55337 |
| Fetal_Brain_Male__DNase | -9.69E-08 | 6.62E-07 | 0.55817 |
| Brain_Angular_Gyrus__H3K4me1 | -5.90E-08 | 3.65E-07 | 0.56412 |
| Testis_ENTEX__H3K36me3 | -2.61E-07 | 1.59E-06 | 0.5652 |
| NHDF-Ad_Adult_Dermal_Fibroblast_Primary_Cells__H3K4me3 | -2.43E-07 | 1.48E-06 | 0.56543 |
| Rectal_Mucosa_Donor_31__H3K27ac | -6.37E-08 | 3.52E-07 | 0.57175 |
| Ovary__DNase | -1.50E-07 | 8.09E-07 | 0.57379 |
| Primary_neutrophils_from_peripheral_blood__H3K36me3 | -3.15E-08 | 1.68E-07 | 0.57416 |
| Primary_hematopoietic_stem_cells__H3K4me1 | -9.50E-08 | 5.08E-07 | 0.57418 |
| Colon-TV_ENTEX__H3K4me3 | -1.89E-07 | 1.00E-06 | 0.57447 |
| Primary_T_regulatory_cells_from_peripheral_blood__H3K4me1 | -7.13E-08 | 3.79E-07 | 0.57451 |
| NHLF_Lung_Fibroblast_Primary_Cells__H3K4me3 | -3.14E-07 | 1.56E-06 | 0.5798 |
| Ovary__H3K4me1 | -9.22E-08 | 4.40E-07 | 0.58293 |
| Fetal_Intestine_Small__H3K27ac | -8.63E-08 | 3.91E-07 | 0.58739 |
| Spleen_ENTEX__H3K4me1 | -1.59E-07 | 6.74E-07 | 0.59334 |
| Primary_hematopoietic_stem_cells_short_term_culture__H3K4me3 | -3.24E-07 | 1.36E-06 | 0.59412 |
| Stomach_Mucosa__H3K9ac | -1.48E-07 | 6.10E-07 | 0.5961 |
| Primary_T_helper_memory_cells_from_peripheral_blood_1__H3K4me3 | -2.16E-07 | 8.59E-07 | 0.59926 |
| Pancreatic_Islets__H3K36me3 | -1.62E-07 | 6.37E-07 | 0.60023 |
| Placenta_Amnion__H3K4me1 | -7.79E-08 | 2.99E-07 | 0.60276 |
| Primary_hematopoietic_stem_cells_short_term_culture__H3K36me3 | -7.34E-08 | 2.72E-07 | 0.60644 |
| Primary_T_helper_cells_from_peripheral_blood__H3K4me3 | -2.27E-07 | 8.34E-07 | 0.6074 |
| Liver__H3K36me3 | -6.00E-08 | 2.01E-07 | 0.61756 |
| Ovary_ENTEX__H3K36me3 | -4.42E-07 | 1.37E-06 | 0.62606 |
| Esoph-Muscularis_ENTEX__H3K27ac | -1.16E-07 | 3.57E-07 | 0.62766 |
| Primary_T_helper_cells_PMA-I_stimulated__H3K36me3 | -1.06E-07 | 3.21E-07 | 0.629 |
| Brain_Anterior_Caudate__H3K36me3 | -1.47E-07 | 4.44E-07 | 0.6295 |
| Primary_T_cells_from_peripheral_blood__H3K27ac | -1.51E-07 | 4.53E-07 | 0.63009 |
| Foreskin_Fibroblast_Primary_Cells_skin01__H3K36me3 | -6.89E-08 | 2.03E-07 | 0.63255 |
| Fetal_Intestine_Small__H3K4me3 | -6.49E-07 | 1.91E-06 | 0.63314 |
| Ovary__H3K36me3 | -1.51E-07 | 4.33E-07 | 0.6364 |
| Primary_hematopoietic_stem_cells_short_term_culture__H3K4me1 | -1.13E-07 | 3.23E-07 | 0.63682 |
| Fetal_Stomach__H3K4me1 | -1.07E-07 | 3.06E-07 | 0.63686 |
| Right_Atrium__H3K4me3 | -4.55E-07 | 1.29E-06 | 0.63825 |
| Fetal_Muscle_Leg__H3K4me3 | -9.28E-07 | 2.53E-06 | 0.64296 |
| Stomach_ENTEX__H3K4me1 | -1.00E-07 | 2.69E-07 | 0.64545 |
| Colon-Sigm_ENTEX__H3K4me3 | -6.50E-07 | 1.69E-06 | 0.64941 |
| Primary_T_cells_effector_memory_enriched_from_peripheral_blood__H3K4me1 | -1.78E-07 | 4.53E-07 | 0.65241 |
| SI-Term-Ileum_ENTEX__H3K4me3 | -2.59E-07 | 6.52E-07 | 0.65441 |
| Fetal_Intestine_Small__DNase | -4.97E-07 | 1.21E-06 | 0.65941 |
| Primary_T_regulatory_cells_from_peripheral_blood__H3K27ac | -1.96E-07 | 4.52E-07 | 0.66745 |
| Rectal_Smooth_Muscle__H3K9ac | -5.97E-07 | 1.34E-06 | 0.67241 |
| Fetal_Intestine_Large__H3K4me1 | -1.52E-07 | 3.34E-07 | 0.67535 |
| Primary_T_helper_naive_cells_from_peripheral_blood_1__H3K36me3 | -1.86E-07 | 3.98E-07 | 0.67992 |
| Lung_ENTEX__H3K36me3 | -1.25E-07 | 2.67E-07 | 0.6806 |
| Fetal_Adrenal_Gland__H3K4me1 | -1.59E-07 | 3.26E-07 | 0.68747 |
| Primary_T_helper_memory_cells_from_peripheral_blood_1__H3K4me1 | -1.43E-07 | 2.83E-07 | 0.69334 |
| Primary_T_killer_memory_cells_from_peripheral_blood__H3K36me3 | -2.60E-07 | 5.09E-07 | 0.69482 |
| Duodenum_Mucosa__H3K4me3 | -3.16E-07 | 6.19E-07 | 0.69549 |
| Fetal_Adrenal_Gland__H3K36me3 | -1.59E-07 | 3.10E-07 | 0.69585 |
| Thymus__H3K27ac | -3.49E-07 | 6.74E-07 | 0.69774 |
| Colon-Sigm_ENTEX__H3K36me3 | -2.57E-07 | 4.95E-07 | 0.69776 |
| Primary_mononuclear_cells_from_peripheral_blood__H3K4me3 | -7.11E-07 | 1.36E-06 | 0.69978 |
| Fetal_Intestine_Large__H3K4me3 | -1.04E-06 | 1.94E-06 | 0.70348 |
| Primary_mononuclear_cells_from_peripheral_blood__H3K9ac | -3.91E-07 | 7.30E-07 | 0.70412 |
| Primary_hematopoietic_stem_cells__H3K4me3 | -7.73E-07 | 1.44E-06 | 0.7043 |
| SI-Term-Ileum_ENTEX__H3K4me1 | -1.98E-07 | 3.65E-07 | 0.70615 |
| Aorta__H3K4me3 | -8.21E-07 | 1.51E-06 | 0.70636 |
| Primary_T_helper_memory_cells_from_peripheral_blood_2__H3K4me1 | -1.51E-07 | 2.79E-07 | 0.70641 |
| Esoph-GJ_ENTEX__H3K4me3 | -5.46E-07 | 1.00E-06 | 0.70671 |
| Colon-Sigm_ENTEX__H3K27ac | -2.00E-07 | 3.58E-07 | 0.71177 |
| SI-Term-Ileum_ENTEX__H3K36me3 | -3.76E-07 | 6.73E-07 | 0.71191 |
| Foreskin_Melanocyte_Primary_Cells_skin01__H3K36me3 | -3.73E-07 | 6.62E-07 | 0.71348 |
| Ganglion_Eminence_derived_primary_cultured_neurospheres__H3K4me1 | -2.51E-07 | 4.42E-07 | 0.71465 |
| Primary_T_helper_17_cells_PMA-I_stimulated__H3K36me3 | -2.11E-07 | 3.71E-07 | 0.71519 |
| Skeletal_Muscle_Male__H3K4me3 | -4.41E-07 | 7.62E-07 | 0.71857 |
| Psoas_Muscle__H3K4me1 | -3.04E-07 | 5.21E-07 | 0.72031 |
| Right_Atrium__H3K4me1 | -2.89E-07 | 4.91E-07 | 0.72169 |
| Primary_T_helper_naive_cells_from_peripheral_blood_1__H3K4me3 | -5.13E-07 | 8.67E-07 | 0.72306 |
| Duodenum_Smooth_Muscle__H3K27ac | -2.88E-07 | 4.85E-07 | 0.72325 |
| Liver__H3K4me3 | -2.59E-07 | 4.33E-07 | 0.72462 |
| Placenta__DNase | -3.30E-07 | 5.43E-07 | 0.72861 |
| Esoph-Muscularis_ENTEX__H3K36me3 | -1.81E-07 | 2.93E-07 | 0.7315 |
| Foreskin_Melanocyte_Primary_Cells_skin01__DNase | -3.86E-07 | 6.25E-07 | 0.73151 |
| Fetal_Muscle_Trunk__H3K4me3 | -1.65E-06 | 2.66E-06 | 0.73315 |
| Brain_Inferior_Temporal_Lobe__H3K27ac | -1.57E-07 | 2.52E-07 | 0.73428 |
| Rectal_Mucosa_Donor_29__H3K4me3 | -4.45E-07 | 7.00E-07 | 0.73778 |
| Primary_T_killer_naive_cells_from_peripheral_blood__H3K36me3 | -3.14E-07 | 4.93E-07 | 0.73788 |
| Cortex_derived_primary_cultured_neurospheres__H3K4me1 | -2.16E-07 | 3.37E-07 | 0.73927 |
| Spleen__H3K4me3 | -4.37E-07 | 6.81E-07 | 0.73943 |
| Sigmoid_Colon__H3K27ac | -2.73E-07 | 4.26E-07 | 0.73973 |
| Fetal_Intestine_Large__DNase | -6.05E-07 | 9.40E-07 | 0.74014 |
| Small_Intestine__H3K27ac | -3.06E-07 | 4.66E-07 | 0.74436 |
| Vagina_ENTEX__H3K36me3 | -6.66E-07 | 1.01E-06 | 0.74442 |
| Rectal_Mucosa_Donor_31__H3K4me3 | -5.47E-07 | 8.28E-07 | 0.7455 |
| Esoph-GJ_ENTEX__H3K4me1 | -2.35E-07 | 3.55E-07 | 0.74614 |
| Primary_T_helper_cells_from_peripheral_blood__H3K36me3 | -1.83E-07 | 2.68E-07 | 0.75303 |
| Primary_T_helper_cells_PMA-I_stimulated__H3K27ac | -1.85E-07 | 2.70E-07 | 0.7535 |
| Primary_Natural_Killer_cells_from_peripheral_blood__H3K36me3 | -2.33E-07 | 3.36E-07 | 0.75566 |
| Placenta_Amnion__H3K27ac | -5.97E-07 | 8.33E-07 | 0.76315 |
| Fetal_Stomach__H3K27ac | -4.53E-07 | 6.29E-07 | 0.7642 |
| Primary_T_killer_naive_cells_from_peripheral_blood__H3K4me3 | -8.67E-07 | 1.20E-06 | 0.76583 |
| Esophagus__H3K36me3 | -3.52E-07 | 4.69E-07 | 0.77343 |
| Fetal_Thymus__H3K4me1 | -1.64E-07 | 2.19E-07 | 0.77408 |
| Small_Intestine__DNase | -4.59E-07 | 6.09E-07 | 0.77457 |
| Primary_T_helper_memory_cells_from_peripheral_blood_2__H3K4me3 | -7.12E-07 | 9.42E-07 | 0.77522 |
| Fetal_Intestine_Small__H3K4me1 | -2.47E-07 | 3.26E-07 | 0.77565 |
| Rectal_Mucosa_Donor_29__H3K9ac | -6.27E-07 | 8.19E-07 | 0.77805 |
| Lung__H3K36me3 | -3.68E-07 | 4.76E-07 | 0.78028 |
| Primary_T_helper_naive_cells_from_peripheral_blood_1__H3K4me1 | -2.38E-07 | 3.04E-07 | 0.78316 |
| Fetal_Lung__H3K4me3 | -1.05E-06 | 1.32E-06 | 0.78575 |
| Fetal_Thymus__H3K27ac | -3.28E-07 | 3.96E-07 | 0.79649 |
| Primary_B_cells_from_peripheral_blood__H3K36me3 | -1.92E-07 | 2.29E-07 | 0.79974 |
| Fetal_Brain_Male__H3K4me3 | -2.03E-06 | 2.39E-06 | 0.80178 |
| Right_Ventricle__H3K4me1 | -2.96E-07 | 3.46E-07 | 0.80387 |
| Spleen_ENTEX__H3K27ac | -1.66E-07 | 1.92E-07 | 0.80558 |
| Sigmoid_Colon__H3K4me1 | -6.69E-07 | 7.70E-07 | 0.8074 |
| Esoph-GJ_ENTEX__H3K27ac | -3.02E-07 | 3.46E-07 | 0.80916 |
| Foreskin_Melanocyte_Primary_Cells_skin03__H3K4me1 | -2.38E-07 | 2.70E-07 | 0.81097 |
| Skeletal_Muscle_Female__H3K4me3 | -5.18E-07 | 5.86E-07 | 0.81163 |
| Duodenum_Mucosa__H3K9ac | -5.95E-07 | 6.68E-07 | 0.81356 |
| Skeletal_Muscle_Male__H3K36me3 | -3.46E-07 | 3.78E-07 | 0.81987 |
| liver_ENTEX__H3K27ac | -1.89E-07 | 2.06E-07 | 0.82078 |
| Gastric__H3K27ac | -5.26E-07 | 5.72E-07 | 0.82114 |
| Brain_Hippocampus_Middle__H3K36me3 | -4.01E-07 | 4.31E-07 | 0.82373 |
| skeletal_muscle_ENTEX__H3K4me3 | -3.88E-07 | 4.14E-07 | 0.82562 |
| Pancreas__H3K36me3 | -4.30E-07 | 4.58E-07 | 0.82611 |
| Brain_Inferior_Temporal_Lobe__H3K36me3 | -3.90E-07 | 4.04E-07 | 0.83242 |
| Gastric__H3K36me3 | -4.80E-07 | 4.91E-07 | 0.83567 |
| Primary_B_cells_from_cord_blood__H3K36me3 | -9.10E-07 | 9.26E-07 | 0.83712 |
| Adrenal_gland_ENTEX__H3K4me3 | -9.93E-07 | 1.00E-06 | 0.83983 |
| Primary_B_cells_from_peripheral_blood__H3K4me1 | -2.12E-07 | 2.12E-07 | 0.84137 |
| Brain_Angular_Gyrus__H3K36me3 | -5.19E-07 | 5.16E-07 | 0.8431 |
| Left_Ventricle__H3K4me1 | -3.54E-07 | 3.51E-07 | 0.84333 |
| Thymus__H3K4me3 | -1.33E-06 | 1.32E-06 | 0.84403 |
| Primary_hematopoietic_stem_cells_G-CSF-mobilized_Male__DNase | -7.73E-07 | 7.64E-07 | 0.84429 |
| Primary_mononuclear_cells_from_peripheral_blood__H3K4me1 | -9.00E-07 | 8.86E-07 | 0.84506 |
| Fetal_Stomach__H3K4me3 | -2.37E-06 | 2.33E-06 | 0.84574 |
| Skeletal_Muscle_Female__H3K27ac | -2.79E-07 | 2.74E-07 | 0.84606 |
| Skin_tissue_ENTEX__H3K4me3 | -1.20E-06 | 1.17E-06 | 0.84677 |
| Thyroid_gland_ENTEX__H3K4me3 | -9.53E-07 | 9.29E-07 | 0.8476 |
| Esophagus__H3K4me3 | -9.62E-07 | 9.31E-07 | 0.84926 |
| Stomach_Smooth_Muscle__H3K9ac | -9.09E-07 | 8.73E-07 | 0.85106 |
| Brain_Cingulate_Gyrus__H3K36me3 | -5.54E-07 | 5.12E-07 | 0.86036 |
| Fetal_Brain_Female__H3K36me3 | -4.40E-07 | 3.99E-07 | 0.86497 |
| Primary_T_regulatory_cells_from_peripheral_blood__H3K36me3 | -2.91E-07 | 2.61E-07 | 0.86794 |
| Fetal_Thymus__H3K4me3 | -1.61E-06 | 1.42E-06 | 0.87141 |
| Gastric__H3K4me1 | -5.69E-07 | 4.99E-07 | 0.87292 |
| Prostate_ENTEX__H3K36me3 | -2.25E-06 | 1.94E-06 | 0.87627 |
| Fetal_Stomach__DNase | -1.42E-06 | 1.22E-06 | 0.87732 |
| Fetal_Thymus__H3K36me3 | -2.28E-07 | 1.96E-07 | 0.87747 |
| Foreskin_Melanocyte_Primary_Cells_skin03__H3K4me3 | -1.85E-06 | 1.58E-06 | 0.87866 |
| Thymus__H3K36me3 | -6.09E-07 | 5.21E-07 | 0.87867 |
| Stomach_ENTEX__H3K27ac | -2.72E-07 | 2.31E-07 | 0.88051 |
| Duodenum_Mucosa__H3K4me1 | -4.07E-07 | 3.45E-07 | 0.88058 |
| Placenta__H3K4me3 | -1.85E-06 | 1.57E-06 | 0.8815 |
| Foreskin_Melanocyte_Primary_Cells_skin01__H3K4me3 | -1.69E-06 | 1.43E-06 | 0.88169 |
| Primary_T_killer_naive_cells_from_peripheral_blood__H3K4me1 | -2.94E-07 | 2.47E-07 | 0.88271 |
| Colon_Smooth_Muscle__H3K9ac | -7.99E-07 | 6.70E-07 | 0.88351 |
| Primary_T_helper_cells_from_peripheral_blood__H3K27ac | -5.69E-07 | 4.75E-07 | 0.88463 |
| Primary_T_helper_cells_PMA-I_stimulated__H3K4me1 | -2.77E-07 | 2.30E-07 | 0.88548 |
| Spleen_ENTEX__H3K36me3 | -2.33E-07 | 1.90E-07 | 0.88984 |
| Skeletal_Muscle_Female__H3K4me1 | -2.94E-07 | 2.40E-07 | 0.88998 |
| Brain_Dorsolateral_Prefrontal_Cortex__H3K36me3 | -6.26E-07 | 5.10E-07 | 0.89022 |
| Primary_mononuclear_cells_from_peripheral_blood__H3K27ac | -1.07E-06 | 8.60E-07 | 0.89312 |
| Foreskin_Melanocyte_Primary_Cells_skin01__H3K4me1 | -1.28E-06 | 1.00E-06 | 0.8987 |
| Fetal_Heart__H3K9ac | -5.39E-07 | 4.22E-07 | 0.89917 |
| Fetal_Adrenal_Gland__DNase | -9.77E-07 | 7.54E-07 | 0.90257 |
| Skeletal_Muscle_Female__H3K36me3 | -4.12E-07 | 3.17E-07 | 0.90284 |
| Fetal_Adrenal_Gland__H3K27ac | -4.32E-07 | 3.28E-07 | 0.90654 |
| Thymus__H3K4me1 | -5.46E-07 | 4.13E-07 | 0.9069 |
| Primary_T_cells_from_cord_blood__H3K36me3 | -7.46E-07 | 5.64E-07 | 0.90695 |
| skeletal_muscle_ENTEX__H3K36me3 | -2.50E-07 | 1.87E-07 | 0.90881 |
| Small_Intestine__H3K4me1 | -6.94E-07 | 5.18E-07 | 0.9097 |
| Primary_T_cells_from_peripheral_blood__H3K36me3 | -3.64E-07 | 2.68E-07 | 0.91268 |
| Primary_T_helper_cells_from_peripheral_blood__H3K4me1 | -3.60E-07 | 2.65E-07 | 0.91287 |
| liver_ENTEX__H3K4me3 | -7.87E-07 | 5.76E-07 | 0.91413 |
| Right_Ventricle__H3K27ac | -4.50E-07 | 3.26E-07 | 0.91587 |
| Placenta__H3K4me1 | -4.48E-07 | 3.22E-07 | 0.91774 |
| Liver__H3K9ac | -6.31E-07 | 4.48E-07 | 0.92052 |
| Skeletal_Muscle_Female__H3K9ac | -7.60E-07 | 5.39E-07 | 0.92085 |
| Left_Ventricle__H3K4me3 | -1.80E-06 | 1.28E-06 | 0.92089 |
| Skeletal_Muscle_Male__H3K4me1 | -4.12E-07 | 2.91E-07 | 0.92144 |
| Primary_T_cells_from_peripheral_blood__H3K4me1 | -3.88E-07 | 2.72E-07 | 0.92326 |
| Right_Atrium__H3K27ac | -5.83E-07 | 4.08E-07 | 0.92361 |
| Primary_Natural_Killer_cells_from_peripheral_blood__DNase | -1.15E-06 | 8.05E-07 | 0.92381 |
| Small_Intestine__H3K4me3 | -2.60E-06 | 1.81E-06 | 0.92438 |
| Primary_T_helper_naive_cells_from_peripheral_blood_2__H3K4me3 | -1.10E-06 | 7.67E-07 | 0.92447 |
| Skeletal_Muscle_Male__H3K9ac | -6.81E-07 | 4.64E-07 | 0.9286 |
| Placenta_Amnion__H3K4me3 | -3.47E-06 | 2.36E-06 | 0.92924 |
| Fetal_Adrenal_Gland__H3K4me3 | -3.36E-06 | 2.27E-06 | 0.93076 |
| Sigmoid_Colon__H3K4me3 | -3.35E-06 | 2.24E-06 | 0.93271 |
| Esoph-Mucosa_ENTEX__H3K36me3 | -6.22E-07 | 4.08E-07 | 0.93645 |
| Primary_T_killer_naive_cells_from_peripheral_blood__H3K9ac | -1.55E-06 | 1.02E-06 | 0.93682 |
| Pancreas_ENTEX__H3K36me3 | -4.41E-07 | 2.87E-07 | 0.93754 |
| Placenta__H3K27ac | -4.05E-07 | 2.63E-07 | 0.93804 |
| Testis_ENTEX__H3K4me3 | -7.03E-07 | 4.54E-07 | 0.93934 |
| Foreskin_Melanocyte_Primary_Cells_skin01__H3K27ac | -1.86E-06 | 1.19E-06 | 0.94046 |
| Primary_T_killer_memory_cells_from_peripheral_blood__H3K4me1 | -4.88E-07 | 3.12E-07 | 0.94108 |
| Left_Ventricle__H3K36me3 | -6.64E-07 | 4.24E-07 | 0.9414 |
| Gastric__DNase | -1.40E-06 | 8.87E-07 | 0.94245 |
| Right_Ventricle__H3K4me3 | -1.52E-06 | 9.61E-07 | 0.94285 |
| Psoas_Muscle__DNase | -8.70E-07 | 5.47E-07 | 0.94415 |
| Primary_T_cells_from_cord_blood__H3K4me3 | -2.68E-06 | 1.65E-06 | 0.94711 |
| Psoas_Muscle__H3K27ac | -7.18E-07 | 4.43E-07 | 0.94747 |
| Right_Ventricle__H3K36me3 | -7.04E-07 | 4.29E-07 | 0.94966 |
| Primary_T_killer_naive_cells_from_peripheral_blood__H3K27ac | -9.90E-07 | 5.93E-07 | 0.95242 |
| Foreskin_Melanocyte_Primary_Cells_skin03__H3K27ac | -8.86E-07 | 5.26E-07 | 0.95393 |
| Spleen__H3K36me3 | -5.79E-07 | 3.34E-07 | 0.95832 |
| Primary_T_helper_naive_cells_from_peripheral_blood_2__H3K27ac | -6.19E-07 | 3.51E-07 | 0.96133 |
| Primary_T_cells_from_cord_blood__H3K4me1 | -7.37E-07 | 4.16E-07 | 0.96172 |
| Fetal_Kidney__H3K4me1 | -1.37E-06 | 7.72E-07 | 0.9619 |
| Fetal_Thymus__DNase | -1.12E-06 | 6.00E-07 | 0.96899 |
| Fetal_Heart__H3K4me1 | -3.66E-07 | 1.96E-07 | 0.96906 |
| Primary_T_killer_memory_cells_from_peripheral_blood__H3K4me3 | -1.74E-06 | 9.20E-07 | 0.97047 |
| Heart-LV_ENTEX__H3K4me1 | -7.44E-07 | 3.91E-07 | 0.97129 |
| Fetal_Heart__DNase | -8.42E-07 | 4.40E-07 | 0.97222 |
| Heart-Atrial_ENTEX__H3K36me3 | -5.74E-07 | 2.93E-07 | 0.97476 |
| Primary_B_cells_from_cord_blood__H3K4me1 | -5.37E-07 | 2.71E-07 | 0.97621 |
| Heart-LV_ENTEX__H3K27ac | -6.15E-07 | 3.10E-07 | 0.97651 |
| Fetal_Kidney__H3K9ac | -4.14E-06 | 2.07E-06 | 0.97703 |
| Heart-LV_ENTEX__H3K4me3 | -1.88E-06 | 9.41E-07 | 0.97704 |
| Primary_T_cells_from_peripheral_blood__DNase | -1.21E-06 | 5.93E-07 | 0.9797 |
| Primary_T_helper_naive_cells_from_peripheral_blood_2__H3K4me1 | -4.70E-07 | 2.28E-07 | 0.98029 |
| Primary_B_cells_from_peripheral_blood__H3K27ac | -8.28E-07 | 3.94E-07 | 0.98216 |
| Primary_T_helper_naive_cells_from_peripheral_blood_1__H3K27ac | -1.30E-06 | 6.18E-07 | 0.98219 |
| Fetal_Kidney__DNase | -1.38E-06 | 6.44E-07 | 0.98392 |
| Gastric__H3K4me3 | -2.26E-06 | 1.05E-06 | 0.98471 |
| Primary_T_helper_naive_cells_from_peripheral_blood__H3K9ac | -1.95E-06 | 8.94E-07 | 0.98539 |
| Primary_B_cells_from_peripheral_blood__H3K4me3 | -2.43E-06 | 1.09E-06 | 0.98689 |
| Primary_T_helper_naive_cells_from_peripheral_blood_2__H3K36me3 | -6.98E-07 | 3.12E-07 | 0.98748 |
| skeletal_muscle_ENTEX__H3K4me1 | -3.68E-07 | 1.64E-07 | 0.98766 |
| Primary_B_cells_from_cord_blood__H3K4me3 | -3.70E-06 | 1.62E-06 | 0.98889 |
| Primary_T_cells_from_cord_blood__DNase | -2.29E-06 | 1.00E-06 | 0.9891 |
| Primary_B_cells_from_peripheral_blood__DNase | -1.26E-06 | 5.48E-07 | 0.98941 |
| Fetal_Kidney__H3K4me3 | -4.50E-06 | 1.92E-06 | 0.99052 |
| Heart-Atrial_ENTEX__H3K4me3 | -2.68E-06 | 1.14E-06 | 0.99076 |
| Primary_T_killer_memory_cells_from_peripheral_blood__H3K27ac | -1.60E-06 | 6.77E-07 | 0.99082 |
| Fetal_Heart__H3K4me3 | -1.71E-06 | 6.95E-07 | 0.99315 |
| skeletal_muscle_ENTEX__H3K27ac | -3.29E-07 | 1.33E-07 | 0.99328 |
| Primary_mononuclear_cells_from_peripheral_blood__H3K36me3 | -7.72E-07 | 2.89E-07 | 0.99624 |
| Primary_T_helper_memory_cells_from_peripheral_blood_2__H3K27ac | -1.06E-06 | 3.72E-07 | 0.99787 |
| Left_Ventricle__H3K27ac | -7.77E-07 | 2.52E-07 | 0.99897 |
| Right_Atrium__H3K36me3 | -2.36E-06 | 7.57E-07 | 0.99908 |
| Heart-Atrial_ENTEX__H3K27ac | -7.41E-07 | 1.81E-07 | 0.99998 |
| Esoph-Muscularis_ENTEX__H3K4me1 | -1.09E-06 | 2.57E-07 | 0.99999 |

**Table S6: LDSC-SEG results for 489 tissue chromatin annotations.** None were significant after FDR correction (FDR < 0.1).

| Tissue | # Genes | Beta | s.e. | $P_{GP,T}$ |
| --- | --- | --- | --- | --- |
| Cells_Transformed_fibroblasts | 16517 | 0.025 | 0.008 | 0.001 |
| Nerve_Tibial | 16517 | 0.034 | 0.012 | 0.002 |
| Adipose_Subcutaneous | 16517 | 0.029 | 0.012 | 0.009 |
| Breast_Mammary_Tissue | 16517 | 0.033 | 0.014 | 0.012 |
| Cervix_Ectocervix | 16517 | 0.03 | 0.015 | 0.021 |
| Artery_Tibial | 16517 | 0.023 | 0.012 | 0.024 |
| Artery_Aorta | 16517 | 0.023 | 0.012 | 0.025 |
| Vagina | 16517 | 0.021 | 0.013 | 0.048 |
| Small_Intestine_Terminal_Ileum | 16517 | 0.015 | 0.009 | 0.055 |
| Uterus | 16517 | 0.018 | 0.013 | 0.078 |
| Minor_Salivary_Gland | 16517 | 0.016 | 0.011 | 0.08 |
| Cervix_Endocervix | 16517 | 0.019 | 0.014 | 0.081 |
| Adipose_Visceral_Omentum | 16517 | 0.018 | 0.013 | 0.081 |
| Skin_Sun_Exposed_Lower_leg | 16517 | 0.012 | 0.009 | 0.083 |
| Artery_Coronary | 16517 | 0.018 | 0.014 | 0.088 |
| Esophagus_Mucosa | 16517 | 0.011 | 0.008 | 0.095 |
| Fallopian_Tube | 16517 | 0.018 | 0.014 | 0.097 |
| Skin_Not_Sun_Exposed_Suprapubic | 16517 | 0.011 | 0.009 | 0.101 |
| Thyroid | 16517 | 0.014 | 0.011 | 0.109 |
| Whole_Blood | 16517 | 0.008 | 0.006 | 0.113 |
| Prostate | 16517 | 0.016 | 0.013 | 0.115 |
| Cells_EBV-transformed_lymphocytes | 16517 | 0.006 | 0.006 | 0.14 |
| Spleen | 16517 | 0.008 | 0.008 | 0.148 |
| Colon_Transverse | 16517 | 0.012 | 0.012 | 0.154 |
| Lung | 16517 | 0.01 | 0.011 | 0.164 |
| Muscle_Skeletal | 16517 | 0.006 | 0.008 | 0.232 |
| Bladder | 16517 | 0.009 | 0.014 | 0.252 |
| Esophagus_Gastroesophageal_Junction | 16517 | 0.008 | 0.015 | 0.286 |
| Colon_Sigmoid | 16517 | 0.008 | 0.014 | 0.292 |
| Ovary | 16517 | 0.004 | 0.011 | 0.368 |
| Esophagus_Muscularis | 16517 | 0.004 | 0.014 | 0.393 |
| Kidney_Cortex | 16517 | -0.002 | 0.011 | 0.592 |
| Testis | 16517 | -0.002 | 0.006 | 0.603 |
| Pancreas | 16517 | -0.006 | 0.01 | 0.725 |
| Brain_Spinal_cord_cervical_c-1 | 16517 | -0.006 | 0.01 | 0.731 |
| Stomach | 16517 | -0.008 | 0.014 | 0.736 |
| Brain_Cerebellum | 16517 | -0.005 | 0.007 | 0.767 |
| Adrenal_Gland | 16517 | -0.008 | 0.011 | 0.784 |
| Brain_Cerebellar_Hemisphere | 16517 | -0.006 | 0.007 | 0.804 |
| Liver | 16517 | -0.006 | 0.007 | 0.821 |
| Brain_Hippocampus | 16517 | -0.018 | 0.009 | 0.976 |
| Heart_Atrial_Appendage | 16517 | -0.021 | 0.011 | 0.978 |
| Brain_Substantia_nigra | 16517 | -0.02 | 0.01 | 0.98 |
| Brain_Amygdala | 16517 | -0.019 | 0.009 | 0.982 |
| Brain_Cortex | 16517 | -0.017 | 0.008 | 0.985 |
| Heart_Left_Ventricle | 16517 | -0.022 | 0.01 | 0.987 |
| Brain_Anterior_cingulate_cortex_BA24 | 16517 | -0.019 | 0.008 | 0.989 |
| Brain_Frontal_Cortex_BA9 | 16517 | -0.018 | 0.008 | 0.991 |
| Brain_Hypothalamus | 16517 | -0.021 | 0.009 | 0.991 |
| Brain_Caudate_basal_ganglia | 16517 | -0.021 | 0.009 | 0.992 |
| Brain_Putamen_basal_ganglia | 16517 | -0.022 | 0.009 | 0.992 |
| Brain_Nucleus_accumbens_basal_ganglia | 16517 | -0.021 | 0.008 | 0.993 |
| Pituitary | 16517 | -0.029 | 0.01 | 0.999 |

**Table S7: FUMA gene property analysis for 53 GTEx tissues.**

| Category | DEG Set | N_genes | N_overlap | $P_{DEG}$ |
| --- | --- | --- | --- | --- |
| DEG.up | Artery_Tibial | 1152 | 10 | 5.50E-05 |
| DEG.up | Artery_Aorta | 1197 | 10 | 7.80E-05 |
| DEG.up | Cervix_Endocervix | 82 | 2 | 9.65E-04 |
| DEG.twoside | Cervix_Endocervix | 82 | 2 | 9.65E-04 |
| DEG.up | Artery_Coronary | 983 | 7 | 1.92E-03 |
| DEG.up | Uterus | 1751 | 9 | 6.70E-03 |
| DEG.twoside | Skin_Not_Sun_Exposed_Suprapubic | 3636 | 15 | 6.71E-03 |
| DEG.down | Kidney_Cortex | 739 | 5 | 7.54E-03 |
| DEG.twoside | Vagina | 1841 | 9 | 9.46E-03 |
| DEG.twoside | Uterus | 2537 | 11 | 1.25E-02 |
| DEG.twoside | Skin_Sun_Exposed_Lower_leg | 3691 | 14 | 1.87E-02 |
| DEG.twoside | Artery_Aorta | 2688 | 11 | 1.93E-02 |
| DEG.twoside | Artery_Tibial | 3044 | 12 | 2.05E-02 |
| DEG.twoside | Artery_Coronary | 1782 | 8 | 2.18E-02 |
| DEG.up | Fallopian_Tube | 100 | 1 | 2.35E-02 |
| DEG.twoside | Fallopian_Tube | 100 | 1 | 2.35E-02 |
| DEG.up | Colon_Sigmoid | 960 | 5 | 2.47E-02 |
| DEG.up | Bladder | 267 | 2 | 2.53E-02 |
| DEG.twoside | Colon_Sigmoid | 2151 | 9 | 2.61E-02 |
| DEG.up | Skin_Not_Sun_Exposed_Suprapubic | 1853 | 8 | 2.73E-02 |
| DEG.down | Pituitary | 1560 | 7 | 2.82E-02 |
| DEG.up | Vagina | 1274 | 6 | 2.89E-02 |
| DEG.up | Brain_Substantia_nigra | 1583 | 7 | 3.05E-02 |
| DEG.up | Skin_Sun_Exposed_Lower_leg | 1899 | 8 | 3.14E-02 |
| DEG.twoside | Bladder | 295 | 2 | 3.26E-02 |
| DEG.up | Breast_Mammary_Tissue | 1355 | 6 | 3.86E-02 |
| DEG.down | Vagina | 567 | 3 | 4.47E-02 |
| DEG.twoside | Adipose_Subcutaneous | 2386 | 9 | 4.87E-02 |
| DEG.up | Adipose_Subcutaneous | 1433 | 6 | 4.98E-02 |
| DEG.twoside | Brain_Substantia_nigra | 5312 | 17 | 5.24E-02 |
| DEG.twoside | Esophagus_Mucosa | 4206 | 14 | 5.47E-02 |
| DEG.down | Skin_Not_Sun_Exposed_Suprapubic | 1783 | 7 | 5.55E-02 |
| DEG.twoside | Kidney_Cortex | 2160 | 8 | 6.32E-02 |
| DEG.up | Nerve_Tibial | 1867 | 7 | 6.92E-02 |
| DEG.down | Cells_EBV-transformed_lymphocytes | 1555 | 6 | 7.12E-02 |
| DEG.down | Liver | 4733 | 15 | 7.13E-02 |
| DEG.down | Esophagus_Mucosa | 2554 | 9 | 7.15E-02 |
| DEG.down | Small_Intestine_Terminal_Ileum | 968 | 4 | 7.74E-02 |
| DEG.twoside | Breast_Mammary_Tissue | 1925 | 7 | 7.98E-02 |
| DEG.twoside | Nerve_Tibial | 2635 | 9 | 8.47E-02 |
| DEG.up | Adipose_Visceral_Omentum | 1366 | 5 | 1.01E-01 |
| DEG.down | Prostate | 505 | 2 | 1.17E-01 |
| DEG.twoside | Brain_Hypothalamus | 5456 | 16 | 1.18E-01 |
| DEG.up | Lung | 2122 | 7 | 1.23E-01 |
| DEG.down | Skin_Sun_Exposed_Lower_leg | 1792 | 6 | 1.27E-01 |
| DEG.down | Pancreas | 5136 | 15 | 1.30E-01 |
| DEG.twoside | Esophagus_Muscularis | 2191 | 7 | 1.41E-01 |
| DEG.down | Stomach | 1173 | 4 | 1.43E-01 |
| DEG.up | Esophagus_Gastroesophageal_Junction | 865 | 3 | 1.48E-01 |
| DEG.down | Colon_Sigmoid | 1191 | 4 | 1.49E-01 |
| DEG.twoside | Adipose_Visceral_Omentum | 2251 | 7 | 1.57E-01 |
| DEG.up | Esophagus_Muscularis | 902 | 3 | 1.64E-01 |
| DEG.twoside | Brain_Cerebellar_Hemisphere | 5759 | 16 | 1.74E-01 |
| DEG.down | Brain_Hypothalamus | 3095 | 9 | 1.87E-01 |
| DEG.down | Esophagus_Muscularis | 1289 | 4 | 1.88E-01 |
| DEG.down | Adipose_Subcutaneous | 953 | 3 | 1.88E-01 |
| DEG.up | Brain_Hypothalamus | 2361 | 7 | 1.90E-01 |
| DEG.up | Esophagus_Mucosa | 1652 | 5 | 1.94E-01 |
| DEG.down | Colon_Transverse | 966 | 3 | 1.95E-01 |
| DEG.twoside | Pancreas | 5901 | 16 | 2.04E-01 |
| DEG.down | Brain_Cerebellar_Hemisphere | 2064 | 6 | 2.12E-01 |
| DEG.up | Brain_Spinal_cord_cervical_c-1 | 1376 | 4 | 2.25E-01 |
| DEG.twoside | Lung | 2859 | 8 | 2.28E-01 |
| DEG.up | Brain_Cerebellum | 3664 | 10 | 2.39E-01 |
| DEG.up | Brain_Cerebellar_Hemisphere | 3695 | 10 | 2.48E-01 |
| DEG.down | Brain_Putamen_basal_ganglia | 4103 | 11 | 2.53E-01 |
| DEG.down | Brain_Substantia_nigra | 3729 | 10 | 2.58E-01 |
| DEG.up | Brain_Cortex | 2570 | 7 | 2.59E-01 |
| DEG.up | Brain_Amygdala | 1854 | 5 | 2.74E-01 |
| DEG.down | Nerve_Tibial | 768 | 2 | 2.74E-01 |
| DEG.twoside | Brain_Cerebellum | 5806 | 15 | 2.81E-01 |
| DEG.down | Uterus | 786 | 2 | 2.86E-01 |
| DEG.up | Ovary | 2275 | 6 | 2.91E-01 |
| DEG.up | Brain_Frontal_Cortex_BA9 | 2664 | 7 | 2.92E-01 |
| DEG.twoside | Esophagus_Gastroesophageal_Junction | 1902 | 5 | 2.94E-01 |
| DEG.up | Brain_Anterior_cingulate_cortex_BA24 | 2294 | 6 | 2.98E-01 |
| DEG.up | Adrenal_Gland | 1940 | 5 | 3.10E-01 |
| DEG.down | Adrenal_Gland | 1562 | 4 | 3.11E-01 |
| DEG.twoside | Adrenal_Gland | 3502 | 9 | 3.11E-01 |
| DEG.twoside | Small_Intestine_Terminal_Ileum | 3128 | 8 | 3.18E-01 |
| DEG.down | Thyroid | 874 | 2 | 3.44E-01 |
| DEG.down | Adipose_Visceral_Omentum | 885 | 2 | 3.51E-01 |
| DEG.twoside | Liver | 6138 | 15 | 3.74E-01 |
| DEG.down | Spleen | 1338 | 3 | 3.93E-01 |
| DEG.twoside | Stomach | 2130 | 5 | 3.94E-01 |
| DEG.down | Breast_Mammary_Tissue | 570 | 1 | 3.94E-01 |
| DEG.down | Brain_Cerebellum | 2142 | 5 | 3.99E-01 |
| DEG.up | Spleen | 2950 | 7 | 4.00E-01 |
| DEG.down | Minor_Salivary_Gland | 969 | 2 | 4.06E-01 |
| DEG.down | Cells_Transformed_fibroblasts | 1773 | 4 | 4.13E-01 |
| DEG.twoside | Brain_Putamen_basal_ganglia | 5861 | 14 | 4.15E-01 |
| DEG.down | Brain_Caudate_basal_ganglia | 3413 | 8 | 4.22E-01 |
| DEG.twoside | Brain_Cortex | 5499 | 13 | 4.31E-01 |
| DEG.twoside | Cells_EBV-transformed_lymphocytes | 5515 | 13 | 4.36E-01 |
| DEG.up | Kidney_Cortex | 1421 | 3 | 4.39E-01 |
| DEG.twoside | Spleen | 4288 | 10 | 4.41E-01 |
| DEG.down | Esophagus_Gastroesophageal_Junction | 1037 | 2 | 4.50E-01 |
| DEG.down | Testis | 2257 | 5 | 4.50E-01 |
| DEG.twoside | Pituitary | 4749 | 11 | 4.57E-01 |
| DEG.down | Brain_Hippocampus | 3936 | 9 | 4.62E-01 |
| DEG.twoside | Ovary | 3587 | 8 | 4.86E-01 |
| DEG.up | Minor_Salivary_Gland | 1108 | 2 | 4.94E-01 |
| DEG.up | Brain_Hippocampus | 1958 | 4 | 5.02E-01 |
| DEG.up | Whole_Blood | 1556 | 3 | 5.11E-01 |
| DEG.twoside | Brain_Frontal_Cortex_BA9 | 5755 | 13 | 5.12E-01 |
| DEG.twoside | Colon_Transverse | 2419 | 5 | 5.21E-01 |
| DEG.down | Lung | 737 | 1 | 5.27E-01 |
| DEG.twoside | Prostate | 2457 | 5 | 5.37E-01 |
| DEG.up | Pancreas | 765 | 1 | 5.47E-01 |
| DEG.twoside | Brain_Hippocampus | 5894 | 13 | 5.55E-01 |
| DEG.down | Brain_Cortex | 2929 | 6 | 5.56E-01 |
| DEG.up | Testis | 5895 | 13 | 5.56E-01 |
| DEG.twoside | Minor_Salivary_Gland | 2077 | 4 | 5.57E-01 |
| DEG.down | Brain_Nucleus_accumbens_basal_ganglia | 3372 | 7 | 5.62E-01 |
| DEG.twoside | Brain_Caudate_basal_ganglia | 5521 | 12 | 5.71E-01 |
| DEG.up | Brain_Caudate_basal_ganglia | 2108 | 4 | 5.71E-01 |
| DEG.down | Artery_Coronary | 799 | 1 | 5.71E-01 |
| DEG.up | Small_Intestine_Terminal_Ileum | 2160 | 4 | 5.94E-01 |
| DEG.down | Whole_Blood | 4756 | 10 | 5.97E-01 |
| DEG.twoside | Testis | 8152 | 18 | 6.01E-01 |
| DEG.up | Brain_Putamen_basal_ganglia | 1758 | 3 | 6.11E-01 |
| DEG.down | Ovary | 1312 | 2 | 6.11E-01 |
| DEG.down | Heart_Atrial_Appendage | 3514 | 7 | 6.13E-01 |
| DEG.up | Heart_Atrial_Appendage | 866 | 1 | 6.16E-01 |
| DEG.down | Brain_Frontal_Cortex_BA9 | 3091 | 6 | 6.17E-01 |
| DEG.up | Brain_Nucleus_accumbens_basal_ganglia | 2258 | 4 | 6.35E-01 |
| DEG.down | Muscle_Skeletal | 4508 | 9 | 6.55E-01 |
| DEG.up | Stomach | 957 | 1 | 6.71E-01 |
| DEG.twoside | Whole_Blood | 6312 | 13 | 6.78E-01 |
| DEG.twoside | Brain_Amygdala | 5888 | 12 | 6.79E-01 |
| DEG.up | Colon_Transverse | 1453 | 2 | 6.81E-01 |
| DEG.twoside | Brain_Anterior_cingulate_cortex_BA24 | 5905 | 12 | 6.84E-01 |
| DEG.up | Prostate | 1952 | 3 | 6.95E-01 |
| DEG.down | Heart_Left_Ventricle | 5149 | 10 | 7.14E-01 |
| DEG.twoside | Brain_Nucleus_accumbens_basal_ganglia | 5630 | 11 | 7.26E-01 |
| DEG.twoside | Thyroid | 3433 | 6 | 7.31E-01 |
| DEG.twoside | Heart_Atrial_Appendage | 4380 | 8 | 7.45E-01 |
| DEG.up | Thyroid | 2559 | 4 | 7.46E-01 |
| DEG.up | Muscle_Skeletal | 1115 | 1 | 7.51E-01 |
| DEG.up | Cells_EBV-transformed_lymphocytes | 3960 | 7 | 7.53E-01 |
| DEG.twoside | Cells_Transformed_fibroblasts | 3995 | 7 | 7.62E-01 |
| DEG.down | Brain_Amygdala | 4034 | 7 | 7.72E-01 |
| DEG.down | Brain_Anterior_cingulate_cortex_BA24 | 3611 | 6 | 7.81E-01 |
| DEG.twoside | Brain_Spinal_cord_cervical_c-1 | 5450 | 10 | 7.89E-01 |
| DEG.up | Cells_Transformed_fibroblasts | 2222 | 3 | 7.90E-01 |
| DEG.twoside | Muscle_Skeletal | 5623 | 10 | 8.26E-01 |
| DEG.down | Artery_Tibial | 1892 | 2 | 8.41E-01 |
| DEG.down | Artery_Aorta | 1491 | 1 | 8.79E-01 |
| DEG.down | Brain_Spinal_cord_cervical_c-1 | 4074 | 6 | 8.80E-01 |
| DEG.twoside | Heart_Left_Ventricle | 5940 | 10 | 8.82E-01 |
| DEG.up | Pituitary | 3189 | 4 | 8.97E-01 |
| DEG.up | Heart_Left_Ventricle | 791 | 0 | 1.00E+00 |
| DEG.up | Liver | 1405 | 0 | 1.00E+00 |

**Table S8: FUMA DEG analysis for 53 GTEx tissues.** The up-regulated DEG sets for the tibial artery and the aorta artery tissues are significant ($P_{DEG}\leq{0.05}/{153}$).

| Study_Experiment | CellType | ObservedGenes | Beta | s.e. | P_GP,CT |
| --- | --- | --- | --- | --- | --- |
| MouseCellAtlas_Neonatal_Calvaria | Stromal_cell_Col1a1_high | 14459 | 0.47 | 0.117 | 3.14E-05 |
| MouseCellAtlas_Neonatal_Rib | Stromal_cell_Tnmd_high | 14459 | 0.403 | 0.109 | 1.15E-04 |
| MouseCellAtlas_all | Pancreas.Stromal_cell_Fn1_high | 14459 | 0.226 | 0.0629 | 1.62E-04 |
| MouseCellAtlas_Neonatal_all | Neonatal_Muscle.Skeletal_muscle_cell_Myl1_high | 14459 | 0.303 | 0.0859 | 2.12E-04 |
| MouseCellAtlas_Adult_all | Pancreas.Stromal_cell_Fn1_high | 14459 | 0.217 | 0.0618 | 2.18E-04 |
| MouseCellAtlas_Neonatal_all | Neonatal_Skin.Osteoblast_Ppic_high | 14459 | 0.435 | 0.129 | 3.73E-04 |
| MouseCellAtlas_Neonatal_all | Neonatal_Muscle.Tendon_stem.progenitor_cell | 14459 | 0.31 | 0.0919 | 3.75E-04 |
| DropViz_all_level2 | SN.Microglia_C1qb_Tmem119.9_2 | 14805 | 0.00886 | 0.00266 | 4.30E-04 |
| MouseCellAtlas_Neonatal_all | Neonatal_Skin.Stromal_cell_Gas6_high | 14459 | 0.376 | 0.113 | 4.34E-04 |
| DropViz_all_level1 | SN.Microglia | 14805 | 0.00953 | 0.00287 | 4.49E-04 |
| MouseCellAtlas_Neonatal_all | Neonatal_Muscle.Stromal_cell_Ndn_high | 14459 | 0.376 | 0.114 | 5.06E-04 |
| TabulaMuris_FACS_Tongue | basal_cell_of_epidermis | 13888 | 0.0762 | 0.0232 | 5.21E-04 |
| MouseCellAtlas_Neonatal_all | Neonatal_Muscle.Epithelial_cell | 14459 | 0.29 | 0.0887 | 5.47E-04 |
| DropViz_SN_level2 | Microglia_C1qb_Tmem119.9_2 | 14805 | 0.00861 | 0.0027 | 7.24E-04 |
| MouseCellAtlas_all | Mammary_Gland.Stromal_cell_Pi16_high | 14459 | 0.197 | 0.062 | 7.49E-04 |
| MouseCellAtlas_Neonatal_Rib | Stromal_cell_Acta1_high | 14459 | 0.319 | 0.101 | 7.73E-04 |
| MouseCellAtlas_Neonatal_all | Neonatal_Heart.Stromal_cell_Col3a1_high | 14459 | 0.456 | 0.145 | 8.21E-04 |
| MouseCellAtlas_Adult_all | Mammary_Gland.Stromal_cell_Pi16_high | 14459 | 0.192 | 0.0612 | 8.37E-04 |
| GSE104276_Human_Prefrontal_cortex_per_ages | GW10_Microglia | 17199 | 0.0124 | 0.00396 | 8.62E-04 |
| MouseCellAtlas_all | Mammary_Gland.Muscle_cell_Pi16_high | 14459 | 0.214 | 0.0689 | 9.33E-04 |
| MouseCellAtlas_Adult_all | Mammary_Gland.Muscle_cell_Pi16_high | 14459 | 0.21 | 0.0681 | 0.0010444 |
| DropViz_SN_level1 | Microglia | 14805 | 0.0095 | 0.00309 | 0.0010681 |
| MouseCellAtlas_Neonatal_all | Neonatal_Skin.Osteoblast_Dlk1_high | 14459 | 0.34 | 0.111 | 0.0010832 |
| MouseCellAtlas_all | Pancreas.Stromal_cell_Mfap4_high | 14459 | 0.168 | 0.0548 | 0.0011023 |
| DropViz_all_level2 | TH.Endothelial_Tip_Dcn_Nenf.6_2 | 14805 | 0.0115 | 0.00377 | 0.0011031 |
| MouseCellAtlas_Neonatal_Muscle | Skeletal_muscle_cell_Myl1_high | 14459 | 0.289 | 0.095 | 0.0011642 |
| Linnarsson_MouseBrainAtlas_level5 | ENT4 | 15140 | 0.0855 | 0.0281 | 0.0011868 |
| TabulaMuris_FACS_Mammary | stromal_cell | 13888 | 0.028 | 0.00931 | 0.0013357 |
| MouseCellAtlas_Adult_all | Pancreas.Stromal_cell_Mfap4_high | 14459 | 0.161 | 0.0538 | 0.0013774 |
| MouseCellAtlas_all | Neonatal_Muscle.Skeletal_muscle_cell_Myl1_high | 14459 | 0.206 | 0.0689 | 0.0013869 |
| DropViz_all_level1 | TH.Endothelial_tip | 14805 | 0.0159 | 0.00531 | 0.0014052 |
| MouseCellAtlas_Neonatal_all | Neonatal_Heart.Stromal_cell_Fmod_high | 14459 | 0.362 | 0.123 | 0.0016346 |
| MouseCellAtlas_Neonatal_all | Neonatal_Skin.Neuron | 14459 | 0.296 | 0.101 | 0.0016822 |
| MouseCellAtlas_all | Muscle.Stromal_cell | 14459 | 0.192 | 0.0659 | 0.0017819 |
| MouseCellAtlas_all | Neonatal_Heart.Epithelial_cell | 14459 | 0.205 | 0.071 | 0.001916 |
| MouseCellAtlas_Muscle | Stromal_cell | 14459 | 0.184 | 0.0636 | 0.0019378 |
| MouseCellAtlas_Adult_all | Muscle.Stromal_cell | 14459 | 0.186 | 0.065 | 0.002078 |
| DropViz_TH_level2 | Endothelial_Tip_Dcn_Nenf.6_2 | 14805 | 0.011 | 0.00385 | 0.002103 |
| MouseCellAtlas_all | Neonatal_Muscle.Epithelial_cell | 14459 | 0.206 | 0.0725 | 0.0022847 |
| MouseCellAtlas_all | Neonatal_Muscle.Tendon_stem.progenitor_cell | 14459 | 0.204 | 0.072 | 0.0023404 |
| TabulaMuris_droplet_all | Heart.fibroblast | 13888 | 0.0956 | 0.0339 | 0.002383 |
| MouseCellAtlas_all | Neonatal_Skin.Neuron | 14459 | 0.246 | 0.0877 | 0.002518 |
| Allen_Mouse_ALM_level1 | Endo | 14744 | 0.0163 | 0.00588 | 0.0027332 |
| TabulaMuris_FACS_all | Aorta.smooth_muscle_cell | 13888 | 0.0415 | 0.0149 | 0.0027547 |
| TabulaMuris_droplet_Mammary | stromal_cell | 13888 | 0.117 | 0.0421 | 0.0027687 |
| MouseCellAtlas_Neonatal_all | Neonatal_Heart.Epithelial_cell | 14459 | 0.206 | 0.0744 | 0.002779 |
| Linnarsson_MouseBrainAtlas_level6_rank4 | Gent | 15140 | 0.0939 | 0.0341 | 0.0029275 |
| MouseCellAtlas_all | Neonatal_Heart.Stromal_cell_Col3a1_high | 14459 | 0.305 | 0.112 | 0.0032767 |
| MouseCellAtlas_Fetal_Brain | Hippocampus_neurons_Asic4_high | 14459 | 0.598 | 0.22 | 0.0033263 |
| DropViz_all_level2 | SN.Polydendrocyte_Tnr_Tmem2.5_2 | 14805 | 0.013 | 0.00479 | 0.0033332 |
| Linnarsson_MouseBrainAtlas_level5 | ENTG2 | 15140 | 0.0837 | 0.0312 | 0.0036429 |
| MouseCellAtlas_all | Pancreas.Stromal_cell_Smoc2_high | 14459 | 0.17 | 0.0636 | 0.0037313 |
| GSE104276_Human_Prefrontal_cortex_per_ages | GW10_GABAergic_neurons | 17199 | 0.0195 | 0.00729 | 0.0038033 |
| Allen_Mouse_ALM_level2 | Endo | 14744 | 0.0149 | 0.00558 | 0.0038429 |
| MouseCellAtlas_all | Neonatal_Muscle.Stromal_cell_Ndn_high | 14459 | 0.228 | 0.0855 | 0.0038486 |
| Allen_Mouse_ALM_level2 | Vip_Chat_1 | 14744 | 0.0321 | 0.0121 | 0.0039229 |
| DropViz_all_level2 | TH.Microglia_C1qb_Tmem119.7_2 | 14805 | 0.0115 | 0.00434 | 0.0039229 |
| MouseCellAtlas_all | Neonatal_Skin.Osteoblast_Ppic_high | 14459 | 0.245 | 0.0922 | 0.0039509 |
| MouseCellAtlas_Adult_all | Pancreas.Stromal_cell_Smoc2_high | 14459 | 0.167 | 0.063 | 0.0040342 |
| MouseCellAtlas_Fetal_Stomache | Stomach_cell_Kazald1_high | 14459 | 0.526 | 0.198 | 0.0040346 |
| MouseCellAtlas_all | Neonatal_Heart.Stromal_cell_Fmod_high | 14459 | 0.263 | 0.0993 | 0.0040836 |
| MouseCellAtlas_all | Neonatal_Skin.Stromal_cell_Gas6_high | 14459 | 0.215 | 0.0816 | 0.0042688 |
| MouseCellAtlas_Neonatal_Muscle | Tendon_stem.progenitor_cell | 14459 | 0.253 | 0.0964 | 0.0043898 |
| Linnarsson_MouseBrainAtlas_level5 | ENTG5 | 15140 | 0.0766 | 0.0292 | 0.004392 |
| MouseCellAtlas_Neonatal_Muscle | Epithelial_cell | 14459 | 0.238 | 0.0918 | 0.0048055 |
| MouseCellAtlas_Pancreas | Stromal_cell_Fn1_high | 14459 | 0.207 | 0.0801 | 0.0048312 |
| MouseCellAtlas_Mesenchymal_Stem_Cell_Cultured | Mesenchymal_stem_cell_Cxcl10_high | 14459 | 0.4 | 0.156 | 0.0051837 |
| DropViz_TH_level1 | Endothelial_tip | 14805 | 0.0142 | 0.00554 | 0.0052378 |
| GSE106678_Mouse_Cortex | Ex18 | 14573 | 0.274 | 0.108 | 0.0053607 |
| MouseCellAtlas_Neonatal_Skin | Stromal_cell_Gas6_high | 14459 | 0.323 | 0.126 | 0.0053915 |
| Linnarsson_MouseBrainAtlas_level5 | ENT8 | 15140 | 0.0612 | 0.0242 | 0.0056528 |
| Linnarsson_MouseBrainAtlas_level5 | COP2 | 15140 | 0.086 | 0.0341 | 0.0058263 |
| TabulaMuris_FACS_all | Aorta.fibroblast | 13888 | 0.0191 | 0.00759 | 0.0058565 |
| MouseCellAtlas_Neonatal_Skin | Osteoblast_Ppic_high | 14459 | 0.364 | 0.144 | 0.0058669 |
| MouseCellAtlas_Adult_all | Mammary_Gland.Stromal_cell | 14459 | 0.194 | 0.0769 | 0.0059344 |
| MouseCellAtlas_all | Mammary_Gland.Stromal_cell | 14459 | 0.193 | 0.077 | 0.0062012 |
| Linnarsson_GSE74672_Mouse_Hypothalamus_Neurons_level2 | Vglut2_16_Gm5595_Tnr | 14550 | 0.0527 | 0.0211 | 0.0062349 |
| MouseCellAtlas_Neonatal_all | Neonatal_Muscle.Endothelial_cell | 14459 | 0.225 | 0.0902 | 0.0063157 |
| MouseCellAtlas_all | Neonatal_Muscle.Endothelial_cell | 14459 | 0.198 | 0.0797 | 0.0064759 |
| MouseCellAtlas_Neonatal_all | Neonatal_Skin.Muscle_cell_Actc1_high | 14459 | 0.241 | 0.0968 | 0.00648 |
| MouseCellAtlas_Neonatal_Heart | Epithelial_cell | 14459 | 0.204 | 0.0827 | 0.006731 |
| DropViz_TH_level2 | Microglia_C1qb_Tmem119.7_2 | 14805 | 0.0113 | 0.00457 | 0.0067635 |
| DropViz_all_level2 | GP.Endothelial_Tip_Dcn_Col15a1.9_2 | 14805 | 0.00932 | 0.00379 | 0.0069768 |
| MouseCellAtlas_Ovary | Ovarian_vascular_surface_endothelium_cell | 14459 | 0.166 | 0.0677 | 0.0070123 |
| MouseCellAtlas_Neonatal_Muscle | Stromal_cell_Ndn_high | 14459 | 0.298 | 0.121 | 0.0070695 |
| MouseCellAtlas_all | Neonatal_Skin.Osteoblast_Dlk1_high | 14459 | 0.198 | 0.0814 | 0.0074352 |
| MouseCellAtlas_Liver | B_cell_Fcmr_high | 14459 | 0.288 | 0.119 | 0.0074914 |
| TabulaMuris_FACS_all | Fat.mesenchymal_stem_cell_of_adipose | 13888 | 0.0181 | 0.00748 | 0.0076513 |
| DropViz_PC_level2 | Microglia_C1qb_Tmem119-Fos.11_3 | 14805 | 0.00611 | 0.00252 | 0.0076737 |
| MouseCellAtlas_Neonatal_Heart | Stromal_cell_Col3a1_high | 14459 | 0.402 | 0.166 | 0.0078389 |
| DropViz_all_level2 | PC.Microglia_C1qb_Tmem119-Fos.11_3 | 14805 | 0.00611 | 0.00253 | 0.0078962 |
| Linnarsson_MouseBrainAtlas_level6_rank4 | Vper | 15140 | 0.0775 | 0.0321 | 0.0079342 |
| MouseCellAtlas_all | Neonatal_Heart.Endothelial_cell_Enpp2_high | 14459 | 0.193 | 0.0802 | 0.0079886 |
| Linnarsson_MouseBrainAtlas_level6_rank2 | Neural_crest_like_glia | 15140 | 0.11 | 0.0458 | 0.0081035 |
| Linnarsson_MouseBrainAtlas_level5 | ENTG3 | 15140 | 0.0665 | 0.0277 | 0.0081127 |
| MouseCellAtlas_Fetal_Lung | Stromal_cell_Ptn_high | 14459 | 0.563 | 0.236 | 0.0084971 |
| MouseCellAtlas_Mammary_Gland | Mammary_Gland_Pregnancy.Stromal_cell | 14459 | 0.0928 | 0.0389 | 0.0086035 |
| DropViz_all_level1 | SN.Endothelial_tip | 14805 | 0.012 | 0.00505 | 0.0089292 |
| MouseCellAtlas_Adult_all | Lung.Endothelial_cell_Kdr_high | 14459 | 0.168 | 0.071 | 0.0090029 |
| MouseCellAtlas_all | Pancreas.Smooth_muscle_cell_Rgs5_high | 14459 | 0.17 | 0.0721 | 0.0090415 |
| MouseCellAtlas_Neonatal_Calvaria | Stromal_cell_Col3a1_high | 14459 | 0.198 | 0.0837 | 0.0090959 |
| TabulaMuris_droplet_all | Heart.endothelial_cell | 13888 | 0.0909 | 0.0385 | 0.0091124 |
| MouseCellAtlas_Liver | Endothelial_cell | 14459 | 0.273 | 0.116 | 0.0092926 |
| DropViz_SN_level2 | Polydendrocyte_Tnr_Tmem2.5_2 | 14805 | 0.0118 | 0.00504 | 0.0094281 |
| MouseCellAtlas_Adult_all | Pancreas.Smooth_muscle_cell_Rgs5_high | 14459 | 0.168 | 0.0716 | 0.0094554 |
| DropViz_PC_level1 | Microglia | 14805 | 0.0115 | 0.00491 | 0.0094555 |
| DropViz_CB_level2 | Endothelial_Stalk_Flt1_Mgp.10_1 | 14805 | 0.00959 | 0.0041 | 0.0097018 |
| TabulaMuris_FACS_Kidney | endothelial_cell | 13888 | 0.0259 | 0.0111 | 0.0097077 |
| MouseCellAtlas_Adult_all | Lung.Endothelial_cells_Vwf_high | 14459 | 0.148 | 0.0632 | 0.0097459 |
| TabulaMuris_FACS_Muscle | mesenchymal_stem_cell | 13888 | 0.0194 | 0.00831 | 0.009807 |
| MouseCellAtlas_Fetal_Stomache | Stromal_cell_Cdkn1c_high | 14459 | 0.493 | 0.212 | 0.0098544 |
| DropViz_all_level1 | TH.Microglia | 14805 | 0.0111 | 0.00479 | 0.0099925 |
| MouseCellAtlas_all | Lung.Endothelial_cells_Vwf_high | 14459 | 0.146 | 0.063 | 0.010396 |
| Linnarsson_MouseBrainAtlas_level5 | ENTG6 | 15140 | 0.0628 | 0.0272 | 0.010504 |
| MouseCellAtlas_all | Lung.Endothelial_cell_Kdr_high | 14459 | 0.162 | 0.0701 | 0.010637 |
| Linnarsson_MouseBrainAtlas_level5 | ENMFB | 15140 | 0.0491 | 0.0214 | 0.010916 |
| DropViz_HC_level2 | Neuron_Slc17a7_Fezf2-Rxfp1.5_15 | 14805 | 0.0191 | 0.00832 | 0.010921 |
| Linnarsson_GSE60361_Mouse_Cortex_Hippocampus_level2 | Peric | 14010 | 0.0809 | 0.0354 | 0.01111 |
| Linnarsson_MouseBrainAtlas_level5 | VECA | 15140 | 0.0698 | 0.0306 | 0.011355 |
| MouseCellAtlas_Adult_all | Bladder.Vascular_smooth_muscle_progenitor_cell | 14459 | 0.111 | 0.0486 | 0.011408 |
| MouseCellAtlas_all | Bladder.Vascular_smooth_muscle_progenitor_cell | 14459 | 0.11 | 0.0486 | 0.011615 |
| DropViz_GP_level2 | Endothelial_Tip_Dcn_Col15a1.9_2 | 14805 | 0.00881 | 0.00389 | 0.01181 |
| TabulaMuris_FACS_Fat | mesenchymal_stem_cell_of_adipose | 13888 | 0.0163 | 0.00719 | 0.011825 |
| MouseCellAtlas_all | Neonatal_Heart.Vascular_endothelial_cell | 14459 | 0.178 | 0.0785 | 0.011871 |
| Linnarsson_MouseBrainAtlas_level5 | ENTG4 | 15140 | 0.0696 | 0.0308 | 0.011886 |
| DropViz_all_level2 | TH.Endothelial_Tip_Dcn.6_1 | 14805 | 0.00918 | 0.00406 | 0.011905 |
| DropViz_STR_level2 | Endothelial_Stalk_Flt1_Gkn3.7_2 | 14805 | 0.0103 | 0.00457 | 0.011934 |
| Linnarsson_MouseBrainAtlas_level6_rank3 | Enteric_neurons | 15140 | 0.0726 | 0.0322 | 0.012009 |
| DropViz_all_level2 | SN.Endothelial_Stalk_Flt1_Vwf.12_4 | 14805 | 0.00983 | 0.00437 | 0.012141 |
| MouseCellAtlas_all | Bladder.Mesenchymal_stromal_cell | 14459 | 0.135 | 0.0601 | 0.012192 |
| DropViz_all_level1 | SN.Endothelial_stalk | 14805 | 0.0137 | 0.00611 | 0.012226 |
| MouseCellAtlas_Mammary_Gland | Mammary_Gland_Involution.Muscle_cell_Pi16_high | 14459 | 0.133 | 0.0592 | 0.012364 |
| Linnarsson_GSE104323_Mouse_Dentate_gyrus | Neuroblast | 14909 | 0.137 | 0.0612 | 0.012411 |
| MouseCellAtlas_all | Neonatal_Muscle.Neuron | 14459 | 0.196 | 0.0873 | 0.012492 |
| DropViz_all_level2 | SN.Endothelial_Tip_Dcn_Mgp.14_1 | 14805 | 0.0085 | 0.0038 | 0.012541 |
| TabulaMuris_droplet_all | Mammary.stromal_cell | 13888 | 0.0832 | 0.0372 | 0.012629 |
| MouseCellAtlas_Neonatal_all | Neonatal_Heart.Vascular_endothelial_cell | 14459 | 0.196 | 0.0875 | 0.012689 |
| MouseCellAtlas_Neonatal_Skin | Neuron | 14459 | 0.234 | 0.105 | 0.012695 |
| MouseCellAtlas_Adult_all | Small_Intestine.Stromal_cell_Adamdec1_high | 14459 | 0.102 | 0.0457 | 0.012696 |
| DropViz_all_level2 | ENT.Endothelial_Stalk_Flt1_Gkn3.2_4 | 14805 | 0.00916 | 0.0041 | 0.012723 |
| MouseCellAtlas_Adult_all | Mammary_Gland.Dendritic_cell_Siglech_high | 14459 | 0.174 | 0.0782 | 0.012937 |
| MouseCellAtlas_all | Bladder.Stromal_cell_Dpt_high | 14459 | 0.108 | 0.0486 | 0.013019 |
| MouseCellAtlas_Neonatal_Skin | Osteoblast_Dlk1_high | 14459 | 0.261 | 0.118 | 0.01311 |
| Linnarsson_MouseBrainAtlas_level5 | ENT3 | 15140 | 0.0599 | 0.027 | 0.013164 |
| TabulaMuris_droplet_all | Muscle.mesenchymal_stem_cell | 13888 | 0.0743 | 0.0335 | 0.013336 |
| MouseCellAtlas_all | Small_Intestine.Stromal_cell_Adamdec1_high | 14459 | 0.101 | 0.0456 | 0.013543 |
| MouseCellAtlas_Adult_all | Bladder.Mesenchymal_stromal_cell | 14459 | 0.13 | 0.059 | 0.013572 |
| TabulaMuris_droplet_Trachea | endothelial_cell | 13888 | 0.146 | 0.0661 | 0.013637 |
| MouseCellAtlas_Adult_all | Bladder.Stromal_cell_Dpt_high | 14459 | 0.106 | 0.0481 | 0.013764 |
| TabulaMuris_droplet_all | Kidney.fenestrated_cell | 13888 | 0.0942 | 0.0429 | 0.014047 |
| MouseCellAtlas_Neonatal_all | Neonatal_Muscle.Neuron | 14459 | 0.216 | 0.0984 | 0.014103 |
| GSE106678_Mouse_Cortex | Ex2 | 14573 | 0.221 | 0.101 | 0.01414 |
| Linnarsson_MouseBrainAtlas_level6_rank1 | Vascular_cells | 15140 | 0.103 | 0.0471 | 0.014253 |
| MouseCellAtlas_Neonatal_Heart | Stromal_cell_Fmod_high | 14459 | 0.297 | 0.136 | 0.014277 |
| Linnarsson_GSE95315_Mouse_Dentate_gyrus | Endothelial | 11815 | 0.0849 | 0.0388 | 0.014302 |
| MouseCellAtlas_Lung | Endothelial_cell_Kdr_high | 14459 | 0.165 | 0.0752 | 0.014341 |
| MouseCellAtlas_Neonatal_all | Neonatal_Heart.Endothelial_cell_Enpp2_high | 14459 | 0.184 | 0.084 | 0.01442 |
| DropViz_all_level1 | PC.Microglia | 14805 | 0.0101 | 0.00461 | 0.01447 |
| MouseCellAtlas_Neonatal_all | Neonatal_Skin.Stromal_cell_Akr1c18_high | 14459 | 0.161 | 0.0739 | 0.014489 |
| DropViz_all_level2 | SN.Endothelial_Stalk_Flt1_Cdkn1c.12_1 | 14805 | 0.00987 | 0.00453 | 0.014758 |
| DropViz_all_level2 | TH.Endothelial_Stalk_Flt1_Gkn3.4_1 | 14805 | 0.01 | 0.00462 | 0.014866 |
| DropViz_all_level2 | GP.Endothelial_Stalk_Flt1_Mgp.7_3 | 14805 | 0.00903 | 0.00416 | 0.01505 |
| MouseCellAtlas_all | Ovary.Ovarian_vascular_surface_endothelium_cell | 14459 | 0.136 | 0.0627 | 0.015067 |
| TabulaMuris_droplet_Kidney | fenestrated_cell | 13888 | 0.108 | 0.05 | 0.015101 |
| TabulaMuris_droplet_Muscle | mesenchymal_stem_cell | 13888 | 0.103 | 0.0477 | 0.015212 |
| TabulaMuris_droplet_Heart | fibroblast | 13888 | 0.0908 | 0.0419 | 0.015253 |
| MouseCellAtlas_all | Neonatal_Skin.Muscle_cell_Actc1_high | 14459 | 0.165 | 0.0766 | 0.01548 |
| MouseCellAtlas_Embryo_all | Embryonic_Mesenchyme.Stromal_Cell_fabp7_high | 14459 | 0.276 | 0.128 | 0.015673 |
| DropViz_all_level2 | GP.Endothelial_Stalk_Flt1_Cdkn1c.7_2 | 14805 | 0.00986 | 0.0046 | 0.016014 |
| Linnarsson_MouseBrainAtlas_level6_rank3 | Neural_crestlike_glia | 15140 | 0.0772 | 0.036 | 0.016117 |
| MouseCellAtlas_Uterus | Stromal_cell_Has1_high | 14459 | 0.155 | 0.0723 | 0.016215 |
| Linnarsson_GSE104323_Mouse_Dentate_gyrus | Cajal_Retzius | 14909 | 0.122 | 0.0568 | 0.016238 |
| MouseCellAtlas_Neonatal_all | Neonatal_Rib.Stromal_cell_Tnmd_high | 14459 | 0.214 | 0.1 | 0.016326 |
| Linnarsson_MouseBrainAtlas_level5 | PER2 | 15140 | 0.0488 | 0.0229 | 0.016529 |
| MouseCellAtlas_all | Mammary_Gland.Dendritic_cell_Siglech_high | 14459 | 0.163 | 0.0764 | 0.016554 |
| MouseCellAtlas_all | Uterus.Muscle_cell_Mgp_high | 14459 | 0.172 | 0.0806 | 0.016603 |
| TabulaMuris_FACS_Brain_Neurons | smooth_muscle_cell | 13888 | 0.0126 | 0.00594 | 0.016626 |
| MouseCellAtlas_Mammary_Gland | Mammary_Gland_Virgin.Stromal_cell_Pi16_high | 14459 | 0.108 | 0.0507 | 0.016659 |
| MouseCellAtlas_Adult_all | Pancreas.Endothelial_cell_Fabp4_high | 14459 | 0.167 | 0.0787 | 0.016777 |
| Linnarsson_MouseBrainAtlas_level5 | ENT9 | 15140 | 0.0443 | 0.0208 | 0.016796 |
| GSE87544_Mouse_Hypothalamus | Epith1 | 13878 | 0.0675 | 0.0318 | 0.016859 |
| DropViz_all_level2 | SN.Endothelial_Stalk_Flt1_Gkn3.12_2 | 14805 | 0.00771 | 0.00364 | 0.017005 |
| Linnarsson_MouseBrainAtlas_level5 | PSNP1 | 15140 | 0.04 | 0.0189 | 0.01713 |
| Linnarsson_MouseBrainAtlas_level6_rank4 | Vend | 15140 | 0.07 | 0.0331 | 0.017162 |
| Linnarsson_MouseBrainAtlas_level6_rank4 | Enne | 15140 | 0.063 | 0.0298 | 0.017212 |
| DropViz_ENT_level2 | Endothelial_Stalk_Flt1_Gkn3.2_4 | 14805 | 0.00996 | 0.00472 | 0.017369 |
| Allen_Mouse_ALM_level1 | Glia | 14744 | 0.0177 | 0.0084 | 0.017482 |
| TabulaMuris_FACS_all | Mammary.stromal_cell | 13888 | 0.0156 | 0.00741 | 0.017589 |
| MouseCellAtlas_all | Uterus.Stromal_cell_Has1_high | 14459 | 0.0984 | 0.0469 | 0.017882 |
| TabulaMuris_FACS_all | Muscle.mesenchymal_stem_cell | 13888 | 0.0153 | 0.0073 | 0.017981 |
| GSE98816_Mouse_Brain_Vascular | FB2 | 14130 | 0.0163 | 0.00779 | 0.018053 |
| MouseCellAtlas_Adult_all | Lung.Endothelial_cell_Tmem100_high | 14459 | 0.177 | 0.0845 | 0.018126 |
| MouseCellAtlas_Embryo_all | Fetal_Stomache.Stomach_cell_Kazald1_high | 14459 | 0.356 | 0.17 | 0.018157 |
| GSE87544_Mouse_Hypothalamus | Glu11 | 13878 | 0.144 | 0.0686 | 0.018164 |
| TabulaMuris_FACS_Heart | fibroblast | 13888 | 0.0223 | 0.0107 | 0.018274 |
| Allen_Mouse_VISp_level2 | L6a_Syt17 | 13868 | 0.0253 | 0.0121 | 0.018446 |
| MouseCellAtlas_all | Pancreas.Endothelial_cell_Fabp4_high | 14459 | 0.163 | 0.0781 | 0.018497 |
| MouseCellAtlas_Adult_all | Uterus.Muscle_cell_Mgp_high | 14459 | 0.164 | 0.0789 | 0.018928 |
| MouseCellAtlas_Neonatal_all | Neonatal_Skin.Muscle_cell_Lrrc15_high | 14459 | 0.227 | 0.11 | 0.018993 |
| Linnarsson_MouseBrainAtlas_level5 | PER1 | 15140 | 0.0604 | 0.0292 | 0.019338 |
| DropViz_CB_level2 | Endothelial_Tip_Dcn_Apod.11_2 | 14805 | 0.00697 | 0.00338 | 0.019575 |
| MouseCellAtlas_all | Lung.Endothelial_cell_Tmem100_high | 14459 | 0.173 | 0.084 | 0.019834 |
| DropViz_TH_level2 | Endothelial_Tip_Dcn.6_1 | 14805 | 0.00867 | 0.00422 | 0.019889 |
| Linnarsson_GSE74672_Mouse_Hypothalamus_level1 | endothelial | 14550 | 0.114 | 0.0553 | 0.019952 |
| MouseCellAtlas_all | Uterus.Granulocyte | 14459 | 0.169 | 0.0823 | 0.019959 |
| MouseCellAtlas_Adult_all | Uterus.Granulocyte | 14459 | 0.168 | 0.0817 | 0.020072 |
| MouseCellAtlas_Embryonic_Mesenchyme | Neuron_Npy_high | 14459 | 0.225 | 0.11 | 0.020656 |
| MouseCellAtlas_Small_Intestine | Stromal_cell_Adamdec1_high | 14459 | 0.0927 | 0.0454 | 0.020676 |
| MouseCellAtlas_Adult_all | Uterus.Stromal_cell_Has1_high | 14459 | 0.0933 | 0.0457 | 0.020729 |
| MouseCellAtlas_all | Mammary_Gland.Stromal_cell_Col3a1_high | 14459 | 0.141 | 0.0694 | 0.020744 |
| Linnarsson_GSE76381_Mouse_Midbrain | NbL2 | 14550 | 0.0772 | 0.038 | 0.02102 |
| Linnarsson_GSE76381_Mouse_Midbrain | Peric | 14550 | 0.0756 | 0.0373 | 0.021302 |
| TabulaMuris_FACS_all | Lung.mesothelial_cell | 13888 | 0.00796 | 0.00393 | 0.021354 |
| DropViz_all_level2 | CB.Endothelial_Stalk_Flt1_Mgp.10_1 | 14805 | 0.00713 | 0.00353 | 0.021705 |
| GSE104276_Human_Prefrontal_cortex_per_ages | GW16_OPC | 17199 | 0.018 | 0.00891 | 0.02198 |
| Allen_Mouse_ALM_level2 | Micro_1 | 14744 | 0.0062 | 0.00308 | 0.022014 |
| MouseCellAtlas_Neonatal_all | Neonatal_Muscle.Muscle_cell_Actc1_high | 14459 | 0.164 | 0.0813 | 0.022143 |
| MouseCellAtlas_all | Neonatal_Muscle.Muscle_cell_Actc1_high | 14459 | 0.142 | 0.0705 | 0.022155 |
| MouseCellAtlas_Adult_all | Mammary_Gland.Stromal_cell_Col3a1_high | 14459 | 0.137 | 0.0684 | 0.022164 |
| MouseCellAtlas_Embryonic_Mesenchyme | Stromal_Cell_fabp7_high | 14459 | 0.281 | 0.14 | 0.022772 |
| Linnarsson_GSE74672_Mouse_Hypothalamus_Neurons_level2 | circadian_1_VipGrp | 14550 | 0.0795 | 0.0398 | 0.022797 |
| MouseCellAtlas_Bone_Marrow | Bone_Marrow_c_kit.Megakaryocyte_progenitor_cell | 14459 | 0.169 | 0.0845 | 0.022838 |
| DropViz_TH_level1 | Microglia | 14805 | 0.0104 | 0.00522 | 0.023075 |
| MouseCellAtlas_Adult_all | Small_Intestine.Dendritic_cell_Siglech_high | 14459 | 0.137 | 0.0689 | 0.023101 |
| TabulaMuris_FACS_Lung | mesothelial_cell | 13888 | 0.00813 | 0.00408 | 0.023139 |
| MouseCellAtlas_Adult_all | Ovary.Ovarian_vascular_surface_endothelium_cell | 14459 | 0.116 | 0.0583 | 0.023165 |
| TabulaMuris_droplet_all | Bladder.mesenchymal_cell | 13888 | 0.0568 | 0.0285 | 0.02325 |
| DropViz_HC_level2 | Endothelial_Tip_Dcn_Il33.17_4 | 14805 | 0.00685 | 0.00344 | 0.023272 |
| Linnarsson_MouseBrainAtlas_level5 | PSPEP7 | 15140 | 0.0729 | 0.0367 | 0.023527 |
| Linnarsson_GSE95315_Mouse_Dentate_gyrus | Cajal_Retzius | 11815 | 0.156 | 0.0786 | 0.023528 |
| MouseCellAtlas_all | Neonatal_Skin.Stromal_cell_Akr1c18_high | 14459 | 0.126 | 0.0632 | 0.023538 |
| DropViz_all_level1 | GP.Endothelial_tip | 14805 | 0.0113 | 0.00569 | 0.02377 |
| DropViz_SN_level2 | Endothelial_Tip_Dcn_Mgp.14_1 | 14805 | 0.00786 | 0.00397 | 0.023798 |
| GSE106678_Mouse_Cortex | Ex26 | 14573 | 0.335 | 0.17 | 0.024229 |
| DropViz_HC_level2 | Neuron_Slc17a7_Fezf2-Efna5.5_14 | 14805 | 0.0184 | 0.00934 | 0.024253 |
| MouseCellAtlas_Bladder | Vascular_smooth_muscle_progenitor_cell | 14459 | 0.105 | 0.0534 | 0.024308 |
| Linnarsson_MouseBrainAtlas_level5 | ENT5 | 15140 | 0.0609 | 0.0309 | 0.024444 |
| MouseCellAtlas_Fetal_Lung | Erythroblast_Hba.x_high | 14459 | 0.199 | 0.101 | 0.024487 |
| Allen_Mouse_VISp_level2 | L2_Ngb | 13868 | 0.0243 | 0.0124 | 0.024957 |
| DropViz_all_level1 | ENT.Endothelial_stalk | 14805 | 0.0111 | 0.00568 | 0.025535 |
| MouseCellAtlas_Lung | Endothelial_cells_Vwf_high | 14459 | 0.131 | 0.067 | 0.025596 |
| MouseCellAtlas_Embryo_all | Embryonic_Mesenchyme.Neuron_Npy_high | 14459 | 0.191 | 0.0978 | 0.025643 |
| DropViz_GP_level2 | Endothelial_Stalk_Flt1_Mgp.7_3 | 14805 | 0.00835 | 0.00429 | 0.025916 |
| TabulaMuris_droplet_all | Trachea.endothelial_cell | 13888 | 0.12 | 0.0619 | 0.025948 |
| DropViz_all_level2 | TH.Endothelial_Stalk_Flt1_Vwf.4_4 | 14805 | 0.00833 | 0.00429 | 0.026102 |
| DropViz_all_level2 | GP.Polydendrocyte_Tnr_Pdgfa-Pik3r3.4_1 | 14805 | 0.0102 | 0.00524 | 0.026209 |
| TabulaMuris_FACS_all | Heart.fibroblast | 13888 | 0.0163 | 0.00838 | 0.026213 |
| DropViz_all_level2 | ENT.Endothelial_Stalk_Flt1_Tgfb2.2_3 | 14805 | 0.00804 | 0.00415 | 0.026404 |
| Allen_Mouse_ALM_level2 | Vip_Lect1 | 14744 | 0.0185 | 0.00955 | 0.026482 |
| MouseCellAtlas_Pancreas | Stromal_cell_Mfap4_high | 14459 | 0.138 | 0.0714 | 0.026524 |
| MouseCellAtlas_Kidney | Stromal_cell_Mgp_high | 14459 | 0.0908 | 0.047 | 0.026571 |
| DropViz_TH_level2 | Endothelial_Stalk_Flt1_Gkn3.4_1 | 14805 | 0.00928 | 0.00481 | 0.026914 |
| MouseCellAtlas_Adult_all | Mammary_Gland.Macrophage | 14459 | 0.0997 | 0.0517 | 0.026951 |
| Linnarsson_GSE60361_Mouse_Cortex_Hippocampus_level2 | Vend1 | 14010 | 0.0499 | 0.0259 | 0.027228 |
| DropViz_SN_level2 | Endothelial_Stalk_Flt1_Vwf.12_4 | 14805 | 0.00874 | 0.00455 | 0.027375 |
| MouseCellAtlas_Liver | Dendritic_cell_Siglech_high | 14459 | 0.195 | 0.101 | 0.02752 |
| DropViz_all_level2 | HC.Endothelial_Tip_Dcn_Il33.17_4 | 14805 | 0.00688 | 0.00358 | 0.027528 |
| DropViz_STR_level2 | Neurogenesis_Sox4_Stmn2.2_2 | 14805 | 0.00837 | 0.00437 | 0.027628 |
| TabulaMuris_FACS_all | Lung.stromal_cell | 13888 | 0.0157 | 0.00819 | 0.027943 |
| MouseCellAtlas_Fetal_Stomache | Erythroblast_Hbb.bs_high | 14459 | 0.485 | 0.254 | 0.028212 |
| MouseCellAtlas_Neonatal_all | Neonatal_Rib.Stromal_cell_Acta1_high | 14459 | 0.194 | 0.102 | 0.028374 |
| DropViz_GP_level2 | Endothelial_Stalk_Flt1_Cdkn1c.7_2 | 14805 | 0.00909 | 0.00478 | 0.028633 |
| MouseCellAtlas_Neonatal_Rib | Endothelial_cell | 14459 | 0.148 | 0.078 | 0.02868 |
| MouseCellAtlas_Fetal_Stomache | Stromal_cell_Dcn_high | 14459 | 0.403 | 0.213 | 0.029086 |
| MouseCellAtlas_all | Pancreas.Erythroblast_Igkc_high | 14459 | 0.15 | 0.0791 | 0.029111 |
| MouseCellAtlas_Testis | Spermatids_Hmgb4_high | 14459 | 0.094 | 0.0497 | 0.029221 |
| MouseCellAtlas_Adult_all | Kidney.Stromal_cell_Mgp_high | 14459 | 0.0888 | 0.047 | 0.02934 |
| MouseCellAtlas_all | Uterus.Stromal_cell_Ccl11_high | 14459 | 0.0926 | 0.049 | 0.029391 |
| DropViz_all_level1 | TH.Endothelial_stalk | 14805 | 0.0108 | 0.00574 | 0.029491 |
| MouseCellAtlas_all | Uterus.Endothelial_cell_Tm4sf1_high | 14459 | 0.167 | 0.0884 | 0.02955 |
| Linnarsson_MouseBrainAtlas_level5 | NFOL2 | 15140 | 0.0478 | 0.0254 | 0.029703 |
| MouseCellAtlas_all | Kidney.Stromal_cell_Mgp_high | 14459 | 0.0886 | 0.047 | 0.02978 |
| MouseCellAtlas_Fetal_Intestine | Stromal_cell_Stmn2_high_ | 14459 | 0.318 | 0.169 | 0.029954 |
| Linnarsson_GSE101601_Mouse_Somatosensory_cortex | Pericytes | 14550 | 0.101 | 0.0539 | 0.029974 |
| MouseCellAtlas_all | Small_Intestine.Dendritic_cell_Siglech_high | 14459 | 0.125 | 0.0666 | 0.030309 |
| MouseCellAtlas_Adult_all | Uterus.Endothelial_cell_Tm4sf1_high | 14459 | 0.164 | 0.0873 | 0.030506 |
| MouseCellAtlas_Adult_all | Pancreas.Erythroblast_Igkc_high | 14459 | 0.147 | 0.0784 | 0.030565 |
| DropViz_all_level2 | GP.Endothelial_Stalk_Flt1_Car4.7_5 | 14805 | 0.0086 | 0.00459 | 0.030663 |
| MouseCellAtlas_Uterus | Stromal_cell_Ccl11_high | 14459 | 0.147 | 0.0787 | 0.030669 |
| Linnarsson_MouseBrainAtlas_level5 | ENTG7 | 15140 | 0.0575 | 0.0308 | 0.030968 |
| DropViz_all_level2 | GP.Endothelial_Tip_Dcn_Mgp.9_1 | 14805 | 0.00733 | 0.00393 | 0.031128 |
| TabulaMuris_FACS_Diaphragm | mesenchymal_stem_cell | 13888 | 0.0184 | 0.00984 | 0.031151 |
| DropViz_all_level2 | ENT.Mural_Rgs5Acta2_Junb.3_6 | 14805 | 0.00482 | 0.00259 | 0.031245 |
| MouseCellAtlas_Trophoblast_Stem_Cell | MEF | 14459 | 0.192 | 0.103 | 0.031253 |
| Linnarsson_MouseBrainAtlas_level5 | VECV | 15140 | 0.055 | 0.0296 | 0.031511 |
| Allen_Mouse_VISp_level2 | Oligo_96.Rik | 13868 | 0.0117 | 0.00632 | 0.032141 |
| MouseCellAtlas_Adult_all | Bladder.Epithelial_cell_Gm23935_high | 14459 | 0.115 | 0.0624 | 0.03215 |
| MouseCellAtlas_Adult_all | Uterus.Stromal_cell_Ccl11_high | 14459 | 0.0889 | 0.0481 | 0.032212 |
| MouseCellAtlas_all | Lung.Stromal_cell_Dcn_high | 14459 | 0.11 | 0.0595 | 0.03264 |
| MouseCellAtlas_all | Bladder.Epithelial_cell_Gm23935_high | 14459 | 0.115 | 0.0625 | 0.032648 |
| DropViz_all_level2 | PC.Endothelial_Stalk_Flt1_Vwf.12_2 | 14805 | 0.00865 | 0.00469 | 0.032649 |
| Linnarsson_GSE104323_Mouse_Dentate_gyrus | Endothelial | 14909 | 0.086 | 0.0467 | 0.032725 |
| Linnarsson_GSE75330_Mouse_Oligodendrocytes | NFOL1 | 14541 | 0.0887 | 0.0482 | 0.032789 |
| MouseCellAtlas_all | Neonatal_Skin.Muscle_cell_Lrrc15_high | 14459 | 0.155 | 0.0842 | 0.032822 |
| DropViz_all_level2 | ENT.Polydendrocyte_Tnr_Ctps.5_3 | 14805 | 0.00807 | 0.00439 | 0.032926 |
| MouseCellAtlas_Bone_Marrow | Bone_Marrow_Mesenchyme.Endothelial_cells_Ly6c1_high | 14459 | 0.175 | 0.095 | 0.032927 |
| DropViz_all_level2 | CB.Endothelial_Tip_Dcn_Apod.11_2 | 14805 | 0.00556 | 0.00303 | 0.032992 |
| DropViz_SN_level2 | Endothelial_Stalk_Flt1_Gkn3.12_2 | 14805 | 0.00691 | 0.00377 | 0.03316 |
| Linnarsson_GSE103840_Mouse_Dorsal_horn | Glut_Cck_Trh | 14550 | 0.103 | 0.0563 | 0.033256 |
| GSE106678_Mouse_Cortex | Ex4 | 14573 | 0.208 | 0.113 | 0.03329 |
| DropViz_SN_level2 | Endothelial_Stalk_Flt1_Cdkn1c.12_1 | 14805 | 0.00871 | 0.00475 | 0.033323 |
| DropViz_HC_level2 | Endothelial_Tip_Dcn_Mgp.17_3 | 14805 | 0.0067 | 0.00366 | 0.033406 |
| Linnarsson_MouseBrainAtlas_level5 | PSNP5 | 15140 | 0.0321 | 0.0175 | 0.03363 |
| MouseCellAtlas_Small_Intestine | Dendritic_cell_Siglech_high | 14459 | 0.127 | 0.0696 | 0.033727 |
| MouseCellAtlas_Adult_all | Lung.Stromal_cell_Dcn_high | 14459 | 0.108 | 0.0589 | 0.033771 |
| Linnarsson_GSE60361_Mouse_Cortex_Hippocampus_level1 | endothelial_mural | 14010 | 0.0759 | 0.0415 | 0.033799 |
| MouseCellAtlas_Uterus | Granulocyte | 14459 | 0.218 | 0.119 | 0.033878 |
| MouseCellAtlas_Neonatal_all | Neonatal_Muscle.Mesenchymal_cell | 14459 | 0.222 | 0.122 | 0.034274 |
| MouseCellAtlas_Adult_all | Liver.B_cell_Fcmr_high | 14459 | 0.146 | 0.0803 | 0.034304 |
| MouseCellAtlas_all | Placenta.B_cell | 14459 | 0.0683 | 0.0375 | 0.034413 |
| DropViz_all_level2 | SN.Endothelial_Tip_Dcn_Ifitm1.14_2 | 14805 | 0.00667 | 0.00367 | 0.034668 |
| MouseCellAtlas_Bladder | Stromal_cell_Dpt_high | 14459 | 0.101 | 0.0559 | 0.034719 |
| MouseCellAtlas_Adult_all | Lung.B_Cell | 14459 | 0.156 | 0.0862 | 0.034778 |
| TabulaMuris_FACS_all | Trachea.stromal_cell | 13888 | 0.0144 | 0.00795 | 0.035166 |
| DropViz_all_level2 | TH.Endothelial_Stalk_Flt1_Car4.4_3 | 14805 | 0.00846 | 0.00468 | 0.035299 |
| MouseCellAtlas_Uterus | Muscle_cell_Mgp_high | 14459 | 0.185 | 0.102 | 0.035355 |
| MouseCellAtlas_Lung | Endothelial_cell_Tmem100_high | 14459 | 0.171 | 0.0945 | 0.035562 |
| MouseCellAtlas_Placenta | B_cell | 14459 | 0.0691 | 0.0383 | 0.035651 |
| TabulaMuris_droplet_Marrow | macrophage | 13888 | 0.0831 | 0.0461 | 0.0358 |
| MouseCellAtlas_all | Lung.B_Cell | 14459 | 0.156 | 0.0865 | 0.035946 |
| DropViz_all_level2 | SN.Endothelial_Stalk_Flt1_Car4.12_3 | 14805 | 0.00824 | 0.00458 | 0.036047 |
| DroNc_Human_Hippocampus | END | 16914 | 0.151 | 0.0841 | 0.036345 |
| GSE106678_Mouse_Cortex | Oligo2 | 14573 | 0.107 | 0.0598 | 0.036596 |
| MouseCellAtlas_Neonatal_all | Neonatal_Calvaria.Stromal_cell_Col1a1_high | 14459 | 0.188 | 0.105 | 0.036963 |
| Linnarsson_GSE95752_Mouse_Dentate_gyrus | Endothelial | 12517 | 0.106 | 0.0594 | 0.037331 |
| MouseCellAtlas_all | Bladder.Stromal_cell_Car3_high | 14459 | 0.0849 | 0.0476 | 0.037352 |
| DropViz_HC_level2 | Neuron_Slc17a7_Calb2-Vgll3.6_5 | 14805 | 0.0135 | 0.00762 | 0.038047 |
| DropViz_ENT_level2 | Mural_Rgs5Acta2_Junb.3_6 | 14805 | 0.00482 | 0.00272 | 0.038273 |
| DropViz_PC_level2 | Endothelial_Stalk_Flt1_Vwf.12_2 | 14805 | 0.00775 | 0.00438 | 0.038428 |
| MouseCellAtlas_Adult_all | Mammary_Gland.Endothelial_cell | 14459 | 0.113 | 0.0643 | 0.038803 |
| Linnarsson_GSE95315_Mouse_Dentate_gyrus | Neuroblast | 11815 | 0.116 | 0.0657 | 0.038825 |
| DropViz_all_level2 | SN.Neuron_Th_C1ql3.4_4 | 14805 | 0.00939 | 0.00532 | 0.038853 |
| Allen_Mouse_LGd_level2 | OPC_Pdgfra | 14545 | 0.0134 | 0.0076 | 0.038955 |
| MouseCellAtlas_all | Lung.Stromal_cell_Inmt_high | 14459 | 0.114 | 0.0646 | 0.039077 |
| DropViz_all_level2 | ENT.Oligodendrocyte_Trf_Ctps.1_5 | 14805 | 0.00847 | 0.00481 | 0.039092 |
| MouseCellAtlas_Testis | Spermatogonia_Tbc1d23_high | 14459 | 0.0835 | 0.0474 | 0.039124 |
| DropViz_ENT_level2 | Endothelial_Stalk_Flt1_Tgfb2.2_3 | 14805 | 0.00843 | 0.00479 | 0.039164 |
| MouseCellAtlas_Adult_all | Lung.Stromal_cell_Inmt_high | 14459 | 0.113 | 0.0643 | 0.039181 |
| MouseCellAtlas_Adult_all | Mammary_Gland.B_cell_Ly6d_high | 14459 | 0.158 | 0.0897 | 0.039192 |
| MouseCellAtlas_Adult_all | Pancreas.Endothelial_cell_Tm4sf1_high | 14459 | 0.137 | 0.0782 | 0.039454 |
| MouseCellAtlas_Stomach | Stomach_cell_Mt2_high | 14459 | 0.24 | 0.136 | 0.039458 |
| DropViz_all_level2 | HC.Endothelial_Tip_Dcn_Mgp.17_3 | 14805 | 0.00673 | 0.00383 | 0.03965 |
| DropViz_FC_level1 | Endothelial_stalk | 14805 | 0.0112 | 0.00639 | 0.039704 |
| MouseCellAtlas_Embryo_all | Fetal_Intestine.Neuronal_Cells_Rtn1_high | 14459 | 0.2 | 0.114 | 0.039845 |
| MouseCellAtlas_Adult_all | Bladder.Stromal_cell_Car3_high | 14459 | 0.0821 | 0.0469 | 0.040067 |
| MouseCellAtlas_Bladder | Mesenchymal_stromal_cell | 14459 | 0.124 | 0.071 | 0.040254 |
| MouseCellAtlas_all | Mammary_Gland.Macrophage | 14459 | 0.0851 | 0.0487 | 0.040397 |
| MouseCellAtlas_Pancreas | Stromal_cell_Smoc2_high | 14459 | 0.146 | 0.0835 | 0.040517 |
| Linnarsson_GSE60361_Mouse_Cortex_Hippocampus_level2 | Vend2 | 14010 | 0.059 | 0.0339 | 0.040878 |
| GSE92332_Mouse_Epithelium_droplet | Endocrine | 11993 | 0.0965 | 0.0554 | 0.040879 |
| Linnarsson_MouseBrainAtlas_level5 | PSPEP5 | 15140 | 0.0492 | 0.0283 | 0.041058 |
| DropViz_all_level2 | FC.Endothelial_Stalk_Flt1_Mgp.12_2 | 14805 | 0.00705 | 0.00407 | 0.041593 |
| DropViz_all_level2 | ENT.Endothelial_Stalk_Flt1_Vwf.2_2 | 14805 | 0.00642 | 0.00371 | 0.041714 |
| MouseCellAtlas_Embryo_all | Fetal_Stomache.Stromal_cell_Cdkn1c_high | 14459 | 0.302 | 0.174 | 0.041893 |
| MouseCellAtlas_Peripheral_Blood | B_cell_Rps27rt_high | 14459 | 0.159 | 0.0922 | 0.042092 |
| MouseCellAtlas_Embryonic_Mesenchyme | Neuronal_Progenitors | 14459 | 0.157 | 0.091 | 0.04217 |
| MouseCellAtlas_all | Mammary_Gland.Endothelial_cell | 14459 | 0.11 | 0.0637 | 0.042454 |
| TabulaMuris_FACS_all | Kidney.endothelial_cell | 13888 | 0.0158 | 0.00919 | 0.042487 |
| Allen_Mouse_ALM_level1 | SMC | 14744 | 0.00897 | 0.00521 | 0.042497 |
| MouseCellAtlas_all | Pancreas.Endothelial_cell_Tm4sf1_high | 14459 | 0.134 | 0.0777 | 0.042513 |
| MouseCellAtlas_Embryo_all | Placenta.B_cell | 14459 | 0.0634 | 0.0369 | 0.042796 |
| TabulaMuris_droplet_Heart | endothelial_cell | 13888 | 0.0811 | 0.0472 | 0.042935 |
| DropViz_all_level2 | SN.Astrocyte_Gja1_Igfbp2.7_3 | 14805 | 0.00498 | 0.00291 | 0.043278 |
| MouseCellAtlas_Stomach | Pit_cell_Gm26917_high | 14459 | 0.143 | 0.0837 | 0.043686 |
| DropViz_TH_level2 | Endothelial_Stalk_Flt1_Vwf.4_4 | 14805 | 0.00761 | 0.00446 | 0.043863 |
| MouseCellAtlas_Neonatal_Heart | Vascular_endothelial_cell | 14459 | 0.166 | 0.0974 | 0.044299 |
| MouseCellAtlas_all | Neonatal_Muscle.Macrophage_Pf4_high | 14459 | 0.131 | 0.0773 | 0.044553 |
| DropViz_FC_level2 | Endothelial_Stalk_Flt1_Mgp.12_2 | 14805 | 0.00668 | 0.00393 | 0.044647 |
| DropViz_HC_level1 | Endothelial_stalk | 14805 | 0.00975 | 0.00574 | 0.044733 |
| MouseCellAtlas_all | Liver.B_cell_Fcmr_high | 14459 | 0.132 | 0.0775 | 0.044762 |
| Allen_Mouse_ALM_level2 | Micro_2 | 14744 | 0.00894 | 0.00527 | 0.044838 |
| GSE104276_Human_Prefrontal_cortex_per_ages | GW26_GABAergic_neurons | 17199 | 0.019 | 0.0112 | 0.04484 |
| Allen_Mouse_ALM_level2 | Vip_Sfrp2_1 | 14744 | 0.0211 | 0.0125 | 0.044847 |
| MouseCellAtlas_Fetal_Intestine | Neuronal_Cells_Rtn1_high | 14459 | 0.205 | 0.121 | 0.045172 |
| MouseCellAtlas_all | Neonatal_Heart.Dividing_cell | 14459 | 0.168 | 0.0995 | 0.045435 |
| Linnarsson_MouseBrainAtlas_level5 | PSPEP2 | 15140 | 0.0369 | 0.0218 | 0.045591 |
| Linnarsson_GSE60361_Mouse_Cortex_Hippocampus_level2 | CA1Pyr1 | 14010 | 0.0405 | 0.024 | 0.045625 |
| MouseCellAtlas_Embryo_all | Embryonic_Mesenchyme.Neuronal_Progenitors | 14459 | 0.142 | 0.0842 | 0.046015 |
| MouseCellAtlas_all | Neonatal_Muscle.Mesenchymal_cell | 14459 | 0.155 | 0.0919 | 0.046029 |
| MouseCellAtlas_all | Mammary_Gland.B_cell_Ly6d_high | 14459 | 0.148 | 0.0879 | 0.046069 |
| DropViz_PC_level2 | Neuron_Gad1Gad2_Synpr-Sncg.4_5 | 14805 | 0.0102 | 0.00609 | 0.046842 |
| MouseCellAtlas_all | Uterus.Endothelial_cell_Cldn5_high | 14459 | 0.116 | 0.069 | 0.04685 |
| Allen_Mouse_ALM_level2 | Oligo | 14744 | 0.0096 | 0.00573 | 0.046948 |
| DropViz_all_level2 | SN.Endothelial_Tip_Dcn_Alcam.14_3 | 14805 | 0.00441 | 0.00263 | 0.046971 |
| MouseCellAtlas_Neonatal_Muscle | Endothelial_cell | 14459 | 0.151 | 0.0903 | 0.047119 |
| Linnarsson_MouseBrainAtlas_level5 | SCINH11 | 15140 | 0.0792 | 0.0474 | 0.047275 |
| Linnarsson_MouseBrainAtlas_level5 | ENT6 | 15140 | 0.0477 | 0.0285 | 0.047308 |
| MouseCellAtlas_all | Neonatal_Rib.Stromal_cell_Tnmd_high | 14459 | 0.12 | 0.0718 | 0.047515 |
| MouseCellAtlas_Embryo_all | Fetal_Intestine.Stromal_cell_Stmn2_high_ | 14459 | 0.229 | 0.137 | 0.047676 |
| MouseCellAtlas_Adult_all | Uterus.Endothelial_cell_Cldn5_high | 14459 | 0.114 | 0.0683 | 0.047722 |
| MouseCellAtlas_Neonatal_all | Neonatal_Skin.Endothelial_cell | 14459 | 0.139 | 0.0834 | 0.047745 |
| DropViz_HC_level2 | Endothelial_Stalk_Flt1_Cdkn2b.15_1 | 14805 | 0.00742 | 0.00445 | 0.04777 |
| Allen_Mouse_ALM_level2 | SMC | 14744 | 0.00822 | 0.00494 | 0.047914 |
| DropViz_STR_level1 | Endothelial_stalk | 14805 | 0.00994 | 0.00598 | 0.048278 |
| Linnarsson_GSE76381_Human_Midbrain | DA1 | 14835 | 0.075 | 0.0451 | 0.048323 |
| TabulaMuris_FACS_Aorta | smooth_muscle_cell | 13888 | 0.0287 | 0.0173 | 0.048323 |
| MouseCellAtlas_all | Neonatal_Skin.Endothelial_cell | 14459 | 0.118 | 0.0709 | 0.048594 |
| GSE99235_Mouse_Lung_Vascular | CP1 | 14559 | 0.0117 | 0.00704 | 0.048605 |
| DropViz_SN_level1 | Endothelial_tip | 14805 | 0.00883 | 0.00532 | 0.048628 |
| TabulaMuris_FACS_Lung | stromal_cell | 13888 | 0.0127 | 0.00767 | 0.048721 |
| Linnarsson_MouseBrainAtlas_level5 | PSPEP3 | 15140 | 0.0363 | 0.0219 | 0.049012 |
| MouseCellAtlas_Liver | Erythroblast_Hbb.bt_high | 14459 | 0.119 | 0.072 | 0.049073 |
| MouseCellAtlas_Bladder | Epithelial_cell_Gm23935_high | 14459 | 0.141 | 0.0854 | 0.049426 |
| Linnarsson_MouseBrainAtlas_level5 | VECC | 15140 | 0.0463 | 0.0281 | 0.049465 |
| MouseCellAtlas_Adult_all | Mammary_Gland.T_cell_Grwd1_high | 14459 | 0.0635 | 0.0385 | 0.049519 |
| DropViz_all_level2 | GP.Mitotic_MkI67.4_6 | 14805 | 0.00458 | 0.00278 | 0.04967 |
| MouseCellAtlas_Neonatal_Skin | Muscle_cell_Actc1_high | 14459 | 0.162 | 0.0985 | 0.049731 |
| DropViz_FC_level2 | Microglia_C1qb_Tmem119.11_1 | 14805 | 0.00934 | 0.00567 | 0.049829 |
| DropViz_GP_level2 | Endothelial_Tip_Dcn_Mgp.9_1 | 14805 | 0.00668 | 0.00406 | 0.049941 |
| DropViz_all_level2 | FC.Endothelial_Stalk_Flt1_Lrg1.12_5 | 14805 | 0.00562 | 0.00342 | 0.050269 |
| TabulaMuris_droplet_Liver | endothelial_cell | 13888 | 0.0792 | 0.0483 | 0.050623 |
| DropViz_HC_level2 | Microglia_C1qb_Tmem119.10_1 | 14805 | 0.00743 | 0.00454 | 0.050874 |
| Allen_Mouse_ALM_level2 | Vip_Gpc3_2 | 14744 | 0.0203 | 0.0124 | 0.050958 |
| DropViz_all_level2 | ENT.Endothelial_Stalk_Flt1_Car4.2_1 | 14805 | 0.0069 | 0.00422 | 0.050998 |
| DropViz_ENT_level2 | Polydendrocyte_Tnr_Ctps.5_3 | 14805 | 0.00844 | 0.00516 | 0.051027 |
| GSE82187_Mouse_Striatum | OPC | 13103 | 0.037 | 0.0227 | 0.051172 |
| DropViz_PC_level2 | Microglia_C1qb_Tmem119.11_1 | 14805 | 0.00923 | 0.00565 | 0.051217 |
| GSE92332_Mouse_Epithelium_SMARTseq | Endocrine | 13578 | 0.0221 | 0.0135 | 0.051291 |
| MouseCellAtlas_all | Mammary_Gland.T_cell_Grwd1_high | 14459 | 0.0627 | 0.0384 | 0.051333 |
| GSE67835_Human_Cortex | fetal_replicating | 16585 | 0.0142 | 0.00872 | 0.051554 |
| Linnarsson_GSE76381_Human_Midbrain | DA0 | 14835 | 0.114 | 0.0698 | 0.051561 |
| TabulaMuris_FACS_all | Brain_Neurons.smooth_muscle_cell | 13888 | 0.00884 | 0.00542 | 0.051594 |
| DropViz_ENT_level1 | Endothelial_stalk | 14805 | 0.00933 | 0.00573 | 0.05172 |
| Linnarsson_MouseBrainAtlas_level5 | ENTG1 | 15140 | 0.0308 | 0.0189 | 0.05173 |
| DropViz_GP_level2 | Polydendrocyte_Tnr_Pdgfa-Pik3r3.4_1 | 14805 | 0.00894 | 0.0055 | 0.051955 |
| MouseCellAtlas_all | Uterus.Stromal_cell_Hsd11b2_high | 14459 | 0.115 | 0.0708 | 0.052056 |
| Linnarsson_GSE78845_Mouse_Ganglia | Glutamatergic_neurons_ | 12817 | 0.0348 | 0.0214 | 0.052058 |
| DropViz_GP_level2 | Endothelial_Stalk_Flt1_Car4.7_5 | 14805 | 0.00781 | 0.0048 | 0.052092 |
| DropViz_FC_level2 | Endothelial_Stalk_Flt1_Lrg1.12_5 | 14805 | 0.00542 | 0.00334 | 0.052381 |
| MouseCellAtlas_Neonatal_Heart | Endothelial_cell_Enpp2_high | 14459 | 0.149 | 0.0917 | 0.052608 |
| MouseCellAtlas_Neonatal_Skin | Stromal_cell_Akr1c18_high | 14459 | 0.123 | 0.0758 | 0.05279 |
| Linnarsson_MouseBrainAtlas_level5 | ENT7 | 15140 | 0.0397 | 0.0245 | 0.052827 |
| DropViz_CB_level1 | Endothelial_stalk | 14805 | 0.00828 | 0.00512 | 0.052861 |
| MouseCellAtlas_all | Embryonic_Mesenchyme.Stromal_Cell_fabp7_high | 14459 | 0.161 | 0.0997 | 0.052909 |
| DropViz_all_level2 | HC.Endothelial_Stalk_Flt1_Cdkn2b.15_1 | 14805 | 0.0078 | 0.00485 | 0.05392 |
| MouseCellAtlas_Fetal_Intestine | Enterocyte_progenitor_late_Ube2c_high | 14459 | 0.308 | 0.191 | 0.054065 |
| TabulaMuris_droplet_Tongue | basal_cell_of_epidermis | 13888 | 0.118 | 0.0735 | 0.054162 |
| DropViz_all_level2 | TH.Oligodendrocyte_Trf_Ctps.8_3 | 14805 | 0.00894 | 0.00557 | 0.054355 |
| DropViz_PC_level2 | Neuron_Slc17a7_Bcl6-Ndst4.3_6 | 14805 | 0.00713 | 0.00445 | 0.054547 |
| TabulaMuris_droplet_all | Muscle.endothelial_cell | 13888 | 0.0785 | 0.0492 | 0.055498 |
| MouseCellAtlas_Liver | Stromal_cell | 14459 | 0.108 | 0.0677 | 0.055593 |
| MouseCellAtlas_Neonatal_Rib | Oligodendrocyte | 14459 | 0.102 | 0.0642 | 0.05608 |
| DropViz_all_level2 | SN.Macrophage_C1qb_Mrc1.9_1 | 14805 | 0.00589 | 0.00371 | 0.056168 |
| DropViz_all_level1 | GP.Endothelial_stalk | 14805 | 0.00975 | 0.00614 | 0.056214 |
| MouseCellAtlas_Fetal_Lung | Stromal_cell_Ankfy1_high | 14459 | 0.422 | 0.266 | 0.056307 |
| DropViz_all_level2 | FC.Microglia_C1qb_Tmem119.11_1 | 14805 | 0.00896 | 0.00565 | 0.056316 |
| MouseCellAtlas_Adult_all | Uterus.Stromal_cell_Hsd11b2_high | 14459 | 0.11 | 0.0693 | 0.0566 |
| DropViz_all_level2 | GP.Endothelial_Stalk_Flt1_Apold1.7_1 | 14805 | 0.00697 | 0.0044 | 0.056743 |
| MouseCellAtlas_Liver | Kuppfer_cell | 14459 | 0.146 | 0.0924 | 0.056836 |
| TabulaMuris_FACS_Trachea | stromal_cell | 13888 | 0.0171 | 0.0108 | 0.05717 |
| GSE104276_Human_Prefrontal_cortex_per_ages | GW10_Stem_cells | 17199 | 0.018 | 0.0114 | 0.057209 |
| MouseCellAtlas_Adult_all | Pancreas.Endothelial_cell_Lrg1_high | 14459 | 0.0962 | 0.061 | 0.057367 |
| MouseCellAtlas_all | Bone_Marrow.Endothelial_cells_Ly6c1_high | 14459 | 0.169 | 0.107 | 0.057391 |
| TabulaMuris_droplet_all | Muscle.chondroblast | 13888 | 0.0438 | 0.0278 | 0.057878 |
| MouseCellAtlas_Neonatal_all | Neonatal_Skin.Smooth_muscle_cell_Acta2_high | 14459 | 0.16 | 0.102 | 0.057962 |
| DropViz_all_level2 | SN.Polydendrocyte_Tnr_Opalin.5_3 | 14805 | 0.00729 | 0.00464 | 0.058031 |
| DropViz_HC_level2 | Oligodendrocyte_Trf_Man1a.8_4 | 14805 | 0.00807 | 0.00513 | 0.058096 |
| Linnarsson_MouseBrainAtlas_level6_rank2 | Vascular_cells | 15140 | 0.0642 | 0.0409 | 0.05822 |
| Allen_Mouse_ALM_level2 | Vip_Mybpc1_1 | 14744 | 0.0187 | 0.0119 | 0.058246 |
| MouseCellAtlas_all | Pancreas.Endothelial_cell_Lrg1_high | 14459 | 0.0955 | 0.061 | 0.058867 |
| MouseCellAtlas_all | Neonatal_Muscle.Glial_cell | 14459 | 0.142 | 0.0909 | 0.059188 |
| Allen_Mouse_LGd_level1 | OPC | 14545 | 0.0129 | 0.00824 | 0.059239 |
| MouseCellAtlas_Mammary_Gland | Mammary_Gland_Lactation.Stromal_cell | 14459 | 0.217 | 0.139 | 0.059367 |
| TabulaMuris_FACS_all | Pancreas.endothelial_cell | 13888 | 0.0117 | 0.00748 | 0.059523 |
| DropViz_SN_level2 | Endothelial_Tip_Dcn_Ifitm1.14_2 | 14805 | 0.00595 | 0.00382 | 0.060044 |
| DropViz_ENT_level2 | Oligodendrocyte_Trf_Ctps.1_5 | 14805 | 0.00911 | 0.00587 | 0.060292 |
| DropViz_ENT_level2 | Endothelial_Stalk_Flt1_Vwf.2_2 | 14805 | 0.0065 | 0.00419 | 0.060494 |
| DropViz_TH_level2 | Endothelial_Stalk_Flt1_Car4.4_3 | 14805 | 0.0076 | 0.00491 | 0.060596 |
| MouseCellAtlas_all | Fetal_Kidney.Stromal_cell_Mgp_high | 14459 | 0.0453 | 0.0293 | 0.060853 |
| DropViz_FC_level2 | Neuron_Slc17a7_Nptxr-Drd1.6_6 | 14805 | 0.00423 | 0.00274 | 0.060996 |
| DropViz_all_level2 | HC.Neuron_Slc17a7_Fezf2-Rxfp1.5_15 | 14805 | 0.011 | 0.00709 | 0.061006 |
| DropViz_SN_level2 | Endothelial_Tip_Dcn_Alcam.14_3 | 14805 | 0.00415 | 0.00269 | 0.061068 |
| Linnarsson_MouseBrainAtlas_level5 | PER3 | 15140 | 0.0791 | 0.0512 | 0.061102 |
| TabulaMuris_droplet_Lung | stromal_cell | 13888 | 0.0666 | 0.0432 | 0.061502 |
| Linnarsson_GSE75330_Mouse_Oligodendrocytes | COP | 14541 | 0.0588 | 0.0382 | 0.061905 |
| MouseCellAtlas_Spleen | Dendritic_cell_S100a4_high | 14459 | 0.193 | 0.125 | 0.06209 |
| MouseCellAtlas_Neonatal_all | Neonatal_Heart.Dividing_cell | 14459 | 0.173 | 0.113 | 0.062258 |
| MouseCellAtlas_all | Neonatal_Skin.Smooth_muscle_cell_Acta2_high | 14459 | 0.129 | 0.0844 | 0.062623 |
| MouseCellAtlas_Adult_all | Pancreas.Macrophage | 14459 | 0.132 | 0.086 | 0.062666 |
| MouseCellAtlas_all | Embryonic_Mesenchyme.Neuron_Npy_high | 14459 | 0.126 | 0.0821 | 0.062697 |
| DropViz_SN_level2 | Astrocyte_Gja1_Igfbp2.7_3 | 14805 | 0.00457 | 0.00298 | 0.06276 |
| TabulaMuris_droplet_Kidney | endothelial_cell | 13888 | 0.0538 | 0.0351 | 0.062792 |
| GSE98816_Mouse_Brain_Vascular | FB1 | 14130 | 0.0135 | 0.00882 | 0.062922 |
| Linnarsson_GSE74672_Mouse_Hypothalamus_Neurons_level2 | Vglut2_1_Penk | 14550 | 0.0294 | 0.0192 | 0.062948 |
| Linnarsson_MouseBrainAtlas_level5 | PSPEP6 | 15140 | 0.0476 | 0.0311 | 0.062985 |
| DropViz_STR_level2 | Neuron_Slc17a7_Dkk3.13_8 | 14805 | 0.00599 | 0.00391 | 0.062992 |
| DropViz_HC_level2 | Endothelial_Stalk_Flt1_Lcn2.15_2 | 14805 | 0.00585 | 0.00382 | 0.063012 |
| TabulaMuris_droplet_all | Lung.stromal_cell | 13888 | 0.0669 | 0.0438 | 0.063216 |
| DropViz_all_level2 | PC.Endothelial_Tip_Dcn_Inmt.14_5 | 14805 | 0.0051 | 0.00334 | 0.063346 |
| DropViz_all_level2 | PC.Microglia_C1qb_Tmem119.11_1 | 14805 | 0.00856 | 0.00561 | 0.063355 |
| MouseCellAtlas_Adult_all | Bladder.Endothelial_cell_Ly6c1_high | 14459 | 0.091 | 0.0597 | 0.063674 |
| GSE87544_Mouse_Hypothalamus | Epith2 | 13878 | 0.0398 | 0.0261 | 0.063705 |
| Linnarsson_MouseBrainAtlas_level6_rank3 | Vascular_cells | 15140 | 0.051 | 0.0335 | 0.063843 |
| MouseCellAtlas_Adult_all | Mammary_Gland.T_cell | 14459 | 0.142 | 0.0933 | 0.064315 |
| Linnarsson_GSE75330_Mouse_Oligodendrocytes | NFOL2 | 14541 | 0.0612 | 0.0403 | 0.064425 |
| MouseCellAtlas_Embryo_all | Fetal_Kidney.Stromal_cell_Mgp_high | 14459 | 0.0443 | 0.0292 | 0.064638 |
| DropViz_PC_level2 | Endothelial_Tip_Dcn_Inmt.14_5 | 14805 | 0.00488 | 0.00323 | 0.06544 |
| MouseCellAtlas_Embryo_all | Embryonic_Mesenchyme.Stromal_Cell_Cxcl14_high | 14459 | 0.196 | 0.13 | 0.065544 |
| MouseCellAtlas_Adult_all | Liver.Endothelial_cell | 14459 | 0.112 | 0.0746 | 0.065764 |
| MouseCellAtlas_Kidney | S3_proximal_tubule_cells | 14459 | 0.0889 | 0.059 | 0.065919 |
| Linnarsson_GSE76381_Human_Midbrain | Gaba | 14835 | 0.0751 | 0.0499 | 0.066046 |
| GSE106678_Mouse_Cortex | Ex7 | 14573 | 0.22 | 0.146 | 0.066413 |
| TabulaMuris_droplet_all | Kidney.endothelial_cell | 13888 | 0.0428 | 0.0285 | 0.066583 |
| Linnarsson_GSE101601_Mouse_Somatosensory_cortex | Endothelial | 14550 | 0.0514 | 0.0343 | 0.066907 |
| DropViz_all_level2 | PC.Neuron_Gad1Gad2_Synpr-Sncg.4_5 | 14805 | 0.00877 | 0.00586 | 0.067055 |
| MouseCellAtlas_all | Uterus.Stromal_cell_Cxcl14_high | 14459 | 0.151 | 0.101 | 0.067068 |
| MouseCellAtlas_Adult_all | Liver.Dendritic_cell_Siglech_high | 14459 | 0.108 | 0.0719 | 0.067091 |
| GSE92332_Mouse_Epithelium_SMARTseq | Tuft | 13578 | 0.0116 | 0.00779 | 0.067723 |
| MouseCellAtlas_all | Uterus.Stromal_cell_Gm23935_high | 14459 | 0.101 | 0.0678 | 0.068026 |
| MouseCellAtlas_Fetal_Brain | Postmitotic_neurons_Lhx9_high | 14459 | 0.254 | 0.17 | 0.068076 |
| DropViz_all_level2 | HC.Microglia_C1qb_Tmem119.10_1 | 14805 | 0.00705 | 0.00473 | 0.068248 |
| DropViz_CB_level1 | Endothelial_tip | 14805 | 0.00697 | 0.00468 | 0.068307 |
| DropViz_HC_level1 | Microglia | 14805 | 0.00836 | 0.00561 | 0.06831 |
| MouseCellAtlas_Fetal_Stomache | Progenitor_cell | 14459 | 0.314 | 0.211 | 0.068391 |
| MouseCellAtlas_all | Bladder.Endothelial_cell_Ly6c1_high | 14459 | 0.088 | 0.0592 | 0.068678 |
| Allen_Mouse_ALM_level2 | Vip_Chat_2 | 14744 | 0.0161 | 0.0108 | 0.068773 |
| MouseCellAtlas_all | Neonatal_Skin.Dividing_cell | 14459 | 0.141 | 0.0951 | 0.068851 |
| MouseCellAtlas_all | Mammary_Gland.T_cell | 14459 | 0.138 | 0.093 | 0.069044 |
| MouseCellAtlas_all | Neonatal_Rib.Stromal_cell_Acta1_high | 14459 | 0.105 | 0.0707 | 0.069165 |
| DropViz_all_level2 | FC.Neuron_Slc17a7_Nptxr-Drd1.6_6 | 14805 | 0.00398 | 0.00268 | 0.069212 |
| MouseCellAtlas_Embryonic_Mesenchyme | Ganglion_cell_Gal_high | 14459 | 0.117 | 0.079 | 0.069481 |
| DropViz_STR_level2 | Oligodendrocyte_Trf_Ctps.3_3 | 14805 | 0.00795 | 0.00537 | 0.06964 |
| TabulaMuris_droplet_Bladder | mesenchymal_cell | 13888 | 0.0479 | 0.0325 | 0.069961 |
| GSE82187_Mouse_Striatum | Macrophage | 13103 | 0.0375 | 0.0254 | 0.069966 |
| DropViz_GP_level2 | Mitotic_MkI67.4_6 | 14805 | 0.00414 | 0.00281 | 0.070362 |
| MouseCellAtlas_Mammary_Gland | Mammary_Gland_Virgin.Dendritic_cell_Siglech_high | 14459 | 0.09 | 0.0611 | 0.070441 |
| MouseCellAtlas_Adult_all | Lung.Conventional_dendritic_cell_Gngt2_high | 14459 | 0.129 | 0.0879 | 0.070628 |
| GSE104276_Human_Prefrontal_cortex_per_ages | GW16_GABAergic_neurons | 17199 | 0.0204 | 0.0139 | 0.071049 |
| DropViz_all_level2 | FC.Endothelial_Stalk_Flt1_Cfh.12_4 | 14805 | 0.00748 | 0.0051 | 0.071199 |
| DropViz_GP_level1 | Endothelial_tip | 14805 | 0.00843 | 0.00575 | 0.071383 |
| DropViz_all_level2 | HC.Endothelial_Stalk_Flt1_Lcn2.15_2 | 14805 | 0.00597 | 0.00408 | 0.071722 |
| MouseCellAtlas_Embryo_all | Embryonic_Mesenchyme.Ganglion_cell_Gal_high | 14459 | 0.105 | 0.072 | 0.071749 |
| MouseCellAtlas_Ovary | Large_luteal_cell | 14459 | 0.132 | 0.0901 | 0.072205 |
| MouseCellAtlas_Adult_all | Bone_Marrow.Endothelial_cells_Ly6c1_high | 14459 | 0.146 | 0.1 | 0.072316 |
| Allen_Mouse_VISp_level2 | Vip_Chat | 13868 | 0.0207 | 0.0142 | 0.072945 |
| TabulaMuris_FACS_all | Aorta.endothelial_cell | 13888 | 0.0132 | 0.00905 | 0.073137 |
| Linnarsson_MouseBrainAtlas_level6_rank4 | Pspn | 15140 | 0.0408 | 0.0281 | 0.073244 |
| Linnarsson_GSE76381_Mouse_Midbrain | Endo | 14550 | 0.039 | 0.0269 | 0.073371 |
| MouseCellAtlas_all | Pancreas.Macrophage | 14459 | 0.122 | 0.0839 | 0.073625 |
| MouseCellAtlas_Adult_all | Liver.Stromal_cell | 14459 | 0.0828 | 0.0571 | 0.073632 |
| DropViz_all_level2 | FC.Endothelial_Stalk_Flt1_Gkn3.12_1 | 14805 | 0.00763 | 0.00527 | 0.07385 |
| MouseCellAtlas_Peripheral_Blood | Macrophage_S100a4_high | 14459 | 0.151 | 0.104 | 0.073925 |
| MouseCellAtlas_Peripheral_Blood | B_cell_Ly6d_high | 14459 | 0.155 | 0.107 | 0.074046 |
| MouseCellAtlas_Neonatal_Muscle | Neuron | 14459 | 0.149 | 0.103 | 0.07407 |
| MouseCellAtlas_Adult_all | Pancreas.Macrophage_Ly6c2_high | 14459 | 0.103 | 0.0709 | 0.074172 |
| DropViz_all_level2 | HC.Oligodendrocyte_Trf_Man1a.8_4 | 14805 | 0.00809 | 0.0056 | 0.074256 |
| Allen_Mouse_ALM_level2 | Astro | 14744 | 0.00869 | 0.00602 | 0.074262 |
| MouseCellAtlas_Adult_all | Uterus.Stromal_cell_Gm23935_high | 14459 | 0.0956 | 0.0662 | 0.07438 |
| MouseCellAtlas_Uterus | Stromal_cell_Hsd11b2_high | 14459 | 0.16 | 0.111 | 0.074578 |
| MouseCellAtlas_Bone_Marrow | Bone_Marrow_Mesenchyme.Fibroblasts | 14459 | 0.0756 | 0.0524 | 0.074599 |
| GSE89164_Mouse_Hindbrain | Endothelial_cells | 12262 | 0.122 | 0.0847 | 0.074626 |
| DropViz_FC_level2 | Endothelial_Stalk_Flt1_Cfh.12_4 | 14805 | 0.00704 | 0.00489 | 0.074895 |
| Linnarsson_GSE104323_Mouse_Dentate_gyrus | Immature_Pyr | 14909 | 0.0581 | 0.0404 | 0.074943 |
| MouseCellAtlas_Pancreas | Smooth_muscle_cell_Rgs5_high | 14459 | 0.119 | 0.0823 | 0.075029 |
| MouseCellAtlas_Embryo_all | Embryonic_Mesenchyme.Stromal_cell_Cxcl12_high | 14459 | 0.246 | 0.171 | 0.075463 |
| TabulaMuris_droplet_all | Bladder.leukocyte | 13888 | 0.0515 | 0.0359 | 0.075517 |
| Linnarsson_GSE59739_Mouse_Dorsal_root_ganglion_level1 | Tyrosine_hydroxylase_containing | 13848 | 0.0262 | 0.0183 | 0.075844 |
| DropViz_SN_level2 | Endothelial_Stalk_Flt1_Car4.12_3 | 14805 | 0.00687 | 0.0048 | 0.076125 |
| Allen_Mouse_VISp_level1 | Gluta_L2 | 13868 | 0.0176 | 0.0123 | 0.076153 |
| GSE67835_Human_Cortex_woFetal | OPC | 16585 | 0.0131 | 0.00915 | 0.076256 |
| MouseCellAtlas_Neonatal_Rib | Muscle_cell_Actc1_high | 14459 | 0.0828 | 0.0579 | 0.076295 |
| DropViz_CB_level2 | Endothelial_Stalk_Flt1_Car4.10_2 | 14805 | 0.00601 | 0.00422 | 0.077062 |
| TabulaMuris_FACS_all | Heart.endothelial_cell | 13888 | 0.0134 | 0.00939 | 0.077268 |
| DropViz_FC_level2 | Endothelial_Stalk_Flt1_Gkn3.12_1 | 14805 | 0.00718 | 0.00505 | 0.077315 |
| DropViz_all_level2 | STR.Endothelial_Stalk_Flt1_Gkn3.7_2 | 14805 | 0.00627 | 0.00441 | 0.077394 |
| MouseCellAtlas_Adult_all | Uterus.Stromal_cell_Cxcl14_high | 14459 | 0.138 | 0.0968 | 0.077414 |
| MouseCellAtlas_all | Embryonic_Mesenchyme.Neuronal_Progenitors | 14459 | 0.107 | 0.0755 | 0.077692 |
| Linnarsson_GSE103840_Mouse_Dorsal_horn | Glut_Cck_Cpne4 | 14550 | 0.0863 | 0.0607 | 0.077732 |
| MouseCellAtlas_all | Neonatal_Calvaria.Stromal_cell_Col1a1_high | 14459 | 0.106 | 0.075 | 0.078527 |
| Allen_Mouse_VISp_level2 | Vip_Gpc3 | 13868 | 0.0198 | 0.014 | 0.078667 |
| Allen_Mouse_ALM_level2 | L5_ALM_Trh | 14744 | 0.00837 | 0.00592 | 0.078782 |
| GSE87544_Mouse_Hypothalamus | IMO | 13878 | 0.0548 | 0.0388 | 0.078908 |
| Linnarsson_MouseBrainAtlas_level5 | OBNBL3 | 15140 | 0.0741 | 0.0525 | 0.078966 |
| DropViz_ENT_level2 | Endothelial_Stalk_Flt1_Car4.2_1 | 14805 | 0.00688 | 0.00488 | 0.079199 |
| GSE93374_Mouse_Arc_ME_level1 | EndothelialCells | 13325 | 0.0692 | 0.0491 | 0.079396 |
| MouseCellAtlas_Fetal_Brain | Neural_progenitor_cell | 14459 | 0.276 | 0.196 | 0.07941 |
| DropViz_FC_level2 | Endothelial_Tip_Dcn_Coch.14_3 | 14805 | 0.00533 | 0.00378 | 0.079457 |
| MouseCellAtlas_Spleen | Monocyte | 14459 | 0.146 | 0.104 | 0.079592 |
| DropViz_all_level2 | FC.Endothelial_Tip_Dcn_Coch.14_3 | 14805 | 0.00542 | 0.00385 | 0.079872 |
| GSE81547_Human_Pancreas | mesenchymal | 16741 | 0.0104 | 0.00739 | 0.080199 |
| MouseCellAtlas_all | Liver.Endothelial_cell | 14459 | 0.102 | 0.0723 | 0.080247 |
| MouseCellAtlas_Neonatal_Rib | Osteoblast | 14459 | 0.0954 | 0.068 | 0.080314 |
| GSE87544_Mouse_Hypothalamus | OPC | 13878 | 0.0756 | 0.0539 | 0.080379 |
| MouseCellAtlas_Liver | Erythroblast_Hbb.bs_high | 14459 | 0.211 | 0.151 | 0.080679 |
| Allen_Mouse_VISp_level2 | L5b_Tph2 | 13868 | 0.0172 | 0.0123 | 0.080738 |
| Allen_Mouse_LGd_level2 | Olig1_Opalin | 14545 | 0.0087 | 0.00623 | 0.081357 |
| DropViz_all_level2 | PC.Neuron_Slc17a7_Bcl6-Ndst4.3_6 | 14805 | 0.00579 | 0.00415 | 0.081371 |
| Linnarsson_MouseBrainAtlas_level5 | ENT2 | 15140 | 0.0323 | 0.0232 | 0.081667 |
| Allen_Mouse_VISp_level2 | L6a_Car12 | 13868 | 0.0175 | 0.0126 | 0.081751 |
| DropViz_all_level1 | FC.Endothelial_stalk | 14805 | 0.00892 | 0.00643 | 0.082777 |
| DropViz_HC_level2 | Neuron_Slc17a7_Ptgfr.5_13 | 14805 | 0.00762 | 0.00551 | 0.083397 |
| MouseCellAtlas_Lung | Stromal_cell_Dcn_high | 14459 | 0.0827 | 0.0599 | 0.083648 |
| MouseCellAtlas_Kidney | Fenestrated_endothelial_cell_Plvap_high | 14459 | 0.141 | 0.103 | 0.083916 |
| MouseCellAtlas_Lung | B_Cell | 14459 | 0.145 | 0.105 | 0.083981 |
| MouseCellAtlas_Embryo_all | Fetal_Stomache.Stromal_cell_Dcn_high | 14459 | 0.243 | 0.176 | 0.084217 |
| MouseCellAtlas_Embryonic_Mesenchyme | Stromal_Cell_Cxcl14_high | 14459 | 0.202 | 0.147 | 0.084281 |
| MouseCellAtlas_all | Neonatal_Heart.Ventricle_cardiomyocyte_Kcnj8_high | 14459 | 0.129 | 0.0936 | 0.084335 |
| MouseCellAtlas_Peripheral_Blood | Macrophage_Ace_high | 14459 | 0.143 | 0.104 | 0.084365 |
| MouseCellAtlas_Mammary_Gland | Mammary_Gland_Pregnancy.T_cell_Grwd1_high | 14459 | 0.0516 | 0.0375 | 0.084401 |
| MouseCellAtlas_all | Liver.Dendritic_cell_Siglech_high | 14459 | 0.0949 | 0.0691 | 0.08481 |
| MouseCellAtlas_Bone_Marrow | Bone_Marrow_Mesenchyme.MSC | 14459 | 0.0998 | 0.0728 | 0.085365 |
| MouseCellAtlas_all | Mammary_Gland.Muscle_cell_Inmt_high | 14459 | 0.095 | 0.0694 | 0.085499 |
| MouseCellAtlas_all | Liver.Stromal_cell | 14459 | 0.0765 | 0.0559 | 0.085524 |
| TabulaMuris_FACS_Heart | endothelial_cell | 13888 | 0.0151 | 0.011 | 0.085536 |
| Linnarsson_GSE76381_Mouse_Midbrain | NbDA | 14550 | 0.102 | 0.0743 | 0.085665 |
| DroNc_Mouse_Hippocampus | OPC | 12425 | 0.234 | 0.171 | 0.085732 |
| MouseCellAtlas_Adult_all | Mammary_Gland.Muscle_cell_Inmt_high | 14459 | 0.0941 | 0.0689 | 0.08603 |
| MouseCellAtlas_all | Pancreas.Macrophage_Ly6c2_high | 14459 | 0.0944 | 0.0692 | 0.086248 |
| DropViz_all_level2 | TH.Endothelial_Stalk_Flt1_Pi16.4_2 | 14805 | 0.00386 | 0.00283 | 0.086274 |
| DropViz_all_level2 | PC.Endothelial_Stalk_Flt1_Gkn3.12_4 | 14805 | 0.00612 | 0.00449 | 0.086327 |
| MouseCellAtlas_Bone_Marrow | Bone_Marrow_Mesenchyme.Stromal_cell_Col1a2_high | 14459 | 0.0755 | 0.0554 | 0.086536 |
| MouseCellAtlas_Lung | Stromal_cell_Inmt_high | 14459 | 0.0899 | 0.0662 | 0.087415 |
| MouseCellAtlas_Embryo_all | Fetal_Brain.Hippocampus_neurons_Asic4_high | 14459 | 0.134 | 0.0988 | 0.087687 |
| DropViz_all_level2 | GP.Neuron_Chat-Gad1Gad2-Slc17a8_Ngfr.1_1 | 14805 | 0.00911 | 0.00673 | 0.08805 |
| MouseCellAtlas_all | Lung.Conventional_dendritic_cell_Gngt2_high | 14459 | 0.114 | 0.0844 | 0.088127 |
| GSE93374_Mouse_Arc_ME_level2 | Fibroblasts1 | 13325 | 0.0676 | 0.05 | 0.088273 |
| MouseCellAtlas_Embryo_all | Fetal_Intestine.Enterocyte_progenitor_late_Ube2c_high | 14459 | 0.194 | 0.144 | 0.088289 |
| TabulaMuris_FACS_all | Bladder.mesenchymal_cell | 13888 | 0.00867 | 0.00643 | 0.088889 |
| MouseCellAtlas_Adult_all | Liver.Erythroblast_Hbb.bt_high | 14459 | 0.0764 | 0.0567 | 0.089048 |
| MouseCellAtlas_Neonatal_all | Neonatal_Skin.Dividing_cell | 14459 | 0.152 | 0.113 | 0.089291 |
| MouseCellAtlas_Mammary_Gland | Mammary_Gland_Involution.Stromal_cell | 14459 | 0.0848 | 0.0632 | 0.089648 |
| MouseCellAtlas_Neonatal_Muscle | Muscle_cell_Actc1_high | 14459 | 0.12 | 0.0893 | 0.089755 |
| TabulaMuris_FACS_Diaphragm | endothelial_cell | 13888 | 0.015 | 0.0111 | 0.089777 |
| TabulaMuris_droplet_all | Heart.endocardial_cell | 13888 | 0.0411 | 0.0307 | 0.089891 |
| Allen_Mouse_VISp_level2 | OPC_Pdgfra | 13868 | 0.0107 | 0.00798 | 0.089907 |
| DropViz_all_level1 | CB.Endothelial_stalk | 14805 | 0.0067 | 0.005 | 0.090078 |
| MouseCellAtlas_all | Neonatal_Heart.Endothelial_cell_Igfbp5_high | 14459 | 0.131 | 0.0976 | 0.090122 |
| DropViz_all_level2 | TH.Polydendrocyte_Tnr_Pdgfa.12_1 | 14805 | 0.00628 | 0.00469 | 0.09014 |
| MouseCellAtlas_all | Fetal_Stomache.Stomach_cell_Kazald1_high | 14459 | 0.156 | 0.116 | 0.090166 |
| TabulaMuris_FACS_all | Diaphragm.mesenchymal_stem_cell | 13888 | 0.0104 | 0.00779 | 0.090399 |
| DropViz_HC_level2 | Neuron_Slc17a7_Pvrl3-Grm5.6_2 | 14805 | 0.00944 | 0.00707 | 0.090857 |
| MouseCellAtlas_Neonatal_Rib | Neuron_Mpz_high | 14459 | 0.117 | 0.0876 | 0.090906 |
| DropViz_all_level1 | SN.Macrophage | 14805 | 0.00576 | 0.00432 | 0.091411 |
| DropViz_all_level1 | GP.Mitotic | 14805 | 0.00376 | 0.00282 | 0.091502 |
| TabulaMuris_droplet_Mammary | endothelial_cell | 13888 | 0.0485 | 0.0364 | 0.091659 |
| DropViz_HC_level2 | Neuron_Slc17a7_Dcn-Camk2d.5_9 | 14805 | 0.0107 | 0.00806 | 0.091789 |
| DropViz_all_level2 | GP.Endothelial_Stalk_Flt1_Lcn2.7_4 | 14805 | 0.00563 | 0.00426 | 0.092868 |
| DropViz_PC_level2 | Endothelial_Stalk_Flt1_Gkn3.12_4 | 14805 | 0.00556 | 0.00421 | 0.093068 |
| DropViz_GP_level2 | Endothelial_Stalk_Flt1_Apold1.7_1 | 14805 | 0.00602 | 0.00456 | 0.093212 |
| Linnarsson_GSE103840_Mouse_Dorsal_horn | Glut_Nts | 14550 | 0.0814 | 0.0617 | 0.093468 |
| TabulaMuris_FACS_all | Lung.dendritic_cell | 13888 | 0.0106 | 0.00802 | 0.093581 |
| DropViz_TH_level1 | Endothelial_stalk | 14805 | 0.00757 | 0.00574 | 0.093732 |
| Allen_Mouse_ALM_level2 | L5_ALM_Hapln1 | 14744 | 0.00656 | 0.00499 | 0.094028 |
| TabulaMuris_FACS_Kidney | fenestrated_cell | 13888 | 0.0136 | 0.0103 | 0.094305 |
| Allen_Mouse_VISp_level1 | NonNeu_Endo | 13868 | 0.00947 | 0.00721 | 0.094514 |
| DropViz_all_level2 | GP.Neuron_Slc17a6_Cck.2_2 | 14805 | 0.00854 | 0.0065 | 0.09455 |
| TabulaMuris_FACS_Aorta | fibroblast | 13888 | 0.0122 | 0.00932 | 0.094556 |
| MouseCellAtlas_Embryo_all | Fetal_Lung.Erythroblast_Hba.x_high | 14459 | 0.115 | 0.0875 | 0.094622 |
| GSE104276_Human_Prefrontal_cortex_per_ages | GW19_Neurons | 17199 | 0.0162 | 0.0124 | 0.09469 |
| Linnarsson_GSE95315_Mouse_Dentate_gyrus | Pericytes | 11815 | 0.0701 | 0.0534 | 0.094853 |
| MouseCellAtlas_Testis | Spermatids_Cst13_high | 14459 | 0.109 | 0.0834 | 0.095443 |
| GSE93374_Mouse_Arc_ME_level2 | Endothelial | 13325 | 0.0662 | 0.0507 | 0.095769 |
| DropViz_all_level2 | PC.Endothelial_Stalk_Flt1_Car4.12_1 | 14805 | 0.00655 | 0.00503 | 0.096091 |
| MouseCellAtlas_all | Lung.T_Cell_Cd8b1_high | 14459 | 0.103 | 0.0793 | 0.096122 |
| Allen_Mouse_VISp_level2 | Endo_Xdh | 13868 | 0.00899 | 0.00689 | 0.096169 |
| DroNc_Mouse_Hippocampus | exPFC5 | 12425 | 0.298 | 0.229 | 0.096537 |
| DropViz_SN_level2 | Macrophage_C1qb_Mrc1.9_1 | 14805 | 0.00505 | 0.00388 | 0.096579 |
| Allen_Mouse_VISp_level1 | NonNeu_OPC | 13868 | 0.0111 | 0.0085 | 0.096706 |
| TabulaMuris_droplet_Muscle | chondroblast | 13888 | 0.0475 | 0.0366 | 0.096837 |
| MouseCellAtlas_Bladder | Stromal_cell_Car3_high | 14459 | 0.0701 | 0.0541 | 0.097283 |
| DropViz_all_level1 | HC.Endothelial_stalk | 14805 | 0.00749 | 0.0058 | 0.098323 |
| TabulaMuris_droplet_Kidney | fibroblast | 13888 | 0.0568 | 0.044 | 0.098374 |
| MouseCellAtlas_Embryonic_Mesenchyme | Stromal_cell_Cxcl12_high | 14459 | 0.262 | 0.203 | 0.098405 |
| MouseCellAtlas_all | Pancreas.Acinar_cell | 14459 | 0.0626 | 0.0485 | 0.0986 |
| DropViz_STR_level2 | Polydendrocye_Tnr_Pdgfa.5_3 | 14805 | 0.00584 | 0.00453 | 0.099009 |
| TabulaMuris_FACS_all | Aorta.epicardial_adipocyte | 13888 | 0.0246 | 0.0192 | 0.099571 |
| DropViz_HC_level2 | Polydendrocyte_Tnr_Ptprz1.9_4 | 14805 | 0.00717 | 0.00559 | 0.099736 |
| Linnarsson_GSE60361_Mouse_Cortex_Hippocampus_level2 | CA1PyrInt | 14010 | 0.0368 | 0.0288 | 0.10051 |
| MouseCellAtlas_Adult_all | Pancreas.Acinar_cell | 14459 | 0.0616 | 0.0483 | 0.10077 |
| MouseCellAtlas_Bone_Marrow | Bone_Marrow_Mesenchyme.Osteoblasts | 14459 | 0.0737 | 0.0577 | 0.10078 |
| MouseCellAtlas_Neonatal_all | Neonatal_Muscle.Glial_cell | 14459 | 0.121 | 0.095 | 0.10082 |
| DropViz_all_level2 | SN.Neuron_Th_Vcan.4_5 | 14805 | 0.00778 | 0.0061 | 0.10114 |
| DropViz_TH_level2 | Oligodendrocyte_Trf_Ctps.8_3 | 14805 | 0.00757 | 0.00594 | 0.10126 |
| DropViz_all_level2 | PC.Endothelial_Stalk_Flt1_Gm9946.12_3 | 14805 | 0.0065 | 0.00511 | 0.10157 |
| MouseCellAtlas_Fetal_Lung | Epithelial_cell_Sftpc_high | 14459 | 0.228 | 0.179 | 0.10164 |
| DropViz_PC_level2 | Endothelial_Stalk_Flt1_Car4.12_1 | 14805 | 0.00594 | 0.00468 | 0.10204 |
| MouseCellAtlas_all | Liver.Erythroblast_Hbb.bt_high | 14459 | 0.0705 | 0.0555 | 0.10211 |
| MouseCellAtlas_Liver | T_cell_Gzma_high | 14459 | 0.131 | 0.104 | 0.10216 |
| MouseCellAtlas_Embryo_all | Embryonic_Mesenchyme.Endothelial_Cell | 14459 | 0.0932 | 0.0736 | 0.10268 |
| MouseCellAtlas_Peripheral_Blood | Macrophage_Flt.ps1_high | 14459 | 0.0989 | 0.0783 | 0.10313 |
| Linnarsson_GSE60361_Mouse_Cortex_Hippocampus_level2 | S1PyrL23 | 14010 | 0.0336 | 0.0266 | 0.10315 |
| MouseCellAtlas_Embryo_all | Fetal_Stomache.Erythroblast_Hbb.bs_high | 14459 | 0.249 | 0.197 | 0.10329 |
| DroNc_Mouse_Hippocampus | END | 12425 | 0.0968 | 0.0767 | 0.10331 |
| MouseCellAtlas_Adult_all | Mammary_Gland.Ductal_luminal_cell | 14459 | 0.151 | 0.12 | 0.10335 |
| TabulaMuris_FACS_Brain_Neurons | endothelial_cell | 13888 | 0.00975 | 0.00772 | 0.10345 |
| DropViz_all_level2 | FC.Endothelial_Stalk_Flt1_Car4.12_3 | 14805 | 0.00669 | 0.0053 | 0.10352 |
| Linnarsson_GSE67602_Mouse_Skin_Epidermis | IFE_DI | 14528 | 0.0759 | 0.0601 | 0.10352 |
| MouseCellAtlas_Adult_all | Lung.Plasmacytoid_dendritic_cell | 14459 | 0.115 | 0.0912 | 0.10353 |
| Linnarsson_GSE59739_Mouse_Dorsal_root_ganglion_level3 | Tyrosine_hydroxylase_containing | 13848 | 0.0169 | 0.0134 | 0.10373 |
| MouseCellAtlas_Adult_all | Lung.Alveolar_macrophage_Pclaf_high | 14459 | 0.0871 | 0.0692 | 0.104 |
| MouseCellAtlas_Adult_all | Lung.T_Cell_Cd8b1_high | 14459 | 0.0967 | 0.0769 | 0.10425 |
| DropViz_all_level2 | GP.Oligodendrocyte_Trf_Ttyh1.10_3 | 14805 | 0.00708 | 0.00563 | 0.10426 |
| MouseCellAtlas_Neonatal_all | Neonatal_Heart.Ventricle_cardiomyocyte_Kcnj8_high | 14459 | 0.131 | 0.105 | 0.10457 |
| MouseCellAtlas_Embryo_all | Embryonic_Mesenchyme.Skeletal_Muscle | 14459 | 0.0949 | 0.0758 | 0.10524 |
| MouseCellAtlas_Neonatal_all | Neonatal_Muscle.Macrophage_Pf4_high | 14459 | 0.0926 | 0.074 | 0.1054 |
| MouseCellAtlas_Adult_all | Mammary_Gland.Mast_cell | 14459 | 0.0801 | 0.064 | 0.10568 |
| Linnarsson_GSE59739_Mouse_Dorsal_root_ganglion_level2 | Tyrosine_hydroxylase_containing | 13848 | 0.0184 | 0.0147 | 0.1057 |
| DropViz_SN_level2 | Neuron_Th_C1ql3.4_4 | 14805 | 0.0069 | 0.00553 | 0.10579 |
| MouseCellAtlas_all | Mammary_Gland.NK_cell_Cd8b1_high | 14459 | 0.0805 | 0.0645 | 0.10582 |
| MouseCellAtlas_all | Bone_Marrow.Megakaryocyte_progenitor_cell | 14459 | 0.0993 | 0.0796 | 0.10609 |
| DropViz_all_level2 | ENT.Endothelial_Tip_Dcn.3_2 | 14805 | 0.00384 | 0.00308 | 0.10619 |
| DropViz_FC_level2 | Endothelial_Stalk_Flt1_Car4.12_3 | 14805 | 0.00633 | 0.00508 | 0.10631 |
| MouseCellAtlas_Mammary_Gland | Mammary_Gland_Lactation.Secretory_alveoli_cell_Cidea.Elovl5_high | 14459 | 0.103 | 0.0829 | 0.1068 |
| MouseCellAtlas_Kidney | Stromal_cell_Ptgds_high | 14459 | 0.114 | 0.0914 | 0.10695 |
| MouseCellAtlas_Fetal_Intestine | Stromal_cell_Adamdec1_high | 14459 | 0.32 | 0.258 | 0.10748 |
| Linnarsson_MouseBrainAtlas_level5 | PSNP4 | 15140 | 0.0193 | 0.0156 | 0.10765 |
| DropViz_PC_level2 | Neuron_Sc17a7_Oprk1.2_15 | 14805 | 0.0102 | 0.00827 | 0.10779 |
| DropViz_HC_level2 | Neuron_Slc17a7_Rgs14.6_4 | 14805 | 0.01 | 0.00809 | 0.10809 |
| DropViz_all_level2 | CB.Endothelial_Stalk_Flt1_Car4.10_2 | 14805 | 0.00448 | 0.00363 | 0.10831 |
| DropViz_PC_level2 | Endothelial_Stalk_Flt1_Gm9946.12_3 | 14805 | 0.00585 | 0.00474 | 0.10839 |
| GSE104276_Human_Prefrontal_cortex_all_ages | GABAergic_neurons | 17199 | 0.0208 | 0.0168 | 0.10851 |
| MouseCellAtlas_Neonatal_all | Neonatal_Calvaria.Stromal_cell_Col3a1_high | 14459 | 0.0986 | 0.08 | 0.10886 |
| MouseCellAtlas_all | Bone_Marrow.Fibroblasts | 14459 | 0.0682 | 0.0553 | 0.10886 |
| Allen_Mouse_LGd_level1 | Olig1 | 14545 | 0.00884 | 0.00718 | 0.10913 |
| Linnarsson_GSE104323_Mouse_Dentate_gyrus | NFOL | 14909 | 0.0638 | 0.0519 | 0.10949 |
| TabulaMuris_FACS_Liver | endothelial_cell_of_hepatic_sinusoid | 13888 | 0.0107 | 0.00869 | 0.10976 |
| Linnarsson_GSE74672_Mouse_Hypothalamus_Neurons_level2 | Hcrt | 14550 | 0.055 | 0.0448 | 0.10983 |
| MouseCellAtlas_Adult_all | Thymus.Pre_T_cell | 14459 | 0.0526 | 0.043 | 0.11033 |
| MouseCellAtlas_all | Fetal_Intestine.Stromal_cell_Stmn2_high_ | 14459 | 0.128 | 0.105 | 0.11065 |
| DropViz_STR_level2 | Endothelial_Stalk_Flt1_Car4.7_1 | 14805 | 0.00602 | 0.00492 | 0.11069 |
| GSE93374_Mouse_Arc_ME_level2 | Mural_Cells1 | 13325 | 0.0664 | 0.0543 | 0.11093 |
| DropViz_PC_level2 | Microglia_C1qb_Tmem119-Junb.11_4 | 14805 | 0.00315 | 0.00258 | 0.11118 |
| MouseCellAtlas_all | Lung.Alveolar_macrophage_Pclaf_high | 14459 | 0.0836 | 0.0685 | 0.11135 |
| TabulaMuris_FACS_Pancreas | endothelial_cell | 13888 | 0.00835 | 0.00685 | 0.11156 |
| Linnarsson_GSE95752_Mouse_Dentate_gyrus | OPC | 12517 | 0.0881 | 0.0725 | 0.11217 |
| GSE104276_Human_Prefrontal_cortex_per_ages | GW23_Neurons | 17199 | 0.0151 | 0.0124 | 0.11234 |
| GSE89232_Human_Blood | CB_pre_cDC | 13922 | 0.0341 | 0.0281 | 0.1124 |
| MouseCellAtlas_all | Embryonic_Mesenchyme.Endothelial_Cell | 14459 | 0.0848 | 0.0699 | 0.11268 |
| MouseCellAtlas_all | Neonatal_Heart.Endothelial_cell_Eln_high | 14459 | 0.0664 | 0.0548 | 0.11275 |
| MouseCellAtlas_all | Neonatal_Muscle.T_cell | 14459 | 0.0871 | 0.0719 | 0.11284 |
| MouseCellAtlas_all | Mammary_Gland.Mast_cell | 14459 | 0.0767 | 0.0634 | 0.11323 |
| GSE104276_Human_Prefrontal_cortex_per_ages | GW16_Neurons | 17199 | 0.0168 | 0.0139 | 0.11339 |
| DropViz_all_level2 | ENT.Neuron_Gad1Gad2_Otx2.4_11 | 14805 | 0.00357 | 0.00296 | 0.11386 |
| TabulaMuris_FACS_Bladder | mesenchymal_cell | 13888 | 0.00807 | 0.00669 | 0.11399 |
| DropViz_all_level2 | PC.Microglia_C1qb_Tmem119-Junb.11_4 | 14805 | 0.00312 | 0.00258 | 0.11409 |
| DropViz_FC_level2 | Neuron_Slc17a6-Slc17a7_Nr4a2.5_1 | 14805 | 0.0112 | 0.00931 | 0.11424 |
| MouseCellAtlas_Small_Intestine | Epithelium_of_small_intestinal_villi_Gm23935_high | 14459 | 0.0966 | 0.0803 | 0.11445 |
| MouseCellAtlas_all | Thymus.Pre_T_cell | 14459 | 0.0515 | 0.0429 | 0.11484 |
| Linnarsson_GSE104323_Mouse_Dentate_gyrus | nIPC | 14909 | 0.0522 | 0.0435 | 0.11518 |
| DropViz_TH_level2 | Endothelial_Stalk_Flt1_Pi16.4_2 | 14805 | 0.00343 | 0.00286 | 0.11561 |
| DropViz_all_level2 | FC.Polydendrocyte_Tnr_Pdgfa.10_3 | 14805 | 0.00654 | 0.00547 | 0.11588 |
| MouseCellAtlas_Adult_all | Mammary_Gland.NK_cell_Cd8b1_high | 14459 | 0.0745 | 0.0623 | 0.1159 |
| DropViz_HC_level2 | Neuron_Slc17a7_Pvrl3-Fos.6_8 | 14805 | 0.00979 | 0.00819 | 0.11597 |
| DroNc_Mouse_Hippocampus | exCA1 | 12425 | 0.22 | 0.184 | 0.11637 |
| MouseCellAtlas_Mammary_Gland | Mammary_Gland_Virgin.Stromal_cell_Col3a1_high | 14459 | 0.0675 | 0.0567 | 0.11669 |
| DropViz_all_level2 | SN.Oligodendrocyte_Tfr_Ctps.10_5 | 14805 | 0.0065 | 0.00546 | 0.11686 |
| DropViz_HC_level2 | Neuron_Slc17a7_Fibcd1-Lypd1.5_1 | 14805 | 0.0124 | 0.0104 | 0.11723 |
| TabulaMuris_FACS_Lung | dendritic_cell | 13888 | 0.0099 | 0.00833 | 0.11733 |
| TabulaMuris_FACS_all | Trachea.epithelial_cell | 13888 | 0.0188 | 0.0159 | 0.11849 |
| Linnarsson_GSE67602_Mouse_Skin_Epidermis | OB | 14528 | 0.0646 | 0.0546 | 0.11866 |
| TabulaMuris_droplet_all | Kidney.fibroblast | 13888 | 0.0345 | 0.0293 | 0.11919 |
| DropViz_SN_level2 | Polydendrocyte_Tnr_Opalin.5_3 | 14805 | 0.00577 | 0.0049 | 0.11945 |
| MouseCellAtlas_all | Mammary_Gland.Ductal_luminal_cell | 14459 | 0.137 | 0.116 | 0.11956 |
| MouseCellAtlas_all | Embryonic_Mesenchyme.Ganglion_cell_Gal_high | 14459 | 0.0737 | 0.0627 | 0.11971 |
| Allen_Mouse_LGd_level2 | Micro_Ctss | 14545 | 0.00627 | 0.00533 | 0.11972 |
| DropViz_all_level2 | HC.Neuron_Slc17a7_Fezf2-Efna5.5_14 | 14805 | 0.00895 | 0.00761 | 0.11976 |
| Linnarsson_MouseBrainAtlas_level6_rank4 | Gsat | 15140 | 0.0374 | 0.0318 | 0.11986 |
| MouseCellAtlas_all | Fetal_Intestine.Neuronal_Cells_Rtn1_high | 14459 | 0.0983 | 0.0837 | 0.12034 |
| DropViz_all_level1 | CB.Endothelial_tip | 14805 | 0.00506 | 0.00431 | 0.12045 |
| Allen_Mouse_LGd_level1 | Micro | 14545 | 0.00665 | 0.00567 | 0.12047 |
| MouseCellAtlas_Neonatal_all | Neonatal_Heart.Endothelial_cell_Igfbp5_high | 14459 | 0.13 | 0.111 | 0.12048 |
| MouseCellAtlas_Embryo_all | Placenta.Stromal_cell_Acta2_high | 14459 | 0.0864 | 0.0737 | 0.12049 |
| MouseCellAtlas_Embryo_all | Fetal_Brain.Postmitotic_neurons_Lhx9_high | 14459 | 0.117 | 0.1 | 0.1205 |
| Allen_Mouse_VISp_level2 | L5b_Cdh13 | 13868 | 0.0158 | 0.0135 | 0.1206 |
| Linnarsson_GSE60361_Mouse_Cortex_Hippocampus_level2 | ClauPyr | 14010 | 0.0196 | 0.0167 | 0.1206 |
| MouseCellAtlas_Fetal_Stomache | Endocrine_progenitor_cell | 14459 | 0.132 | 0.112 | 0.12094 |
| GSE104276_Human_Prefrontal_cortex_per_ages | GW10_Neurons | 17199 | 0.0143 | 0.0123 | 0.12096 |
| MouseCellAtlas_all | Lung.Plasmacytoid_dendritic_cell | 14459 | 0.103 | 0.0882 | 0.12111 |
| MouseCellAtlas_Adult_all | Bone_Marrow.Fibroblasts | 14459 | 0.0625 | 0.0534 | 0.12114 |
| DropViz_FC_level2 | Polydendrocyte_Tnr_Pdgfa.10_3 | 14805 | 0.00603 | 0.00517 | 0.12143 |
| Linnarsson_MouseBrainAtlas_level5 | MFOL1 | 15140 | 0.0323 | 0.0277 | 0.12146 |
| Linnarsson_GSE104323_Mouse_Dentate_gyrus | Ependymal | 14909 | 0.0546 | 0.0468 | 0.12148 |
| MouseCellAtlas_Bone_Marrow | Bone_Marrow_Mesenchyme.CXCL12.abundant_reticular_cell | 14459 | 0.0727 | 0.0625 | 0.1223 |
| Linnarsson_GSE95315_Mouse_Dentate_gyrus | VLMC | 11815 | 0.0492 | 0.0423 | 0.12236 |
| DropViz_PC_level1 | Endothelial_stalk | 14805 | 0.00752 | 0.00647 | 0.12252 |
| DropViz_SN_level1 | Endothelial_stalk | 14805 | 0.00695 | 0.00599 | 0.12303 |
| MouseCellAtlas_Adult_all | Thymus.T_cell_Id2_high | 14459 | 0.0702 | 0.0607 | 0.12379 |
| MouseCellAtlas_Prostate | Glandular_epithelium | 14459 | 0.258 | 0.224 | 0.1238 |
| TabulaMuris_droplet_Lung | endothelial_cell | 13888 | 0.0471 | 0.0408 | 0.12397 |
| MouseCellAtlas_all | Placenta.Stromal_cell_Acta2_high | 14459 | 0.0817 | 0.0708 | 0.12411 |
| MouseCellAtlas_all | Fetal_Stomache.Stromal_cell_Cdkn1c_high | 14459 | 0.142 | 0.123 | 0.12454 |
| MouseCellAtlas_Embryonic_Mesenchyme | Endothelial_Cell | 14459 | 0.0884 | 0.0768 | 0.12486 |
| MouseCellAtlas_Embryonic_Mesenchyme | Skeletal_Muscle | 14459 | 0.0933 | 0.0811 | 0.12494 |
| Linnarsson_MouseBrainAtlas_level6_rank4 | Gepe | 15140 | 0.0348 | 0.0302 | 0.1251 |
| Linnarsson_GSE95315_Mouse_Dentate_gyrus | NFOL | 11815 | 0.0577 | 0.0502 | 0.12511 |
| GSE67835_Human_Cortex | fetal_quiescent | 16585 | 0.0103 | 0.009 | 0.12531 |
| DropViz_GP_level1 | Mitotic | 14805 | 0.00347 | 0.00303 | 0.12571 |
| MouseCellAtlas_all | Embryonic_Mesenchyme.Skeletal_Muscle | 14459 | 0.0799 | 0.0698 | 0.12605 |
| MouseCellAtlas_Embryo_all | Fetal_Intestine.Endothelial_cell | 14459 | 0.108 | 0.0948 | 0.12656 |
| MouseCellAtlas_Bone_Marrow | Bone_Marrow_Mesenchyme.Granulocyte_monocyte_progenitors | 14459 | 0.0734 | 0.0644 | 0.1272 |
| DropViz_STR_level2 | Macrophage_C1qb_Mrc1.6_1 | 14805 | 0.00345 | 0.00303 | 0.12726 |
| GSE99235_Mouse_Lung_Vascular | FB4 | 14559 | 0.0104 | 0.00913 | 0.12804 |
| GSE104276_Human_Prefrontal_cortex_per_ages | GW16_Microglia | 17199 | 0.00798 | 0.00704 | 0.12823 |
| DropViz_all_level1 | PC.Endothelial_stalk | 14805 | 0.00735 | 0.00648 | 0.12838 |
| MouseCellAtlas_all | Thymus.T_cell_Id2_high | 14459 | 0.0686 | 0.0606 | 0.12879 |
| MouseCellAtlas_all | Neonatal_Muscle.Muscle_cell_Myl9_high | 14459 | 0.0876 | 0.0774 | 0.12884 |
| MouseCellAtlas_all | Embryonic_Mesenchyme.Stromal_Cell_Cxcl14_high | 14459 | 0.115 | 0.102 | 0.12893 |
| MouseCellAtlas_Muscle | Endothelial_cell | 14459 | 0.0807 | 0.0714 | 0.12918 |
| MouseCellAtlas_Adult_all | Pancreas.Erythroblast_Hbb.bt_high | 14459 | 0.066 | 0.0584 | 0.12925 |
| MouseCellAtlas_all | Neonatal_Calvaria.Stromal_cell_Col3a1_high | 14459 | 0.0776 | 0.0688 | 0.12946 |
| MouseCellAtlas_Adult_all | Pancreas.Ductal_cell | 14459 | 0.0907 | 0.0805 | 0.13007 |
| MouseCellAtlas_Adult_all | Mammary_Gland.Macrophage_Pf4_high | 14459 | 0.0786 | 0.0699 | 0.13032 |
| MouseCellAtlas_Neonatal_Calvaria | Muscle_cell_Actc1_high | 14459 | 0.091 | 0.0809 | 0.13045 |
| MouseCellAtlas_Liver | Macrophage_Chil3_high | 14459 | 0.13 | 0.115 | 0.13055 |
| MouseCellAtlas_all | Pancreas.Ductal_cell | 14459 | 0.0911 | 0.0811 | 0.13077 |
| MouseCellAtlas_Adult_all | Bone_Marrow.Megakaryocyte_progenitor_cell | 14459 | 0.0824 | 0.0734 | 0.13092 |
| MouseCellAtlas_Uterus | Stromal_cell_Gm23935_high | 14459 | 0.114 | 0.101 | 0.13098 |
| DropViz_all_level2 | HC.Neuron_Slc17a7_Calb2-Vgll3.6_5 | 14805 | 0.0072 | 0.00644 | 0.13166 |
| GSE104276_Human_Prefrontal_cortex_per_ages | GW26_Neurons | 17199 | 0.0147 | 0.0131 | 0.13191 |
| TabulaMuris_droplet_all | Lung.endothelial_cell | 13888 | 0.0456 | 0.0408 | 0.13219 |
| MouseCellAtlas_Mammary_Gland | Mammary_Gland_Pregnancy.Mast_cell | 14459 | 0.0534 | 0.0479 | 0.13235 |
| MouseCellAtlas_Small_Intestine | Paneth_cell | 14459 | 0.0803 | 0.072 | 0.13244 |
| Linnarsson_MouseBrainAtlas_level6_rank4 | Psnn | 15140 | 0.0206 | 0.0185 | 0.13255 |
| MouseCellAtlas_Fetal_Intestine | Endothelial_cell | 14459 | 0.113 | 0.101 | 0.13287 |
| DropViz_PC_level2 | Polydendrocyte_Tnr_Cspg5.10_1 | 14805 | 0.00674 | 0.00606 | 0.13303 |
| GSE104276_Human_Prefrontal_cortex_per_ages | GW26_Stem_cells | 17199 | 0.00633 | 0.00569 | 0.13304 |
| DropViz_all_level2 | GP.Mural_Rgs5Acta2_Pappa2.8_5 | 14805 | 0.00291 | 0.00262 | 0.13309 |
| MouseCellAtlas_all | Pancreas.Erythroblast_Hbb.bt_high | 14459 | 0.0646 | 0.0583 | 0.1338 |
| MouseCellAtlas_Kidney | Proximal_tubule_cell_Cyp4a14_high | 14459 | 0.0845 | 0.0762 | 0.13384 |
| MouseCellAtlas_all | Bone_Marrow.MSC | 14459 | 0.0874 | 0.0789 | 0.13393 |
| Allen_Mouse_ALM_level1 | L5_Tshz2 | 14744 | 0.00899 | 0.00812 | 0.13403 |
| Linnarsson_MouseBrainAtlas_level5 | TEGLU19 | 15140 | 0.0436 | 0.0394 | 0.13423 |
| Linnarsson_GSE60361_Mouse_Cortex_Hippocampus_level2 | Oligo1 | 14010 | 0.0215 | 0.0194 | 0.13474 |
| DropViz_HC_level2 | Mural_Rgs5Acta2_Ccnd1.16_2 | 14805 | 0.00461 | 0.00418 | 0.13485 |
| MouseCellAtlas_Adult_all | Peripheral_Blood.B_cell_Rps27rt_high | 14459 | 0.0859 | 0.0779 | 0.13502 |
| Allen_Mouse_ALM_level2 | L2.3_ALM_Agmat | 14744 | 0.0103 | 0.00933 | 0.13543 |
| DropViz_all_level2 | ENT.Polydendrocyte_Tnr_Pdgfa.5_2 | 14805 | 0.00499 | 0.00454 | 0.13543 |
| MouseCellAtlas_Brain | Oligodendrocyte_precursor_cell | 14459 | 0.0929 | 0.0846 | 0.13606 |
| Linnarsson_MouseBrainAtlas_level5 | HBCHO1 | 15140 | 0.0246 | 0.0224 | 0.13632 |
| DropViz_HC_level2 | Neuron_Slc17a7_Dcn-Pou3f1.5_6 | 14805 | 0.0114 | 0.0104 | 0.13635 |
| MouseCellAtlas_Lung | Conventional_dendritic_cell_Gngt2_high | 14459 | 0.114 | 0.104 | 0.13673 |
| Linnarsson_GSE74672_Mouse_Hypothalamus_Neurons_level2 | Sst_2_high | 14550 | 0.0308 | 0.0281 | 0.13678 |
| MouseCellAtlas_Neonatal_Rib | Muscle_cell_Acta2_high | 14459 | 0.0786 | 0.0718 | 0.13692 |
| Linnarsson_GSE101601_Mouse_Somatosensory_cortex | Myelin.forming_oligodendrotye | 14550 | 0.0417 | 0.0381 | 0.13713 |
| MouseCellAtlas_Mammary_Gland | Mammary_Gland_Pregnancy.Endothelial_cell | 14459 | 0.0609 | 0.0557 | 0.1373 |
| Allen_Mouse_ALM_level2 | Vip_Prss12_1 | 14744 | 0.0103 | 0.00947 | 0.13731 |
| MouseCellAtlas_Adult_all | Pancreas.Granulocyte | 14459 | 0.0427 | 0.0391 | 0.1374 |
| MouseCellAtlas_all | Neonatal_Skin.Epithelial_cell | 14459 | 0.104 | 0.0953 | 0.13773 |
| Linnarsson_MouseBrainAtlas_level5 | HBCHO3 | 15140 | 0.0211 | 0.0193 | 0.13776 |
| MouseCellAtlas_Neonatal_all | Neonatal_Heart.Endothelial_cell_Eln_high | 14459 | 0.0637 | 0.0584 | 0.13782 |
| MouseCellAtlas_all | Neonatal_Muscle.Skeletal_muscle_cell_Tnnc2_high | 14459 | 0.0364 | 0.0334 | 0.13793 |
| MouseCellAtlas_Thymus | Pre_T_cell | 14459 | 0.062 | 0.0569 | 0.13796 |
| MouseCellAtlas_all | Bone_Marrow.Stromal_cell_Col1a2_high | 14459 | 0.0634 | 0.0582 | 0.13809 |
| DropViz_all_level2 | GP.Endothelial_Tip_Dcn_Ifitm1.9_3 | 14805 | 0.00411 | 0.00378 | 0.13836 |
| Allen_Mouse_LGd_level2 | Olig1_Rassf10 | 14545 | 0.00664 | 0.00612 | 0.13886 |
| MouseCellAtlas_Adult_all | Liver.T_cell_Gzma_high | 14459 | 0.0822 | 0.0758 | 0.13896 |
| MouseCellAtlas_all | Neonatal_Skin.Erythroblast | 14459 | 0.0875 | 0.0806 | 0.139 |
| MouseCellAtlas_Adult_all | Mammary_Gland.Endothelial_cell_Aqp1_high | 14459 | 0.0706 | 0.0652 | 0.13943 |
| MouseCellAtlas_Adult_all | Lung.AT2_Cell | 14459 | 0.0636 | 0.0587 | 0.13956 |
| DropViz_GP_level2 | Endothelial_Stalk_Flt1_Lcn2.7_4 | 14805 | 0.0048 | 0.00444 | 0.13974 |
| DropViz_CB_level2 | Endothelial_Stalk_Flt1_Lrg1.10_3 | 14805 | 0.00454 | 0.0042 | 0.13979 |
| MouseCellAtlas_all | Mammary_Gland.Endothelial_cell_Aqp1_high | 14459 | 0.0706 | 0.0655 | 0.14065 |
| MouseCellAtlas_Peripheral_Blood | Dendritic_cell_Siglech_high | 14459 | 0.124 | 0.115 | 0.14113 |
| TabulaMuris_FACS_all | Fat.endothelial_cell | 13888 | 0.0109 | 0.0101 | 0.1412 |
| Linnarsson_GSE78845_Mouse_Ganglia | Noradregergic_neurons_4 | 12817 | 0.0447 | 0.0416 | 0.14122 |
| DropViz_PC_level2 | Neuron_Sc17a7_Sema3e-Nnat.2_17 | 14805 | 0.0126 | 0.0117 | 0.14124 |
| MouseCellAtlas_all | Peripheral_Blood.B_cell_Rps27rt_high | 14459 | 0.0833 | 0.0775 | 0.14129 |
| DropViz_PC_level2 | Mural_Acta2Rgs5_Kcnj8-Abcc9.13_4 | 14805 | 0.00364 | 0.00339 | 0.14141 |
| GSE89164_Mouse_Hindbrain | Excitatory_neurons | 12262 | 0.151 | 0.141 | 0.14141 |
| GSE104276_Human_Prefrontal_cortex_per_ages | GW12_OPC | 17199 | 0.0049 | 0.00456 | 0.14148 |
| MouseCellAtlas_Neonatal_Skin | Muscle_cell_Lrrc15_high | 14459 | 0.119 | 0.111 | 0.14197 |
| MouseCellAtlas_Embryo_all | Fetal_Stomache.Progenitor_cell | 14459 | 0.19 | 0.178 | 0.14297 |
| Linnarsson_MouseBrainAtlas_level5 | HBGLU3 | 15140 | 0.055 | 0.0516 | 0.14298 |
| Linnarsson_GSE76381_Mouse_Midbrain | NbM | 14550 | 0.0626 | 0.0587 | 0.14315 |
| DropViz_all_level2 | PC.Polydendrocyte_Tnr_Cspg5.10_1 | 14805 | 0.00677 | 0.00635 | 0.14317 |
| MouseCellAtlas_all | Pancreas.Granulocyte | 14459 | 0.0414 | 0.0389 | 0.14346 |
| GSE81547_Human_Pancreas | ductal | 16741 | 0.00942 | 0.00887 | 0.14398 |
| DropViz_STR_level2 | Oligodendrocyte_Trf_Il33.3_2 | 14805 | 0.0067 | 0.00631 | 0.14418 |
| MouseCellAtlas_Stomach | Muscle_cell | 14459 | 0.0668 | 0.0629 | 0.14429 |
| DropViz_all_level2 | PC.Mural_Acta2Rgs5_Kcnj8-Abcc9.13_4 | 14805 | 0.0037 | 0.00349 | 0.14437 |
| Linnarsson_MouseBrainAtlas_level5 | EPSC | 15140 | 0.029 | 0.0274 | 0.14439 |
| DropViz_PC_level2 | Endothelial_Tip_Dcn_Nnat.14_3 | 14805 | 0.00402 | 0.00379 | 0.14452 |
| TabulaMuris_droplet_Muscle | endothelial_cell | 13888 | 0.0579 | 0.0547 | 0.14484 |
| MouseCellAtlas_all | Lung.AT2_Cell | 14459 | 0.0619 | 0.0585 | 0.14503 |
| MouseCellAtlas_Fetal_Brain | Dopaminergic_neurons_ | 14459 | 0.149 | 0.141 | 0.1452 |
| TabulaMuris_FACS_Skin | basal_cell_of_epidermis | 13888 | 0.0202 | 0.0191 | 0.14552 |
| DroNc_Mouse_Hippocampus | exPFC7 | 12425 | 0.192 | 0.182 | 0.14583 |
| DropViz_all_level1 | HC.Microglia | 14805 | 0.00573 | 0.00544 | 0.14601 |
| DropViz_all_level2 | SN.Mural_Rgs5Acta2_Rgs5.13_1 | 14805 | 0.00459 | 0.00436 | 0.14602 |
| DropViz_FC_level2 | Mural_Rgs5Acta2_Col3a1.13_4 | 14805 | 0.00377 | 0.00358 | 0.14603 |
| Linnarsson_GSE59739_Mouse_Dorsal_root_ganglion_level3 | Non_peptidergic_nocieptor2 | 13848 | 0.0124 | 0.0118 | 0.14643 |
| DropViz_all_level2 | FC.Mural_Rgs5Acta2_Col3a1.13_4 | 14805 | 0.00385 | 0.00366 | 0.14648 |
| MouseCellAtlas_Embryo_all | Embryonic_Mesenchyme.Neuron_Igfbpl1_high | 14459 | 0.123 | 0.117 | 0.14666 |
| DropViz_all_level2 | SN.Neuron_Th_Cbln1.4_2 | 14805 | 0.0052 | 0.00495 | 0.14676 |
| DropViz_STR_level1 | Macrophage | 14805 | 0.0036 | 0.00343 | 0.1471 |
| Linnarsson_GSE103840_Mouse_Dorsal_horn | Glut_Tac1_1 | 14550 | 0.0667 | 0.0639 | 0.14803 |
| DropViz_all_level2 | FC.Oligodendrocyte_Tnf_Igsf8.9_1 | 14805 | 0.00643 | 0.00616 | 0.14815 |
| MouseCellAtlas_Adult_all | Liver.Kuppfer_cell | 14459 | 0.0643 | 0.0616 | 0.1483 |
| MouseCellAtlas_all | Neonatal_Muscle.Dendritic_cell | 14459 | 0.0746 | 0.0715 | 0.14844 |
| DropViz_ENT_level2 | Endothelial_Tip_Dcn.3_2 | 14805 | 0.00346 | 0.00332 | 0.14867 |
| MouseCellAtlas_Adult_all | Pancreas.T_cell | 14459 | 0.0701 | 0.0673 | 0.14873 |
| DropViz_all_level2 | PC.Endothelial_Tip_Dcn_Nnat.14_3 | 14805 | 0.00404 | 0.00389 | 0.14911 |
| MouseCellAtlas_Embryo_all | Fetal_Intestine.Stromal_cell_Adamdec1_high | 14459 | 0.186 | 0.179 | 0.14927 |
| DropViz_HC_level2 | Neuron_Slc17a7_Fibcd1-Kitl.5_4 | 14805 | 0.00983 | 0.00946 | 0.14936 |
| DropViz_all_level2 | ENT.Oligodendrocyte_Trf_Il33.1_4 | 14805 | 0.00544 | 0.00523 | 0.14948 |
| MouseCellAtlas_Embryonic_Mesenchyme | Neuron_Igfbpl1_high | 14459 | 0.14 | 0.134 | 0.14948 |
| MouseCellAtlas_Bone_Marrow | Bone_Marrow_Mesenchyme.Endothelial_cells | 14459 | 0.0777 | 0.075 | 0.15026 |
| MouseCellAtlas_Kidney | Stromal_cell_Dcn_high | 14459 | 0.0648 | 0.0627 | 0.15047 |
| DropViz_TH_level2 | Polydendrocyte_Tnr_Pdgfa.12_1 | 14805 | 0.00508 | 0.00491 | 0.15048 |
| Linnarsson_GSE101601_Mouse_Somatosensory_cortex | Pyramidal_cells_layer_23 | 14550 | 0.0466 | 0.0451 | 0.1506 |
| Linnarsson_MouseBrainAtlas_level5 | TEGLU16 | 15140 | 0.0335 | 0.0324 | 0.15087 |
| MouseCellAtlas_Neonatal_all | Neonatal_Muscle.Muscle_cell_Myl9_high | 14459 | 0.0918 | 0.0889 | 0.15103 |
| DropViz_all_level2 | GP.Endothelial_Stalk_Flt1_Angpt2.7_6 | 14805 | 0.00469 | 0.00455 | 0.15124 |
| DropViz_PC_level2 | Neuron_Slc17a7_Bcl6-Pvalb.3_9 | 14805 | 0.00944 | 0.00917 | 0.15169 |
| GSE89164_Mouse_Hindbrain | Inhibitory_neurons | 12262 | 0.162 | 0.158 | 0.1519 |
| MouseCellAtlas_Fetal_Liver | Stem_and_progenitor_cell | 14459 | 0.146 | 0.142 | 0.15206 |
| DropViz_all_level2 | HC.Polydendrocyte_Tnr_Ptprz1.9_4 | 14805 | 0.00595 | 0.00581 | 0.15261 |
| MouseCellAtlas_all | Placenta.Labyrinthine_trophoblast | 14459 | 0.0513 | 0.05 | 0.15269 |
| DropViz_all_level2 | SN.Neuron_Th_Nefl.4_7 | 14805 | 0.00618 | 0.00603 | 0.15278 |
| TabulaMuris_FACS_all | Diaphragm.endothelial_cell | 13888 | 0.00962 | 0.00939 | 0.1528 |
| TabulaMuris_FACS_all | Trachea.endothelial_cell | 13888 | 0.00798 | 0.00779 | 0.15283 |
| MouseCellAtlas_all | Mammary_Gland.Macrophage_Pf4_high | 14459 | 0.069 | 0.0674 | 0.15292 |
| DropViz_all_level2 | TH.Astrocyte_Gja1_Vegfa.11_2 | 14805 | 0.00582 | 0.00568 | 0.15296 |
| MouseCellAtlas_all | Embryonic_Mesenchyme.Stromal_cell_Cxcl12_high | 14459 | 0.131 | 0.128 | 0.15299 |
| MouseCellAtlas_all | Fetal_Lung.Erythroblast_Hba.x_high | 14459 | 0.0739 | 0.0722 | 0.15322 |
| MouseCellAtlas_Adult_all | Bone_Marrow.MSC | 14459 | 0.0762 | 0.0746 | 0.15369 |
| Linnarsson_GSE74672_Mouse_Hypothalamus_Neurons_level2 | Vglut2_14_Col9a2 | 14550 | 0.0331 | 0.0324 | 0.15373 |
| MouseCellAtlas_all | Pancreas.T_cell | 14459 | 0.068 | 0.067 | 0.15497 |
| Linnarsson_MouseBrainAtlas_level5 | EPEN | 15140 | 0.0266 | 0.0262 | 0.15519 |
| Linnarsson_MouseBrainAtlas_level5 | PSPEP8 | 15140 | 0.0267 | 0.0263 | 0.15524 |
| DropViz_all_level1 | ENT.Endothelial_tip | 14805 | 0.00352 | 0.00347 | 0.15528 |
| DropViz_CB_level2 | Mural_Acta2Rgs5_Rgs5.11_5 | 14805 | 0.00299 | 0.00296 | 0.15562 |
| MouseCellAtlas_all | Muscle.Endothelial_cell | 14459 | 0.0725 | 0.0716 | 0.15572 |
| DropViz_HC_level2 | Oligodendrocyte_Trf_Ctps.8_5 | 14805 | 0.00345 | 0.00341 | 0.15576 |
| MouseCellAtlas_Adult_all | Muscle.Endothelial_cell | 14459 | 0.072 | 0.0712 | 0.15601 |
| MouseCellAtlas_Adult_all | Pancreas.Dendrtic_cell | 14459 | 0.0755 | 0.0747 | 0.15606 |
| Linnarsson_MouseBrainAtlas_level5 | PSPEP1 | 15140 | 0.0211 | 0.0209 | 0.15632 |
| Allen_Mouse_ALM_level2 | L5_ALM_Arhgap25_3 | 14744 | 0.01 | 0.00995 | 0.15712 |
| MouseCellAtlas_Adult_all | Bone_Marrow.Stromal_cell_Col1a2_high | 14459 | 0.0556 | 0.0554 | 0.15747 |
| DropViz_STR_level2 | Mural_Rgs5Acta2_Col3a1.8_5 | 14805 | 0.00303 | 0.00302 | 0.1575 |
| DropViz_FC_level2 | Oligodendrocyte_Tnf_Igsf8.9_1 | 14805 | 0.00572 | 0.00569 | 0.15757 |
| Linnarsson_MouseBrainAtlas_level5 | DGNBL1 | 15140 | 0.0396 | 0.0395 | 0.15772 |
| TabulaMuris_FACS_Liver | B_cell | 13888 | 0.00992 | 0.0099 | 0.15821 |
| DropViz_GP_level1 | Endothelial_stalk | 14805 | 0.00608 | 0.00607 | 0.15855 |
| GSE67835_Human_Cortex | OPC | 16585 | 0.00882 | 0.00882 | 0.1587 |
| MouseCellAtlas_all | Pancreas.β.cell | 14459 | 0.0598 | 0.0598 | 0.1587 |
| DropViz_all_level2 | SN.Mural_Rgs5Acta2_Kcnj8.13_2 | 14805 | 0.00291 | 0.00291 | 0.15935 |
| MouseCellAtlas_Adult_all | Pancreas.β.cell | 14459 | 0.0587 | 0.0592 | 0.16068 |
| MouseCellAtlas_all | Liver.T_cell_Gzma_high | 14459 | 0.0726 | 0.0733 | 0.16087 |
| MouseCellAtlas_all | Fetal_Intestine.Endothelial_cell | 14459 | 0.0832 | 0.084 | 0.16089 |
| TabulaMuris_FACS_all | Lung.endothelial_cell | 13888 | 0.00971 | 0.00981 | 0.16118 |
| Linnarsson_MouseBrainAtlas_level5 | COP1 | 15140 | 0.0335 | 0.0339 | 0.16154 |
| GSE100597_Mouse_Embryo | E4.5 | 13879 | 0.0125 | 0.0126 | 0.16167 |
| Linnarsson_MouseBrainAtlas_level5 | SATG2 | 15140 | 0.0275 | 0.0279 | 0.16223 |
| MouseCellAtlas_Neonatal_all | Neonatal_Muscle.Skeletal_muscle_cell_Tnnc2_high | 14459 | 0.0336 | 0.0341 | 0.16231 |
| MouseCellAtlas_all | Fetal_Brain.Hippocampus_neurons_Asic4_high | 14459 | 0.0813 | 0.0826 | 0.16231 |
| DroNc_Human_Hippocampus | MG | 16914 | 0.223 | 0.227 | 0.16248 |
| MouseCellAtlas_all | Neonatal_Rib.Endothelial_cell | 14459 | 0.0643 | 0.0655 | 0.16311 |
| DroNc_Mouse_Hippocampus | exPFC3 | 12425 | 0.298 | 0.304 | 0.16319 |
| MouseCellAtlas_Mammary_Gland | Mammary_Gland_Lactation.Secretory_alveoli_cell_Wfdc3.Wap_high | 14459 | 0.054 | 0.0551 | 0.16328 |
| MouseCellAtlas_Neonatal_all | Neonatal_Skin.Epithelial_cell | 14459 | 0.113 | 0.115 | 0.16329 |
| DropViz_all_level2 | STR.Neurogenesis_Sox4_Stmn2.2_2 | 14805 | 0.00392 | 0.004 | 0.16341 |
| MouseCellAtlas_all | Placenta.PE_lineage_cell_Gkn2_high | 14459 | 0.0655 | 0.0669 | 0.16386 |
| MouseCellAtlas_Embryo_all | Fetal_Stomache.Endocrine_progenitor_cell | 14459 | 0.103 | 0.105 | 0.16417 |
| TabulaMuris_FACS_Brain_Neurons | neuronal_stem_cell | 13888 | 0.00626 | 0.00641 | 0.16441 |
| DropViz_all_level2 | HC.Mural_Rgs5Acta2_Ccnd1.16_2 | 14805 | 0.00434 | 0.00445 | 0.16453 |
| TabulaMuris_FACS_Fat | endothelial_cell | 13888 | 0.00958 | 0.00986 | 0.16562 |
| Linnarsson_GSE104323_Mouse_Dentate_gyrus | OPC | 14909 | 0.0811 | 0.0835 | 0.16567 |
| Linnarsson_GSE101601_Human_Temporal_cortex | Glut_3 | 17177 | 0.167 | 0.172 | 0.16568 |
| Linnarsson_GSE103840_Mouse_Dorsal_horn | GABA_Tac2 | 14550 | 0.0392 | 0.0404 | 0.16582 |
| MouseCellAtlas_all | Brain.Pan.GABAergic | 14459 | 0.0676 | 0.0697 | 0.16635 |
| DropViz_GP_level2 | Mural_Rgs5Acta2_Pappa2.8_5 | 14805 | 0.00254 | 0.00263 | 0.16692 |
| DropViz_all_level2 | FC.Polydendrocyte_Tnr_Opalin.10_4 | 14805 | 0.00427 | 0.00442 | 0.16717 |
| DropViz_all_level2 | GP.Oligodendrocyte_Trf_Ctps.10_4 | 14805 | 0.00473 | 0.00491 | 0.1673 |
| Linnarsson_MouseBrainAtlas_level5 | HBGLU1 | 15140 | 0.0246 | 0.0255 | 0.1675 |
| DropViz_all_level2 | HC.Neuron_Slc17a7_Ptgfr.5_13 | 14805 | 0.00492 | 0.00511 | 0.16775 |
| DropViz_all_level2 | GP.Polydendrocyte_Tnr_Pdgfa-Tmem2.4_2 | 14805 | 0.0047 | 0.00488 | 0.16777 |
| MouseCellAtlas_all | Pancreas.Dendrtic_cell | 14459 | 0.0708 | 0.0736 | 0.16796 |
| MouseCellAtlas_Adult_all | Small_Intestine.Paneth_cell | 14459 | 0.0574 | 0.0597 | 0.16811 |
| MouseCellAtlas_Testis | Spermatids_Tnp1_high | 14459 | 0.102 | 0.106 | 0.16852 |
| Linnarsson_GSE59739_Mouse_Dorsal_root_ganglion_level2 | Non_peptidergic_nocieptor2 | 13848 | 0.0125 | 0.013 | 0.16882 |
| MouseCellAtlas_all | Placenta.Stromal_cell | 14459 | 0.087 | 0.0908 | 0.16899 |
| DropViz_all_level2 | GP.Neuron_Slc17a7_Neurod2-C1ql3.2_12 | 14805 | 0.00491 | 0.00513 | 0.16938 |
| DropViz_all_level2 | CB.Endothelial_Stalk_Flt1_Lrg1.10_3 | 14805 | 0.00349 | 0.00365 | 0.16958 |
| MouseCellAtlas_all | Small_Intestine.Mast_cell | 14459 | 0.0409 | 0.0428 | 0.1698 |
| Linnarsson_GSE76381_Human_Midbrain | DA2 | 14835 | 0.0497 | 0.0521 | 0.16999 |
| GSE92332_Mouse_Epithelium_SMARTseq | Goblet | 13578 | 0.00989 | 0.0104 | 0.17021 |
| MouseCellAtlas_Uterus | Endothelial_cell_Cldn5_high | 14459 | 0.0749 | 0.0787 | 0.17052 |
| TabulaMuris_FACS_Colon | Brush_cell_of_epithelium_proper_of_large_intestine | 13888 | 0.00911 | 0.00957 | 0.17075 |
| MouseCellAtlas_Adult_all | Small_Intestine.Mast_cell | 14459 | 0.0404 | 0.0425 | 0.17096 |
| DropViz_FC_level2 | Polydendrocyte_Tnr_Opalin.10_4 | 14805 | 0.00402 | 0.00423 | 0.17097 |
| MouseCellAtlas_Embryo_all | Placenta.Endothelial_cell_Maged2_high | 14459 | 0.0886 | 0.0932 | 0.17102 |
| DropViz_all_level2 | GP.Mural_Rgs5Acta2_Lmcd1.8_3 | 14805 | 0.00296 | 0.00313 | 0.17142 |
| Linnarsson_GSE74672_Mouse_Hypothalamus_Neurons_level2 | Vglut2_17_A930013F10RikPou2f2 | 14550 | 0.018 | 0.019 | 0.17161 |
| TabulaMuris_droplet_all | Mammary.endothelial_cell | 13888 | 0.0297 | 0.0314 | 0.17193 |
| MouseCellAtlas_Bone_Marrow | Bone_Marrow_c_kit.Macrophage_C1qc_high | 14459 | 0.0581 | 0.0614 | 0.1721 |
| MouseCellAtlas_Embryonic_Stem_Cell | ES_Nedd4_high | 14459 | 0.148 | 0.157 | 0.17276 |
| TabulaMuris_FACS_all | Heart.smooth_muscle_cell | 13888 | 0.00796 | 0.00844 | 0.17277 |
| Linnarsson_GSE75330_Mouse_Oligodendrocytes | MFOL1 | 14541 | 0.0454 | 0.0481 | 0.1728 |
| TabulaMuris_droplet_Marrow | monocyte | 13888 | 0.0434 | 0.0461 | 0.17313 |
| DropViz_HC_level2 | Neuron_Slc17a7_Fibcd1-Grm5.5_5 | 14805 | 0.0054 | 0.00573 | 0.17317 |
| Linnarsson_MouseBrainAtlas_level5 | TEGLU13 | 15140 | 0.0336 | 0.0357 | 0.17321 |
| Allen_Mouse_VISp_level2 | L5_Ucma | 13868 | 0.012 | 0.0128 | 0.17362 |
| MouseCellAtlas_Fetal_Liver | Megakaryocyte | 14459 | 0.083 | 0.0886 | 0.17432 |
| MouseCellAtlas_Embryo_all | Fetal_Lung.Stromal_cell_Ptn_high | 14459 | 0.143 | 0.153 | 0.17438 |
| GSE106678_Mouse_Cortex | EC | 14573 | 0.0703 | 0.0751 | 0.17459 |
| MouseCellAtlas_all | Liver.Kuppfer_cell | 14459 | 0.0556 | 0.0594 | 0.17464 |
| MouseCellAtlas_Spleen | Marginal_zone_B_cell | 14459 | 0.123 | 0.131 | 0.17503 |
| DropViz_all_level2 | PC.Oligodendrocyte_Tfr_Ctps.9_1 | 14805 | 0.00568 | 0.00608 | 0.17516 |
| MouseCellAtlas_Uterus | Endothelial_cell_Tm4sf1_high | 14459 | 0.0933 | 0.0999 | 0.17517 |
| MouseCellAtlas_all | Small_Intestine.Paneth_cell | 14459 | 0.0555 | 0.0594 | 0.17524 |
| TabulaMuris_FACS_all | Pancreas.pancreatic_acinar_cell | 13888 | 0.00884 | 0.00949 | 0.17559 |
| DropViz_all_level2 | TH.Polydendrocyte_Tnr_Ctps.12_2 | 14805 | 0.00443 | 0.00477 | 0.17653 |
| DropViz_FC_level1 | Endothelial_tip | 14805 | 0.00637 | 0.00686 | 0.17656 |
| Linnarsson_MouseBrainAtlas_level5 | NFOL1 | 15140 | 0.023 | 0.0247 | 0.17657 |
| MouseCellAtlas_Adult_all | Mammary_Gland.Myeloid_leukocyte | 14459 | 0.0524 | 0.0564 | 0.17669 |
| MouseCellAtlas_Embryo_all | Placenta.Stromal_cell | 14459 | 0.0868 | 0.0935 | 0.17677 |
| MouseCellAtlas_all | Placenta.Endothelial_cell_Maged2_high | 14459 | 0.0818 | 0.0882 | 0.17686 |
| MouseCellAtlas_Neonatal_all | Neonatal_Skin.Erythroblast | 14459 | 0.085 | 0.0916 | 0.1769 |
| MouseCellAtlas_Adult_all | Liver.T_cell_Trbc2_high | 14459 | 0.0845 | 0.0912 | 0.17702 |
| MouseCellAtlas_Ovary | Stroma_cell_ | 14459 | 0.106 | 0.115 | 0.17708 |
| DropViz_all_level2 | FC.Neuron_Slc17a6-Slc17a7_Nr4a2.5_1 | 14805 | 0.00727 | 0.00785 | 0.17711 |
| MouseCellAtlas_Adult_all | Liver.Macrophage_Chil3_high | 14459 | 0.0714 | 0.0771 | 0.1773 |
| DropViz_FC_level2 | Neuron_Gad1Gad2_Cplx3-Dpy19l1.1_8 | 14805 | 0.00499 | 0.00539 | 0.17731 |
| MouseCellAtlas_Kidney | S1_proximal_tubule_cells | 14459 | 0.0663 | 0.0717 | 0.17739 |
| TabulaMuris_FACS_Kidney | leukocyte | 13888 | 0.00803 | 0.00868 | 0.17742 |
| DropViz_all_level2 | ENT.Polydendrocyte_Tnr_Bmp4.5_1 | 14805 | 0.00426 | 0.00461 | 0.17765 |
| MouseCellAtlas_all | Bone_Marrow.Osteoblasts | 14459 | 0.0527 | 0.0571 | 0.17799 |
| MouseCellAtlas_Muscle | Muscle_cell_Tnnc1_high | 14459 | 0.029 | 0.0314 | 0.17821 |
| TabulaMuris_FACS_all | Brain_Neurons.endothelial_cell | 13888 | 0.0073 | 0.00792 | 0.17848 |
| GSE93374_Mouse_Arc_ME_level1 | MuralCells | 13325 | 0.0612 | 0.0664 | 0.17855 |
| DropViz_HC_level2 | Polydendrocyte_Tnr_Pdgfa.9_1 | 14805 | 0.00465 | 0.00505 | 0.17866 |
| MouseCellAtlas_all | Kidney.Stromal_cell_Dcn_high | 14459 | 0.0571 | 0.062 | 0.17877 |
| GSE89232_Human_Blood | Blood_pre_cDC | 13922 | 0.0627 | 0.0682 | 0.17879 |
| TabulaMuris_droplet_Trachea | basal_cell_of_epithelium_of_trachea | 13888 | 0.0677 | 0.0738 | 0.17961 |
| MouseCellAtlas_Placenta | PE_lineage_cell_Gkn2_high | 14459 | 0.0656 | 0.0716 | 0.17972 |
| MouseCellAtlas_Thymus | T_cell_Id2_high | 14459 | 0.0804 | 0.0878 | 0.17977 |
| Linnarsson_GSE95752_Mouse_Dentate_gyrus | OL | 12517 | 0.0299 | 0.0327 | 0.18001 |
| DropViz_all_level2 | TH.Oligodendrocyte_Trf_Ndrg2.8_4 | 14805 | 0.00519 | 0.00568 | 0.18033 |
| GSE99235_Mouse_Lung_Vascular | FB2 | 14559 | 0.00736 | 0.00806 | 0.18077 |
| Linnarsson_GSE74672_Mouse_Hypothalamus_Neurons_level2 | Vglut2_12_Mgat4b | 14550 | 0.0185 | 0.0204 | 0.18131 |
| DropViz_all_level2 | GP.Neuron_Gad1Gad2_Lamp5-Cplx3.2_22 | 14805 | 0.00332 | 0.00365 | 0.18149 |
| MouseCellAtlas_Embryo_all | Embryonic_Mesenchyme.Stromal_Cell_Agtr2_high | 14459 | 0.113 | 0.124 | 0.18171 |
| MouseCellAtlas_Placenta | Stromal_cell_Acta2_high | 14459 | 0.0643 | 0.0708 | 0.18174 |
| DropViz_ENT_level1 | Endothelial_tip | 14805 | 0.00354 | 0.0039 | 0.18206 |
| MouseCellAtlas_Neonatal_all | Neonatal_Rib.Endothelial_cell | 14459 | 0.0705 | 0.0778 | 0.18256 |
| Linnarsson_GSE60361_Mouse_Cortex_Hippocampus_level2 | Epend | 14010 | 0.0227 | 0.0251 | 0.1826 |
| DropViz_all_level2 | CB.Mural_Acta2Rgs5_Rgs5.11_5 | 14805 | 0.00243 | 0.00269 | 0.18272 |
| GSE106678_Mouse_Cortex | Ex8 | 14573 | 0.0852 | 0.0942 | 0.18302 |
| DropViz_GP_level2 | Oligodendrocyte_Trf_Ttyh1.10_3 | 14805 | 0.00536 | 0.00594 | 0.1833 |
| Allen_Mouse_VISp_level2 | SMC_Myl9 | 13868 | 0.00692 | 0.00768 | 0.18377 |
| MouseCellAtlas_Bone_Marrow | Bone_Marrow.B_cell_Igkc_high | 14459 | 0.052 | 0.0577 | 0.18383 |
| MouseCellAtlas_Peripheral_Blood | T_cell_Trbc2_high | 14459 | 0.0866 | 0.0962 | 0.18409 |
| Linnarsson_GSE103840_Mouse_Dorsal_horn | Glut_Tac1_2 | 14550 | 0.0637 | 0.0709 | 0.18464 |
| MouseCellAtlas_Adult_all | Kidney.Stromal_cell_Dcn_high | 14459 | 0.0545 | 0.0608 | 0.18491 |
| Linnarsson_MouseBrainAtlas_level6_rank4 | Ngnb | 15140 | 0.0451 | 0.0503 | 0.18532 |
| GSE93374_Mouse_Arc_ME_level1 | Oligodend1 | 13325 | 0.0615 | 0.0688 | 0.18564 |
| Linnarsson_MouseBrainAtlas_level6_rank3 | Peripheral_sensory_neurons | 15140 | 0.0199 | 0.0222 | 0.1857 |
| DropViz_PC_level2 | Oligodendrocyte_Tfr_Ctps.9_1 | 14805 | 0.00482 | 0.0054 | 0.18604 |
| MouseCellAtlas_Placenta | Labyrinthine_trophoblast | 14459 | 0.0468 | 0.0525 | 0.18636 |
| MouseCellAtlas_Kidney | Epithelial_cell_Cryab_high | 14459 | 0.0635 | 0.0714 | 0.18688 |
| MouseCellAtlas_Adult_all | Mammary_Gland.B_cell_Cd79a.Fcer2a_high | 14459 | 0.0669 | 0.0754 | 0.1875 |
| DropViz_HC_level2 | Neuron_Slc17a7_Cbln4.2_1 | 14805 | 0.00736 | 0.0083 | 0.18752 |
| DropViz_all_level2 | HC.Oligodendrocyte_Trf_Ctps.8_5 | 14805 | 0.00313 | 0.00353 | 0.18763 |
| DropViz_all_level2 | PC.Neuron_Sc17a7_Oprk1.2_15 | 14805 | 0.00606 | 0.00684 | 0.18796 |
| MouseCellAtlas_Neonatal_Skin | Endothelial_cell | 14459 | 0.0757 | 0.0858 | 0.18862 |
| TabulaMuris_FACS_Marrow | Fraction_A_pre.pro_B_cell | 13888 | 0.00859 | 0.00974 | 0.18885 |
| MouseCellAtlas_all | Placenta.NK_cell | 14459 | 0.0542 | 0.0614 | 0.18897 |
| GSE104276_Human_Prefrontal_cortex_per_ages | GW09_GABAergic_neurons | 17199 | 0.00469 | 0.00532 | 0.18912 |
| DropViz_HC_level2 | Neuron_Slc17a7_Fibcd1-Nrip3.5_2 | 14805 | 0.00823 | 0.00937 | 0.18971 |
| MouseCellAtlas_all | Neonatal_Rib.Oligodendrocyte | 14459 | 0.0498 | 0.0568 | 0.19012 |
| Linnarsson_MouseBrainAtlas_level5 | PSPEP4 | 15140 | 0.0231 | 0.0263 | 0.19022 |
| MouseCellAtlas_Adult_all | Brain.Pan.GABAergic | 14459 | 0.0583 | 0.0665 | 0.19022 |
| MouseCellAtlas_all | Mammary_Gland.B_cell_Cd79a.Fcer2a_high | 14459 | 0.0665 | 0.076 | 0.19064 |
| MouseCellAtlas_Kidney | Proximal_tubule_cell_Osgin1_high | 14459 | 0.0703 | 0.0804 | 0.191 |
| MouseCellAtlas_Kidney | Proximal_tubule_brush_border_cell | 14459 | 0.061 | 0.07 | 0.19166 |
| MouseCellAtlas_Adult_all | Bone_Marrow.Osteoblasts | 14459 | 0.0482 | 0.0554 | 0.19178 |
| MouseCellAtlas_all | Neonatal_Muscle.Adipocyte. | 14459 | 0.046 | 0.0528 | 0.1918 |
| MouseCellAtlas_Embryo_all | Fetal_Brain.Pyramidal_neuron_cell | 14459 | 0.0904 | 0.104 | 0.19183 |
| TabulaMuris_FACS_all | Pancreas.pancreatic_stellate_cell | 13888 | 0.00631 | 0.00726 | 0.19217 |
| Linnarsson_MouseBrainAtlas_level5 | SCGLU6 | 15140 | 0.0528 | 0.0607 | 0.19219 |
| MouseCellAtlas_Adult_all | Small_Intestine.Epithelium_of_small_intestinal_villi_Gm23935_high | 14459 | 0.0576 | 0.0662 | 0.19229 |
| DropViz_ENT_level2 | Neuron_Gad1Gad2_Otx2.4_11 | 14805 | 0.00252 | 0.0029 | 0.19253 |
| TabulaMuris_FACS_all | Kidney.fenestrated_cell | 13888 | 0.00741 | 0.00854 | 0.19265 |
| Allen_Mouse_ALM_level2 | OPC | 14744 | 0.00475 | 0.00547 | 0.19293 |
| MouseCellAtlas_all | Neonatal_Skin.Keratinocyte | 14459 | 0.0666 | 0.0769 | 0.1932 |
| Linnarsson_MouseBrainAtlas_level5 | ENT1 | 15140 | 0.0192 | 0.0222 | 0.19325 |
| Allen_Mouse_ALM_level2 | Vip_Sncg_2 | 14744 | 0.00952 | 0.011 | 0.19386 |
| DropViz_GP_level2 | Neuron_Chat-Gad1Gad2-Slc17a8_Ngfr.1_1 | 14805 | 0.00595 | 0.00689 | 0.19401 |
| Allen_Mouse_VISp_level1 | NonNeu_SMC | 13868 | 0.00706 | 0.0082 | 0.19452 |
| DropViz_PC_level2 | Neuron_Slc17a7-Slc17a6_Tshz2-Rasd1.6_3 | 14805 | 0.00733 | 0.00852 | 0.19479 |
| Allen_Mouse_ALM_level2 | Lamp5_Nxph1_2 | 14744 | 0.00924 | 0.0107 | 0.1948 |
| TabulaMuris_FACS_Muscle | endothelial_cell | 13888 | 0.00844 | 0.00982 | 0.19501 |
| DropViz_GP_level2 | Neuron_Slc17a6_Cck.2_2 | 14805 | 0.00575 | 0.0067 | 0.19526 |
| MouseCellAtlas_Adult_all | Spleen.Monocyte | 14459 | 0.0665 | 0.0775 | 0.19561 |
| DropViz_GP_level2 | Endothelial_Tip_Dcn_Ifitm1.9_3 | 14805 | 0.00332 | 0.00387 | 0.19569 |
| MouseCellAtlas_Mammary_Gland | Mammary_Gland_Pregnancy.Macrophage | 14459 | 0.0329 | 0.0383 | 0.19572 |
| Linnarsson_GSE101601_Human_Temporal_cortex | GABA_3 | 17177 | 0.152 | 0.178 | 0.19624 |
| MouseCellAtlas_all | Fetal_Stomache.Stromal_cell_Dcn_high | 14459 | 0.102 | 0.119 | 0.19704 |
| TabulaMuris_FACS_Colon | enteroendocrine_cell | 13888 | 0.00766 | 0.009 | 0.19733 |
| MouseCellAtlas_Embryo_all | Fetal_Brain.Neural_progenitor_cell | 14459 | 0.0839 | 0.0987 | 0.19751 |
| MouseCellAtlas_Testis | Pre.Sertoli_cell_Ctsl_high | 14459 | 0.0712 | 0.0836 | 0.19751 |
| Linnarsson_GSE103840_Mouse_Dorsal_horn | Glut_Tac2_1 | 14550 | 0.0572 | 0.0673 | 0.19769 |
| TabulaMuris_FACS_Heart | smooth_muscle_cell | 13888 | 0.00918 | 0.0108 | 0.19773 |
| DropViz_PC_level2 | Neuron_Slc17a7_Bcl6-Ptgfr.3_7 | 14805 | 0.00309 | 0.00364 | 0.19779 |
| GSE106678_Mouse_Cortex | Inh5 | 14573 | 0.0836 | 0.0986 | 0.19804 |
| MouseCellAtlas_all | Liver.T_cell_Trbc2_high | 14459 | 0.0749 | 0.0884 | 0.19831 |
| MouseCellAtlas_Small_Intestine | Mast_cell | 14459 | 0.0363 | 0.0428 | 0.1986 |
| MouseCellAtlas_Adult_all | Uterus.Macrophage | 14459 | 0.0589 | 0.0695 | 0.19868 |
| MouseCellAtlas_all | Pancreas.Smooth_muscle_cell_Acta2_high | 14459 | 0.0577 | 0.0683 | 0.19907 |
| MouseCellAtlas_Kidney | T_cell | 14459 | 0.0909 | 0.108 | 0.19918 |
| MouseCellAtlas_Stomach | Dividing_cell | 14459 | 0.0871 | 0.103 | 0.19919 |
| MouseCellAtlas_Brain | Pan.GABAergic | 14459 | 0.0535 | 0.0634 | 0.19931 |
| GSE92332_Mouse_Epithelium_droplet | Tuft | 11993 | 0.0408 | 0.0483 | 0.19933 |
| TabulaMuris_FACS_all | Lung.type_I_pneumocyte | 13888 | 0.00304 | 0.0036 | 0.19937 |
| MouseCellAtlas_Adult_all | Lung.NK_Cell | 14459 | 0.0844 | 0.1 | 0.19996 |
| Linnarsson_GSE103840_Mouse_Dorsal_horn | Glut_Gal | 14550 | 0.0339 | 0.0403 | 0.20026 |
| MouseCellAtlas_all | Neonatal_Skin.Macrophage_Pf4_high | 14459 | 0.0535 | 0.0636 | 0.20034 |
| MouseCellAtlas_all | Neonatal_Muscle.Smooth_muscle_cell_Mylk_high | 14459 | 0.0539 | 0.0642 | 0.20044 |
| MouseCellAtlas_Mammary_Gland | Mammary_Gland_Involution.Muscle_cell_Inmt_high | 14459 | 0.0518 | 0.0617 | 0.20065 |
| MouseCellAtlas_all | Neonatal_Skin.Lymphatic_vessel_endothelial_cell | 14459 | 0.0491 | 0.0586 | 0.20096 |
| MouseCellAtlas_all | Thymus.abT_cell | 14459 | 0.0844 | 0.101 | 0.20109 |
| MouseCellAtlas_Small_Intestine | Epithelium_of_small_intestinal_villi_S100g_high | 14459 | 0.0463 | 0.0552 | 0.20125 |
| Linnarsson_GSE103840_Mouse_Dorsal_horn | Glut_Tac2_3 | 14550 | 0.0474 | 0.0567 | 0.20152 |
| Linnarsson_GSE103840_Mouse_Dorsal_horn | Glut_Reln_Nmur2 | 14550 | 0.0587 | 0.0703 | 0.20203 |
| Linnarsson_GSE74672_Mouse_Hypothalamus_Neurons_level2 | Vglut2_18_Zfp458Ppp1r12b | 14550 | 0.0163 | 0.0195 | 0.20203 |
| MouseCellAtlas_all | Fetal_Brain.Postmitotic_neurons_Lhx9_high | 14459 | 0.0683 | 0.0819 | 0.20213 |
| MouseCellAtlas_Adult_all | Thymus.abT_cell | 14459 | 0.0826 | 0.0993 | 0.20262 |
| MouseCellAtlas_Adult_all | Pancreas.Smooth_muscle_cell_Acta2_high | 14459 | 0.0562 | 0.0675 | 0.20274 |
| GSE89164_Mouse_Hindbrain | Oligodendrotype_myelinating | 12262 | 0.0698 | 0.084 | 0.20307 |
| DropViz_all_level2 | FC.Oligodendrocyte_Tnf_Igsf8-Ctps.9_2 | 14805 | 0.00409 | 0.00494 | 0.20383 |
| Linnarsson_GSE104323_Mouse_Dentate_gyrus | RGL_young | 14909 | 0.0555 | 0.0671 | 0.20427 |
| MouseCellAtlas_Embryo_all | Placenta.PE_lineage_cell_Gkn2_high | 14459 | 0.0527 | 0.0637 | 0.20438 |
| MouseCellAtlas_all | Fetal_Intestine.Enterocyte_progenitor_late_Ube2c_high | 14459 | 0.0808 | 0.098 | 0.20498 |
| TabulaMuris_FACS_Skin | keratinocyte_stem_cell | 13888 | 0.0105 | 0.0128 | 0.20506 |
| MouseCellAtlas_Fetal_Brain | Pyramidal_neuron_cell | 14459 | 0.139 | 0.169 | 0.20527 |
| MouseCellAtlas_Lung | AT2_Cell | 14459 | 0.0541 | 0.0658 | 0.20537 |
| MouseCellAtlas_all | Lung.NK_Cell | 14459 | 0.0824 | 0.1 | 0.20585 |
| DropViz_CB_level2 | Microglia_C1qb_Mrc1-Tmem119.5_1 | 14805 | 0.0026 | 0.00318 | 0.20623 |
| MouseCellAtlas_all | Uterus.Keratinocyte | 14459 | 0.0542 | 0.0663 | 0.20676 |
| Linnarsson_MouseBrainAtlas_level5 | PSNP6 | 15140 | 0.0122 | 0.015 | 0.20677 |
| DropViz_all_level2 | FC.Neuron_Gad1Gad2_Cplx3-Dpy19l1.1_8 | 14805 | 0.00419 | 0.00514 | 0.20706 |
| DropViz_STR_level2 | Microglia_C1qb_Tmem119.6_2 | 14805 | 0.00406 | 0.00497 | 0.20721 |
| GSE104276_Human_Prefrontal_cortex_per_ages | GW26_OPC | 17199 | 0.0103 | 0.0127 | 0.20733 |
| DropViz_all_level2 | GP.Microglia_C1qb_Tmem119.11_1 | 14805 | 0.00323 | 0.00396 | 0.20742 |
| DropViz_FC_level2 | Oligodendrocyte_Tnf_Igsf8-Ctps.9_2 | 14805 | 0.00381 | 0.00468 | 0.20776 |
| MouseCellAtlas_Embryo_all | Placenta.Labyrinthine_trophoblast | 14459 | 0.0376 | 0.0463 | 0.20816 |
| TabulaMuris_FACS_Pancreas | pancreatic_acinar_cell | 13888 | 0.00828 | 0.0102 | 0.20828 |
| MouseCellAtlas_all | Liver.Macrophage_Chil3_high | 14459 | 0.0598 | 0.0736 | 0.20829 |
| MouseCellAtlas_Adult_all | Mammary_Gland.NK_cell_Gzma_high | 14459 | 0.074 | 0.0912 | 0.20856 |
| MouseCellAtlas_Adult_all | Mammary_Gland.NK_cells_Gzmb_high | 14459 | 0.0402 | 0.0496 | 0.20889 |
| DropViz_HC_level2 | Neuron_Slc17a7_Pvrl3-St18.6_3 | 14805 | 0.00723 | 0.00893 | 0.20892 |
| MouseCellAtlas_all | Small_Intestine.Epithelium_of_small_intestinal_villi_Gm23935_high | 14459 | 0.0526 | 0.065 | 0.20925 |
| DropViz_PC_level2 | Macrophage_C1qb_Mrc1.11_2 | 14805 | 0.00322 | 0.00398 | 0.20963 |
| GSE93374_Mouse_Arc_ME_level2 | Oligodendro1 | 13325 | 0.0266 | 0.0329 | 0.20968 |
| MouseCellAtlas_all | Mesenchymal_Stem_Cell_Cultured.Mesenchymal_stem_cell_Cxcl10_high | 14459 | 0.0396 | 0.0491 | 0.21044 |
| MouseCellAtlas_Adult_all | Uterus.Keratinocyte | 14459 | 0.0525 | 0.0653 | 0.21053 |
| MouseCellAtlas_Embryo_all | Fetal_Intestine.Stromal_cell_Upk3b_high | 14459 | 0.113 | 0.141 | 0.21082 |
| TabulaMuris_FACS_all | Muscle.endothelial_cell | 13888 | 0.00693 | 0.00862 | 0.21085 |
| DropViz_all_level1 | SN.Astrocyte | 14805 | 0.0048 | 0.00598 | 0.21096 |
| MouseCellAtlas_all | Mammary_Gland.NK_cells_Gzmb_high | 14459 | 0.0404 | 0.0503 | 0.21103 |
| DropViz_all_level2 | HC.Neuron_Slc17a7_Pvrl3-Grm5.6_2 | 14805 | 0.00492 | 0.00613 | 0.21124 |
| DropViz_all_level2 | STR.Neuron_Slc17a7_Dkk3.13_8 | 14805 | 0.00317 | 0.00396 | 0.21159 |
| GSE104276_Human_Prefrontal_cortex_per_ages | GW08_GABAergic_neurons | 17199 | 0.00404 | 0.00506 | 0.21267 |
| MouseCellAtlas_all | Mammary_Gland.Myeloid_leukocyte | 14459 | 0.0422 | 0.0531 | 0.21309 |
| MouseCellAtlas_all | Fetal_Stomache.Endocrine_progenitor_cell | 14459 | 0.0719 | 0.0904 | 0.21317 |
| Linnarsson_GSE101601_Human_Temporal_cortex | Glut_4 | 17177 | 0.0982 | 0.124 | 0.21368 |
| TabulaMuris_FACS_all | Marrow.Fraction_A_pre.pro_B_cell | 13888 | 0.00589 | 0.00742 | 0.21371 |
| MouseCellAtlas_Mammary_Gland | Mammary_Gland_Pregnancy.B_cell_Ly6d_high | 14459 | 0.0552 | 0.0697 | 0.21385 |
| GSE82187_Mouse_Striatum | Oligo | 13103 | 0.0175 | 0.0221 | 0.21395 |
| MouseCellAtlas_Adult_all | Spleen.Dendritic_cell_S100a4_high | 14459 | 0.066 | 0.0834 | 0.21411 |
| MouseCellAtlas_Adult_all | Pancreas.B_cell | 14459 | 0.0434 | 0.0547 | 0.21414 |
| DropViz_all_level2 | TH.Oligodendrocyte_Trf_Il33.8_2 | 14805 | 0.00471 | 0.00595 | 0.2143 |
| MouseCellAtlas_Pancreas | Erythroblast_Igkc_high | 14459 | 0.0735 | 0.0929 | 0.21454 |
| TabulaMuris_FACS_Brain_Neurons | oligodendrocyte | 13888 | 0.0052 | 0.00658 | 0.21459 |
| DropViz_FC_level2 | Endothelial_Tip_Dcn_Col15a1.14_4 | 14805 | 0.0038 | 0.00481 | 0.21482 |
| Linnarsson_MouseBrainAtlas_level5 | HYPEP8 | 15140 | 0.0242 | 0.0306 | 0.21496 |
| DropViz_FC_level2 | Endothelial_Tip_Dcn_Dapl1.14_2 | 14805 | 0.00319 | 0.00407 | 0.21656 |
| MouseCellAtlas_Adult_all | Small_Intestine.Stromal_cell_Dcn_high | 14459 | 0.0309 | 0.0394 | 0.21671 |
| MouseCellAtlas_Adult_all | Mammary_Gland.Secretory_alveoli_cell_Cidea.Elovl5_high | 14459 | 0.0516 | 0.0659 | 0.21715 |
| MouseCellAtlas_Embryo_all | Fetal_Brain.Stromal_cell | 14459 | 0.0854 | 0.109 | 0.21735 |
| MouseCellAtlas_Lung | Plasmacytoid_dendritic_cell | 14459 | 0.0871 | 0.112 | 0.21806 |
| TabulaMuris_FACS_Lung | endothelial_cell | 13888 | 0.00713 | 0.00917 | 0.21855 |
| MouseCellAtlas_Adult_all | Small_Intestine.T_cell_Ms4a4b_high | 14459 | 0.0499 | 0.0643 | 0.21866 |
| Linnarsson_MouseBrainAtlas_level5 | MFOL2 | 15140 | 0.0188 | 0.0242 | 0.21873 |
| MouseCellAtlas_Fetal_Intestine | Stromal_cell_Upk3b_high | 14459 | 0.126 | 0.163 | 0.21968 |
| MouseCellAtlas_Fetal_Liver | B_cell | 14459 | 0.0607 | 0.0786 | 0.22007 |
| MouseCellAtlas_all | Bone_Marrow.CXCL12.abundant_reticular_cell | 14459 | 0.0499 | 0.0648 | 0.22046 |
| GSE93374_Mouse_Arc_ME_level2 | Oligodendro6 | 13325 | 0.0558 | 0.0725 | 0.221 |
| DropViz_all_level2 | PC.Macrophage_C1qb_Mrc1.11_2 | 14805 | 0.00313 | 0.00407 | 0.22105 |
| DropViz_all_level2 | FC.Endothelial_Tip_Dcn_Dapl1.14_2 | 14805 | 0.00319 | 0.00415 | 0.22124 |
| DropViz_all_level2 | SN.Oligodendrocyte_Tfr_Il33.10_4 | 14805 | 0.00444 | 0.00579 | 0.22136 |
| MouseCellAtlas_Adult_all | Liver.Erythroblast_Hbb.bs_high | 14459 | 0.0622 | 0.081 | 0.22137 |
| Linnarsson_GSE95752_Mouse_Dentate_gyrus | nIPC | 12517 | 0.0308 | 0.0402 | 0.22139 |
| Linnarsson_GSE60361_Mouse_Cortex_Hippocampus_level2 | Mgl1 | 14010 | 0.0231 | 0.0301 | 0.2216 |
| MouseCellAtlas_all | Mammary_Gland.NK_cell_Gzma_high | 14459 | 0.0689 | 0.0901 | 0.22224 |
| MouseCellAtlas_all | Small_Intestine.Stromal_cell_Dcn_high | 14459 | 0.0301 | 0.0393 | 0.22234 |
| DropViz_all_level2 | GP.Neuron_Gad1Gad2_Hpcal4.2_4 | 14805 | 0.00579 | 0.00758 | 0.22243 |
| MouseCellAtlas_Liver | T_cell_Trbc2_high | 14459 | 0.0917 | 0.12 | 0.22274 |
| DropViz_SN_level2 | Mural_Rgs5Acta2_Kcnj8.13_2 | 14805 | 0.00227 | 0.00298 | 0.22321 |
| TabulaMuris_droplet_all | Heart.erythrocyte | 13888 | 0.0246 | 0.0324 | 0.22329 |
| DropViz_all_level2 | CB.Microglia_C1qb_Mrc1-Tmem119.5_1 | 14805 | 0.00223 | 0.00293 | 0.2234 |
| MouseCellAtlas_all | Embryonic_Mesenchyme.Stromal_Cell_Agtr2_high | 14459 | 0.0811 | 0.107 | 0.22366 |
| TabulaMuris_FACS_all | Brain_Microglia.microglial_cell | 13888 | 0.00568 | 0.00748 | 0.22373 |
| MouseCellAtlas_all | Embryonic_Mesenchyme.Neuron_Igfbpl1_high | 14459 | 0.0729 | 0.0961 | 0.22402 |
| TabulaMuris_FACS_Lung | type_I_pneumocyte | 13888 | 0.00292 | 0.00385 | 0.22422 |
| MouseCellAtlas_Placenta | NK_cell | 14459 | 0.0526 | 0.0695 | 0.22474 |
| MouseCellAtlas_Pancreas | Endothelial_cell_Fabp4_high | 14459 | 0.0748 | 0.099 | 0.22482 |
| MouseCellAtlas_all | Pancreas.B_cell | 14459 | 0.041 | 0.0542 | 0.2249 |
| MouseCellAtlas_all | Placenta.Macrophage_Apoe_high | 14459 | 0.0602 | 0.0798 | 0.22532 |
| MouseCellAtlas_Bladder | Endothelial_cell_Ly6c1_high | 14459 | 0.0491 | 0.0652 | 0.22562 |
| DropViz_PC_level2 | Mural_Acta2Rgs5_Abcc9.13_5 | 14805 | 0.0022 | 0.00293 | 0.22588 |
| Linnarsson_GSE101601_Mouse_Somatosensory_cortex | Oligodendrocyte_precursor_cell | 14550 | 0.0461 | 0.0613 | 0.22597 |
| MouseCellAtlas_all | Uterus.Macrophage | 14459 | 0.0502 | 0.0668 | 0.22605 |
| DropViz_all_level2 | FC.Endothelial_Tip_Dcn_Col15a1.14_4 | 14805 | 0.00364 | 0.00485 | 0.22633 |
| MouseCellAtlas_Neonatal_Muscle | Skeletal_muscle_cell_Tnnc2_high | 14459 | 0.0269 | 0.0358 | 0.22657 |
| MouseCellAtlas_all | Spleen.Monocyte | 14459 | 0.0553 | 0.0738 | 0.22663 |
| MouseCellAtlas_Adult_all | Kidney.Stromal_cell_Ptgds_high | 14459 | 0.0555 | 0.0741 | 0.22686 |
| Linnarsson_GSE103840_Mouse_Dorsal_horn | Glut_Cck_Maf | 14550 | 0.0477 | 0.0638 | 0.22741 |
| DropViz_GP_level2 | Endothelial_Stalk_Flt1_Angpt2.7_6 | 14805 | 0.00354 | 0.00473 | 0.22753 |
| MouseCellAtlas_Neonatal_Skin | Smooth_muscle_cell_Acta2_high | 14459 | 0.0775 | 0.104 | 0.22759 |
| DropViz_GP_level2 | Mural_Rgs5Acta2_Lmcd1.8_3 | 14805 | 0.00236 | 0.00317 | 0.22777 |
| Linnarsson_MouseBrainAtlas_level5 | HBINH3 | 15140 | 0.0499 | 0.0669 | 0.22793 |
| MouseCellAtlas_Embryo_all | Embryonic_Mesenchyme.Stromal_Cell_Cldn11_high | 14459 | 0.0647 | 0.0872 | 0.22898 |
| GSE106678_Mouse_Cortex | Ex21 | 14573 | 0.103 | 0.138 | 0.22915 |
| Linnarsson_GSE103840_Mouse_Dorsal_horn | GABA_Npy_Qrfpr | 14550 | 0.0518 | 0.0699 | 0.22921 |
| MouseCellAtlas_Thymus | abT_cell | 14459 | 0.148 | 0.2 | 0.22934 |
| Linnarsson_GSE76381_Human_Midbrain | NbGaba | 14835 | 0.032 | 0.0431 | 0.22941 |
| MouseCellAtlas_Neonatal_Muscle | Mesenchymal_cell | 14459 | 0.101 | 0.136 | 0.22949 |
| MouseCellAtlas_Adult_all | Kidney.S3_proximal_tubule_cells | 14459 | 0.0326 | 0.044 | 0.22961 |
| Allen_Mouse_VISp_level2 | Micro_Ctss | 13868 | 0.0049 | 0.00662 | 0.22982 |
| MouseCellAtlas_all | Small_Intestine.T_cell_Ms4a4b_high | 14459 | 0.047 | 0.0636 | 0.2301 |
| Linnarsson_MouseBrainAtlas_level5 | VLMC1 | 15140 | 0.0342 | 0.0463 | 0.23019 |
| MouseCellAtlas_Embryo_all | Placenta.NK_cell | 14459 | 0.0434 | 0.0589 | 0.23026 |
| DropViz_all_level2 | PC.Mural_Acta2Rgs5_Abcc9.13_5 | 14805 | 0.00221 | 0.003 | 0.23083 |
| DropViz_ENT_level2 | Polydendrocyte_Tnr_Pdgfa.5_2 | 14805 | 0.00388 | 0.00528 | 0.23098 |
| MouseCellAtlas_Lung | Alveolar_macrophage_Pclaf_high | 14459 | 0.0541 | 0.0737 | 0.23143 |
| Linnarsson_GSE101601_Mouse_Somatosensory_cortex | Differentiation_committed_oligodendrocyte_precursors | 14550 | 0.0566 | 0.0771 | 0.23158 |
| GSE87544_Mouse_Hypothalamus | Glu13 | 13878 | 0.0317 | 0.0433 | 0.23208 |
| Allen_Mouse_VISp_level1 | NonNeu_Micro | 13868 | 0.00502 | 0.00686 | 0.2322 |
| DropViz_HC_level1 | Endothelial_tip | 14805 | 0.00443 | 0.00605 | 0.23237 |
| MouseCellAtlas_Adult_all | Stomach.Muscle_cell | 14459 | 0.0477 | 0.0653 | 0.23247 |
| MouseCellAtlas_Embryonic_Mesenchyme | Stromal_Cell_Agtr2_high | 14459 | 0.0991 | 0.136 | 0.23261 |
| MouseCellAtlas_all | Kidney.Stromal_cell_Ptgds_high | 14459 | 0.0539 | 0.074 | 0.23308 |
| DropViz_all_level2 | GP.Ependymal_Ccdc153.6_1 | 14805 | 0.00242 | 0.00332 | 0.23331 |
| MouseCellAtlas_Neonatal_all | Neonatal_Muscle.Smooth_muscle_cell_Mylk_high | 14459 | 0.0517 | 0.0712 | 0.23371 |
| MouseCellAtlas_all | Kidney.S3_proximal_tubule_cells | 14459 | 0.032 | 0.044 | 0.23379 |
| DropViz_all_level2 | PC.Neuron_Slc17a7_Bcl6-Ptgfr.3_7 | 14805 | 0.00251 | 0.00346 | 0.23386 |
| DropViz_all_level2 | HC.Polydendrocyte_Tnr_Pdgfa.9_1 | 14805 | 0.00396 | 0.00546 | 0.23401 |
| MouseCellAtlas_Testis | Spermatocyte_Cabs1_high | 14459 | 0.065 | 0.0896 | 0.23405 |
| DropViz_FC_level2 | Endothelial_Tip_Dcn_Inmt.14_5 | 14805 | 0.00223 | 0.00308 | 0.23439 |
| DropViz_SN_level2 | Oligodendrocyte_Tfr_Ctps.10_5 | 14805 | 0.0043 | 0.00594 | 0.23446 |
| DropViz_all_level2 | HC.Neuron_Slc17a7_Dcn-Camk2d.5_9 | 14805 | 0.00493 | 0.00682 | 0.23493 |
| MouseCellAtlas_Neonatal_Rib | Muscle_cell | 14459 | 0.0725 | 0.1 | 0.23523 |
| MouseCellAtlas_Adult_all | Bladder.Umbrella_cell | 14459 | 0.0154 | 0.0213 | 0.23548 |
| TabulaMuris_droplet_Spleen | B_cell | 13888 | 0.0667 | 0.0926 | 0.23579 |
| MouseCellAtlas_all | Neonatal_Rib.Muscle_cell_Actc1_high | 14459 | 0.0363 | 0.0504 | 0.23581 |
| Linnarsson_GSE104323_Mouse_Dentate_gyrus | VLMC | 14909 | 0.027 | 0.0375 | 0.23595 |
| DropViz_all_level2 | TH.Mural_Rgs5Acta2_Car4.5_2 | 14805 | 0.00353 | 0.00491 | 0.23598 |
| DropViz_HC_level2 | Neuron_Slc17a7_FIbcd1-Inhba.5_7 | 14805 | 0.00691 | 0.00962 | 0.23616 |
| Linnarsson_GSE76381_Human_Midbrain | NbML5 | 14835 | 0.048 | 0.0668 | 0.2362 |
| MouseCellAtlas_Lung | T_Cell_Cd8b1_high | 14459 | 0.0619 | 0.0862 | 0.2362 |
| MouseCellAtlas_all | Embryonic_Mesenchyme.Stromal_Cell_Cldn11_high | 14459 | 0.0598 | 0.0833 | 0.23653 |
| MouseCellAtlas_Embryo_all | Embryonic_Mesenchyme.Neuron_Isl1_high | 14459 | 0.0699 | 0.0975 | 0.23661 |
| Linnarsson_MouseBrainAtlas_level5 | CBINH1 | 15140 | 0.0464 | 0.0648 | 0.23693 |
| MouseCellAtlas_Neonatal_Heart | Dividing_cell | 14459 | 0.0891 | 0.124 | 0.23694 |
| DropViz_HC_level2 | Oligodendrocyte_Trf_Il33.8_3 | 14805 | 0.00398 | 0.00556 | 0.23709 |
| MouseCellAtlas_Adult_all | Mammary_Gland.Secretory_alveoli_cell_Wfdc3.Wap_high | 14459 | 0.0345 | 0.0482 | 0.23739 |
| DroNc_Human_Hippocampus | OPC | 16914 | 0.185 | 0.259 | 0.2375 |
| DropViz_all_level2 | SN.Mural_Rgs5Acta2_Cbr2.13_4 | 14805 | 0.00277 | 0.00389 | 0.23767 |
| MouseCellAtlas_Adult_all | Bladder.Vascular_endothelial_cell | 14459 | 0.0485 | 0.068 | 0.2381 |
| DropViz_all_level2 | FC.Endothelial_Tip_Dcn_Inmt.14_5 | 14805 | 0.00221 | 0.00312 | 0.23902 |
| MouseCellAtlas_Bone_Marrow | Bone_Marrow_c_kit.Basophil | 14459 | 0.0726 | 0.102 | 0.23921 |
| Allen_Mouse_VISp_level2 | Vip_Parm1 | 13868 | 0.00979 | 0.0138 | 0.23943 |
| MouseCellAtlas_Adult_all | Bone_Marrow.CXCL12.abundant_reticular_cell | 14459 | 0.0435 | 0.0614 | 0.2395 |
| MouseCellAtlas_Peripheral_Blood | NK_cell_Gzma_high | 14459 | 0.0859 | 0.122 | 0.2399 |
| MouseCellAtlas_all | Stomach.Muscle_cell | 14459 | 0.0459 | 0.065 | 0.2399 |
| MouseCellAtlas_Mammary_Gland | Mammary_Gland_Lactation.Secretory_alveoli_cell_Ehf_high | 14459 | 0.0636 | 0.0901 | 0.24004 |
| Linnarsson_GSE67602_Mouse_Skin_Epidermis | uHF_I | 14528 | 0.0612 | 0.0866 | 0.24006 |
| MouseCellAtlas_Neonatal_all | Neonatal_Rib.Oligodendrocyte | 14459 | 0.0432 | 0.0612 | 0.24009 |
| MouseCellAtlas_Adult_all | Peripheral_Blood.B_cell_Ly6d_high | 14459 | 0.0588 | 0.0833 | 0.24023 |
| MouseCellAtlas_all | Spleen.Dendritic_cell_S100a4_high | 14459 | 0.0565 | 0.0801 | 0.24026 |
| MouseCellAtlas_Adult_all | Peripheral_Blood.Macrophage_Flt.ps1_high | 14459 | 0.0464 | 0.0659 | 0.24059 |
| MouseCellAtlas_Prostate | T_cell | 14459 | 0.164 | 0.233 | 0.24115 |
| MouseCellAtlas_all | Fetal_Stomache.Erythroblast_Hbb.bs_high | 14459 | 0.087 | 0.124 | 0.24135 |
| MouseCellAtlas_Fetal_Stomache | Epithelial_cell | 14459 | 0.145 | 0.207 | 0.24184 |
| MouseCellAtlas_Bone_Marrow | Bone_Marrow_c_kit.B_cell | 14459 | 0.0711 | 0.102 | 0.242 |
| TabulaMuris_FACS_Brain_Microglia | microglial_cell | 13888 | 0.0125 | 0.0179 | 0.24229 |
| MouseCellAtlas_Neonatal_all | Neonatal_Muscle.T_cell | 14459 | 0.0475 | 0.068 | 0.24231 |
| MouseCellAtlas_all | Bladder.Umbrella_cell | 14459 | 0.0148 | 0.0213 | 0.24288 |
| GSE92332_Mouse_Epithelium_droplet | Paneth | 11993 | 0.0568 | 0.0815 | 0.24289 |
| DropViz_all_level2 | TH.Neuron_Slc17a6_Rora_C1ql2.2_5 | 14805 | 0.00436 | 0.00626 | 0.24328 |
| DropViz_TH_level2 | Astrocyte_Gja1_Vegfa.11_2 | 14805 | 0.00421 | 0.00605 | 0.24329 |
| MouseCellAtlas_Neonatal_Heart | Ventricle_cardiomyocyte_Kcnj8_high | 14459 | 0.0877 | 0.126 | 0.24361 |
| TabulaMuris_droplet_all | Bladder.basal_cell_of_urothelium | 13888 | 0.0153 | 0.0221 | 0.24378 |
| MouseCellAtlas_all | Mammary_Gland.Secretory_alveoli_cell_Cidea.Elovl5_high | 14459 | 0.0448 | 0.0645 | 0.24382 |
| MouseCellAtlas_Neonatal_all | Neonatal_Muscle.Dendritic_cell | 14459 | 0.0497 | 0.0716 | 0.24388 |
| DropViz_HC_level2 | Neurogenesis_Sox4_Ppp2r2b.13_6 | 14805 | 0.00317 | 0.00457 | 0.24392 |
| MouseCellAtlas_Kidney | B_cell | 14459 | 0.0377 | 0.0544 | 0.24398 |
| Linnarsson_GSE95315_Mouse_Dentate_gyrus | Neuroblast2 | 11815 | 0.0464 | 0.067 | 0.24403 |
| Allen_Mouse_VISp_level1 | NonNeu_Oligo | 13868 | 0.00531 | 0.00766 | 0.2441 |
| MouseCellAtlas_all | Ovary.Large_luteal_cell | 14459 | 0.0411 | 0.0593 | 0.24414 |
| TabulaMuris_FACS_Marrow | hematopoietic_stem_cell | 13888 | 0.00554 | 0.008 | 0.24418 |
| MouseCellAtlas_Mammary_Gland | Mammary_Gland_Lactation.Secretory_alveoli_cell_Thy1_high | 14459 | 0.0708 | 0.102 | 0.24431 |
| MouseCellAtlas_all | Neonatal_Skin.Adipocyte | 14459 | 0.0421 | 0.0608 | 0.24438 |
| MouseCellAtlas_Placenta | Endothelial_cell_Maged2_high | 14459 | 0.0629 | 0.0909 | 0.24445 |
| GSE93374_Mouse_Arc_ME_level1 | Fibroblast | 13325 | 0.0609 | 0.088 | 0.24454 |
| GSE87544_Mouse_Hypothalamus | GABA16 | 13878 | 0.0547 | 0.0792 | 0.24481 |
| Linnarsson_GSE104323_Mouse_Dentate_gyrus | Immature_GC | 14909 | 0.0437 | 0.0632 | 0.24491 |
| Linnarsson_MouseBrainAtlas_level5 | DGNBL2 | 15140 | 0.0283 | 0.0412 | 0.24615 |
| TabulaMuris_FACS_Spleen | myeloid_cell | 13888 | 0.0106 | 0.0155 | 0.24616 |
| MouseCellAtlas_all | Peripheral_Blood.B_cell_Ly6d_high | 14459 | 0.0573 | 0.0834 | 0.24626 |
| Linnarsson_GSE95315_Mouse_Dentate_gyrus | nIPC | 11815 | 0.0318 | 0.0464 | 0.24642 |
| Linnarsson_GSE74672_Mouse_Hypothalamus_level1 | oligos | 14550 | 0.0247 | 0.036 | 0.24658 |
| MouseCellAtlas_Adult_all | Peripheral_Blood.Macrophage_S100a4_high | 14459 | 0.0549 | 0.0801 | 0.24664 |
| MouseCellAtlas_all | Ovary.Stroma_cell_ | 14459 | 0.0693 | 0.101 | 0.24684 |
| TabulaMuris_droplet_Lung | dendritic_cell | 13888 | 0.0315 | 0.046 | 0.24685 |
| GSE104276_Human_Prefrontal_cortex_per_ages | GW26_Microglia | 17199 | 0.00556 | 0.00814 | 0.24719 |
| Linnarsson_GSE60361_Mouse_Cortex_Hippocampus_level1 | pyramidal_CA1 | 14010 | 0.0196 | 0.0288 | 0.24723 |
| MouseCellAtlas_all | Bladder.Vascular_endothelial_cell | 14459 | 0.0461 | 0.0677 | 0.24759 |
| MouseCellAtlas_Mammary_Gland | Mammary_Gland_Lactation.Natural_kill_cell | 14459 | 0.084 | 0.123 | 0.24781 |
| DropViz_STR_level2 | Mural_Rgs5Acta2_Kcnj8.8_4 | 14805 | 0.00251 | 0.00369 | 0.24786 |
| MouseCellAtlas_Adult_all | Peripheral_Blood.Macrophage_Ace_high | 14459 | 0.0558 | 0.082 | 0.24819 |
| TabulaMuris_FACS_all | Marrow.hematopoietic_stem_cell | 13888 | 0.00444 | 0.00656 | 0.24932 |
| Linnarsson_MouseBrainAtlas_level5 | SCINH8 | 15140 | 0.0326 | 0.0483 | 0.24937 |
| DropViz_CB_level1 | Microglia | 14805 | 0.00232 | 0.00343 | 0.24971 |
| DropViz_SN_level2 | Mural_Rgs5Acta2_Rgs5.13_1 | 14805 | 0.00306 | 0.00455 | 0.25047 |
| GSE99235_Mouse_Lung_Vascular | FB3 | 14559 | 0.00553 | 0.00822 | 0.2505 |
| DropViz_SN_level2 | Neuron_Th_Vcan.4_5 | 14805 | 0.00428 | 0.00638 | 0.25093 |
| MouseCellAtlas_Neonatal_Heart | Endothelial_cell_Eln_high | 14459 | 0.0439 | 0.0653 | 0.25095 |
| MouseCellAtlas_all | Fetal_Stomache.Progenitor_cell | 14459 | 0.0828 | 0.124 | 0.25135 |
| DropViz_all_level2 | PC.Neuron_Slc17a7_Bcl6-Pvalb.3_9 | 14805 | 0.00487 | 0.00727 | 0.25152 |
| MouseCellAtlas_Mammary_Gland | Mammary_Gland_Pregnancy.Luminal_cell | 14459 | 0.0136 | 0.0203 | 0.25161 |
| DroNc_Mouse_Hippocampus | exPFC8 | 12425 | 0.119 | 0.178 | 0.25212 |
| MouseCellAtlas_Neonatal_Calvaria | Muscle_cell_Myl9_high | 14459 | 0.0626 | 0.0939 | 0.25266 |
| MouseCellAtlas_Adult_all | Mammary_Gland.Luminal_cell_Krt19_high_ | 14459 | 0.0472 | 0.0709 | 0.25303 |
| DropViz_HC_level2 | Neuron_Slc17a7_Fibcd1-Nptx2.5_3 | 14805 | 0.0065 | 0.00982 | 0.25376 |
| MouseCellAtlas_Mammary_Gland | Mammary_Gland_Lactation.Secretory_alveoli_cell_Fth1.Cib1_high | 14459 | 0.0319 | 0.0481 | 0.25382 |
| MouseCellAtlas_Adult_all | Bone_Marrow.Macrophage_C1qc_high | 14459 | 0.0402 | 0.0607 | 0.25383 |
| MouseCellAtlas_Small_Intestine | T_cell_Ms4a4b_high | 14459 | 0.0454 | 0.0686 | 0.25399 |
| MouseCellAtlas_all | Muscle.Muscle_cell_Tnnc1_high | 14459 | 0.0197 | 0.0298 | 0.25406 |
| TabulaMuris_FACS_all | Kidney.leukocyte | 13888 | 0.00579 | 0.00876 | 0.25406 |
| DropViz_ENT_level2 | Oligodendrocyte_Trf_Il33.1_4 | 14805 | 0.00448 | 0.00677 | 0.25424 |
| MouseCellAtlas_all | Liver.Erythroblast_Hbb.bs_high | 14459 | 0.0513 | 0.0775 | 0.2543 |
| MouseCellAtlas_Neonatal_Calvaria | Endothelial_cell | 14459 | 0.0665 | 0.101 | 0.25453 |
| MouseCellAtlas_Stomach | Stomach_cell_Gkn2_high | 14459 | 0.0702 | 0.107 | 0.25534 |
| MouseCellAtlas_all | Mammary_Gland.Secretory_alveoli_cell_Wfdc3.Wap_high | 14459 | 0.0313 | 0.0476 | 0.25562 |
| Linnarsson_MouseBrainAtlas_level5 | HBINH6 | 15140 | 0.0194 | 0.0295 | 0.25591 |
| TabulaMuris_droplet_Bladder | leukocyte | 13888 | 0.0247 | 0.0377 | 0.25598 |
| DropViz_HC_level2 | Neurogenesis_Sox4_Cdc20.13_3 | 14805 | 0.00243 | 0.0037 | 0.256 |
| DropViz_PC_level2 | Neuron_Sc17a7_Sema3e-Onecut2.2_18 | 14805 | 0.00717 | 0.0109 | 0.25634 |
| Linnarsson_GSE75330_Mouse_Oligodendrocytes | OPC | 14541 | 0.0314 | 0.048 | 0.25638 |
| DropViz_FC_level2 | Neuron_Slc17a7_Parm1_Lsamp.7_3 | 14805 | 0.00637 | 0.00973 | 0.25646 |
| MouseCellAtlas_Adult_all | Mammary_Gland.Dendritic_cell_Ccl22_high | 14459 | 0.0409 | 0.0626 | 0.25664 |
| TabulaMuris_FACS_Spleen | B_cell | 13888 | 0.0124 | 0.019 | 0.25674 |
| MouseCellAtlas_Adult_all | Testis.Spermatids_Hmgb4_high | 14459 | 0.0216 | 0.0331 | 0.2568 |
| Linnarsson_GSE76381_Human_Midbrain | NbML1 | 14835 | 0.0448 | 0.0687 | 0.25714 |
| GSE99235_Mouse_Lung_Vascular | CP2 | 14559 | 0.0075 | 0.0115 | 0.2575 |
| MouseCellAtlas_all | Mammary_Gland.Luminal_cell_Krt19_high_ | 14459 | 0.0466 | 0.0716 | 0.25766 |
| MouseCellAtlas_Adult_all | Muscle.Muscle_cell_Tnnc1_high | 14459 | 0.0193 | 0.0297 | 0.25784 |
| MouseCellAtlas_Neonatal_Muscle | Macrophage_Pf4_high | 14459 | 0.05 | 0.077 | 0.25797 |
| MouseCellAtlas_Adult_all | Lung.Conventional_dendritic_cell_H2.M2_high | 14459 | 0.032 | 0.0493 | 0.25816 |
| GSE93374_Mouse_Arc_ME_level2 | Fibroblasts3 | 13325 | 0.0562 | 0.0868 | 0.25857 |
| Linnarsson_GSE60361_Mouse_Cortex_Hippocampus_level2 | Int15 | 14010 | 0.024 | 0.0371 | 0.25897 |
| Allen_Mouse_ALM_level2 | Vip_Prss12_2 | 14744 | 0.0064 | 0.00992 | 0.2596 |
| MouseCellAtlas_Adult_all | Ovary.Large_luteal_cell | 14459 | 0.0361 | 0.0564 | 0.26066 |
| DropViz_GP_level2 | Oligodendrocyte_Trf_Ctps.10_4 | 14805 | 0.00325 | 0.00508 | 0.2613 |
| MouseCellAtlas_all | Testis.Spermatids_Hmgb4_high | 14459 | 0.0211 | 0.0331 | 0.26187 |
| MouseCellAtlas_Embryo_all | Fetal_Brain.Dopaminergic_neurons_ | 14459 | 0.0512 | 0.0802 | 0.2619 |
| MouseCellAtlas_Neonatal_all | Neonatal_Rib.Muscle_cell_Actc1_high | 14459 | 0.0364 | 0.0571 | 0.26202 |
| MouseCellAtlas_Fetal_Intestine | Enterocyte_progenitor | 14459 | 0.166 | 0.261 | 0.26233 |
| DropViz_all_level2 | GP.Polydendrocyte_Tnr_Opalin.4_3 | 14805 | 0.00274 | 0.0043 | 0.26236 |
| Allen_Mouse_ALM_level2 | L5_ALM_Oprk1 | 14744 | 0.00441 | 0.00694 | 0.26265 |
| MouseCellAtlas_Adult_all | Mammary_Gland.Macrophage_C1qc_high | 14459 | 0.0431 | 0.0679 | 0.26266 |
| MouseCellAtlas_Muscle | Muscle_progenitor_cell | 14459 | 0.0469 | 0.074 | 0.26287 |
| MouseCellAtlas_Peripheral_Blood | T_cell_Gm14303_high | 14459 | 0.0525 | 0.0831 | 0.26351 |
| MouseCellAtlas_Peripheral_Blood | Macrophage_Pf4_high | 14459 | 0.0333 | 0.0527 | 0.26357 |
| DropViz_all_level2 | GP.Macrophage_C1qb_Mrc1.11_2 | 14805 | 0.0024 | 0.0038 | 0.26384 |
| DropViz_GP_level2 | Polydendrocyte_Tnr_Pdgfa-Tmem2.4_2 | 14805 | 0.00319 | 0.00505 | 0.26386 |
| DropViz_GP_level2 | Neuron_Gad1Gad2_Lamp5-Cplx3.2_22 | 14805 | 0.0023 | 0.00365 | 0.26437 |
| DropViz_all_level2 | HC.Neuron_Slc17a7_Rgs14.6_4 | 14805 | 0.00424 | 0.00673 | 0.2645 |
| Linnarsson_GSE101601_Mouse_Somatosensory_cortex | Pyramidal_cells_layer_4_Plcxd2 | 14550 | 0.0243 | 0.0386 | 0.26457 |
| MouseCellAtlas_Placenta | Stromal_cell | 14459 | 0.0561 | 0.0892 | 0.26457 |
| Linnarsson_MouseBrainAtlas_level5 | HBNOR | 15140 | 0.0246 | 0.0391 | 0.26471 |
| GSE106678_Mouse_Cortex | Ex14 | 14573 | 0.0709 | 0.113 | 0.26474 |
| MouseCellAtlas_Fetal_Liver | Dendritic_cell | 14459 | 0.0796 | 0.127 | 0.26499 |
| GSE92332_Mouse_Epithelium_droplet | Enterocyte_Mature_Distal | 11993 | 0.0204 | 0.0326 | 0.26542 |
| Linnarsson_GSE74672_Mouse_Hypothalamus_Neurons_level2 | Vglut2_5_Myt1Lhx9 | 14550 | 0.0147 | 0.0236 | 0.26638 |
| Linnarsson_GSE95752_Mouse_Dentate_gyrus | Cajal_Retzius | 12517 | 0.0347 | 0.0556 | 0.26638 |
| DropViz_all_level2 | STR.Macrophage_C1qb_Mrc1.6_1 | 14805 | 0.00182 | 0.00292 | 0.2671 |
| GSE87544_Mouse_Hypothalamus | MO | 13878 | 0.02 | 0.0322 | 0.26754 |
| MouseCellAtlas_Peripheral_Blood | Neutrophil_Il1b_high | 14459 | 0.054 | 0.0873 | 0.26799 |
| MouseCellAtlas_Embryonic_Mesenchyme | Neuron_Isl1_high | 14459 | 0.069 | 0.111 | 0.26804 |
| MouseCellAtlas_all | Peripheral_Blood.Macrophage_Flt.ps1_high | 14459 | 0.0391 | 0.0634 | 0.26847 |
| DropViz_FC_level2 | Neuron_Slc17a7_Parm1_Cadm2.7_5 | 14805 | 0.00377 | 0.00612 | 0.26876 |
| MouseCellAtlas_all | Mammary_Gland.Dendritic_cell_Ccl22_high | 14459 | 0.038 | 0.0618 | 0.26927 |
| DropViz_all_level2 | TH.Astrocyte_Gja1_Myoc.11_1 | 14805 | 0.00248 | 0.00403 | 0.26932 |
| MouseCellAtlas_Neonatal_all | Neonatal_Muscle.Adipocyte. | 14459 | 0.0332 | 0.0541 | 0.26985 |
| Linnarsson_GSE74672_Mouse_Hypothalamus_Neurons_level2 | Dopamine_1 | 14550 | 0.0295 | 0.0482 | 0.2703 |
| MouseCellAtlas_Embryonic_Mesenchyme | Stromal_Cell_Cldn11_high | 14459 | 0.0554 | 0.0907 | 0.27079 |
| GSE93374_Mouse_Arc_ME_neurons | Kiss1_Tac2 | 13325 | 0.187 | 0.307 | 0.27105 |
| DropViz_all_level2 | PC.Neuron_Sc17a7_Sema3e-Nnat.2_17 | 14805 | 0.00505 | 0.00829 | 0.27119 |
| MouseCellAtlas_all | Neonatal_Rib.Neuron_Mpz_high | 14459 | 0.046 | 0.0756 | 0.27138 |
| MouseCellAtlas_all | Muscle.Muscle_progenitor_cell | 14459 | 0.0503 | 0.0826 | 0.27139 |
| MouseCellAtlas_Embryo_all | Fetal_Intestine.Enterocyte_progenitor | 14459 | 0.108 | 0.178 | 0.27159 |
| Linnarsson_MouseBrainAtlas_level6_rank3 | Immature_neural | 15140 | 0.0329 | 0.0543 | 0.27243 |
| MouseCellAtlas_all | Fetal_Lung.Stromal_cell_Ptn_high | 14459 | 0.065 | 0.107 | 0.27245 |
| Linnarsson_GSE76381_Mouse_Midbrain | DA1 | 14550 | 0.0347 | 0.0572 | 0.27248 |
| MouseCellAtlas_Small_Intestine | Stromal_cell_Dcn_high | 14459 | 0.0235 | 0.0388 | 0.27274 |
| MouseCellAtlas_all | Placenta.Monocyte | 14459 | 0.0463 | 0.0766 | 0.27292 |
| MouseCellAtlas_Adult_all | Lung.Interstitial_macrophage | 14459 | 0.0516 | 0.0855 | 0.27295 |
| MouseCellAtlas_Fetal_Brain | Granule_neurons | 14459 | 0.105 | 0.174 | 0.2731 |
| MouseCellAtlas_all | Peripheral_Blood.Macrophage_S100a4_high | 14459 | 0.0465 | 0.0771 | 0.2731 |
| MouseCellAtlas_Neonatal_Muscle | Glial_cell | 14459 | 0.0621 | 0.103 | 0.27323 |
| MouseCellAtlas_Mammary_Gland | Mammary_Gland_Pregnancy.T_cell | 14459 | 0.0401 | 0.0667 | 0.27356 |
| Linnarsson_GSE101601_Human_Temporal_cortex | GABA_2 | 17177 | 0.0889 | 0.148 | 0.2736 |
| GSE104276_Human_Prefrontal_cortex_per_ages | GW09_Neurons | 17199 | 0.00618 | 0.0103 | 0.27371 |
| MouseCellAtlas_all | Fetal_Intestine.Stromal_cell_Adamdec1_high | 14459 | 0.0698 | 0.117 | 0.27473 |
| DropViz_TH_level2 | Polydendrocyte_Tnr_Ctps.12_2 | 14805 | 0.00296 | 0.00495 | 0.27494 |
| TabulaMuris_FACS_Pancreas | pancreatic_stellate_cell | 13888 | 0.00421 | 0.00705 | 0.27546 |
| MouseCellAtlas_all | Lung.Conventional_dendritic_cell_H2.M2_high | 14459 | 0.0288 | 0.0484 | 0.27563 |
| DropViz_SN_level1 | Macrophage | 14805 | 0.00277 | 0.00466 | 0.27629 |
| MouseCellAtlas_all | Fetal_Intestine.Stromal_cell_Upk3b_high | 14459 | 0.0666 | 0.112 | 0.27629 |
| MouseCellAtlas_Embryo_all | Fetal_Brain.Erythroblast_Hbb.bh1_high | 14459 | 0.0364 | 0.0613 | 0.27642 |
| MouseCellAtlas_Brain | Neuron | 14459 | 0.0606 | 0.102 | 0.27659 |
| MouseCellAtlas_all | Peripheral_Blood.Macrophage_Ace_high | 14459 | 0.0461 | 0.0785 | 0.27853 |
| MouseCellAtlas_Adult_all | Small_Intestine.Epithelium_of_small_intestinal_villi_S100g_high | 14459 | 0.0288 | 0.0492 | 0.27915 |
| Allen_Mouse_ALM_level2 | L5_ALM_Arhgap25_2 | 14744 | 0.006 | 0.0103 | 0.27951 |
| MouseCellAtlas_all | Bone_Marrow.Macrophage_C1qc_high | 14459 | 0.0344 | 0.0589 | 0.27982 |
| MouseCellAtlas_Adult_all | Small_Intestine.Macrophage_Apoe_high | 14459 | 0.037 | 0.0634 | 0.27987 |
| MouseCellAtlas_Mammary_Gland | Mammary_Gland_Involution.Macrophage_Pf4_high | 14459 | 0.036 | 0.0617 | 0.27995 |
| Linnarsson_MouseBrainAtlas_level5 | PSNP3 | 15140 | 0.00896 | 0.0154 | 0.28 |
| Linnarsson_GSE74672_Mouse_Hypothalamus_Neurons_level2 | Avp_2_high | 14550 | 0.0169 | 0.0291 | 0.28015 |
| GSE104276_Human_Prefrontal_cortex_per_ages | GW23_GABAergic_neurons | 17199 | 0.00746 | 0.0128 | 0.28062 |
| Linnarsson_GSE74672_Mouse_Hypothalamus_Neurons_level2 | GABA_8 | 14550 | 0.0272 | 0.0469 | 0.2809 |
| MouseCellAtlas_Mammary_Gland | Mammary_Gland_Lactation.Secretory_alveoli_cell_Igkv1.110.P4hb_high | 14459 | 0.0507 | 0.0875 | 0.28101 |
| MouseCellAtlas_Mammary_Gland | Mammary_Gland_Lactation.Secretory_alveoli_cell_Crip1_high | 14459 | 0.0393 | 0.0677 | 0.28107 |
| MouseCellAtlas_Embryo_all | Fetal_Brain.Granule_neurons | 14459 | 0.0559 | 0.0964 | 0.28118 |
| GSE93374_Mouse_Arc_ME_level2 | Oligodendro5 | 13325 | 0.0241 | 0.0416 | 0.28121 |
| TabulaMuris_FACS_all | Lung.type_II_pneumocyte | 13888 | 0.00454 | 0.00784 | 0.28133 |
| MouseCellAtlas_all | Fetal_Brain.Pyramidal_neuron_cell | 14459 | 0.0483 | 0.0834 | 0.28134 |
| GSE106678_Mouse_Cortex | Ex6 | 14573 | 0.0775 | 0.134 | 0.2814 |
| Linnarsson_MouseBrainAtlas_level5 | MBDOP2 | 15140 | 0.0239 | 0.0414 | 0.28158 |
| TabulaMuris_FACS_Trachea | endothelial_cell | 13888 | 0.0058 | 0.01 | 0.28181 |
| MouseCellAtlas_Fetal_Brain | Schwann_cell | 14459 | 0.0943 | 0.163 | 0.28183 |
| MouseCellAtlas_Spleen | Dendritic_cell_Siglech_high | 14459 | 0.0498 | 0.0863 | 0.28184 |
| DropViz_CB_level2 | Endothelial_Tip_Dcn_Nnat.11_1 | 14805 | 0.00217 | 0.00377 | 0.2821 |
| Linnarsson_MouseBrainAtlas_level5 | TEGLU18 | 15140 | 0.0235 | 0.0408 | 0.28212 |
| DropViz_all_level2 | HC.Neuron_Slc17a7_Pvrl3-Fos.6_8 | 14805 | 0.0039 | 0.00677 | 0.28223 |
| MouseCellAtlas_Bone_Marrow | Bone_Marrow.Macrophage_S100a4_high | 14459 | 0.0241 | 0.0418 | 0.28237 |
| MouseCellAtlas_Adult_all | Thymus.T_cell_Ms4a4b_high | 14459 | 0.0499 | 0.0867 | 0.28244 |
| GSE87544_Mouse_Hypothalamus | Macro | 13878 | 0.0171 | 0.0297 | 0.28254 |
| Linnarsson_GSE59739_Mouse_Dorsal_root_ganglion_level3 | Non_peptidergic_nocieptor3 | 13848 | 0.00507 | 0.00883 | 0.28292 |
| DropViz_FC_level2 | Neuron_Slc17a7_Parm1_Ndnf.7_1 | 14805 | 0.00508 | 0.00887 | 0.28328 |
| Linnarsson_MouseBrainAtlas_level5 | MOL2 | 15140 | 0.016 | 0.028 | 0.28362 |
| Linnarsson_MouseBrainAtlas_level5 | HBSER2 | 15140 | 0.0131 | 0.023 | 0.28382 |
| DropViz_GP_level2 | Microglia_C1qb_Tmem119.11_1 | 14805 | 0.00232 | 0.00405 | 0.28398 |
| MouseCellAtlas_Mammary_Gland | Mammary_Gland_Lactation.Muscel_cell | 14459 | 0.049 | 0.0858 | 0.28402 |
| TabulaMuris_FACS_all | Brain_Neurons.neuronal_stem_cell | 13888 | 0.00312 | 0.00548 | 0.28464 |
| DropViz_all_level2 | SN.Neuron_Th_Cyp26b1.4_3 | 14805 | 0.00345 | 0.00606 | 0.28471 |
| DropViz_GP_level2 | Neuron_Slc17a7_Neurod2-C1ql3.2_12 | 14805 | 0.0029 | 0.00511 | 0.28485 |
| MouseCellAtlas_all | Thymus.T_cell_Ms4a4b_high | 14459 | 0.0498 | 0.0877 | 0.28506 |
| MouseCellAtlas_all | Fetal_Brain.Neural_progenitor_cell | 14459 | 0.0469 | 0.0828 | 0.28545 |
| GSE67835_Human_Cortex_woFetal | microglia | 16585 | 0.00437 | 0.00773 | 0.28597 |
| MouseCellAtlas_all | Mammary_Gland.B_cell_Cd79a_high | 14459 | 0.0423 | 0.0749 | 0.28604 |
| MouseCellAtlas_Neonatal_all | Neonatal_Skin.Lymphatic_vessel_endothelial_cell | 14459 | 0.0351 | 0.0621 | 0.28605 |
| Linnarsson_MouseBrainAtlas_level5 | HBINH9 | 15140 | 0.0285 | 0.0507 | 0.28679 |
| GSE87544_Mouse_Hypothalamus | Ependy | 13878 | 0.0159 | 0.0282 | 0.28699 |
| Linnarsson_MouseBrainAtlas_level6_rank4 | Gsch | 15140 | 0.0152 | 0.027 | 0.28737 |
| GSE104276_Human_Prefrontal_cortex_all_ages | Neurons | 17199 | 0.00947 | 0.0169 | 0.28742 |
| Linnarsson_GSE95752_Mouse_Dentate_gyrus | GABA_Cnr1 | 12517 | 0.0251 | 0.0448 | 0.28759 |
| MouseCellAtlas_Adult_all | Mammary_Gland.B_cell_Cd79a_high | 14459 | 0.0411 | 0.0735 | 0.28802 |
| Linnarsson_GSE60361_Mouse_Cortex_Hippocampus_level2 | Oligo2 | 14010 | 0.0129 | 0.0231 | 0.28804 |
| DropViz_PC_level2 | Oligodendrocyte_Tfr_Tmem2.9_2 | 14805 | 0.002 | 0.00358 | 0.28817 |
| GSE92332_Mouse_Epithelium_droplet | Stem | 11993 | 0.039 | 0.0698 | 0.28831 |
| Allen_Mouse_VISp_level2 | L2_3_Ptgs2 | 13868 | 0.00776 | 0.0139 | 0.28858 |
| DropViz_all_level2 | PC.Oligodendrocyte_Tfr_Tmem2.9_2 | 14805 | 0.00212 | 0.00381 | 0.28891 |
| DropViz_HC_level2 | Gad1Gad2_Htr3a_Nnat.1_16 | 14805 | 0.0034 | 0.00611 | 0.2891 |
| MouseCellAtlas_Neonatal_all | Neonatal_Skin.Keratinocyte | 14459 | 0.0473 | 0.0852 | 0.28944 |
| MouseCellAtlas_all | Fetal_Brain.Stromal_cell | 14459 | 0.0496 | 0.0895 | 0.28976 |
| MouseCellAtlas_all | Placenta.Dendritic_cell | 14459 | 0.0503 | 0.0908 | 0.28976 |
| MouseCellAtlas_Adult_all | Bone_Marrow.B_cell_Igkc_high | 14459 | 0.0309 | 0.0558 | 0.28992 |
| MouseCellAtlas_Fetal_Brain | Neuron_Kpna2_high | 14459 | 0.073 | 0.132 | 0.29037 |
| MouseCellAtlas_Uterus | Stromal_cell_Cxcl14_high | 14459 | 0.0701 | 0.127 | 0.29113 |
| MouseCellAtlas_Embryo_all | Embryonic_Mesenchyme.Erythroid_Cell | 14459 | 0.0368 | 0.067 | 0.29119 |
| MouseCellAtlas_Mammary_Gland | Mammary_Gland_Lactation.Secretory_alveoli_cell_Fth1.Wfdc3_high | 14459 | 0.0448 | 0.0815 | 0.2912 |
| Linnarsson_MouseBrainAtlas_level5 | CBNBL2 | 15140 | 0.0445 | 0.081 | 0.29135 |
| GSE93374_Mouse_Arc_ME_level1 | Oligodend2 | 13325 | 0.0193 | 0.0353 | 0.29203 |
| TabulaMuris_droplet_all | Liver.endothelial_cell | 13888 | 0.0204 | 0.0372 | 0.29203 |
| DropViz_all_level2 | SN.Astrocyte_Gja1_Myoc.7_1 | 14805 | 0.00247 | 0.00452 | 0.29234 |
| DropViz_HC_level2 | Neuron_Slc17a7_Pvrl3-Nos1.6_1 | 14805 | 0.00531 | 0.00973 | 0.29255 |
| MouseCellAtlas_Adult_all | Kidney.Epithelial_cell_Cryab_high | 14459 | 0.0338 | 0.062 | 0.29286 |
| DropViz_all_level2 | ENT.Mural_Rgs5Acta2_Kcnj8.3_1 | 14805 | 0.00176 | 0.00322 | 0.29287 |
| TabulaMuris_FACS_all | Heart.endocardial_cell | 13888 | 0.00466 | 0.00856 | 0.293 |
| GSE106678_Mouse_Cortex | Ex23 | 14573 | 0.0752 | 0.138 | 0.29321 |
| DropViz_PC_level2 | Neuron_Slc17a7_Bcl6-Tcerg1l.3_4 | 14805 | 0.0059 | 0.0108 | 0.29327 |
| GSE93374_Mouse_Arc_ME_level2 | Mural_Cells2 | 13325 | 0.035 | 0.0644 | 0.29329 |
| MouseCellAtlas_Fetal_Stomache | Erythroblast_Hba.x_HIGH | 14459 | 0.0503 | 0.0926 | 0.29346 |
| Linnarsson_GSE67602_Mouse_Skin_Epidermis | IFE_B | 14528 | 0.0312 | 0.0573 | 0.29348 |
| DropViz_all_level2 | HC.Neuron_Slc17a7_Fibcd1-Grm5.5_5 | 14805 | 0.00281 | 0.00518 | 0.29382 |
| DropViz_all_level2 | PC.Neuron_Slc17a7-Slc17a6_Tshz2-Rasd1.6_3 | 14805 | 0.0037 | 0.00683 | 0.29398 |
| Linnarsson_MouseBrainAtlas_level6_rank4 | Goli | 15140 | 0.0159 | 0.0293 | 0.29401 |
| MouseCellAtlas_Placenta | Macrophage_Apoe_high | 14459 | 0.0455 | 0.0842 | 0.2943 |
| MouseCellAtlas_Adult_all | Kidney.Fenestrated_endothelial_cell_Plvap_high | 14459 | 0.0375 | 0.0694 | 0.29438 |
| MouseCellAtlas_all | Mammary_Gland.Macrophage_C1qc_high | 14459 | 0.0351 | 0.0649 | 0.29439 |
| MouseCellAtlas_Muscle | Muscle_cell_Tnnc2_high | 14459 | 0.0184 | 0.0341 | 0.29457 |
| DropViz_SN_level2 | Neuron_Th_Cbln1.4_2 | 14805 | 0.00273 | 0.00506 | 0.29461 |
| Linnarsson_MouseBrainAtlas_level5 | HBGLU10 | 15140 | 0.0311 | 0.0578 | 0.29512 |
| MouseCellAtlas_Mammary_Gland | Mammary_Gland_Involution.NK_cell_Cd8b1_high | 14459 | 0.0284 | 0.0527 | 0.29522 |
| MouseCellAtlas_all | Bone_Marrow.Endothelial_cells | 14459 | 0.0427 | 0.0793 | 0.29525 |
| Linnarsson_GSE95752_Mouse_Dentate_gyrus | VLMC | 12517 | 0.0229 | 0.0428 | 0.29595 |
| MouseCellAtlas_Spleen | Neutrophil | 14459 | 0.0259 | 0.0483 | 0.2961 |
| MouseCellAtlas_Muscle | B_cell_Jchain_high | 14459 | 0.0376 | 0.0702 | 0.29625 |
| DropViz_all_level2 | GP.Oligodendrocyte_Trf_Il33.10_5 | 14805 | 0.00314 | 0.00586 | 0.29634 |
| MouseCellAtlas_all | Kidney.Epithelial_cell_Cryab_high | 14459 | 0.0332 | 0.0621 | 0.29654 |
| GSE93374_Mouse_Arc_ME_level2 | b2_tanycytes2 | 13325 | 0.0552 | 0.104 | 0.29696 |
| Linnarsson_MouseBrainAtlas_level5 | OBNBL4 | 15140 | 0.0435 | 0.0816 | 0.29705 |
| DropViz_TH_level2 | Oligodendrocyte_Trf_Ndrg2.8_4 | 14805 | 0.00324 | 0.00608 | 0.29712 |
| MouseCellAtlas_all | Small_Intestine.Epithelium_of_small_intestinal_villi_S100g_high | 14459 | 0.0256 | 0.0485 | 0.29849 |
| Linnarsson_MouseBrainAtlas_level5 | TEGLU17 | 15140 | 0.0207 | 0.0392 | 0.29874 |
| MouseCellAtlas_Muscle | T_cell | 14459 | 0.0279 | 0.0529 | 0.29925 |
| TabulaMuris_FACS_all | Skin.keratinocyte_stem_cell | 13888 | 0.00441 | 0.00839 | 0.29971 |
| MouseCellAtlas_Adult_all | Lung.Alveolar_bipotent_progenitor | 14459 | 0.027 | 0.0515 | 0.29978 |
| MouseCellAtlas_Embryo_all | Fetal_Intestine.Enteroendocrine | 14459 | 0.0425 | 0.081 | 0.29985 |
| DroNc_Mouse_Hippocampus | MG | 12425 | 0.111 | 0.212 | 0.30008 |
| MouseCellAtlas_Small_Intestine | Macrophage_Apoe_high | 14459 | 0.0347 | 0.0662 | 0.30021 |
| DropViz_GP_level2 | Ependymal_Ccdc153.6_1 | 14805 | 0.00175 | 0.00335 | 0.30062 |
| MouseCellAtlas_all | Bone_Marrow.B_cell_Igkc_high | 14459 | 0.0289 | 0.0553 | 0.30078 |
| DropViz_HC_level2 | Neuron_Slc17a7_Nxph3-Ajap1.3_11 | 14805 | 0.00385 | 0.00739 | 0.30137 |
| MouseCellAtlas_Adult_all | Muscle.Muscle_progenitor_cell | 14459 | 0.0398 | 0.0766 | 0.30159 |
| DropViz_all_level1 | FC.Endothelial_tip | 14805 | 0.00346 | 0.00667 | 0.30221 |
| MouseCellAtlas_Adult_all | Ovary.Stroma_cell_ | 14459 | 0.0444 | 0.0859 | 0.30279 |
| MouseCellAtlas_Embryo_all | Fetal_Brain.Ependymal_cell | 14459 | 0.0433 | 0.084 | 0.30303 |
| MouseCellAtlas_all | Lung.Interstitial_macrophage | 14459 | 0.0421 | 0.0817 | 0.30335 |
| DropViz_all_level2 | CB.Endothelial_Tip_Dcn_Nnat.11_1 | 14805 | 0.00172 | 0.00335 | 0.30375 |
| MouseCellAtlas_all | Kidney.Fenestrated_endothelial_cell_Plvap_high | 14459 | 0.0353 | 0.0689 | 0.30401 |
| Linnarsson_MouseBrainAtlas_level6_rank3 | Oligodendrocytes | 15140 | 0.015 | 0.0293 | 0.3042 |
| MouseCellAtlas_Adult_all | Kidney.T_cell | 14459 | 0.047 | 0.092 | 0.30462 |
| DropViz_all_level2 | GP.Astrocyte_Gja1_Gfap.5_1 | 14805 | 0.00291 | 0.00569 | 0.30472 |
| GSE82187_Mouse_Striatum | Microglia | 13103 | 0.0136 | 0.0267 | 0.30475 |
| MouseCellAtlas_Neonatal_all | Neonatal_Rib.Neuron_Mpz_high | 14459 | 0.0442 | 0.0867 | 0.30532 |
| Linnarsson_MouseBrainAtlas_level5 | TEGLU21 | 15140 | 0.0159 | 0.0312 | 0.30538 |
| DropViz_FC_level2 | Neuron_Slc17a7_Nptxr-Deptor.6_4 | 14805 | 0.00534 | 0.0105 | 0.30558 |
| Linnarsson_GSE67602_Mouse_Skin_Epidermis | TC | 14528 | 0.0216 | 0.0425 | 0.30611 |
| GSE93374_Mouse_Arc_ME_level2 | PVMs | 13325 | 0.0291 | 0.0574 | 0.30648 |
| MouseCellAtlas_all | Pancreas.Endocrine_cell | 14459 | 0.0353 | 0.0698 | 0.30661 |
| GSE81547_Human_Pancreas | PP | 16741 | 0.00637 | 0.0127 | 0.3076 |
| MouseCellAtlas_Embryo_all | Fetal_Brain.Neuron_Kpna2_high | 14459 | 0.043 | 0.0856 | 0.30761 |
| MouseCellAtlas_Embryo_all | Placenta.Macrophage_Apoe_high | 14459 | 0.0355 | 0.0708 | 0.30799 |
| TabulaMuris_FACS_Thymus | mesenchymal_stem_cell | 13888 | 0.00911 | 0.0182 | 0.3082 |
| DropViz_STR_level2 | Neurogenesis_Sox4_Stmn2-Csrp2.2_4 | 14805 | 0.00202 | 0.00404 | 0.30829 |
| DropViz_all_level2 | HC.Oligodendrocyte_Trf_Il33.8_3 | 14805 | 0.00308 | 0.00615 | 0.30863 |
| DropViz_all_level2 | STR.Oligodendrocyte_Trf_Ctps.3_3 | 14805 | 0.00254 | 0.00508 | 0.30863 |
| Linnarsson_MouseBrainAtlas_level5 | HBGLU4 | 15140 | 0.0103 | 0.0207 | 0.30868 |
| MouseCellAtlas_all | Kidney.T_cell | 14459 | 0.0464 | 0.0928 | 0.30873 |
| TabulaMuris_FACS_all | Pancreas.pancreatic_ductal_cell | 13888 | 0.00375 | 0.00751 | 0.30883 |
| Linnarsson_MouseBrainAtlas_level5 | PSNF1 | 15140 | 0.0103 | 0.0207 | 0.30889 |
| DropViz_all_level2 | SN.Neuron_Th_Cadm2.4_6 | 14805 | 0.00155 | 0.00311 | 0.3091 |
| DroNc_Mouse_Hippocampus | exPFC6 | 12425 | 0.109 | 0.218 | 0.3096 |
| GSE87544_Mouse_Hypothalamus | Glu6 | 13878 | 0.0232 | 0.0469 | 0.31014 |
| TabulaMuris_droplet_all | Lung.dendritic_cell | 13888 | 0.0194 | 0.0393 | 0.31029 |
| GSE98816_Mouse_Brain_Vascular | EC3 | 14130 | 0.00557 | 0.0113 | 0.31048 |
| MouseCellAtlas_all | Lung.Alveolar_bipotent_progenitor | 14459 | 0.0252 | 0.0511 | 0.31055 |
| DropViz_CB_level2 | Polydendrocyte_Tnr_Tnr.6_3 | 14805 | 0.00159 | 0.00322 | 0.31061 |
| DropViz_all_level2 | SN.Oligodendrocyte_Tfr_Slco3a1.10_1 | 14805 | 0.00281 | 0.00568 | 0.31066 |
| MouseCellAtlas_Adult_all | Bladder.NK_cell | 14459 | 0.0369 | 0.0751 | 0.31136 |
| Linnarsson_GSE74672_Mouse_Hypothalamus_Neurons_level2 | Gadlow_Gnrh | 14550 | 0.0207 | 0.0421 | 0.31146 |
| MouseCellAtlas_all | Small_Intestine.Macrophage_Apoe_high | 14459 | 0.03 | 0.0611 | 0.31152 |
| MouseCellAtlas_all | Embryonic_Mesenchyme.Neuron_Isl1_high | 14459 | 0.0389 | 0.0796 | 0.31245 |
| MouseCellAtlas_Mammary_Gland | Mammary_Gland_Involution.Endothelial_cell_Aqp1_high | 14459 | 0.0274 | 0.056 | 0.31251 |
| DropViz_FC_level2 | Neuron_Slc17a7_Parm1_Nnat.7_2 | 14805 | 0.00419 | 0.00859 | 0.31276 |
| GSE82187_Mouse_Striatum | Vascular | 13103 | 0.0152 | 0.0313 | 0.31346 |
| DropViz_all_level2 | FC.Neuron_Slc17a7_Parm1_Cadm2.7_5 | 14805 | 0.00267 | 0.00554 | 0.31494 |
| MouseCellAtlas_Adult_all | Pancreas.Endocrine_cell | 14459 | 0.0329 | 0.0684 | 0.31497 |
| MouseCellAtlas_all | Bladder.NK_cell | 14459 | 0.0366 | 0.076 | 0.3152 |
| DropViz_all_level2 | CB.Polydendrocyte_Tnr_Tnr.6_3 | 14805 | 0.00145 | 0.00302 | 0.31536 |
| MouseCellAtlas_Placenta | Monocyte | 14459 | 0.0397 | 0.0827 | 0.31545 |
| GSE93374_Mouse_Arc_ME_neurons | Rgs16_Dlx1 | 13325 | 0.132 | 0.274 | 0.31548 |
| Linnarsson_GSE74672_Mouse_Hypothalamus_Neurons_level2 | Vglut2_13NinlRfx5Zfp346 | 14550 | 0.0132 | 0.0274 | 0.3155 |
| DropViz_ENT_level2 | Polydendrocyte_Tnr_Bmp4.5_1 | 14805 | 0.0025 | 0.00521 | 0.31561 |
| DropViz_all_level2 | SN.Mural_Rgs5Acta2_Fos.13_5 | 14805 | 0.00169 | 0.00353 | 0.31614 |
| DropViz_HC_level2 | Oligodendrocyte_Trf_Plin3.8_1 | 14805 | 0.00243 | 0.0051 | 0.31657 |
| MouseCellAtlas_all | Neonatal_Rib.Osteoblast | 14459 | 0.0259 | 0.0544 | 0.31688 |
| Linnarsson_MouseBrainAtlas_level5 | HBGLU9 | 15140 | 0.0143 | 0.03 | 0.31716 |
| MouseCellAtlas_Stomach | Epithelial_cell_Gkn3_high | 14459 | 0.0282 | 0.0595 | 0.31755 |
| DropViz_all_level2 | HC.Neurogenesis_Sox4_Cdc20.13_3 | 14805 | 0.00177 | 0.00373 | 0.31762 |
| MouseCellAtlas_Mammary_Gland | Mammary_Gland_Lactation.Secretory_alveoli_cell_Ighm1_high | 14459 | 0.0561 | 0.118 | 0.31772 |
| DropViz_all_level1 | CB.Microglia | 14805 | 0.00151 | 0.00319 | 0.31808 |
| TabulaMuris_droplet_all | Bladder.bladder_cell | 13888 | 0.0105 | 0.0222 | 0.3183 |
| Linnarsson_GSE95752_Mouse_Dentate_gyrus | Neuroblast | 12517 | 0.0345 | 0.0731 | 0.31846 |
| MouseCellAtlas_Kidney | Fenestrated_endothelial_cell_Tm4sf1_high | 14459 | 0.0391 | 0.0829 | 0.31861 |
| MouseCellAtlas_Adult_all | Bone_Marrow.Endothelial_cells | 14459 | 0.0345 | 0.0732 | 0.31885 |
| Linnarsson_GSE103840_Mouse_Dorsal_horn | Glut_Qrfpr | 14550 | 0.031 | 0.0658 | 0.31891 |
| DropViz_all_level2 | STR.Mural_Rgs5Acta2_Col3a1.8_5 | 14805 | 0.00136 | 0.0029 | 0.31915 |
| DropViz_all_level1 | STR.Endothelial_stalk | 14805 | 0.00287 | 0.00611 | 0.31929 |
| Linnarsson_GSE74672_Mouse_Hypothalamus_level1 | vsm | 14550 | 0.0263 | 0.0561 | 0.31933 |
| DropViz_PC_level2 | Neuron_Sc17a7_Calb1-Otof.2_6 | 14805 | 0.00484 | 0.0103 | 0.31991 |
| DropViz_all_level2 | TH.Neuron_Slc17a6_Rora_Lypd6.2_7 | 14805 | 0.00319 | 0.00682 | 0.32023 |
| Linnarsson_GSE103840_Mouse_Dorsal_horn | GABA_Calb2_Tac1 | 14550 | 0.0282 | 0.0608 | 0.32149 |
| TabulaMuris_droplet_Marrow | T_cell | 13888 | 0.0212 | 0.0457 | 0.32156 |
| MouseCellAtlas_Neonatal_all | Neonatal_Skin.Macrophage_Pf4_high | 14459 | 0.0282 | 0.0611 | 0.32217 |
| MouseCellAtlas_Neonatal_Calvaria | Neuron | 14459 | 0.0537 | 0.117 | 0.32309 |
| MouseCellAtlas_all | Fetal_Brain.Erythroblast_Hbb.bh1_high | 14459 | 0.0256 | 0.0558 | 0.32313 |
| MouseCellAtlas_Fetal_Intestine | Enteroendocrine | 14459 | 0.0386 | 0.0842 | 0.32345 |
| Allen_Mouse_VISp_level2 | L6b_Rgs12 | 13868 | 0.00517 | 0.0113 | 0.32381 |
| Linnarsson_GSE60361_Mouse_Cortex_Hippocampus_level2 | Vsmc | 14010 | 0.0146 | 0.0319 | 0.32399 |
| DropViz_all_level2 | ENT.Oligodendrocyte_Trf_Ndrg2.1_3 | 14805 | 0.00242 | 0.00531 | 0.32413 |
| Linnarsson_MouseBrainAtlas_level5 | HBGLU2 | 15140 | 0.0172 | 0.0379 | 0.32547 |
| MouseCellAtlas_all | Placenta.PE_lineage_cell_S100g_high | 14459 | 0.0436 | 0.0965 | 0.32556 |
| GSE67835_Human_Cortex_woFetal | endothelial | 16585 | 0.0038 | 0.00842 | 0.32571 |
| Linnarsson_MouseBrainAtlas_level5 | TEGLU14 | 15140 | 0.0125 | 0.0277 | 0.32593 |
| MouseCellAtlas_all | Brain.Oligodendrocyte_precursor_cell | 14459 | 0.0297 | 0.0659 | 0.32629 |
| MouseCellAtlas_Adult_all | Mammary_Gland.Secretory_alveoli_cell | 14459 | 0.024 | 0.0535 | 0.3268 |
| DropViz_STR_level2 | Neuron_Gad1Gad2_Pnoc.14_2 | 14805 | 0.00183 | 0.00409 | 0.32692 |
| DropViz_HC_level2 | Neuron_Slc17a7_Pvrl3-Inhba.6_7 | 14805 | 0.004 | 0.00893 | 0.32707 |
| MouseCellAtlas_Fetal_Liver | Hepatocyte_Afp_high | 14459 | 0.023 | 0.0512 | 0.3271 |
| Allen_Mouse_VISp_level1 | Gluta_L5b | 13868 | 0.00572 | 0.0128 | 0.32716 |
| MouseCellAtlas_all | Fetal_Intestine.Enteroendocrine | 14459 | 0.0331 | 0.0739 | 0.32717 |
| TabulaMuris_FACS_Kidney | fibroblast | 13888 | 0.00988 | 0.0221 | 0.32717 |
| MouseCellAtlas_Stomach | Epithelial_cell_Pla2g1b_high | 14459 | 0.0253 | 0.0567 | 0.32744 |
| DropViz_all_level2 | ENT.Oligodendrocyte_Trf_Cldn11.8_1 | 14805 | 0.00171 | 0.00384 | 0.32772 |
| DropViz_all_level2 | GP.Oligodendrocyte_Trf_Mbp.10_1 | 14805 | 0.0027 | 0.00605 | 0.32785 |
| MouseCellAtlas_all | Embryonic_Mesenchyme.Erythroid_Cell | 14459 | 0.027 | 0.0608 | 0.32847 |
| MouseCellAtlas_Mammary_Gland | Mammary_Gland_Lactation.Secretory_alveoli_cell_Rhob_high | 14459 | 0.0396 | 0.0894 | 0.32892 |
| Linnarsson_MouseBrainAtlas_level5 | SCGLU5 | 15140 | 0.0222 | 0.0501 | 0.32922 |
| DropViz_all_level2 | FC.Neuron_Slc17a7_Parm1_Lsamp.7_3 | 14805 | 0.00353 | 0.00801 | 0.32966 |
| MouseCellAtlas_Peripheral_Blood | Monocyte_Elane_high | 14459 | 0.027 | 0.0613 | 0.32969 |
| TabulaMuris_droplet_Lung | type_II_pneumocyte | 13888 | 0.0147 | 0.0334 | 0.32973 |
| MouseCellAtlas_Stomach | G_cell | 14459 | 0.0311 | 0.0707 | 0.32977 |
| MouseCellAtlas_Adult_all | Brain.Oligodendrocyte_precursor_cell | 14459 | 0.0287 | 0.0653 | 0.33 |
| MouseCellAtlas_Trophoblast_Stem_Cell | TS_Mrpl12_high | 14459 | 0.087 | 0.198 | 0.33008 |
| Linnarsson_MouseBrainAtlas_level5 | SCHW | 15140 | 0.0107 | 0.0243 | 0.33019 |
| MouseCellAtlas_Adult_all | Mammary_Gland.Endothelial_cell_Fabp4_high | 14459 | 0.0344 | 0.0782 | 0.33024 |
| Linnarsson_MouseBrainAtlas_level5 | TEGLU20 | 15140 | 0.0149 | 0.0339 | 0.33036 |
| Linnarsson_GSE76381_Mouse_Midbrain | RN | 14550 | 0.0222 | 0.0506 | 0.33064 |
| DropViz_all_level2 | SN.Polydendrocyte_Tnr_Bmp4.5_1 | 14805 | 0.00204 | 0.00467 | 0.33075 |
| MouseCellAtlas_Adult_all | Small_Intestine.T_cell_Cd7_high | 14459 | 0.0319 | 0.073 | 0.33126 |
| MouseCellAtlas_Adult_all | Peripheral_Blood.Dendritic_cell_Siglech_high | 14459 | 0.0396 | 0.0908 | 0.33142 |
| DropViz_all_level2 | HC.Neuron_Slc17a7_Fibcd1-Lypd1.5_1 | 14805 | 0.00341 | 0.00784 | 0.33199 |
| TabulaMuris_FACS_all | Pancreas.leukocyte | 13888 | 0.00387 | 0.00891 | 0.3321 |
| MouseCellAtlas_Bone_Marrow | Bone_Marrow_c_kit.Monocyte_progenitor_cell_Ctsg_high | 14459 | 0.0314 | 0.0725 | 0.33235 |
| DroNc_Human_Hippocampus | ODC2 | 16914 | 0.0726 | 0.168 | 0.33248 |
| MouseCellAtlas_Adult_all | Lung.Alveolar_macrophage_Ear2_high | 14459 | 0.036 | 0.0832 | 0.33265 |
| MouseCellAtlas_Adult_all | Small_Intestine.T_cell_Ccl5_high | 14459 | 0.0393 | 0.0914 | 0.33345 |
| TabulaMuris_FACS_all | Brain_Neurons.oligodendrocyte | 13888 | 0.00224 | 0.0052 | 0.33349 |
| MouseCellAtlas_Adult_all | Mammary_Gland.Dendritic_cell_Cd209a_high | 14459 | 0.0263 | 0.0611 | 0.33352 |
| GSE87544_Mouse_Hypothalamus | GABA12 | 13878 | 0.0328 | 0.0763 | 0.33369 |
| MouseCellAtlas_Adult_all | Mammary_Gland.Endothelial_cell_Glycam1_high | 14459 | 0.0295 | 0.0688 | 0.3341 |
| TabulaMuris_FACS_all | Heart.leukocyte | 13888 | 0.00413 | 0.00966 | 0.33451 |
| Linnarsson_GSE101601_Mouse_Somatosensory_cortex | Endothelial_bmx | 14550 | 0.0246 | 0.0577 | 0.33472 |
| DropViz_all_level2 | HC.Neurogenesis_Sox4_Ppp2r2b.13_6 | 14805 | 0.00193 | 0.00452 | 0.3348 |
| GSE93374_Mouse_Arc_ME_level1 | Tanycyte1 | 13325 | 0.0432 | 0.101 | 0.3351 |
| MouseCellAtlas_Embryo_all | Fetal_Intestine.Enterocyte_progenitor_late_Ccnb1_high | 14459 | 0.0539 | 0.127 | 0.33533 |
| MouseCellAtlas_Mammary_Gland | Mammary_Gland_Lactation.Secretory_alveoli_cell_Fcer1g_high | 14459 | 0.0276 | 0.065 | 0.33543 |
| DropViz_all_level2 | SN.Neuron_Th_Aldh1a1.4_8 | 14805 | 0.00271 | 0.00639 | 0.33561 |
| GSE87544_Mouse_Hypothalamus | Micro | 13878 | 0.0225 | 0.0531 | 0.33561 |
| DropViz_PC_level1 | Endothelial_tip | 14805 | 0.00299 | 0.00704 | 0.33568 |
| DropViz_SN_level2 | Neuron_Th_Nefl.4_7 | 14805 | 0.00267 | 0.0063 | 0.33591 |
| MouseCellAtlas_Neonatal_Heart | Endothelial_cell_Igfbp5_high | 14459 | 0.057 | 0.135 | 0.33595 |
| DropViz_all_level2 | SN.Neuron_Th_Grin2c.4_9 | 14805 | 0.00273 | 0.00647 | 0.33631 |
| DropViz_all_level1 | GP.Microglia | 14805 | 0.00196 | 0.00464 | 0.33633 |
| TabulaMuris_FACS_all | Skin.basal_cell_of_epidermis | 13888 | 0.00297 | 0.00702 | 0.3364 |
| MouseCellAtlas_Mammary_Gland | Mammary_Gland_Lactation.Secretory_alveoli_cell_Igfbp7_high | 14459 | 0.0367 | 0.0869 | 0.33662 |
| MouseCellAtlas_Embryonic_Mesenchyme | Erythroid_Cell | 14459 | 0.0292 | 0.0696 | 0.33719 |
| MouseCellAtlas_all | Fetal_Brain.Dopaminergic_neurons_ | 14459 | 0.0295 | 0.0704 | 0.33745 |
| MouseCellAtlas_Embryo_all | Fetal_Intestine.Erythroblast_Car2_high | 14459 | 0.0261 | 0.0621 | 0.33747 |
| MouseCellAtlas_Adult_all | Mammary_Gland.Secretory_alveoli_cell_Fth1.Cib1_high | 14459 | 0.0175 | 0.0418 | 0.33778 |
| MouseCellAtlas_Mammary_Gland | Mammary_Gland_Pregnancy.Ductal_luminal_cell | 14459 | 0.0401 | 0.0958 | 0.33778 |
| TabulaMuris_FACS_all | Lung.monocyte | 13888 | 0.00312 | 0.00747 | 0.33799 |
| MouseCellAtlas_Adult_all | Bladder.Basal_epithelial_cell | 14459 | 0.0189 | 0.0454 | 0.33841 |
| MouseCellAtlas_all | Neonatal_Rib.Muscle_cell_Acta2_high | 14459 | 0.0258 | 0.0621 | 0.33871 |
| DropViz_STR_level2 | Mural_Rgs5Acta2_Ccnd1.8_3 | 14805 | 0.00174 | 0.00418 | 0.33877 |
| DropViz_all_level2 | STR.Polydendrocye_Tnr_Pdgfa.5_3 | 14805 | 0.00177 | 0.00426 | 0.33889 |
| MouseCellAtlas_Brain | Macrophage_Klf2_high | 14459 | 0.0286 | 0.0691 | 0.33915 |
| MouseCellAtlas_all | Bladder.Basal_epithelial_cell | 14459 | 0.0191 | 0.0461 | 0.33922 |
| DropViz_HC_level2 | Neuron_Slc17a6_Nnat.2_4 | 14805 | 0.00321 | 0.00774 | 0.33926 |
| DropViz_all_level2 | GP.Polydendrocyte_Tnr_Bmp4.4_4 | 14805 | 0.00236 | 0.00571 | 0.33931 |
| DropViz_PC_level2 | Endothelial_Tip_Dcn_Coch.14_4 | 14805 | 0.00173 | 0.00419 | 0.33949 |
| MouseCellAtlas_Stomach | Epithelial_cell_Krt20_high | 14459 | 0.0352 | 0.0852 | 0.33969 |
| DropViz_STR_level1 | Microglia | 14805 | 0.00238 | 0.00575 | 0.33976 |
| DropViz_all_level1 | SN.Polydendrocyte | 14805 | 0.00296 | 0.00719 | 0.34024 |
| MouseCellAtlas_Embryo_all | Fetal_Stomache.Erythroblast_Hba.x_HIGH | 14459 | 0.0362 | 0.088 | 0.34046 |
| Linnarsson_MouseBrainAtlas_level6_rank4 | Vsmc | 15140 | 0.0192 | 0.0468 | 0.3405 |
| MouseCellAtlas_Neonatal_all | Neonatal_Skin.Adipocyte | 14459 | 0.0256 | 0.0623 | 0.34076 |
| MouseCellAtlas_all | Mammary_Gland.Endothelial_cell_Fabp4_high | 14459 | 0.0318 | 0.0777 | 0.34104 |
| Linnarsson_GSE74672_Mouse_Hypothalamus_level1 | ependymal | 14550 | 0.0192 | 0.0469 | 0.34114 |
| Linnarsson_GSE74672_Mouse_Hypothalamus_Neurons_level2 | Qrfp | 14550 | 0.00854 | 0.0209 | 0.34138 |
| Linnarsson_GSE60361_Mouse_Cortex_Hippocampus_level2 | Choroid | 14010 | 0.0131 | 0.0322 | 0.3415 |
| Linnarsson_GSE76381_Human_Midbrain | NbM | 14835 | 0.0301 | 0.0738 | 0.34166 |
| MouseCellAtlas_all | Neonatal_Skin.Mast_cell | 14459 | 0.0257 | 0.0629 | 0.34176 |
| Linnarsson_MouseBrainAtlas_level5 | HBCHO4 | 15140 | 0.0102 | 0.0252 | 0.34195 |
| DropViz_FC_level1 | Microglia | 14805 | 0.00206 | 0.00507 | 0.34225 |
| TabulaMuris_FACS_Lung | type_II_pneumocyte | 13888 | 0.00336 | 0.00828 | 0.34268 |
| DropViz_all_level1 | SN.Mural | 14805 | 0.00243 | 0.006 | 0.34279 |
| MouseCellAtlas_Prostate | Prostate_gland_cell | 14459 | 0.137 | 0.339 | 0.34311 |
| MouseCellAtlas_all | Neonatal_Skin.Neutrophil | 14459 | 0.0207 | 0.0512 | 0.3433 |
| TabulaMuris_droplet_all | Marrow.macrophage | 13888 | 0.0145 | 0.0361 | 0.34381 |
| Linnarsson_GSE101601_Mouse_Somatosensory_cortex | Pyramidal_cells_layer_23_Ddn | 14550 | 0.0123 | 0.0307 | 0.34383 |
| MouseCellAtlas_Adult_all | Spleen.Marginal_zone_B_cell | 14459 | 0.0353 | 0.0879 | 0.3441 |
| MouseCellAtlas_Adult_all | Mammary_Gland.Secretory_alveoli_cell_Thy1_high | 14459 | 0.0315 | 0.0785 | 0.34416 |
| MouseCellAtlas_Mammary_Gland | Mammary_Gland_Lactation.Secretory_alveoli_cell_mt.Tp_high | 14459 | 0.041 | 0.102 | 0.3443 |
| GSE87544_Mouse_Hypothalamus | GABA11 | 13878 | 0.0217 | 0.0543 | 0.34454 |
| DropViz_HC_level2 | Neuron_Slc17a7_Calb2-Adcyap1.6_6 | 14805 | 0.00345 | 0.00864 | 0.34482 |
| MouseCellAtlas_all | Peripheral_Blood.Dendritic_cell_Siglech_high | 14459 | 0.0359 | 0.0898 | 0.34483 |
| DropViz_all_level2 | GP.Neuron_Gad1Gad2_Vip.2_21 | 14805 | 0.00247 | 0.0062 | 0.34495 |
| Allen_Mouse_VISp_level2 | Oligo_Opalin | 13868 | 0.00274 | 0.00686 | 0.34496 |
| DropViz_all_level1 | GP.Ependymal | 14805 | 0.00146 | 0.00366 | 0.34521 |
| MouseCellAtlas_all | Mammary_Gland.Endothelial_cell_Glycam1_high | 14459 | 0.0271 | 0.0682 | 0.34558 |
| Allen_Mouse_ALM_level2 | Vip_Sfrp2_2 | 14744 | 0.00509 | 0.0128 | 0.34575 |
| MouseCellAtlas_Fetal_Brain | Erythroblast_Hbb.bh1_high | 14459 | 0.0272 | 0.069 | 0.3466 |
| Linnarsson_GSE74672_Mouse_Hypothalamus_Neurons_level2 | Vglut2_7_PgamSnx12 | 14550 | 0.0129 | 0.0331 | 0.3481 |
| DropViz_all_level2 | FC.Neuron_Slc17a7_Parm1_Ndnf.7_1 | 14805 | 0.00293 | 0.00752 | 0.34817 |
| DropViz_GP_level2 | Macrophage_C1qb_Mrc1.11_2 | 14805 | 0.00151 | 0.00388 | 0.34865 |
| MouseCellAtlas_Adult_all | Mammary_Gland.Macrophage_Lyz1_high | 14459 | 0.0313 | 0.0805 | 0.34866 |
| Linnarsson_GSE60361_Mouse_Cortex_Hippocampus_level2 | Pvm1 | 14010 | 0.00941 | 0.0243 | 0.34915 |
| MouseCellAtlas_Mammary_Gland | Mammary_Gland_Lactation.Secretory_alveoli_cell_Rgs2_high_Rora_high | 14459 | 0.0329 | 0.0849 | 0.34915 |
| GSE93374_Mouse_Arc_ME_level1 | PVMMicro | 13325 | 0.0215 | 0.0555 | 0.34919 |
| MouseCellAtlas_Fetal_Intestine | Enterocyte_progenitor_late_Ccnb1_high | 14459 | 0.0598 | 0.154 | 0.34925 |
| MouseCellAtlas_all | Small_Intestine.T_cell_Cd7_high | 14459 | 0.0277 | 0.0715 | 0.34947 |
| MouseCellAtlas_all | Mammary_Gland.Secretory_alveoli_cell | 14459 | 0.0201 | 0.052 | 0.34968 |
| DropViz_TH_level2 | Oligodendrocyte_Trf_Il33.8_2 | 14805 | 0.00247 | 0.00641 | 0.34979 |
| MouseCellAtlas_Adult_all | Kidney.Fenestrated_endothelial_cell_Tm4sf1_high | 14459 | 0.0316 | 0.0819 | 0.34979 |
| DropViz_all_level2 | TH.Neuron_Slc17a6_Rora_Fgf10.2_6 | 14805 | 0.00261 | 0.00676 | 0.34982 |
| MouseCellAtlas_all | Fetal_Brain.Ependymal_cell | 14459 | 0.029 | 0.0751 | 0.34991 |
| MouseCellAtlas_Embryo_all | Fetal_Lung.Epithelial_cell_Sftpc_high | 14459 | 0.0515 | 0.135 | 0.35085 |
| MouseCellAtlas_Embryo_all | Fetal_Stomache.Epithelial_cell | 14459 | 0.0659 | 0.172 | 0.35085 |
| DropViz_all_level2 | PC.Endothelial_Tip_Dcn_Coch.14_4 | 14805 | 0.00168 | 0.00439 | 0.35087 |
| MouseCellAtlas_all | Lung.Alveolar_macrophage_Ear2_high | 14459 | 0.0312 | 0.0814 | 0.35089 |
| MouseCellAtlas_all | Small_Intestine.T_cell_Ccl5_high | 14459 | 0.0341 | 0.0894 | 0.35166 |
| MouseCellAtlas_Pancreas | Acinar_cell | 14459 | 0.0198 | 0.052 | 0.3518 |
| MouseCellAtlas_all | Mammary_Gland.T_cell_Cd8b1_high | 14459 | 0.0313 | 0.0823 | 0.35181 |
| DropViz_TH_level2 | Mural_Rgs5Acta2_Car4.5_2 | 14805 | 0.00191 | 0.00503 | 0.35192 |
| DropViz_all_level2 | ENT.Oligodendrocyte_Trf_Serpinb1a.1_1 | 14805 | 0.00196 | 0.00515 | 0.35195 |
| Linnarsson_GSE101601_Mouse_Somatosensory_cortex | Myelin_forming_oligodendrocyte | 14550 | 0.0121 | 0.0319 | 0.35197 |
| MouseCellAtlas_Adult_all | Mammary_Gland.T_cell_Cd8b1_high | 14459 | 0.0306 | 0.0806 | 0.35226 |
| GSE93374_Mouse_Arc_ME_neurons | Pomc_Glipr1 | 13325 | 0.16 | 0.423 | 0.35233 |
| DropViz_FC_level2 | Endothelial_Tip_Dcn_1500015O10Rik.14_1 | 14805 | 0.00164 | 0.00434 | 0.35262 |
| MouseCellAtlas_all | Bone_Marrow.Granulocyte_monocyte_progenitors | 14459 | 0.0201 | 0.0534 | 0.35331 |
| DropViz_all_level2 | HC.Neuron_Slc17a7_Fibcd1-Kitl.5_4 | 14805 | 0.0028 | 0.00744 | 0.35341 |
| DropViz_all_level1 | PC.Endothelial_tip | 14805 | 0.00254 | 0.00676 | 0.35357 |
| Linnarsson_GSE74672_Mouse_Hypothalamus_Neurons_level2 | Vglut2_11 | 14550 | 0.00723 | 0.0192 | 0.35363 |
| DropViz_SN_level2 | Mural_Rgs5Acta2_Cbr2.13_4 | 14805 | 0.00151 | 0.00404 | 0.35411 |
| Linnarsson_MouseBrainAtlas_level5 | PSNP2 | 15140 | 0.00586 | 0.0157 | 0.3546 |
| TabulaMuris_FACS_Mammary | endothelial_cell | 13888 | 0.00368 | 0.00988 | 0.35469 |
| MouseCellAtlas_Neonatal_Rib | Neuron_Stmn2_high | 14459 | 0.0208 | 0.0559 | 0.35478 |
| DropViz_all_level2 | HC.Neuron_Slc17a7_Dcn-Pou3f1.5_6 | 14805 | 0.00295 | 0.00793 | 0.35481 |
| MouseCellAtlas_Adult_all | Peripheral_Blood.Macrophage_Pf4_high | 14459 | 0.0185 | 0.0496 | 0.35486 |
| MouseCellAtlas_Adult_all | Mammary_Gland.Dendritic_cell_Il1b_high | 14459 | 0.0224 | 0.0604 | 0.35508 |
| MouseCellAtlas_Adult_all | Bone_Marrow.Granulocyte_monocyte_progenitors | 14459 | 0.0195 | 0.0525 | 0.35515 |
| DropViz_all_level2 | STR.Endothelial_Stalk_Flt1_Car4.7_1 | 14805 | 0.00178 | 0.00478 | 0.35523 |
| TabulaMuris_droplet_Thymus | stromal_cell | 13888 | 0.0363 | 0.098 | 0.35544 |
| Linnarsson_GSE101601_Human_Temporal_cortex | GABA_5 | 17177 | 0.0681 | 0.184 | 0.35586 |
| Linnarsson_GSE74672_Mouse_Hypothalamus_Neurons_level2 | Vglut2_4 | 14550 | 0.00889 | 0.0241 | 0.35605 |
| Linnarsson_GSE76381_Human_Midbrain | OMTN | 14835 | 0.022 | 0.0596 | 0.35605 |
| DropViz_STR_level2 | Neuron_Slc17a7_Cplx3.13_7 | 14805 | 0.00177 | 0.0048 | 0.3561 |
| TabulaMuris_droplet_Heart | endocardial_cell | 13888 | 0.0157 | 0.0428 | 0.35695 |
| Linnarsson_GSE78845_Mouse_Ganglia | Cholinergic_neurons__1 | 12817 | 0.0111 | 0.0305 | 0.35815 |
| DropViz_PC_level2 | Endothelial_Tip_Dcn_Mgp-Penk.14_1 | 14805 | 0.00181 | 0.005 | 0.35835 |
| GSE87544_Mouse_Hypothalamus | Glu8 | 13878 | 0.0255 | 0.0704 | 0.3585 |
| MouseCellAtlas_all | Peripheral_Blood.Macrophage_Pf4_high | 14459 | 0.018 | 0.0496 | 0.35874 |
| MouseCellAtlas_all | Spleen.Marginal_zone_B_cell | 14459 | 0.0313 | 0.0867 | 0.35908 |
| TabulaMuris_FACS_Trachea | epithelial_cell | 13888 | 0.006 | 0.0166 | 0.35911 |
| Allen_Mouse_ALM_level1 | Vip | 14744 | 0.00509 | 0.0142 | 0.35971 |
| MouseCellAtlas_Brain | Astroglial_cell | 14459 | 0.0221 | 0.0617 | 0.36012 |
| MouseCellAtlas_Adult_all | Kidney.Proximal_tubule_cell_Cyp4a14_high | 14459 | 0.0193 | 0.0541 | 0.36023 |
| Linnarsson_GSE60361_Mouse_Cortex_Hippocampus_level2 | CA1Pyr2 | 14010 | 0.0111 | 0.031 | 0.36027 |
| Allen_Mouse_ALM_level2 | L5_ALM_Arhgap25_1 | 14744 | 0.00394 | 0.0111 | 0.36067 |
| DropViz_HC_level2 | Macrophage_C1qb_Mrc1.10_2 | 14805 | 0.00118 | 0.00332 | 0.36111 |
| Linnarsson_GSE59739_Mouse_Dorsal_root_ganglion_level3 | Neurofilament_containing1 | 13848 | 0.00426 | 0.012 | 0.36114 |
| TabulaMuris_droplet_all | Trachea.basal_cell_of_epithelium_of_trachea | 13888 | 0.0158 | 0.0444 | 0.36117 |
| MouseCellAtlas_all | Mammary_Gland.Secretory_alveoli_cell_Fth1.Cib1_high | 14459 | 0.0146 | 0.0412 | 0.3617 |
| MouseCellAtlas_Bone_Marrow | Bone_Marrow_c_kit.Monocyte_progenitor_cell_Prtn3_high | 14459 | 0.0325 | 0.092 | 0.36203 |
| DropViz_all_level2 | FC.Endothelial_Tip_Dcn_1500015O10Rik.14_1 | 14805 | 0.00157 | 0.00444 | 0.36206 |
| MouseCellAtlas_all | Mammary_Gland.Dendritic_cell_Cd209a_high | 14459 | 0.0206 | 0.0585 | 0.36234 |
| MouseCellAtlas_all | Kidney.Fenestrated_endothelial_cell_Tm4sf1_high | 14459 | 0.0285 | 0.081 | 0.36244 |
| TabulaMuris_droplet_Lung | monocyte | 13888 | 0.0131 | 0.0373 | 0.36276 |
| MouseCellAtlas_Adult_all | Bladder.Urothelium | 14459 | 0.011 | 0.0314 | 0.36302 |
| DropViz_STR_level2 | Neurogenesis_Sox4_Tuba1c.2_3 | 14805 | 0.00165 | 0.00473 | 0.3633 |
| MouseCellAtlas_all | Placenta.Spiral_artery_trophoblast_giant_cells | 14459 | 0.023 | 0.0659 | 0.36338 |
| DropViz_CB_level2 | Gja1_Htra1.8_2 | 14805 | 0.00167 | 0.00478 | 0.36365 |
| DropViz_all_level2 | CB.Gja1_Htra1.8_2 | 14805 | 0.00149 | 0.00428 | 0.36369 |
| GSE93374_Mouse_Arc_ME_neurons | Pomc_Ttr | 13325 | 0.122 | 0.349 | 0.36377 |
| MouseCellAtlas_Fetal_Stomache | Neuron | 14459 | 0.0475 | 0.137 | 0.36381 |
| MouseCellAtlas_Adult_all | Small_Intestine.B_cell_Ighd_high | 14459 | 0.0283 | 0.0813 | 0.36389 |
| TabulaMuris_FACS_all | Marrow.neutrophil | 13888 | 0.00231 | 0.00665 | 0.36422 |
| Allen_Mouse_VISp_level1 | Gluta_L5 | 13868 | 0.00421 | 0.0121 | 0.36423 |
| TabulaMuris_droplet_all | Lung.type_II_pneumocyte | 13888 | 0.0108 | 0.0312 | 0.3645 |
| MouseCellAtlas_Kidney | Endothelial_cell | 14459 | 0.0258 | 0.0744 | 0.36456 |
| MouseCellAtlas_all | Fetal_Brain.Granule_neurons | 14459 | 0.0276 | 0.0796 | 0.36457 |
| GSE106678_Mouse_Cortex | Ex3 | 14573 | 0.0333 | 0.0965 | 0.36482 |
| MouseCellAtlas_Embryo_all | Placenta.Spiral_artery_trophoblast_giant_cells | 14459 | 0.0243 | 0.0704 | 0.36515 |
| DropViz_all_level2 | GP.Neuron_Gad1Gad2_Tac2.2_3 | 14805 | 0.00264 | 0.00768 | 0.36545 |
| MouseCellAtlas_Adult_all | Mammary_Gland.B_cell_Cd79a.Iglc2_high | 14459 | 0.0297 | 0.0867 | 0.36612 |
| DropViz_PC_level2 | Neuron_Gad1Gad2_Sst-Nr2f2.5_7 | 14805 | 0.00182 | 0.00532 | 0.3662 |
| MouseCellAtlas_all | Mammary_Gland.Dendritic_cell_Il1b_high | 14459 | 0.0204 | 0.0599 | 0.36648 |
| DropViz_PC_level1 | Macrophage | 14805 | 0.0017 | 0.00497 | 0.36651 |
| MouseCellAtlas_Adult_all | Muscle.T_cell | 14459 | 0.0173 | 0.0508 | 0.3666 |
| MouseCellAtlas_Brain | Granulocyte_Ngp_high | 14459 | 0.00915 | 0.0268 | 0.36666 |
| TabulaMuris_FACS_Liver | natural_killer_cell | 13888 | 0.00301 | 0.00889 | 0.36729 |
| MouseCellAtlas_Pancreas | Endothelial_cell_Tm4sf1_high | 14459 | 0.0326 | 0.0962 | 0.36733 |
| TabulaMuris_FACS_all | Liver.endothelial_cell_of_hepatic_sinusoid | 13888 | 0.00266 | 0.00787 | 0.36742 |
| DropViz_GP_level2 | Polydendrocyte_Tnr_Opalin.4_3 | 14805 | 0.00149 | 0.0044 | 0.36748 |
| GSE93374_Mouse_Arc_ME_neurons | Tmem215 | 13325 | 0.204 | 0.603 | 0.36753 |
| DropViz_all_level2 | TH.Neuron_Slc17a7_Tac2_Syt15.1_2 | 14805 | 0.002 | 0.00592 | 0.36764 |
| MouseCellAtlas_all | Kidney.Proximal_tubule_cell_Cyp4a14_high | 14459 | 0.0181 | 0.0538 | 0.36817 |
| MouseCellAtlas_all | Bladder.Urothelium | 14459 | 0.0106 | 0.0315 | 0.36877 |
| MouseCellAtlas_all | Peripheral_Blood.T_cell_Trbc2_high | 14459 | 0.0268 | 0.08 | 0.36888 |
| MouseCellAtlas_Neonatal_all | Neonatal_Rib.Osteoblast | 14459 | 0.0222 | 0.0663 | 0.36893 |
| Linnarsson_GSE101601_Human_Temporal_cortex | Glut_5 | 17177 | 0.069 | 0.207 | 0.36917 |
| MouseCellAtlas_Neonatal_Skin | Dividing_cell | 14459 | 0.0387 | 0.116 | 0.36942 |
| DropViz_TH_level2 | Astrocyte_Gja1_Myoc.11_1 | 14805 | 0.00138 | 0.00414 | 0.36971 |
| MouseCellAtlas_Neonatal_all | Neonatal_Rib.Muscle_cell_Acta2_high | 14459 | 0.0241 | 0.0725 | 0.36985 |
| DropViz_all_level1 | STR.Macrophage | 14805 | 0.00107 | 0.00321 | 0.3701 |
| MouseCellAtlas_Ovary | Granulosa_cell_Kctd14_high | 14459 | 0.0444 | 0.134 | 0.37066 |
| GSE93374_Mouse_Arc_ME_level2 | Oligodendro3 | 13325 | 0.0123 | 0.0373 | 0.37073 |
| MouseCellAtlas_all | Mammary_Gland.Secretory_alveoli_cell_Thy1_high | 14459 | 0.0252 | 0.0766 | 0.37101 |
| MouseCellAtlas_Embryo_all | Fetal_Brain.Schwann_cell | 14459 | 0.0279 | 0.085 | 0.37126 |
| MouseCellAtlas_Small_Intestine | T_cell_Cd7_high | 14459 | 0.0265 | 0.0807 | 0.37144 |
| Allen_Mouse_ALM_level2 | Sst_Syndig1l_1 | 14744 | 0.00269 | 0.0082 | 0.37151 |
| DropViz_all_level2 | HC.Neuron_Slc17a7_Cbln4.2_1 | 14805 | 0.0023 | 0.00704 | 0.37181 |
| MouseCellAtlas_Brain | Hypothalamic_ependymal_cell | 14459 | 0.0135 | 0.0413 | 0.37187 |
| MouseCellAtlas_Adult_all | Peripheral_Blood.T_cell_Trbc2_high | 14459 | 0.0253 | 0.0778 | 0.37241 |
| DropViz_FC_level2 | Neuron_Slc17a7_Parm1_Tshz2.7_7 | 14805 | 0.00258 | 0.00793 | 0.37242 |
| GSE67835_Human_Cortex | microglia | 16585 | 0.00246 | 0.0076 | 0.37294 |
| DropViz_all_level2 | FC.Neuron_Slc17a7_Parm1_Nnat.7_2 | 14805 | 0.00237 | 0.00735 | 0.37373 |
| MouseCellAtlas_Pancreas | Granulocyte | 14459 | 0.0136 | 0.0425 | 0.3741 |
| DropViz_HC_level1 | Oligodendrocyte | 14805 | 0.00203 | 0.00633 | 0.37429 |
| MouseCellAtlas_Embryo_all | Placenta.PE_lineage_cell_S100g_high | 14459 | 0.0294 | 0.0919 | 0.37446 |
| MouseCellAtlas_all | Small_Intestine.B_cell_Ighd_high | 14459 | 0.0258 | 0.0809 | 0.37475 |
| MouseCellAtlas_Embryo_all | Placenta.Progenitor_trophoblast_Gjb3_high | 14459 | 0.0263 | 0.0826 | 0.37487 |
| DropViz_all_level2 | GP.Oligodendrocyte_Trf_Kif5b.10_6 | 14805 | 0.00188 | 0.0059 | 0.37512 |
| MouseCellAtlas_Adult_all | Kidney.B_cell | 14459 | 0.0146 | 0.0459 | 0.37523 |
| MouseCellAtlas_all | Kidney.B_cell | 14459 | 0.0146 | 0.0462 | 0.37585 |
| DropViz_CB_level1 | Polydendrocyte | 14805 | 0.00111 | 0.0035 | 0.37594 |
| MouseCellAtlas_all | Muscle.T_cell | 14459 | 0.0159 | 0.0505 | 0.37615 |
| Allen_Mouse_VISp_level2 | L4_Arf5 | 13868 | 0.00342 | 0.0108 | 0.3762 |
| Linnarsson_MouseBrainAtlas_level5 | OBNBL5 | 15140 | 0.0233 | 0.0739 | 0.37634 |
| MouseCellAtlas_all | Mammary_Gland.Myoepithelial_cell | 14459 | 0.0164 | 0.0521 | 0.37672 |
| MouseCellAtlas_all | Mammary_Gland.B_cell_Cd79a.Iglc2_high | 14459 | 0.0271 | 0.0864 | 0.37679 |
| MouseCellAtlas_Adult_all | Lung.Conventional_dendritic_cell_Mgl2_high | 14459 | 0.0216 | 0.0689 | 0.37716 |
| MouseCellAtlas_all | Fetal_Intestine.Enterocyte_progenitor | 14459 | 0.0364 | 0.116 | 0.37725 |
| MouseCellAtlas_Small_Intestine | T_cell_Ccl5_high | 14459 | 0.0313 | 0.101 | 0.37779 |
| DroNc_Human_Hippocampus | ODC1 | 16914 | 0.0622 | 0.2 | 0.37793 |
| TabulaMuris_FACS_Heart | endocardial_cell | 13888 | 0.00347 | 0.0112 | 0.37797 |
| MouseCellAtlas_all | Mammary_Gland.Macrophage_Lyz1_high | 14459 | 0.0237 | 0.0767 | 0.37876 |
| MouseCellAtlas_all | Neonatal_Muscle.Mast_cell | 14459 | 0.0192 | 0.0623 | 0.37878 |
| MouseCellAtlas_Embryo_all | Placenta.Monocyte | 14459 | 0.0203 | 0.066 | 0.37896 |
| Linnarsson_MouseBrainAtlas_level5 | HBCHO2 | 15140 | 0.00712 | 0.0232 | 0.37924 |
| Linnarsson_MouseBrainAtlas_level5 | SATG1 | 15140 | 0.00737 | 0.024 | 0.37931 |
| MouseCellAtlas_Fetal_Intestine | Erythroblast_Car2_high | 14459 | 0.0193 | 0.0628 | 0.37962 |
| DropViz_all_level2 | PC.Neuron_Sc17a7_Sema3e-Onecut2.2_18 | 14805 | 0.0024 | 0.00784 | 0.37965 |
| MouseCellAtlas_Placenta | Dendritic_cell | 14459 | 0.0287 | 0.0943 | 0.38041 |
| MouseCellAtlas_Lung | Conventional_dendritic_cell_H2.M2_high | 14459 | 0.0165 | 0.0543 | 0.38046 |
| MouseCellAtlas_all | Lung.Stromal_cell_Acta2_high | 14459 | 0.0229 | 0.0753 | 0.3806 |
| DropViz_HC_level2 | Endothelial_Tip_Dcn_Rbp4.17_1 | 14805 | 0.00127 | 0.00418 | 0.38075 |
| MouseCellAtlas_Fetal_Liver | Neutrophil_Ngp_high | 14459 | 0.0219 | 0.072 | 0.38078 |
| MouseCellAtlas_Mammary_Gland | Mammary_Gland_Lactation.Secretory_alveoli_cell_Tmsb10_high | 14459 | 0.0282 | 0.0933 | 0.38101 |
| Linnarsson_GSE103840_Mouse_Dorsal_horn | Glut_Elavl4_Lypd1 | 14550 | 0.0139 | 0.046 | 0.38151 |
| TabulaMuris_droplet_Trachea | stromal_cell | 13888 | 0.0182 | 0.0604 | 0.38152 |
| DropViz_all_level2 | TH.Macrophage_C1qb_Mrc1.7_1 | 14805 | 0.00109 | 0.00362 | 0.38179 |
| DropViz_all_level2 | PC.Endothelial_Tip_Dcn_Mgp-Penk.14_1 | 14805 | 0.00155 | 0.00516 | 0.3818 |
| MouseCellAtlas_Adult_all | Lung.Stromal_cell_Acta2_high | 14459 | 0.0223 | 0.0744 | 0.38229 |
| DropViz_all_level1 | TH.Astrocyte | 14805 | 0.00185 | 0.0062 | 0.38257 |
| DropViz_all_level1 | HC.Endothelial_tip | 14805 | 0.0018 | 0.00602 | 0.38269 |
| MouseCellAtlas_Adult_all | Lung.Clara_Cell | 14459 | 0.0169 | 0.0567 | 0.38286 |
| MouseCellAtlas_Neonatal_Muscle | Muscle_cell_Myl9_high | 14459 | 0.0276 | 0.0927 | 0.38303 |
| DropViz_all_level2 | FC.Neuron_Slc17a7_Nptxr-Deptor.6_4 | 14805 | 0.00247 | 0.00829 | 0.38304 |
| MouseCellAtlas_Adult_all | Mammary_Gland.Myoepithelial_cell | 14459 | 0.0149 | 0.0502 | 0.3833 |
| MouseCellAtlas_Adult_all | Mammary_Gland.Secretory_alveoli_cell_Ehf_high | 14459 | 0.0187 | 0.0629 | 0.38332 |
| DropViz_all_level2 | ENT.Astrocyte_Gja1_Htra1.7_2 | 14805 | 0.00144 | 0.00487 | 0.38351 |
| Linnarsson_GSE60361_Mouse_Cortex_Hippocampus_level1 | microglia | 14010 | 0.00992 | 0.0335 | 0.38375 |
| TabulaMuris_FACS_Marrow | neutrophil | 13888 | 0.00274 | 0.00927 | 0.38381 |
| MouseCellAtlas_all | Fetal_Stomache.Erythroblast_Hba.x_HIGH | 14459 | 0.0223 | 0.076 | 0.38447 |
| MouseCellAtlas_Spleen | NK_cell | 14459 | 0.036 | 0.123 | 0.38462 |
| Allen_Mouse_ALM_level2 | L5a_Hsd11b1_low | 14744 | 0.00185 | 0.0063 | 0.38463 |
| GSE93374_Mouse_Arc_ME_level1 | NG2_OPC | 13325 | 0.0324 | 0.111 | 0.38478 |
| MouseCellAtlas_Adult_all | Muscle.B_cell_Jchain_high | 14459 | 0.0195 | 0.0667 | 0.38496 |
| GSE106678_Mouse_Cortex | Ex24 | 14573 | 0.0387 | 0.132 | 0.38497 |
| GSE106678_Mouse_Cortex | Ex5 | 14573 | 0.0388 | 0.133 | 0.38501 |
| Allen_Mouse_VISp_level2 | L6b_Serpinb11 | 13868 | 0.00362 | 0.0124 | 0.38525 |
| MouseCellAtlas_Adult_all | Mammary_Gland.Secretory_alveoli_cell_Crip1_high | 14459 | 0.0165 | 0.0566 | 0.38527 |
| MouseCellAtlas_Adult_all | Spleen.Neutrophil | 14459 | 0.0122 | 0.0421 | 0.38577 |
| DropViz_STR_level2 | Ependyma_Ccdc153_Ccdc153.1_1 | 14805 | 0.00136 | 0.0047 | 0.38588 |
| MouseCellAtlas_all | Fetal_Intestine.Erythroblast_Car2_high | 14459 | 0.0154 | 0.0536 | 0.38723 |
| GSE93374_Mouse_Arc_ME_level2 | Microglia | 13325 | 0.016 | 0.056 | 0.38741 |
| MouseCellAtlas_Fetal_Brain | Stromal_cell | 14459 | 0.0353 | 0.124 | 0.38776 |
| DropViz_all_level2 | GP.Mural_Rgs5Acta2_Rgs5.8_4 | 14805 | 0.00133 | 0.00466 | 0.38808 |
| MouseCellAtlas_all | Lung.Clara_Cell | 14459 | 0.0161 | 0.0567 | 0.38823 |
| DropViz_FC_level2 | Neuron_Slc17a6_Reln.11_2 | 14805 | 9.41E-04 | 0.00332 | 0.38855 |
| MouseCellAtlas_all | Placenta.Megakaryocyte_progenitor_cell | 14459 | 0.0136 | 0.0484 | 0.38902 |
| DropViz_HC_level2 | Ependymal_Ccdc153.11_1 | 14805 | 0.00119 | 0.00422 | 0.3893 |
| MouseCellAtlas_Neonatal_Skin | Erythroblast | 14459 | 0.0336 | 0.12 | 0.38935 |
| DropViz_all_level2 | ENT.Astrocyte_Gja1_Myoc.7_1 | 14805 | 0.00104 | 0.0037 | 0.38936 |
| MouseCellAtlas_Embryo_all | Fetal_Lung.Stromal_cell_Ankfy1_high | 14459 | 0.0435 | 0.155 | 0.38937 |
| DropViz_PC_level2 | Neuron_Slc17a7_Bcl6-Fos.3_5 | 14805 | 0.00265 | 0.00946 | 0.38966 |
| TabulaMuris_droplet_all | Kidney.smooth_muscle_cell | 13888 | 0.00783 | 0.028 | 0.38968 |
| DropViz_all_level1 | PC.Macrophage | 14805 | 0.00126 | 0.00448 | 0.3897 |
| Linnarsson_GSE74672_Mouse_Hypothalamus_Neurons_level2 | Oxytocin_3 | 14550 | 0.0103 | 0.0369 | 0.38972 |
| MouseCellAtlas_Adult_all | Mammary_Gland.Natural_kill_cell | 14459 | 0.0226 | 0.0813 | 0.39067 |
| GSE106678_Mouse_Cortex | Ex27 | 14573 | 0.0579 | 0.209 | 0.3908 |
| Allen_Mouse_ALM_level2 | Vip_Gpc3_3 | 14744 | 0.00319 | 0.0116 | 0.39135 |
| MouseCellAtlas_all | Muscle.B_cell_Jchain_high | 14459 | 0.0184 | 0.0669 | 0.3915 |
| MouseCellAtlas_all | Placenta.Decidual_stromal_cell | 14459 | 0.0141 | 0.0512 | 0.39177 |
| MouseCellAtlas_Neonatal_all | Neonatal_Skin.Neutrophil | 14459 | 0.0149 | 0.0545 | 0.39205 |
| DropViz_PC_level2 | Neuron_Gad1Gad2-Slc17a8_Synpr-Sncg-Yjefn3.4_4 | 14805 | 0.0017 | 0.00621 | 0.3921 |
| MouseCellAtlas_all | Fetal_Brain.Neuron_Kpna2_high | 14459 | 0.0199 | 0.0728 | 0.39217 |
| MouseCellAtlas_Bone_Marrow | Bone_Marrow.T_cell_Ms4a4b_high | 14459 | 0.0216 | 0.0793 | 0.39272 |
| MouseCellAtlas_all | Muscle.Muscle_cell_Tnnc2_high | 14459 | 0.00868 | 0.0319 | 0.39284 |
| MouseCellAtlas_Fetal_Lung | Neutrophil_S100a8_high | 14459 | 0.0344 | 0.127 | 0.39328 |
| TabulaMuris_FACS_all | Spleen.myeloid_cell | 13888 | 0.00282 | 0.0104 | 0.39331 |
| GSE98816_Mouse_Brain_Vascular | capilEC | 14130 | 0.00272 | 0.0101 | 0.39352 |
| GSE93374_Mouse_Arc_ME_neurons | Fam19a2 | 13325 | 0.101 | 0.377 | 0.39404 |
| MouseCellAtlas_Adult_all | Mammary_Gland.Macrophage_Retnla_high | 14459 | 0.0211 | 0.0788 | 0.39422 |
| DropViz_ENT_level2 | Mural_Rgs5Acta2_Kcnj8.3_1 | 14805 | 9.55E-04 | 0.00356 | 0.39435 |
| GSE82187_Mouse_Striatum | Ependy_C | 13103 | 0.00636 | 0.0237 | 0.39435 |
| Allen_Mouse_VISp_level1 | GABA_Vip | 13868 | 0.0042 | 0.0157 | 0.39438 |
| GSE89164_Mouse_Hindbrain | Granule_cells | 12262 | 0.0219 | 0.0818 | 0.39439 |
| MouseCellAtlas_Mammary_Gland | Mammary_Gland_Pregnancy.Myeloid_leukocyte | 14459 | 0.0124 | 0.0464 | 0.39479 |
| GSE99235_Mouse_Lung_Vascular | FB1 | 14559 | 0.00227 | 0.00853 | 0.395 |
| MouseCellAtlas_Adult_all | Lung.AT1_Cell | 14459 | 0.0187 | 0.0709 | 0.39578 |
| MouseCellAtlas_all | Lung.Conventional_dendritic_cell_Mgl2_high | 14459 | 0.0177 | 0.0673 | 0.39632 |
| DropViz_all_level2 | TH.Mural_Rgs5Acta2_Cnn1.5_1 | 14805 | 0.00123 | 0.00469 | 0.39653 |
| Linnarsson_MouseBrainAtlas_level5 | DEINH8 | 15140 | 0.0173 | 0.0658 | 0.39655 |
| MouseCellAtlas_Embryo_all | Embryonic_Mesenchyme.Ganglion_cell_Mapt_high | 14459 | 0.0256 | 0.0976 | 0.39672 |
| DropViz_all_level2 | HC.Oligodendrocyte_Trf_Plin3.8_1 | 14805 | 0.00146 | 0.00557 | 0.39678 |
| MouseCellAtlas_Small_Intestine | S_cell_Chgb_high | 14459 | 0.0109 | 0.0418 | 0.39691 |
| MouseCellAtlas_all | Embryonic_Mesenchyme.Dendritic_Cell | 14459 | 0.0278 | 0.107 | 0.3973 |
| Linnarsson_MouseBrainAtlas_level5 | SZNBL | 15140 | 0.00909 | 0.0351 | 0.39784 |
| MouseCellAtlas_Embryo_all | Embryonic_Mesenchyme.Ganglion_cell_Cartpt_high | 14459 | 0.0252 | 0.0974 | 0.39786 |
| Linnarsson_GSE103840_Mouse_Dorsal_horn | Glut_Elavl4_Meis2 | 14550 | 0.0158 | 0.0613 | 0.398 |
| Linnarsson_MouseBrainAtlas_level5 | TEGLU12 | 15140 | 0.0105 | 0.0405 | 0.39824 |
| MouseCellAtlas_Adult_all | Muscle.Muscle_cell_Tnnc2_high | 14459 | 0.0081 | 0.0317 | 0.39907 |
| Linnarsson_GSE104323_Mouse_Dentate_gyrus | nIPC_perin | 14909 | 0.0108 | 0.0425 | 0.39921 |
| DropViz_HC_level2 | Neuron_Slc17a7_Nxph3-Cplx3-Pappa2.3_9 | 14805 | 0.00154 | 0.00605 | 0.39923 |
| MouseCellAtlas_Bone_Marrow | Bone_Marrow_Mesenchyme.Megakaryocyte | 14459 | 0.0128 | 0.0503 | 0.39936 |
| DropViz_HC_level2 | Neurogenesis_Sox4_Efhd2.13_1 | 14805 | 0.00106 | 0.00417 | 0.39938 |
| MouseCellAtlas_Embryo_all | Placenta.Dendritic_cell | 14459 | 0.0194 | 0.0761 | 0.39951 |
| DropViz_FC_level2 | Macrophage_C1qb_Mrc1.11_3 | 14805 | 0.00104 | 0.00408 | 0.39987 |
| MouseCellAtlas_Adult_all | Mammary_Gland.Macrophage_Apoe_high | 14459 | 0.0139 | 0.0549 | 0.40005 |
| MouseCellAtlas_Liver | Granulocyte | 14459 | 0.028 | 0.111 | 0.40007 |
| GSE93374_Mouse_Arc_ME_level2 | Oligodendro2 | 13325 | 0.00594 | 0.0235 | 0.40026 |
| DropViz_all_level1 | ENT.Mural | 14805 | 0.0013 | 0.00518 | 0.40082 |
| Linnarsson_GSE67602_Mouse_Skin_Epidermis | LH | 14528 | 0.01 | 0.0399 | 0.40095 |
| MouseCellAtlas_Lung | NK_Cell | 14459 | 0.0298 | 0.119 | 0.40101 |
| DropViz_TH_level2 | Neuron_Slc17a6_Rora_C1ql2.2_5 | 14805 | 0.00172 | 0.00685 | 0.40108 |
| MouseCellAtlas_Adult_all | Prostate.Glandular_epithelium | 14459 | 0.0287 | 0.115 | 0.40113 |
| DropViz_all_level2 | FC.Neuron_Slc17a6_Reln.11_2 | 14805 | 8.32E-04 | 0.00333 | 0.40144 |
| MouseCellAtlas_Adult_all | Mammary_Gland.Secretory_alveoli_cell_Fth1.Wfdc3_high | 14459 | 0.0153 | 0.0615 | 0.40171 |
| DroNc_Mouse_Hippocampus | exPFC2 | 12425 | 0.066 | 0.267 | 0.40246 |
| GSE87544_Mouse_Hypothalamus | Tany | 13878 | 0.00992 | 0.0402 | 0.4025 |
| Linnarsson_MouseBrainAtlas_level5 | SCGLU9 | 15140 | 0.0139 | 0.0563 | 0.40254 |
| MouseCellAtlas_Placenta | Megakaryocyte_progenitor_cell | 14459 | 0.0124 | 0.0501 | 0.4027 |
| MouseCellAtlas_all | Pancreas.Dividing_cell | 14459 | 0.0128 | 0.0519 | 0.40278 |
| MouseCellAtlas_Mammary_Gland | Mammary_Gland_Lactation.Secretory_alveoli_cell_Cd63_high | 14459 | 0.0224 | 0.0917 | 0.40344 |
| GSE98816_Mouse_Brain_Vascular | aEC | 14130 | 0.00248 | 0.0102 | 0.40381 |
| DropViz_all_level2 | HC.Neuron_Slc17a7_Fibcd1-Nrip3.5_2 | 14805 | 0.00178 | 0.0073 | 0.40392 |
| DropViz_STR_level2 | Polydendrocye_Tnr_Cspg5.5_1 | 14805 | 0.00146 | 0.00602 | 0.40406 |
| DropViz_PC_level2 | Polydendrocyte_Tnr_Tmsb4x.10_3 | 14805 | 0.00124 | 0.00514 | 0.40435 |
| MouseCellAtlas_Adult_all | Small_Intestine.S_cell_Chgb_high | 14459 | 0.00909 | 0.0378 | 0.40502 |
| MouseCellAtlas_all | Spleen.Neutrophil | 14459 | 0.00989 | 0.0413 | 0.40525 |
| TabulaMuris_FACS_all | Lung.ciliated_cell | 13888 | 0.00111 | 0.00464 | 0.40573 |
| DropViz_all_level2 | PC.Neuron_Slc17a7_Bcl6-Tcerg1l.3_4 | 14805 | 0.00191 | 0.00805 | 0.40602 |
| MouseCellAtlas_all | Lung.AT1_Cell | 14459 | 0.0167 | 0.0704 | 0.40619 |
| DropViz_FC_level2 | Neuron_Slc17a7_Nptxr-Fos.6_5 | 14805 | 0.00216 | 0.00913 | 0.40637 |
| DropViz_all_level1 | GP.Mural | 14805 | 0.0014 | 0.0059 | 0.40656 |
| DropViz_all_level2 | SN.Astrocyte_Gja1_Cst3.7_2 | 14805 | 0.00132 | 0.00559 | 0.40663 |
| DropViz_SN_level2 | Oligodendrocyte_Tfr_Il33.10_4 | 14805 | 0.00151 | 0.0064 | 0.40669 |
| MouseCellAtlas_Adult_all | Spleen.Dendritic_cell_Siglech_high | 14459 | 0.0156 | 0.0666 | 0.40725 |
| TabulaMuris_FACS_Liver | Kupffer_cell | 13888 | 0.00242 | 0.0103 | 0.40725 |
| MouseCellAtlas_Ovary | Cumulus_cell_Nupr1_high | 14459 | 0.0346 | 0.147 | 0.40733 |
| DropViz_all_level2 | HC.Neuron_Slc17a7_Pvrl3-St18.6_3 | 14805 | 0.0017 | 0.00728 | 0.4076 |
| DropViz_SN_level2 | Neuron_Th_Cadm2.4_6 | 14805 | 7.41E-04 | 0.00318 | 0.4077 |
| MouseCellAtlas_Fetal_Brain | Ependymal_cell | 14459 | 0.0218 | 0.095 | 0.40921 |
| GSE106678_Mouse_Cortex | MG | 14573 | 0.021 | 0.0914 | 0.40934 |
| DropViz_all_level1 | GP.Macrophage | 14805 | 0.00103 | 0.00452 | 0.40959 |
| MouseCellAtlas_Adult_all | Pancreas.Dividing_cell | 14459 | 0.0115 | 0.0506 | 0.40987 |
| Linnarsson_GSE59739_Mouse_Dorsal_root_ganglion_level3 | Peptidergic_nociceptor2 | 13848 | 0.00272 | 0.012 | 0.4099 |
| MouseCellAtlas_Ovary | Granulosa_cell_Inhba_high | 14459 | 0.0278 | 0.122 | 0.4099 |
| DropViz_GP_level2 | Neuron_Gad1Gad2_Hpcal4.2_4 | 14805 | 0.0018 | 0.00793 | 0.41029 |
| TabulaMuris_FACS_Lung | monocyte | 13888 | 0.00174 | 0.00769 | 0.41051 |
| GSE89164_Mouse_Hindbrain | SC | 12262 | 0.0279 | 0.123 | 0.41067 |
| DropViz_all_level2 | SN.Neuron_Gad1Gad2_Rln3.3_5 | 14805 | 9.67E-04 | 0.00428 | 0.4107 |
| DropViz_all_level2 | FC.Macrophage_C1qb_Mrc1.11_3 | 14805 | 9.32E-04 | 0.00414 | 0.41103 |
| DropViz_all_level2 | TH.Neuron_Slc17a7_Tac2_Chat.1_1 | 14805 | 0.00126 | 0.00563 | 0.41109 |
| MouseCellAtlas_Muscle | Granulocyte_monocyte_progenitor_cell | 14459 | 0.0153 | 0.068 | 0.41124 |
| MouseCellAtlas_Spleen | Plasma_cell | 14459 | 0.0132 | 0.0592 | 0.41161 |
| Linnarsson_GSE59739_Mouse_Dorsal_root_ganglion_level2 | Neurofilament_containing1 | 13848 | 0.00272 | 0.0122 | 0.4121 |
| Linnarsson_GSE75330_Mouse_Oligodendrocytes | PPR | 14541 | 0.00853 | 0.0384 | 0.41212 |
| MouseCellAtlas_Adult_all | Mammary_Gland.Secretory_alveoli_cell_Igkv1.110.P4hb_high | 14459 | 0.0144 | 0.0651 | 0.41227 |
| GSE67835_Human_Cortex_woFetal | hybrid | 16585 | 0.00265 | 0.012 | 0.41236 |
| DropViz_all_level2 | GP.Neuron_Gad1Gad2_Drd1-Nefm.3_1 | 14805 | 0.00159 | 0.0072 | 0.4125 |
| MouseCellAtlas_all | Brain.Neuron | 14459 | 0.0205 | 0.093 | 0.41255 |
| GSE89164_Mouse_Hindbrain | Endothelial_progenitors | 12262 | 0.0175 | 0.079 | 0.41261 |
| MouseCellAtlas_Adult_all | Kidney.S1_proximal_tubule_cells | 14459 | 0.0111 | 0.0505 | 0.41276 |
| Linnarsson_GSE74672_Mouse_Hypothalamus_Neurons_level2 | Vglut2_9_Gpr149 | 14550 | 0.00766 | 0.0348 | 0.41293 |
| MouseCellAtlas_all | Fetal_Intestine.Enterocyte_progenitor_late_Ccnb1_high | 14459 | 0.0208 | 0.0946 | 0.41305 |
| MouseCellAtlas_all | Mammary_Gland.Secretory_alveoli_cell_Crip1_high | 14459 | 0.0122 | 0.0556 | 0.41318 |
| DropViz_all_level2 | HC.Macrophage_C1qb_Mrc1.10_2 | 14805 | 7.52E-04 | 0.00343 | 0.41339 |
| MouseCellAtlas_Adult_all | Brain.Neuron | 14459 | 0.0202 | 0.0928 | 0.41364 |
| TabulaMuris_droplet_Spleen | T_cell | 13888 | 0.0169 | 0.0777 | 0.41372 |
| GSE93374_Mouse_Arc_ME_neurons | Th_Lef1 | 13325 | 0.09 | 0.413 | 0.41377 |
| MouseCellAtlas_Kidney | Ureteric_epithelium | 14459 | 0.0117 | 0.0542 | 0.41442 |
| DropViz_all_level2 | PC.Neuron_Gad1Gad2_Sst-Nr2f2.5_7 | 14805 | 0.00108 | 0.005 | 0.41472 |
| MouseCellAtlas_all | Mammary_Gland.Macrophage_Retnla_high | 14459 | 0.0165 | 0.0766 | 0.41478 |
| Linnarsson_GSE60361_Mouse_Cortex_Hippocampus_level2 | Int16 | 14010 | 0.00564 | 0.0262 | 0.4149 |
| MouseCellAtlas_all | Small_Intestine.S_cell_Chgb_high | 14459 | 0.00807 | 0.0375 | 0.41495 |
| MouseCellAtlas_Mammary_Gland | Mammary_Gland_Involution.Mast_cell | 14459 | 0.0104 | 0.0484 | 0.41497 |
| MouseCellAtlas_all | Prostate.Glandular_epithelium | 14459 | 0.0242 | 0.113 | 0.41512 |
| MouseCellAtlas_Adult_all | Uterus.B_cell | 14459 | 0.00884 | 0.0416 | 0.41594 |
| MouseCellAtlas_Adult_all | Brain.Granulocyte_Ngp_high | 14459 | 0.00555 | 0.0262 | 0.41614 |
| TabulaMuris_FACS_all | Fat.granulocyte | 13888 | 0.00205 | 0.00972 | 0.41633 |
| MouseCellAtlas_Adult_all | Mammary_Gland.Muscel_cell | 14459 | 0.0133 | 0.0631 | 0.41661 |
| TabulaMuris_droplet_all | Lung.monocyte | 13888 | 0.00671 | 0.0319 | 0.41664 |
| Linnarsson_GSE103840_Mouse_Dorsal_horn | GABA_Cplx1_Adamts5 | 14550 | 0.0164 | 0.0778 | 0.41668 |
| MouseCellAtlas_Adult_all | Kidney.Proximal_tubule_brush_border_cell | 14459 | 0.0105 | 0.0497 | 0.4167 |
| DropViz_all_level2 | SN.Polydendrocyte_Tnr_Cspg5.6_1 | 14805 | 6.88E-04 | 0.00327 | 0.41673 |
| DropViz_all_level2 | GP.Mural_Rgs5Acta2_Kcnj8.8_2 | 14805 | 8.06E-04 | 0.00384 | 0.41698 |
| MouseCellAtlas_all | Placenta.Progenitor_trophoblast_Gjb3_high | 14459 | 0.0139 | 0.0666 | 0.41726 |
| MouseCellAtlas_all | Mammary_Gland.Secretory_alveoli_cell_Ehf_high | 14459 | 0.0127 | 0.061 | 0.41741 |
| MouseCellAtlas_Placenta | PE_lineage_cell_S100g_high | 14459 | 0.0211 | 0.101 | 0.41743 |
| Linnarsson_GSE60361_Mouse_Cortex_Hippocampus_level2 | Pvm2 | 14010 | 0.00619 | 0.0297 | 0.41751 |
| MouseCellAtlas_all | Kidney.Proximal_tubule_brush_border_cell | 14459 | 0.0104 | 0.05 | 0.41776 |
| MouseCellAtlas_Neonatal_Rib | Granulocyte | 14459 | 0.0186 | 0.0901 | 0.41809 |
| GSE87544_Mouse_Hypothalamus | GABA13 | 13878 | 0.0184 | 0.0894 | 0.41849 |
| MouseCellAtlas_all | Neonatal_Heart.Neutrophil_Retnlg_high | 14459 | 0.0125 | 0.0608 | 0.41852 |
| Linnarsson_GSE76381_Mouse_Midbrain | Epen | 14550 | 0.00518 | 0.0252 | 0.4187 |
| DroNc_Mouse_Hippocampus | exPFC1 | 12425 | 0.0515 | 0.252 | 0.41887 |
| MouseCellAtlas_Fetal_Liver | Mast_cell_Mcpt8_high | 14459 | 0.0195 | 0.0953 | 0.41901 |
| MouseCellAtlas_Embryo_all | Placenta.Megakaryocyte_progenitor_cell | 14459 | 0.00963 | 0.0474 | 0.41952 |
| Linnarsson_GSE76381_Mouse_Midbrain | NProg | 14550 | 0.00793 | 0.0392 | 0.41981 |
| TabulaMuris_FACS_Pancreas | pancreatic_ductal_cell | 13888 | 0.00165 | 0.00818 | 0.41995 |
| MouseCellAtlas_Adult_all | Kidney.Ureteric_epithelium | 14459 | 0.0114 | 0.0566 | 0.42008 |
| TabulaMuris_droplet_Lung | B_cell | 13888 | 0.00916 | 0.0454 | 0.42008 |
| MouseCellAtlas_all | Fetal_Lung.Epithelial_cell_Sftpc_high | 14459 | 0.0186 | 0.0932 | 0.42076 |
| DropViz_PC_level2 | Oligodendrocyte_Tfr_Klk6.9_3 | 14805 | 0.00101 | 0.00504 | 0.42087 |
| DropViz_FC_level2 | Mural_Rgs5Acta2_Cnn1.13_1 | 14805 | 9.70E-04 | 0.00486 | 0.42093 |
| Linnarsson_MouseBrainAtlas_level5 | MOL1 | 15140 | 0.00558 | 0.028 | 0.42101 |
| MouseCellAtlas_all | Kidney.S1_proximal_tubule_cells | 14459 | 0.00996 | 0.0502 | 0.42139 |
| MouseCellAtlas_Fetal_Liver | Neutrophil_Elane_high | 14459 | 0.0205 | 0.104 | 0.42207 |
| MouseCellAtlas_Bone_Marrow | Bone_Marrow.Hematopoietic_stem_progenitor_cell | 14459 | 0.0108 | 0.0553 | 0.42233 |
| TabulaMuris_FACS_all | Fat.neutrophil | 13888 | 0.0015 | 0.00777 | 0.42341 |
| MouseCellAtlas_Mammary_Gland | Mammary_Gland_Lactation.Secretory_alveoli_cell_Cd83_high | 14459 | 0.0161 | 0.0832 | 0.42342 |
| MouseCellAtlas_Muscle | Macrophage_Retnla_high | 14459 | 0.0111 | 0.0574 | 0.42345 |
| DropViz_all_level2 | PC.Neuron_Sc17a7_Calb1-Otof.2_6 | 14805 | 0.00151 | 0.00784 | 0.42349 |
| DropViz_all_level2 | PC.Polydendrocyte_Tnr_Tmsb4x.10_3 | 14805 | 0.00109 | 0.00564 | 0.42354 |
| DropViz_all_level2 | FC.Neuron_Slc17a7_Parm1_Tshz2.7_7 | 14805 | 0.00133 | 0.00693 | 0.42372 |
| Linnarsson_GSE76381_Human_Midbrain | RN | 14835 | 0.0119 | 0.0616 | 0.42372 |
| Linnarsson_GSE103840_Mouse_Dorsal_horn | GABA_Gal_Pnoc | 14550 | 0.0147 | 0.0763 | 0.42379 |
| MouseCellAtlas_all | Mammary_Gland.Macrophage_Apoe_high | 14459 | 0.0103 | 0.0534 | 0.42382 |
| MouseCellAtlas_Embryo_all | Fetal_Stomache.Neuron | 14459 | 0.0243 | 0.127 | 0.42414 |
| DropViz_HC_level2 | Mural_Rgs5Acta2_Cnn1.16_1 | 14805 | 8.52E-04 | 0.00447 | 0.42443 |
| MouseCellAtlas_Placenta | Spiral_artery_trophoblast_giant_cells | 14459 | 0.0148 | 0.0781 | 0.42495 |
| MouseCellAtlas_all | Mammary_Gland.Natural_kill_cell | 14459 | 0.0148 | 0.0784 | 0.42527 |
| MouseCellAtlas_Embryo_all | Embryonic_Mesenchyme.Dendritic_Cell | 14459 | 0.0214 | 0.114 | 0.42534 |
| MouseCellAtlas_all | Fetal_Stomache.Epithelial_cell | 14459 | 0.0215 | 0.115 | 0.42565 |
| DropViz_PC_level2 | Mural_Acta2Rgs5Rgs5.13_3 | 14805 | 7.93E-04 | 0.00427 | 0.42623 |
| MouseCellAtlas_Adult_all | Lung.Nuocyte | 14459 | 0.0161 | 0.0868 | 0.42624 |
| MouseCellAtlas_Adult_all | Peripheral_Blood.T_cell_Gm14303_high | 14459 | 0.0131 | 0.0708 | 0.42637 |
| MouseCellAtlas_all | Kidney.Ureteric_epithelium | 14459 | 0.0105 | 0.0567 | 0.4264 |
| MouseCellAtlas_all | Peripheral_Blood.T_cell_Gm14303_high | 14459 | 0.0133 | 0.0719 | 0.42662 |
| GSE104276_Human_Prefrontal_cortex_all_ages | Microglia | 17199 | 0.00166 | 0.00904 | 0.42731 |
| Linnarsson_GSE76381_Human_Midbrain | Sert | 14835 | 0.00569 | 0.0313 | 0.42791 |
| TabulaMuris_FACS_all | Spleen.B_cell | 13888 | 0.00175 | 0.00972 | 0.42861 |
| TabulaMuris_FACS_Heart | leukocyte | 13888 | 0.0015 | 0.00832 | 0.42867 |
| DropViz_all_level1 | ENT.Polydendrocyte | 14805 | 0.00125 | 0.00697 | 0.42873 |
| DropViz_PC_level2 | Endothelial_Tip_Dcn_Mgp-Clu.14_2 | 14805 | 7.62E-04 | 0.00426 | 0.42892 |
| MouseCellAtlas_Placenta | Granulocyte_Neat1_high | 14459 | 0.00634 | 0.0359 | 0.42993 |
| MouseCellAtlas_Adult_all | Mammary_Gland.T_cell_Ly6c2_high | 14459 | 0.0132 | 0.0749 | 0.43006 |
| MouseCellAtlas_Testis | Erythroblast_Hbb.bs_high | 14459 | 0.0169 | 0.0962 | 0.43012 |
| DropViz_all_level2 | ENT.Mural_Rgs5Acta2_Ccnd1.3_4 | 14805 | 6.48E-04 | 0.00369 | 0.43035 |
| MouseCellAtlas_all | Mammary_Gland.T_cell_Ly6c2_high | 14459 | 0.0134 | 0.0766 | 0.43035 |
| MouseCellAtlas_all | Lung.Nuocyte | 14459 | 0.0153 | 0.0876 | 0.4305 |
| MouseCellAtlas_all | Mammary_Gland.Secretory_alveoli_cell_Fth1.Wfdc3_high | 14459 | 0.0104 | 0.0599 | 0.43088 |
| DropViz_all_level2 | FC.Mural_Rgs5Acta2_Cnn1.13_1 | 14805 | 8.82E-04 | 0.00507 | 0.43098 |
| GSE93374_Mouse_Arc_ME_level2 | NG2_OPC2 | 13325 | 0.0192 | 0.111 | 0.43113 |
| GSE87544_Mouse_Hypothalamus | Glu10 | 13878 | 0.0101 | 0.0586 | 0.43183 |
| GSE104276_Human_Prefrontal_cortex_per_ages | GW12_Neurons | 17199 | 0.00195 | 0.0114 | 0.43189 |
| MouseCellAtlas_all | Brain.Granulocyte_Ngp_high | 14459 | 0.00444 | 0.026 | 0.43215 |
| DropViz_all_level2 | GP.Neuron_Gad1Gad2_Sphkap-Tmem255a.3_7 | 14805 | 0.00133 | 0.0078 | 0.43236 |
| GSE93374_Mouse_Arc_ME_level2 | Pars_Tuber1A | 13325 | 0.00533 | 0.0313 | 0.43243 |
| TabulaMuris_droplet_all | Lung.B_cell | 13888 | 0.00802 | 0.0473 | 0.43262 |
| DropViz_SN_level2 | Mural_Rgs5Acta2_Fos.13_5 | 14805 | 6.15E-04 | 0.00365 | 0.43301 |
| MouseCellAtlas_Embryo_all | Embryonic_Mesenchyme.Muscle | 14459 | 0.0204 | 0.121 | 0.43306 |
| MouseCellAtlas_Adult_all | Mammary_Gland.Dendritic_cell_Fscn1_high | 14459 | 0.011 | 0.0651 | 0.43317 |
| GSE93374_Mouse_Arc_ME_neurons | Rgs16_Slc17a6 | 13325 | 0.0489 | 0.291 | 0.43339 |
| MouseCellAtlas_all | Neonatal_Muscle.Stromal_cell_Spp1_high | 14459 | 0.00777 | 0.0466 | 0.43371 |
| MouseCellAtlas_all | Spleen.Dendritic_cell_Siglech_high | 14459 | 0.0107 | 0.0641 | 0.43395 |
| MouseCellAtlas_Adult_all | Liver.Granulocyte | 14459 | 0.0116 | 0.0698 | 0.43398 |
| DropViz_STR_level2 | Neurogenesis_Sox4_Ung.2_1 | 14805 | 8.25E-04 | 0.00499 | 0.43431 |
| Allen_Mouse_VISp_level2 | L4_Ctxn3 | 13868 | 0.00249 | 0.0151 | 0.43433 |
| GSE104276_Human_Prefrontal_cortex_per_ages | GW16_Stem_cells | 17199 | 0.00214 | 0.0129 | 0.43438 |
| TabulaMuris_droplet_all | Muscle.skeletal_muscle_satellite_cell | 13888 | 0.00661 | 0.04 | 0.43444 |
| MouseCellAtlas_all | Uterus.B_cell | 14459 | 0.0067 | 0.0408 | 0.43468 |
| TabulaMuris_FACS_Pancreas | leukocyte | 13888 | 0.00116 | 0.00708 | 0.43468 |
| MouseCellAtlas_Placenta | Decidual_stromal_cell | 14459 | 0.00895 | 0.0546 | 0.43485 |
| MouseCellAtlas_all | Neonatal_Skin.Macrophage_Lyz2_high | 14459 | 0.0106 | 0.0646 | 0.43517 |
| MouseCellAtlas_Neonatal_all | Neonatal_Skin.Mast_cell | 14459 | 0.0105 | 0.0648 | 0.4357 |
| DropViz_all_level2 | HC.Gad1Gad2_Htr3a_Nnat.1_16 | 14805 | 9.17E-04 | 0.00567 | 0.43579 |
| DropViz_all_level2 | PC.Oligodendrocyte_Tfr_Klk6.9_3 | 14805 | 9.06E-04 | 0.00561 | 0.43586 |
| DropViz_all_level2 | SN.Neuron_Slc17a6-Gad2-Th_Crhbp.3_7 | 14805 | 0.00105 | 0.00652 | 0.43591 |
| DropViz_all_level2 | TH.Ependymal_Ccdc153.10_1 | 14805 | 6.89E-04 | 0.00431 | 0.43655 |
| MouseCellAtlas_Mammary_Gland | Mammary_Gland_Virgin.B_cell_Cd79a.Fcer2a_high | 14459 | 0.00943 | 0.0591 | 0.43665 |
| Linnarsson_MouseBrainAtlas_level5 | HBINH1 | 15140 | 0.00771 | 0.0486 | 0.43698 |
| MouseCellAtlas_Lung | Alveolar_bipotent_progenitor | 14459 | 0.00907 | 0.0573 | 0.43712 |
| DropViz_all_level2 | CB.Neuron_Gad1Gad2_Pvalb-Chrm2.3_4 | 14805 | 7.63E-04 | 0.00484 | 0.43735 |
| GSE93374_Mouse_Arc_ME_level1 | ParsTuber1 | 13325 | 0.00981 | 0.0623 | 0.43745 |
| MouseCellAtlas_all | Placenta.Granulocyte_Neat1_high | 14459 | 0.00544 | 0.0347 | 0.4377 |
| MouseCellAtlas_Bone_Marrow | Bone_Marrow.Macrophage_Ms4a6c_high | 14459 | 0.00759 | 0.0487 | 0.43807 |
| MouseCellAtlas_Embryo_all | Fetal_Brain.Radial_glia_Vim_high | 14459 | 0.0214 | 0.138 | 0.43819 |
| MouseCellAtlas_Adult_all | Kidney.Proximal_tubule_cell_Osgin1_high | 14459 | 0.00845 | 0.0546 | 0.43849 |
| MouseCellAtlas_Embryonic_Mesenchyme | Ganglion_cell_Mapt_high | 14459 | 0.0168 | 0.109 | 0.43864 |
| DropViz_STR_level2 | Mural_Rgs5Acta2_Cnn1.8_2 | 14805 | 5.54E-04 | 0.00361 | 0.43903 |
| Linnarsson_MouseBrainAtlas_level5 | ABC | 15140 | 0.00396 | 0.0259 | 0.43929 |
| MouseCellAtlas_Adult_all | Mammary_Gland.Secretory_alveoli_cell_Fcer1g_high | 14459 | 0.00806 | 0.0532 | 0.43976 |
| Linnarsson_MouseBrainAtlas_level6_rank1 | Glia | 15140 | 0.0075 | 0.0498 | 0.44015 |
| TabulaMuris_FACS_all | Brain_Microglia.macrophage | 13888 | 0.00136 | 0.00903 | 0.44035 |
| GSE106678_Mouse_Cortex | OPC | 14573 | 0.0143 | 0.0955 | 0.44059 |
| MouseCellAtlas_Bone_Marrow | Bone_Marrow_Mesenchyme.Basophils | 14459 | 0.0158 | 0.106 | 0.441 |
| MouseCellAtlas_Mammary_Gland | Mammary_Gland_Lactation.Secretory_alveoli_cell_Ptpn18_high | 14459 | 0.0146 | 0.0987 | 0.44133 |
| MouseCellAtlas_Stomach | Stomach_cell_Muc5ac_high | 14459 | 0.0117 | 0.0794 | 0.44144 |
| MouseCellAtlas_Ovary | luteal_cells | 14459 | 0.0093 | 0.0632 | 0.44148 |
| DropViz_all_level2 | PC.Neuron_Gad1Gad2-Slc17a8_Synpr-Sncg-Yjefn3.4_4 | 14805 | 8.76E-04 | 0.00595 | 0.4415 |
| GSE87544_Mouse_Hypothalamus | Astro | 13878 | 0.00618 | 0.0422 | 0.44174 |
| Linnarsson_GSE101601_Mouse_Somatosensory_cortex | Red_blood_cell | 14550 | 0.0149 | 0.102 | 0.44203 |
| DroNc_Mouse_Hippocampus | ODC | 12425 | 0.0162 | 0.111 | 0.44208 |
| TabulaMuris_FACS_all | Bladder.basal_cell_of_urothelium | 13888 | 7.58E-04 | 0.00527 | 0.44279 |
| MouseCellAtlas_all | Mammary_Gland.Secretory_alveoli_cell_Igkv1.110.P4hb_high | 14459 | 0.00913 | 0.0634 | 0.44281 |
| DropViz_FC_level2 | Oligodendrocyte_Tnf_Ptgds.9_4 | 14805 | 8.94E-04 | 0.00623 | 0.44299 |
| DropViz_all_level2 | PC.Mural_Acta2Rgs5Rgs5.13_3 | 14805 | 6.39E-04 | 0.0045 | 0.44353 |
| MouseCellAtlas_all | Kidney.Proximal_tubule_cell_Osgin1_high | 14459 | 0.00774 | 0.0546 | 0.44364 |
| GSE98816_Mouse_Brain_Vascular | EC2 | 14130 | 0.00158 | 0.0111 | 0.44378 |
| DropViz_all_level2 | CB.Oligodendroyte_Trf_Il33.6_1 | 14805 | 6.74E-04 | 0.00483 | 0.44456 |
| MouseCellAtlas_all | Fetal_Brain.Schwann_cell | 14459 | 0.0103 | 0.074 | 0.44462 |
| MouseCellAtlas_Adult_all | Mammary_Gland.Secretory_alveoli_cell_Ighm1_high | 14459 | 0.0108 | 0.0774 | 0.44468 |
| MouseCellAtlas_Fetal_Stomache | Endocrine_cell | 14459 | 0.0167 | 0.121 | 0.44499 |
| DropViz_all_level2 | CB.Neuron_Slc17a6_Nrgn.4_3 | 14805 | 5.53E-04 | 0.004 | 0.44507 |
| Linnarsson_GSE74672_Mouse_Hypothalamus_Neurons_level2 | Adcyap1_1_Tac1 | 14550 | 0.00574 | 0.0416 | 0.44507 |
| MouseCellAtlas_Brain | Microglia | 14459 | 0.00886 | 0.0644 | 0.44524 |
| MouseCellAtlas_Embryo_all | Fetal_Intestine.Erythroblast_Hbb.y_high | 14459 | 0.011 | 0.0801 | 0.44541 |
| DroNc_Human_Hippocampus | exPFC2 | 16914 | 0.0191 | 0.139 | 0.44545 |
| MouseCellAtlas_Neonatal_Muscle | Smooth_muscle_cell_Mylk_high | 14459 | 0.0101 | 0.074 | 0.44556 |
| Linnarsson_GSE95315_Mouse_Dentate_gyrus | OPC | 11815 | 0.0098 | 0.0717 | 0.44565 |
| DropViz_GP_level2 | Astrocyte_Gja1_Gfap.5_1 | 14805 | 8.11E-04 | 0.00603 | 0.44648 |
| MouseCellAtlas_all | Neonatal_Muscle.Macrophage_Lyz2_high | 14459 | 0.0117 | 0.0886 | 0.4473 |
| MouseCellAtlas_Adult_all | Thymus.DPT_cell | 14459 | 0.0128 | 0.0972 | 0.44752 |
| MouseCellAtlas_Kidney | Stromal_cell_Ankrd1_high | 14459 | 0.00857 | 0.065 | 0.44757 |
| Linnarsson_MouseBrainAtlas_level5 | TEGLU5 | 15140 | 0.00467 | 0.0354 | 0.44761 |
| Linnarsson_GSE76381_Mouse_Midbrain | NbML2 | 14550 | 0.0102 | 0.0777 | 0.44779 |
| DropViz_all_level2 | HC.Endothelial_Tip_Dcn_Rbp4.17_1 | 14805 | 5.78E-04 | 0.00442 | 0.44806 |
| MouseCellAtlas_all | Mammary_Gland.Muscel_cell | 14459 | 0.00799 | 0.0614 | 0.4482 |
| DropViz_CB_level2 | Mural_Acta2Rgs5_Kcnj8.11_4 | 14805 | 3.84E-04 | 0.00297 | 0.44849 |
| MouseCellAtlas_Bone_Marrow | Bone_Marrow_Mesenchyme.Eosinophils | 14459 | 0.0092 | 0.0713 | 0.44863 |
| MouseCellAtlas_all | Thymus.DPT_cell | 14459 | 0.0127 | 0.0984 | 0.44867 |
| Linnarsson_GSE74672_Mouse_Hypothalamus_Neurons_level2 | Adcyap1_2 | 14550 | 0.00619 | 0.0482 | 0.44891 |
| DropViz_all_level1 | CB.Polydendrocyte | 14805 | 4.07E-04 | 0.0032 | 0.44937 |
| DropViz_HC_level2 | Neurogenesis_Sox4_Hist1h2al.13_2 | 14805 | 5.39E-04 | 0.00429 | 0.45001 |
| Linnarsson_GSE59739_Mouse_Dorsal_root_ganglion_level1 | Non_peptidergic_nocieptor | 13848 | 0.00202 | 0.0161 | 0.45003 |
| MouseCellAtlas_Fetal_Stomache | Acinar_cell_Spp1_high | 14459 | 0.02 | 0.16 | 0.45028 |
| DropViz_SN_level2 | Astrocyte_Gja1_Myoc.7_1 | 14805 | 5.96E-04 | 0.00479 | 0.45048 |
| TabulaMuris_FACS_all | Liver.B_cell | 13888 | 0.00109 | 0.0088 | 0.45054 |
| DropViz_GP_level2 | Oligodendrocyte_Trf_Il33.10_5 | 14805 | 7.53E-04 | 0.00619 | 0.45161 |
| DropViz_all_level2 | PC.Endothelial_Tip_Dcn_Mgp-Clu.14_2 | 14805 | 5.30E-04 | 0.00436 | 0.45161 |
| Linnarsson_GSE95315_Mouse_Dentate_gyrus | OL | 11815 | 0.00444 | 0.0368 | 0.45193 |
| DropViz_HC_level2 | Gad1Gad2_Cplx3_Tox.1_12 | 14805 | 8.66E-04 | 0.0072 | 0.45213 |
| MouseCellAtlas_all | Neonatal_Heart.Smooth_muscle_cell | 14459 | 0.00837 | 0.0702 | 0.4526 |
| MouseCellAtlas_Bone_Marrow | Bone_Marrow.Neutrophil_Ngp_high | 14459 | 0.00551 | 0.047 | 0.45327 |
| MouseCellAtlas_Adult_all | Testis.Spermatogonia_Tbc1d23_high | 14459 | 0.00254 | 0.0217 | 0.45334 |
| MouseCellAtlas_Adult_all | Uterus.Dendritic_cell | 14459 | 0.0109 | 0.093 | 0.45348 |
| MouseCellAtlas_all | Embryonic_Mesenchyme.Ganglion_cell_Mapt_high | 14459 | 0.00966 | 0.0831 | 0.45372 |
| MouseCellAtlas_all | Fetal_Lung.Stromal_cell_Ankfy1_high | 14459 | 0.012 | 0.103 | 0.45377 |
| MouseCellAtlas_all | Mammary_Gland.Dendritic_cell_Fscn1_high | 14459 | 0.00726 | 0.0632 | 0.45429 |
| MouseCellAtlas_Adult_all | Peripheral_Blood.NK_cell_Gzma_high | 14459 | 0.0107 | 0.0936 | 0.45431 |
| GSE100597_Mouse_Embryo | E6.5 | 13879 | 0.00132 | 0.0116 | 0.4544 |
| MouseCellAtlas_Adult_all | Mammary_Gland.Secretory_alveoli_cell_Igfbp7_high | 14459 | 0.00716 | 0.0632 | 0.45488 |
| MouseCellAtlas_Embryo_all | Placenta.Decidual_stromal_cell | 14459 | 0.00533 | 0.0474 | 0.45523 |
| MouseCellAtlas_Mammary_Gland | Mammary_Gland_Lactation.Secretory_alveoli_cell_Sh3bgrl3_high | 14459 | 0.00965 | 0.0858 | 0.45524 |
| DropViz_all_level2 | HC.Neuron_Slc17a7_FIbcd1-Inhba.5_7 | 14805 | 8.43E-04 | 0.00755 | 0.45551 |
| GSE106678_Mouse_Cortex | Ex25 | 14573 | 0.0182 | 0.163 | 0.45555 |
| DropViz_all_level1 | FC.Microglia | 14805 | 5.12E-04 | 0.00462 | 0.45581 |
| MouseCellAtlas_Adult_all | Mammary_Gland.Secretory_alveoli_cell_Rhob_high | 14459 | 0.00735 | 0.0665 | 0.45597 |
| DropViz_HC_level2 | Neuron_Gad1Gad2_Sst_Atp2b4.1_3 | 14805 | 5.23E-04 | 0.00474 | 0.45606 |
| DropViz_all_level2 | FC.Oligodendrocyte_Tnf_Ptgds.9_4 | 14805 | 7.46E-04 | 0.00678 | 0.4562 |
| MouseCellAtlas_Mammary_Gland | Mammary_Gland_Involution.Dendritic_cell_Ccl22_high | 14459 | 0.00586 | 0.0535 | 0.45635 |
| Linnarsson_MouseBrainAtlas_level5 | PSNF3 | 15140 | 0.00218 | 0.0199 | 0.45637 |
| MouseCellAtlas_Bone_Marrow | Bone_Marrow_c_kit.Neutrophil_Ngp_high | 14459 | 0.0069 | 0.0631 | 0.4565 |
| MouseCellAtlas_Embryonic_Stem_Cell | ES_Actb_high | 14459 | 0.0226 | 0.207 | 0.45658 |
| DroNc_Mouse_Hippocampus | GABA2 | 12425 | 0.0254 | 0.233 | 0.45665 |
| MouseCellAtlas_Adult_all | Brain.Macrophage_Klf2_high | 14459 | 0.00656 | 0.061 | 0.45718 |
| DropViz_all_level2 | HC.Ependymal_Ccdc153.11_1 | 14805 | 4.67E-04 | 0.00434 | 0.45722 |
| GSE67835_Human_Cortex | endothelial | 16585 | 8.86E-04 | 0.00825 | 0.45725 |
| MouseCellAtlas_all | Testis.Spermatogonia_Tbc1d23_high | 14459 | 0.00232 | 0.0217 | 0.45726 |
| Allen_Mouse_ALM_level2 | L5_ALM_Slc17a8 | 14744 | 4.54E-04 | 0.00423 | 0.45728 |
| Linnarsson_GSE60361_Mouse_Cortex_Hippocampus_level2 | S1PyrL6b | 14010 | 0.00322 | 0.03 | 0.45735 |
| DropViz_FC_level2 | Mural_Rgs5Acta2_Ccnd1.13_2 | 14805 | 5.17E-04 | 0.00489 | 0.45793 |
| Linnarsson_MouseBrainAtlas_level5 | HBADR | 15140 | 0.00346 | 0.0329 | 0.45817 |
| GSE93374_Mouse_Arc_ME_level2 | NG2_OPC1 | 13325 | 0.00555 | 0.0529 | 0.45825 |
| Allen_Mouse_ALM_level2 | L2.3_ALM_Cdh13 | 14744 | 9.89E-04 | 0.00951 | 0.45859 |
| MouseCellAtlas_Small_Intestine | B_cell_Ighd_high | 14459 | 0.00841 | 0.0809 | 0.45859 |
| Linnarsson_GSE59739_Mouse_Dorsal_root_ganglion_level2 | Peptidergic_nociceptor2 | 13848 | 0.00127 | 0.0123 | 0.45875 |
| TabulaMuris_FACS_Fat | granulocyte | 13888 | 0.00116 | 0.0115 | 0.45965 |
| DropViz_all_level2 | CB.Mural_Acta2Rgs5_Kcnj8.11_4 | 14805 | 2.69E-04 | 0.00268 | 0.45999 |
| DropViz_all_level2 | GP.Oligodendrocyte_Trf_Klk6.10_2 | 14805 | 5.53E-04 | 0.00552 | 0.46006 |
| GSE93374_Mouse_Arc_ME_neurons | Slc17a6_Trhr | 13325 | 0.0482 | 0.482 | 0.46018 |
| MouseCellAtlas_Fetal_Lung | Erythroblast_Hbb.bs_high | 14459 | 0.0191 | 0.192 | 0.46023 |
| MouseCellAtlas_Mammary_Gland | Mammary_Gland_Involution.Dendritic_cell_Siglech_high | 14459 | 0.00534 | 0.0538 | 0.46049 |
| MouseCellAtlas_Mammary_Gland | Mammary_Gland_Lactation.Secretory_alveoli_cell_Pa2g4_high | 14459 | 0.00989 | 0.1 | 0.46069 |
| Allen_Mouse_VISp_level2 | Ndnf_Car4 | 13868 | 0.00144 | 0.0149 | 0.46134 |
| DropViz_all_level2 | FC.Neuron_Slc17a7_Nptxr-Fos.6_5 | 14805 | 7.42E-04 | 0.00765 | 0.46138 |
| Linnarsson_GSE67602_Mouse_Skin_Epidermis | IFE_KI | 14528 | 0.00411 | 0.0428 | 0.46172 |
| GSE92332_Mouse_Epithelium_droplet | Enterocyte_Mature_Proximal | 11993 | 0.00274 | 0.0286 | 0.46184 |
| DropViz_all_level2 | SN.Mural_Rgs5Acta2_Cd9.13_3 | 14805 | 4.30E-04 | 0.00451 | 0.46206 |
| TabulaMuris_FACS_Lung | ciliated_cell | 13888 | 4.77E-04 | 0.00505 | 0.46239 |
| MouseCellAtlas_Adult_all | Bladder.Epithelial_cell_Upk3a_high | 14459 | 0.00518 | 0.0553 | 0.46268 |
| MouseCellAtlas_Mammary_Gland | Mammary_Gland_Lactation.T_cell | 14459 | 0.0169 | 0.181 | 0.46269 |
| MouseCellAtlas_all | Embryonic_Mesenchyme.Ganglion_cell_Cartpt_high | 14459 | 0.0073 | 0.078 | 0.46271 |
| MouseCellAtlas_all | Neonatal_Rib.Neuron_Stmn2_high | 14459 | 0.00543 | 0.0586 | 0.46309 |
| MouseCellAtlas_Mammary_Gland | Mammary_Gland_Virgin.B_cell_Jchain_high | 14459 | 0.00396 | 0.0428 | 0.46311 |
| MouseCellAtlas_all | Liver.Granulocyte | 14459 | 0.00621 | 0.0672 | 0.46322 |
| DropViz_HC_level2 | Gad1Gad2_Htr3a_Efba5.1_19 | 14805 | 5.80E-04 | 0.00628 | 0.46323 |
| DropViz_CB_level2 | Oligodendroyte_Trf_Il33.6_1 | 14805 | 4.81E-04 | 0.00528 | 0.46367 |
| GSE99235_Mouse_Lung_Vascular | PC | 14559 | 7.44E-04 | 0.00817 | 0.46371 |
| Linnarsson_GSE76381_Human_Midbrain | Endo | 14835 | 0.00277 | 0.0308 | 0.46421 |
| MouseCellAtlas_all | Peripheral_Blood.NK_cell_Gzma_high | 14459 | 0.0082 | 0.0932 | 0.46495 |
| MouseCellAtlas_all | Bladder.Epithelial_cell_Upk3a_high | 14459 | 0.00492 | 0.056 | 0.46498 |
| MouseCellAtlas_Mammary_Gland | Mammary_Gland_Lactation.Secretory_alveoli_cell_Klf6_high | 14459 | 0.0089 | 0.102 | 0.46531 |
| MouseCellAtlas_Mammary_Gland | Mammary_Gland_Lactation.Secretory_alveoli_cell_Naxd_high | 14459 | 0.00708 | 0.0815 | 0.46538 |
| TabulaMuris_droplet_all | Trachea.stromal_cell | 13888 | 0.00414 | 0.0478 | 0.4655 |
| DropViz_HC_level1 | Macrophage | 14805 | 3.50E-04 | 0.00405 | 0.46552 |
| MouseCellAtlas_all | Mammary_Gland.Secretory_alveoli_cell_Fcer1g_high | 14459 | 0.00451 | 0.0522 | 0.46557 |
| MouseCellAtlas_Embryo_all | Placenta.Granulocyte_Neat1_high | 14459 | 0.00294 | 0.0342 | 0.46575 |
| Linnarsson_MouseBrainAtlas_level6_rank4 | Psnf | 15140 | 0.00191 | 0.0222 | 0.46577 |
| MouseCellAtlas_Adult_all | Muscle.Macrophage_Retnla_high | 14459 | 0.00499 | 0.0583 | 0.46589 |
| MouseCellAtlas_Adult_all | Peripheral_Blood.Neutrophil_Il1b_high | 14459 | 0.00588 | 0.0692 | 0.46615 |
| TabulaMuris_FACS_all | Lung.B_cell | 13888 | 7.91E-04 | 0.00942 | 0.46653 |
| Linnarsson_MouseBrainAtlas_level5 | SCINH7 | 15140 | 0.00398 | 0.0482 | 0.46712 |
| DropViz_ENT_level2 | Oligodendrocyte_Trf_Cldn11.8_1 | 14805 | 3.68E-04 | 0.00446 | 0.46716 |
| MouseCellAtlas_Mammary_Gland | Mammary_Gland_Lactation.Secretory_alveoli_cell_Bckdha_high | 14459 | 0.00832 | 0.102 | 0.4676 |
| Linnarsson_MouseBrainAtlas_level5 | HBINH7 | 15140 | 0.00252 | 0.0311 | 0.46766 |
| GSE93374_Mouse_Arc_ME_neurons | Nr5a1_Adcyap1 | 13325 | 0.0171 | 0.215 | 0.4683 |
| GSE106678_Mouse_Cortex | Ex1 | 14573 | 0.00669 | 0.0851 | 0.4687 |
| DropViz_all_level2 | GP.Neuron_Gad1Gad2_Adora2a.3_3 | 14805 | 5.48E-04 | 0.00699 | 0.46876 |
| DroNc_Mouse_Hippocampus | GABA1 | 12425 | 0.0165 | 0.211 | 0.46892 |
| DropViz_all_level2 | ENT.Neuron_Gad1Gad2-Chat.4_12 | 14805 | 2.39E-04 | 0.00308 | 0.46902 |
| GSE93374_Mouse_Arc_ME_level2 | Fibroblasts2 | 13325 | 0.00573 | 0.0738 | 0.46907 |
| DropViz_GP_level1 | Ependymal | 14805 | 2.94E-04 | 0.00379 | 0.46908 |
| MouseCellAtlas_Adult_all | Mammary_Gland.Secretory_alveoli_cell_Rgs2_high_Rora_high | 14459 | 0.00486 | 0.0627 | 0.46911 |
| DropViz_all_level2 | SN.Neuron_Gad1Gad2_Gad2-Fos.3_8 | 14805 | 3.75E-04 | 0.00488 | 0.46939 |
| DropViz_all_level2 | FC.Mural_Rgs5Acta2_Ccnd1.13_2 | 14805 | 3.86E-04 | 0.00511 | 0.46989 |
| TabulaMuris_FACS_Fat | neutrophil | 13888 | 7.04E-04 | 0.0094 | 0.47016 |
| DropViz_PC_level2 | Oligodendrocyte_Tfr_Il33.9_4 | 14805 | 4.32E-04 | 0.00578 | 0.47023 |
| MouseCellAtlas_Neonatal_Skin | Epithelial_cell | 14459 | 0.00904 | 0.122 | 0.47048 |
| MouseCellAtlas_Bone_Marrow | Bone_Marrow_Mesenchyme.Myoblast | 14459 | 0.0049 | 0.0664 | 0.47063 |
| TabulaMuris_droplet_Lung | mesothelial_cell | 13888 | 0.00192 | 0.0264 | 0.47098 |
| MouseCellAtlas_Bone_Marrow | Bone_Marrow_c_kit.Monocyte_progenitor | 14459 | 0.00712 | 0.0994 | 0.47145 |
| GSE87544_Mouse_Hypothalamus | GABA14 | 13878 | 0.00524 | 0.0756 | 0.47237 |
| Linnarsson_MouseBrainAtlas_level5 | MEGLU6 | 15140 | 0.00263 | 0.0384 | 0.47272 |
| Linnarsson_MouseBrainAtlas_level5 | CR | 15140 | 0.00471 | 0.0692 | 0.47286 |
| MouseCellAtlas_Embryonic_Mesenchyme | Ganglion_cell_Cartpt_high | 14459 | 0.00718 | 0.107 | 0.47326 |
| Linnarsson_GSE76381_Human_Midbrain | ProgFPL | 14835 | 0.00295 | 0.0454 | 0.47409 |
| DropViz_all_level2 | ENT.Mural_Rgs5Acta2_Pdgfa.3_3 | 14805 | 1.89E-04 | 0.00292 | 0.47419 |
| GSE104276_Human_Prefrontal_cortex_per_ages | GW12_Stem_cells | 17199 | 5.62E-04 | 0.00871 | 0.47428 |
| MouseCellAtlas_Adult_all | Spleen.Plasma_cell | 14459 | 0.00327 | 0.0513 | 0.47462 |
| DropViz_STR_level1 | Oligodendrocyte | 14805 | 4.02E-04 | 0.0066 | 0.47571 |
| TabulaMuris_droplet_Kidney | smooth_muscle_cell | 13888 | 0.00202 | 0.0333 | 0.47584 |
| MouseCellAtlas_Neonatal_all | Neonatal_Muscle.Stromal_cell_Spp1_high | 14459 | 0.00309 | 0.0513 | 0.47595 |
| Linnarsson_GSE60361_Mouse_Cortex_Hippocampus_level2 | S1PyrL6 | 14010 | 0.00177 | 0.0295 | 0.47604 |
| MouseCellAtlas_all | Brain.Macrophage_Klf2_high | 14459 | 0.00352 | 0.0599 | 0.47656 |
| Linnarsson_GSE74672_Mouse_Hypothalamus_Neurons_level2 | GABA_12_Nts_2 | 14550 | 0.00294 | 0.0513 | 0.47715 |
| TabulaMuris_FACS_all | Heart.erythrocyte | 13888 | 6.46E-04 | 0.0113 | 0.47723 |
| MouseCellAtlas_Mammary_Gland | Mammary_Gland_Lactation.Secretory_alveoli_cell_Gm1673_high | 14459 | 0.00574 | 0.101 | 0.47738 |
| DropViz_all_level2 | HC.Neuron_Slc17a7_Fibcd1-Nptx2.5_3 | 14805 | 4.29E-04 | 0.00761 | 0.4775 |
| MouseCellAtlas_all | Uterus.Dendritic_cell | 14459 | 0.00504 | 0.0895 | 0.47752 |
| DropViz_all_level2 | PC.Neuron_Slc17a7_Bcl6-Fos.3_5 | 14805 | 4.12E-04 | 0.00734 | 0.47763 |
| DropViz_PC_level2 | Neuron_Gad1Gad2_Cplx3-Reln.4_1 | 14805 | 4.90E-04 | 0.00878 | 0.47776 |
| TabulaMuris_droplet_Lung | ciliated_cell | 13888 | 0.00118 | 0.0213 | 0.47797 |
| DropViz_TH_level2 | Macrophage_C1qb_Mrc1.7_1 | 14805 | 2.05E-04 | 0.00371 | 0.47802 |
| DropViz_all_level2 | HC.Neuron_Slc17a7_Nxph3-Ajap1.3_11 | 14805 | 3.52E-04 | 0.0064 | 0.47807 |
| MouseCellAtlas_all | Fetal_Intestine.Erythroblast_Hbb.y_high | 14459 | 0.00375 | 0.0689 | 0.47831 |
| DropViz_STR_level2 | Polydendrocye_Tnr_Ctps.5_4 | 14805 | 1.75E-04 | 0.00332 | 0.47902 |
| MouseCellAtlas_Adult_all | Mammary_Gland.Secretory_alveoli_cell_mt.Tp_high | 14459 | 0.00357 | 0.0691 | 0.47936 |
| MouseCellAtlas_all | Neonatal_Heart.Cardiac_muscle_cell | 14459 | 0.00309 | 0.0602 | 0.47949 |
| MouseCellAtlas_Neonatal_Muscle | Adipocyte. | 14459 | 0.00291 | 0.0566 | 0.47953 |
| TabulaMuris_FACS_all | Pancreas.pancreatic_D_cell | 13888 | 3.12E-04 | 0.00614 | 0.47971 |
| MouseCellAtlas_Neonatal_Muscle | Dendritic_cell | 14459 | 0.00389 | 0.0765 | 0.47973 |
| MouseCellAtlas_all | Mammary_Gland.Secretory_alveoli_cell_Ighm1_high | 14459 | 0.00374 | 0.0745 | 0.47997 |
| Allen_Mouse_ALM_level2 | Vip_Cd34_2 | 14744 | 3.51E-04 | 0.00705 | 0.48013 |
| DropViz_STR_level2 | Neuron_Slc17a6_Oprk1.13_6 | 14805 | 1.60E-04 | 0.00324 | 0.48032 |
| TabulaMuris_FACS_all | Mammary.endothelial_cell | 13888 | 4.17E-04 | 0.00851 | 0.48047 |
| Linnarsson_MouseBrainAtlas_level5 | HBSER5 | 15140 | 0.00109 | 0.0224 | 0.48061 |
| TabulaMuris_FACS_all | Marrow.monocyte | 13888 | 3.66E-04 | 0.00766 | 0.48092 |
| MouseCellAtlas_Mammary_Gland | Mammary_Gland_Lactation.Secretory_alveoli_cell_Ifi35_high | 14459 | 0.0047 | 0.0985 | 0.48097 |
| MouseCellAtlas_all | Trophoblast_Stem_Cell.MEF | 14459 | 0.0032 | 0.0688 | 0.48149 |
| DropViz_CB_level2 | Neuron_Gad1Gad2_Pvalb-Chrm2.3_4 | 14805 | 2.03E-04 | 0.00445 | 0.48181 |
| MouseCellAtlas_Mammary_Gland | Mammary_Gland_Pregnancy.NK_cell_Gzma_high | 14459 | 0.0029 | 0.0637 | 0.48184 |
| MouseCellAtlas_Neonatal_all | Neonatal_Muscle.Mast_cell | 14459 | 0.00279 | 0.0627 | 0.48227 |
| GSE87544_Mouse_Hypothalamus | SCO | 13878 | 0.00154 | 0.0353 | 0.4826 |
| MouseCellAtlas_all | Fetal_Stomache.Neuron | 14459 | 0.00405 | 0.0954 | 0.48305 |
| MouseCellAtlas_Mammary_Gland | Mammary_Gland_Virgin.Macrophage_C1qc_high | 14459 | 0.00237 | 0.0559 | 0.48313 |
| Linnarsson_GSE76381_Human_Midbrain | OPC | 14835 | 0.00207 | 0.0498 | 0.4834 |
| MouseCellAtlas_all | Peripheral_Blood.Neutrophil_Il1b_high | 14459 | 0.00281 | 0.0678 | 0.4835 |
| GSE87544_Mouse_Hypothalamus | GABA1 | 13878 | 9.88E-04 | 0.024 | 0.48358 |
| MouseCellAtlas_all | Uterus.Smooth_muscle_cell_Rgs5_high | 14459 | 0.00307 | 0.075 | 0.48366 |
| DropViz_all_level2 | GP.Neuron_Gad1Gad2_Adora2a-Pde1c.3_6 | 14805 | 2.72E-04 | 0.00693 | 0.48434 |
| Linnarsson_MouseBrainAtlas_level5 | TEGLU11 | 15140 | 0.00144 | 0.0377 | 0.48474 |
| Linnarsson_GSE103840_Mouse_Dorsal_horn | GABA_Gal_Rspo3 | 14550 | 0.00263 | 0.0691 | 0.48484 |
| DropViz_PC_level2 | Neuron_Sc17a7_Calb1-Lpl.2_5 | 14805 | 4.18E-04 | 0.0112 | 0.48517 |
| DropViz_all_level2 | HC.Neurogenesis_Sox4_Efhd2.13_1 | 14805 | 1.55E-04 | 0.00422 | 0.48539 |
| MouseCellAtlas_Adult_all | Lung.Ciliated_cell | 14459 | 0.00227 | 0.0631 | 0.48567 |
| MouseCellAtlas_Adult_all | Uterus.NK_cell | 14459 | 0.00301 | 0.0862 | 0.48609 |
| Allen_Mouse_ALM_level2 | Vip_Cd34_1 | 14744 | 3.90E-04 | 0.0112 | 0.4861 |
| MouseCellAtlas_Adult_all | Bladder.Dendritic_cell_Lyz2_high | 14459 | 0.00228 | 0.0657 | 0.48616 |
| MouseCellAtlas_Uterus | Keratinocyte | 14459 | 0.00266 | 0.0782 | 0.48646 |
| Linnarsson_MouseBrainAtlas_level6_rank4 | Vlmc | 15140 | 0.00135 | 0.0402 | 0.48662 |
| MouseCellAtlas_all | Mammary_Gland.Secretory_alveoli_cell_Igfbp7_high | 14459 | 0.002 | 0.0613 | 0.48698 |
| DropViz_all_level2 | CB.Oligodendrocyte_Trf_Klk6.6_2 | 14805 | 1.18E-04 | 0.00363 | 0.48704 |
| MouseCellAtlas_Fetal_Intestine | Erythroblast_Hbb.y_high | 14459 | 0.0026 | 0.0833 | 0.48753 |
| Linnarsson_MouseBrainAtlas_level5 | PSNF2 | 15140 | 7.14E-04 | 0.0229 | 0.48756 |
| MouseCellAtlas_Mammary_Gland | Mammary_Gland_Lactation.Secretory_alveoli_cell_Ly6a_high | 14459 | 0.00256 | 0.082 | 0.48756 |
| GSE93374_Mouse_Arc_ME_neurons | Th_Cxcl12 | 13325 | 0.0116 | 0.374 | 0.48762 |
| MouseCellAtlas_Adult_all | Liver.Neutrophil_Ngp_high | 14459 | 0.00166 | 0.0538 | 0.48769 |
| MouseCellAtlas_Fetal_Stomache | Macrophage | 14459 | 0.00386 | 0.125 | 0.48771 |
| DropViz_CB_level2 | Neuron_Slc17a6_Nrgn.4_3 | 14805 | 1.11E-04 | 0.00363 | 0.48784 |
| Linnarsson_GSE76381_Mouse_Midbrain | NbML5 | 14550 | 0.00252 | 0.0836 | 0.48796 |
| TabulaMuris_FACS_all | Kidney.fibroblast | 13888 | 5.22E-04 | 0.0173 | 0.48798 |
| DropViz_HC_level2 | Polydendrocyte_Tnr_Bmp4.9_2 | 14805 | 1.51E-04 | 0.00505 | 0.4881 |
| Linnarsson_GSE60361_Mouse_Cortex_Hippocampus_level2 | SubPyr | 14010 | 8.86E-04 | 0.0299 | 0.48819 |
| MouseCellAtlas_all | Embryonic_Mesenchyme.Muscle | 14459 | 0.00248 | 0.0844 | 0.48829 |
| Linnarsson_GSE74672_Mouse_Hypothalamus_Neurons_level2 | GABA_5_Calcr_Lhx1 | 14550 | 0.00201 | 0.0689 | 0.48838 |
| GSE92332_Mouse_Epithelium_SMARTseq | Paneth | 13578 | 4.24E-04 | 0.0146 | 0.48839 |
| MouseCellAtlas_all | Mammary_Gland.Secretory_alveoli_cell_Rhob_high | 14459 | 0.00182 | 0.0647 | 0.48878 |
| GSE93374_Mouse_Arc_ME_level2 | b2_tanycytes1 | 13325 | 0.00335 | 0.12 | 0.4889 |
| MouseCellAtlas_Adult_all | Uterus.Smooth_muscle_cell_Rgs5_high | 14459 | 0.00204 | 0.0738 | 0.48896 |
| Linnarsson_GSE59739_Mouse_Dorsal_root_ganglion_level3 | Non_peptidergic_nocieptor1 | 13848 | 3.21E-04 | 0.0118 | 0.48918 |
| GSE98816_Mouse_Brain_Vascular | MG | 14130 | 1.59E-04 | 0.00603 | 0.48946 |
| MouseCellAtlas_Adult_all | Small_Intestine.Macrophage_Cxcl2_high | 14459 | 0.00102 | 0.0414 | 0.49018 |
| GSE93374_Mouse_Arc_ME_neurons | Htr3b | 13325 | 0.0108 | 0.44 | 0.4902 |
| MouseCellAtlas_all | Muscle.Macrophage_Retnla_high | 14459 | 0.0013 | 0.0563 | 0.49079 |
| MouseCellAtlas_Adult_all | Spleen.NK_cell | 14459 | 0.00207 | 0.0929 | 0.4911 |
| TabulaMuris_droplet_all | Lung.mesothelial_cell | 13888 | 5.63E-04 | 0.0256 | 0.49124 |
| Allen_Mouse_LGd_level2 | Slc17a6 | 14545 | 1.76E-04 | 0.00898 | 0.4922 |
| Linnarsson_GSE74672_Mouse_Hypothalamus_Neurons_level2 | Dopamine_4 | 14550 | 4.54E-04 | 0.0236 | 0.49234 |
| Linnarsson_GSE76381_Mouse_Midbrain | DA0 | 14550 | 0.00122 | 0.0684 | 0.49289 |
| GSE87544_Mouse_Hypothalamus | POPC | 13878 | 6.86E-04 | 0.0388 | 0.49294 |
| MouseCellAtlas_all | Spleen.Plasma_cell | 14459 | 8.67E-04 | 0.0501 | 0.49309 |
| DropViz_GP_level2 | Oligodendrocyte_Trf_Mbp.10_1 | 14805 | 1.09E-04 | 0.00641 | 0.49322 |
| GSE98816_Mouse_Brain_Vascular | vEC | 14130 | 1.70E-04 | 0.01 | 0.49325 |
| DropViz_HC_level2 | Neuron_Slc17a6_Lhx1.14_1 | 14805 | 1.07E-04 | 0.00698 | 0.49387 |
| MouseCellAtlas_all | Lung.Ciliated_cell | 14459 | 9.20E-04 | 0.0628 | 0.49416 |
| GSE93374_Mouse_Arc_ME_neurons | Sst_Unc13c | 13325 | 0.00615 | 0.427 | 0.49426 |
| MouseCellAtlas_all | Neonatal_Muscle.Neutrophil_Elane_high | 14459 | 8.77E-04 | 0.0636 | 0.4945 |
| Linnarsson_GSE60361_Mouse_Cortex_Hippocampus_level2 | S1PyrL5 | 14010 | 2.86E-04 | 0.0211 | 0.49459 |
| MouseCellAtlas_Neonatal_all | Neonatal_Heart.Neutrophil_Retnlg_high | 14459 | 8.44E-04 | 0.063 | 0.49466 |
| DropViz_STR_level1 | Mural | 14805 | 7.76E-05 | 0.0059 | 0.49476 |
| DropViz_all_level2 | GP.Neuron_Slc17a6_Rspo3.2_9 | 14805 | 9.23E-05 | 0.00731 | 0.49497 |
| GSE93374_Mouse_Arc_ME_level1 | Oligodend3 | 13325 | 5.21E-04 | 0.0447 | 0.49535 |
| MouseCellAtlas_Adult_all | Prostate.T_cell | 14459 | 0.00116 | 0.116 | 0.496 |
| Linnarsson_GSE67602_Mouse_Skin_Epidermis | IFE_KII | 14528 | 2.46E-04 | 0.0248 | 0.49605 |
| DropViz_all_level2 | ENT.Neuron_Gad1Gad2_Pmch.4_10 | 14805 | 3.01E-05 | 0.00305 | 0.49606 |
| MouseCellAtlas_Mammary_Gland | Mammary_Gland_Lactation.Secretory_alveoli_cell_Tmem123_high | 14459 | 7.59E-04 | 0.0799 | 0.49621 |
| DropViz_all_level2 | PC.Oligodendrocyte_Tfr_Il33.9_4 | 14805 | 6.06E-05 | 0.00657 | 0.49632 |
| DropViz_all_level2 | TH.Neuron_Slc17a6_Rora_Atp2b4.2_1 | 14805 | 6.31E-05 | 0.00721 | 0.49651 |
| DropViz_CB_level2 | Oligodendrocyte_Trf_Klk6.6_2 | 14805 | 3.34E-05 | 0.00397 | 0.49664 |
| DropViz_all_level2 | ENT.Neuron_Gad1Gad2-Slc17a6_Cbln1.4_3 | 14805 | 4.61E-05 | 0.00559 | 0.49671 |
| MouseCellAtlas_Small_Intestine | Macrophage_Cxcl2_high | 14459 | 3.36E-04 | 0.0424 | 0.49684 |
| DropViz_all_level2 | STR.Mural_Rgs5Acta2_Kcnj8.8_4 | 14805 | 2.71E-05 | 0.00348 | 0.49689 |
| MouseCellAtlas_Mammary_Gland | Mammary_Gland_Lactation.Secretory_alveoli_cell_Wfdc17_high | 14459 | 6.79E-04 | 0.106 | 0.49745 |
| DropViz_PC_level2 | Neuron_Sc17a7_Calb1-Lpl-Cadm2.2_8 | 14805 | 3.44E-05 | 0.00601 | 0.49772 |
| GSE93374_Mouse_Arc_ME_level1 | Ependymocytes | 13325 | 2.06E-04 | 0.0448 | 0.49817 |
| DropViz_all_level2 | TH.Neuron_Slc17a6_Rora_Lrrtm2.2_9 | 14805 | 2.30E-05 | 0.00595 | 0.49846 |
| MouseCellAtlas_Adult_all | Mammary_Gland.Secretory_alveoli_cell_Tmsb10_high | 14459 | 2.59E-04 | 0.068 | 0.49848 |
| MouseCellAtlas_Embryo_all | Fetal_Stomache.Endocrine_cell | 14459 | 4.25E-04 | 0.112 | 0.49849 |
| MouseCellAtlas_all | Fetal_Stomache.Macrophage | 14459 | 2.93E-04 | 0.117 | 0.49901 |
| MouseCellAtlas_all | Uterus.NK_cell | 14459 | 2.06E-04 | 0.0854 | 0.49904 |
| MouseCellAtlas_all | Mammary_Gland.Secretory_alveoli_cell_Rgs2_high_Rora_high | 14459 | 1.33E-04 | 0.0611 | 0.49913 |
| DropViz_PC_level2 | Neuron_Gad1Gad2_Synpr-Pcdh11x.4_9 | 14805 | 3.09E-06 | 0.00786 | 0.49984 |
| GSE93374_Mouse_Arc_ME_neurons | Hdc | 13325 | -1.17E-05 | 0.0907 | 0.50005 |
| TabulaMuris_droplet_Spleen | macrophage | 13888 | -9.53E-05 | 0.101 | 0.50038 |
| MouseCellAtlas_Thymus | T_cell_Ms4a4b_high | 14459 | -2.62E-04 | 0.177 | 0.50059 |
| DropViz_GP_level1 | Microglia | 14805 | -1.08E-05 | 0.00484 | 0.50089 |
| MouseCellAtlas_Mammary_Gland | Mammary_Gland_Lactation.Secretory_alveoli_cell_Lgals1_high | 14459 | -2.29E-04 | 0.0834 | 0.50109 |
| MouseCellAtlas_Spleen | T_cell | 14459 | -4.09E-04 | 0.125 | 0.50131 |
| DropViz_STR_level2 | Neuron_Gad1Gad2_Adora2a-Fos.11_3 | 14805 | -2.77E-05 | 0.00779 | 0.50142 |
| Linnarsson_GSE74672_Mouse_Hypothalamus_level1 | microglia | 14550 | -2.47E-04 | 0.051 | 0.50193 |
| MouseCellAtlas_all | Ovary.Granulosa_cell_Kctd14_high | 14459 | -5.09E-04 | 0.093 | 0.50218 |
| DropViz_FC_level2 | Neuron_Slc17a7_Parm1_Nefm.7_6 | 14805 | -5.36E-05 | 0.00973 | 0.5022 |
| MouseCellAtlas_Embryo_all | Fetal_Brain.Radial_glia_Fabp7_high | 14459 | -5.66E-04 | 0.0969 | 0.50233 |
| MouseCellAtlas_Adult_all | Peripheral_Blood.Monocyte_Elane_high | 14459 | -3.09E-04 | 0.052 | 0.50237 |
| DropViz_HC_level2 | Mural_Rgs5Acta2_Kcnj8.16_3 | 14805 | -2.43E-05 | 0.00379 | 0.50255 |
| DropViz_STR_level2 | Endothelial_Tip_Dcn.9_1 | 14805 | -3.13E-05 | 0.00485 | 0.50258 |
| DropViz_STR_level2 | Neuron_Gad1Gad2_Adora2a-Th.13_5 | 14805 | -3.92E-05 | 0.00603 | 0.50259 |
| DropViz_all_level2 | HC.Mural_Rgs5Acta2_Cnn1.16_1 | 14805 | -3.69E-05 | 0.00478 | 0.50308 |
| MouseCellAtlas_all | Peripheral_Blood.Monocyte_Elane_high | 14459 | -4.35E-04 | 0.0535 | 0.50325 |
| TabulaMuris_FACS_all | Fat.smooth_muscle_cell | 13888 | -5.42E-05 | 0.0065 | 0.50332 |
| TabulaMuris_droplet_all | Lung.ciliated_cell | 13888 | -1.65E-04 | 0.0199 | 0.50332 |
| DropViz_FC_level2 | Microglia_C1qb_Tmem119-Fos.11_4 | 14805 | -2.34E-05 | 0.00267 | 0.5035 |
| GSE93374_Mouse_Arc_ME_neurons | Qrfp | 13325 | -0.00186 | 0.182 | 0.50408 |
| Linnarsson_GSE74672_Mouse_Hypothalamus_Neurons_level2 | Vglut2_10_Morn4Prrc2a | 14550 | -2.35E-04 | 0.0193 | 0.50487 |
| Linnarsson_GSE60361_Mouse_Cortex_Hippocampus_level2 | Oligo4 | 14010 | -2.41E-04 | 0.0197 | 0.50488 |
| DropViz_GP_level2 | Polydendrocyte_Tnr_Bmp4.4_4 | 14805 | -7.90E-05 | 0.00593 | 0.50532 |
| MouseCellAtlas_Adult_all | Brain.Hypothalamic_ependymal_cell | 14459 | -4.84E-04 | 0.0362 | 0.50534 |
| MouseCellAtlas_Mammary_Gland | Mammary_Gland_Lactation.Secretory_alveoli_cell_Cidea_high | 14459 | -0.0013 | 0.0968 | 0.50534 |
| TabulaMuris_FACS_Aorta | endothelial_cell | 13888 | -1.44E-04 | 0.0108 | 0.50535 |
| Linnarsson_GSE101601_Mouse_Somatosensory_cortex | Astrocyte_il18 | 14550 | -9.44E-04 | 0.0669 | 0.50563 |
| Allen_Mouse_VISp_level2 | L5a_Tcerg1l | 13868 | -2.26E-04 | 0.0153 | 0.5059 |
| MouseCellAtlas_Lung | Interstitial_macrophage | 14459 | -0.00149 | 0.0988 | 0.506 |
| MouseCellAtlas_all | Fetal_Brain.Microglia | 14459 | -0.00133 | 0.0813 | 0.50655 |
| MouseCellAtlas_all | Liver.Neutrophil_Ngp_high | 14459 | -8.80E-04 | 0.0528 | 0.50665 |
| MouseCellAtlas_Bone_Marrow | Bone_Marrow_c_kit.Neutrophil_Fcnb_high | 14459 | -0.00116 | 0.0688 | 0.50674 |
| MouseCellAtlas_all | Prostate.T_cell | 14459 | -0.00201 | 0.115 | 0.50698 |
| GSE87544_Mouse_Hypothalamus | Glu15 | 13878 | -9.10E-04 | 0.051 | 0.50713 |
| MouseCellAtlas_all | Fetal_Brain.Radial_glia_Vim_high | 14459 | -0.00178 | 0.0986 | 0.50722 |
| DropViz_PC_level2 | Neuron_Slc17a7_Bcl6-Npnt.3_10 | 14805 | -1.73E-04 | 0.00943 | 0.50734 |
| MouseCellAtlas_Peripheral_Blood | Basophil_Prss34_high | 14459 | -0.002 | 0.103 | 0.50771 |
| Linnarsson_GSE60361_Mouse_Cortex_Hippocampus_level2 | Int11 | 14010 | -5.17E-04 | 0.0264 | 0.50782 |
| GSE104276_Human_Prefrontal_cortex_per_ages | GW26_Astrocytes | 17199 | -1.71E-04 | 0.00858 | 0.50794 |
| MouseCellAtlas_Adult_all | Mammary_Gland.Secretory_alveoli_cell_Cd63_high | 14459 | -0.00129 | 0.0644 | 0.50798 |
| TabulaMuris_FACS_all | Marrow.B_cell | 13888 | -1.99E-04 | 0.00929 | 0.50855 |
| MouseCellAtlas_all | Brain.Hypothalamic_ependymal_cell | 14459 | -8.02E-04 | 0.0362 | 0.50885 |
| MouseCellAtlas_all | Spleen.NK_cell | 14459 | -0.00206 | 0.0906 | 0.50908 |
| MouseCellAtlas_all | Small_Intestine.Macrophage_Cxcl2_high | 14459 | -9.57E-04 | 0.0408 | 0.50937 |
| DropViz_all_level2 | SN.Neuron_Th-Slc17a6-Lpl.4_1 | 14805 | -1.47E-04 | 0.00619 | 0.50947 |
| TabulaMuris_FACS_all | Thymus.mesenchymal_stem_cell | 13888 | -3.22E-04 | 0.0135 | 0.50953 |
| TabulaMuris_droplet_Mammary | B_cell | 13888 | -0.00125 | 0.052 | 0.50959 |
| MouseCellAtlas_Bone_Marrow | Bone_Marrow.Dendritic_cell_Siglech_high | 14459 | -0.00122 | 0.05 | 0.50971 |
| GSE100597_Mouse_Embryo | E5.5 | 13879 | -3.11E-04 | 0.0125 | 0.50994 |
| DropViz_PC_level2 | Neuron_Slc17a7_Syt6-Slc24a2.1_6 | 14805 | -1.50E-04 | 0.00594 | 0.5101 |
| MouseCellAtlas_all | Neonatal_Muscle.Neutrophil_Hmox1_high | 14459 | -0.00137 | 0.0532 | 0.5103 |
| DropViz_FC_level1 | Macrophage | 14805 | -1.38E-04 | 0.00523 | 0.51051 |
| DropViz_all_level2 | GP.Neuron_Gad1Gad2_Six3-Pvalb.2_20 | 14805 | -1.73E-04 | 0.00642 | 0.51073 |
| DropViz_all_level2 | STR.Microglia_C1qb_Tmem119.6_2 | 14805 | -1.34E-04 | 0.00467 | 0.51146 |
| DropViz_all_level2 | FC.Microglia_C1qb_Tmem119-Fos.11_4 | 14805 | -7.99E-05 | 0.00267 | 0.51193 |
| DropViz_all_level2 | HC.Neuron_Slc17a7_Pvrl3-Nos1.6_1 | 14805 | -2.31E-04 | 0.00756 | 0.51221 |
| MouseCellAtlas_Liver | Neutrophil_Ngp_high | 14459 | -0.00199 | 0.0618 | 0.51285 |
| MouseCellAtlas_all | Mammary_Gland.Secretory_alveoli_cell_mt.Tp_high | 14459 | -0.00216 | 0.0668 | 0.5129 |
| MouseCellAtlas_Mammary_Gland | Mammary_Gland_Involution.B_cell_Cd79a_high | 14459 | -0.00204 | 0.0607 | 0.51337 |
| DropViz_TH_level2 | Neuron_Slc17a6_Rora_Lypd6.2_7 | 14805 | -2.48E-04 | 0.00734 | 0.51346 |
| MouseCellAtlas_Mammary_Gland | Mammary_Gland_Lactation.Secretory_alveoli_cell_Mat2a_high | 14459 | -0.00329 | 0.0922 | 0.51421 |
| DropViz_GP_level2 | Mural_Rgs5Acta2_Rgs5.8_4 | 14805 | -1.79E-04 | 0.00478 | 0.51493 |
| DropViz_ENT_level1 | Mural | 14805 | -2.12E-04 | 0.00558 | 0.51515 |
| Allen_Mouse_ALM_level2 | Vip_Chat_4 | 14744 | -3.43E-04 | 0.00885 | 0.51543 |
| MouseCellAtlas_all | Bladder.Dendritic_cell_Lyz2_high | 14459 | -0.00245 | 0.0619 | 0.51579 |
| MouseCellAtlas_Embryo_all | Fetal_Brain.Microglia | 14459 | -0.00344 | 0.0867 | 0.51585 |
| DropViz_SN_level2 | Polydendrocyte_Tnr_Bmp4.5_1 | 14805 | -2.01E-04 | 0.00492 | 0.51628 |
| MouseCellAtlas_Kidney | Macrophage_Ccl4_high_ | 14459 | -0.00418 | 0.102 | 0.51628 |
| Linnarsson_GSE60361_Mouse_Cortex_Hippocampus_level1 | pyramidal_SS | 14010 | -0.00148 | 0.0357 | 0.51655 |
| MouseCellAtlas_Ovary | Small_luteal_cell | 14459 | -0.00603 | 0.142 | 0.51692 |
| MouseCellAtlas_Brain | Myelinating_oligodendrocyte | 14459 | -0.00325 | 0.0737 | 0.51755 |
| MouseCellAtlas_Embryo_all | Fetal_Stomache.Macrophage | 14459 | -0.00575 | 0.13 | 0.5176 |
| DropViz_all_level2 | STR.Oligodendrocyte_Trf_Il33.3_2 | 14805 | -2.73E-04 | 0.00599 | 0.51818 |
| MouseCellAtlas_Embryo_all | Fetal_Brain.Purkinje_cell | 14459 | -0.0031 | 0.0677 | 0.51823 |
| GSE93374_Mouse_Arc_ME_neurons | Nr5a1_Nfib | 13325 | -0.0162 | 0.354 | 0.51831 |
| GSE92332_Mouse_Epithelium_SMARTseq | Stem | 13578 | -6.68E-04 | 0.0144 | 0.51856 |
| DropViz_all_level2 | ENT.Oligodendrocyte_Trf_Klk6.1_2 | 14805 | -2.36E-04 | 0.00501 | 0.51881 |
| GSE93374_Mouse_Arc_ME_level2 | Pars_Tuber1B | 13325 | -0.00378 | 0.0791 | 0.51906 |
| Linnarsson_GSE74672_Mouse_Hypothalamus_Neurons_level2 | GABA_6_Otof_Lhx1 | 14550 | -0.00292 | 0.0589 | 0.51975 |
| Allen_Mouse_VISp_level1 | Gluta_L2_3 | 13868 | -6.63E-04 | 0.0133 | 0.51983 |
| MouseCellAtlas_Adult_all | Kidney.Stromal_cell_Ankrd1_high | 14459 | -0.00304 | 0.0605 | 0.52007 |
| MouseCellAtlas_Neonatal_Muscle | T_cell | 14459 | -0.00375 | 0.0739 | 0.52024 |
| MouseCellAtlas_all | Fetal_Stomache.Endocrine_cell | 14459 | -0.00485 | 0.0954 | 0.52027 |
| DropViz_SN_level2 | Oligodendrocyte_Tfr_Slco3a1.10_1 | 14805 | -3.23E-04 | 0.00628 | 0.52049 |
| MouseCellAtlas_Adult_all | Ovary.Granulosa_cell_Kctd14_high | 14459 | -0.00433 | 0.0839 | 0.5206 |
| GSE106678_Mouse_Cortex | Inh4 | 14573 | -0.00474 | 0.0914 | 0.52068 |
| MouseCellAtlas_Adult_all | Mammary_Gland.B_cell_Jchain_high | 14459 | -0.00458 | 0.0881 | 0.5207 |
| DropViz_FC_level2 | Neuron_Slc17a7_Nptxr-Calb1.6_1 | 14805 | -5.06E-04 | 0.00965 | 0.52091 |
| MouseCellAtlas_Placenta | Progenitor_trophoblast_Gjb3_high | 14459 | -0.00382 | 0.0721 | 0.52114 |
| TabulaMuris_FACS_all | Marrow.granulocyte | 13888 | -3.63E-04 | 0.00682 | 0.5212 |
| MouseCellAtlas_Adult_all | Testis.Pre.Sertoli_cell_Ctsl_high | 14459 | -0.00241 | 0.0436 | 0.52201 |
| DropViz_PC_level2 | Neuron_Slc17a7-Slc17a6_Calb1-Rorb-Il1rapl2.2_13 | 14805 | -6.78E-04 | 0.0122 | 0.52208 |
| MouseCellAtlas_Mammary_Gland | Mammary_Gland_Involution.Endothelial_cell_Glycam1_high | 14459 | -0.00333 | 0.06 | 0.52213 |
| DropViz_all_level2 | SN.Oligodendrocyte_Tfr_Plin3.10_2 | 14805 | -2.94E-04 | 0.00527 | 0.52227 |
| Linnarsson_MouseBrainAtlas_level5 | HBINH8 | 15140 | -0.00156 | 0.028 | 0.52228 |
| DropViz_all_level2 | HC.Neuron_Slc17a6_Nnat.2_4 | 14805 | -3.72E-04 | 0.00663 | 0.52234 |
| MouseCellAtlas_all | Kidney.Stromal_cell_Ankrd1_high | 14459 | -0.00344 | 0.0606 | 0.52261 |
| MouseCellAtlas_Ovary | Cumulus_cell_Ube2c_high | 14459 | -0.00563 | 0.0989 | 0.5227 |
| DroNc_Human_Hippocampus | GABA2 | 16914 | -0.011 | 0.19 | 0.52306 |
| MouseCellAtlas_Adult_all | Small_Intestine.B_cell_Igkv12.46_high | 14459 | -0.00349 | 0.0603 | 0.5231 |
| MouseCellAtlas_all | Neonatal_Calvaria.Muscle_cell_Actc1_high | 14459 | -0.00381 | 0.0647 | 0.52349 |
| DropViz_SN_level2 | Neuron_Th_Cyp26b1.4_3 | 14805 | -3.88E-04 | 0.00633 | 0.52443 |
| MouseCellAtlas_all | Neonatal_Heart.Atrial_cardiomyocyte_Acta2_high | 14459 | -0.00546 | 0.0879 | 0.52474 |
| MouseCellAtlas_all | Testis.Pre.Sertoli_cell_Ctsl_high | 14459 | -0.00282 | 0.0436 | 0.5258 |
| DropViz_GP_level2 | Mural_Rgs5Acta2_Kcnj8.8_2 | 14805 | -2.55E-04 | 0.00394 | 0.52585 |
| MouseCellAtlas_Neonatal_all | Neonatal_Heart.Smooth_muscle_cell | 14459 | -0.00507 | 0.0781 | 0.5259 |
| MouseCellAtlas_Adult_all | Lung.Dividing_cells | 14459 | -0.00408 | 0.0622 | 0.52612 |
| DropViz_HC_level2 | Neuron_Slc17a6_Htr1a.2_2 | 14805 | -5.25E-04 | 0.00779 | 0.52687 |
| MouseCellAtlas_Mammary_Gland | Mammary_Gland_Involution.Endothelial_cell_Fabp4_high | 14459 | -0.00455 | 0.0673 | 0.52692 |
| GSE87544_Mouse_Hypothalamus | GABA15 | 13878 | -0.00463 | 0.067 | 0.52757 |
| DropViz_TH_level2 | Mural_Rgs5Acta2_Cnn1.5_1 | 14805 | -3.31E-04 | 0.00475 | 0.52773 |
| MouseCellAtlas_Peripheral_Blood | Neutrophil_Ltf_high | 14459 | -0.00365 | 0.0522 | 0.52787 |
| MouseCellAtlas_Adult_all | Small_Intestine.T_cell_Icos_high | 14459 | -0.00603 | 0.0859 | 0.52799 |
| Allen_Mouse_VISp_level2 | Sncg | 13868 | -7.85E-04 | 0.011 | 0.52833 |
| DropViz_FC_level2 | Polydendrocyte_Tnr_Cspg5.10_1 | 14805 | -4.68E-04 | 0.00645 | 0.52893 |
| MouseCellAtlas_all | Mammary_Gland.Secretory_alveoli_cell_Tmsb10_high | 14459 | -0.00489 | 0.0661 | 0.52952 |
| DropViz_all_level2 | HC.Neuron_Slc17a7_Pvrl3-Inhba.6_7 | 14805 | -5.36E-04 | 0.0072 | 0.52966 |
| MouseCellAtlas_all | Lung.Dividing_cells | 14459 | -0.00468 | 0.0629 | 0.52967 |
| MouseCellAtlas_Fetal_Lung | Neutrophil_Gm5483_high | 14459 | -0.0124 | 0.165 | 0.52992 |
| MouseCellAtlas_Adult_all | Mammary_Gland.Secretory_alveoli_cell_Cd83_high | 14459 | -0.00475 | 0.0628 | 0.53014 |
| MouseCellAtlas_Embryonic_Stem_Cell | ES_Rps28_high | 14459 | -0.013 | 0.171 | 0.53017 |
| DropViz_GP_level2 | Neuron_Gad1Gad2_Vip.2_21 | 14805 | -4.79E-04 | 0.00632 | 0.53019 |
| MouseCellAtlas_Mammary_Gland | Mammary_Gland_Pregnancy.NK_cells_Gzmb_high | 14459 | -0.00256 | 0.0334 | 0.53052 |
| TabulaMuris_FACS_Lung | B_cell | 13888 | -7.10E-04 | 0.00924 | 0.53063 |
| TabulaMuris_FACS_Brain_Neurons | oligodendrocyte_precursor_cell | 13888 | -6.46E-04 | 0.00838 | 0.53076 |
| DropViz_STR_level2 | Neuron_Gad1Gad2_Adora2a-Otof.13_4 | 14805 | -5.29E-04 | 0.00684 | 0.53084 |
| Allen_Mouse_VISp_level2 | L5a_Batf3 | 13868 | -0.00121 | 0.0154 | 0.53132 |
| Linnarsson_GSE101601_Mouse_Somatosensory_cortex | Interneuron_vip_npy | 14550 | -0.00326 | 0.0415 | 0.53132 |
| MouseCellAtlas_Uterus | Macrophage | 14459 | -0.00586 | 0.0739 | 0.53162 |
| TabulaMuris_droplet_Marrow | B_cell | 13888 | -0.00311 | 0.0391 | 0.53169 |
| MouseCellAtlas_Adult_all | Brain.Microglia | 14459 | -0.00473 | 0.0595 | 0.53172 |
| Linnarsson_GSE95315_Mouse_Dentate_gyrus | Radial | 11815 | -0.00558 | 0.0701 | 0.53173 |
| MouseCellAtlas_all | Ovary.Cumulus_cell_Nupr1_high | 14459 | -0.008 | 0.0987 | 0.53231 |
| TabulaMuris_FACS_all | Colon.Brush_cell_of_epithelium_proper_of_large_intestine | 13888 | -6.25E-04 | 0.00767 | 0.53248 |
| GSE87544_Mouse_Hypothalamus | Glu9 | 13878 | -0.00547 | 0.0669 | 0.53256 |
| Allen_Mouse_ALM_level2 | Vip_Sncg_1 | 14744 | -5.44E-04 | 0.00663 | 0.53271 |
| DropViz_PC_level2 | Mural_Acta2Rgs5_Acta2.13_1 | 14805 | -3.67E-04 | 0.00447 | 0.53273 |
| MouseCellAtlas_Adult_all | Testis.Spermatids_Cst13_high | 14459 | -0.00295 | 0.0356 | 0.53298 |
| MouseCellAtlas_Adult_all | Stomach.Pit_cell_Gm26917_high | 14459 | -0.00367 | 0.0443 | 0.533 |
| TabulaMuris_FACS_Fat | smooth_muscle_cell | 13888 | -5.45E-04 | 0.00655 | 0.53314 |
| GSE92332_Mouse_Epithelium_droplet | Goblet | 11993 | -0.00336 | 0.0402 | 0.53331 |
| GSE93374_Mouse_Arc_ME_neurons | Rgs16_Vip | 13325 | -0.0194 | 0.231 | 0.53339 |
| DropViz_HC_level1 | Ependymal | 14805 | -4.39E-04 | 0.00517 | 0.53384 |
| Linnarsson_GSE59739_Mouse_Dorsal_root_ganglion_level2 | Non_peptidergic_nocieptor1 | 13848 | -0.00108 | 0.0126 | 0.53391 |
| GSE89164_Mouse_Hindbrain | Cerebellar_neurons | 12262 | -0.00953 | 0.112 | 0.53404 |
| MouseCellAtlas_Adult_all | Bone_Marrow.T_cell_Ms4a4b_high | 14459 | -0.00694 | 0.0806 | 0.53427 |
| DropViz_PC_level2 | Neuron_Sc17a7_Crym.2_16 | 14805 | -9.28E-04 | 0.0107 | 0.53441 |
| MouseCellAtlas_all | Neonatal_Muscle.Neutrophil_Stfa3_high | 14459 | -0.00665 | 0.0763 | 0.53471 |
| DroNc_Mouse_Hippocampus | exCA3 | 12425 | -0.0164 | 0.188 | 0.53482 |
| Linnarsson_MouseBrainAtlas_level5 | TEGLU4 | 15140 | -0.00304 | 0.0347 | 0.53492 |
| Linnarsson_MouseBrainAtlas_level5 | DECHO1 | 15140 | -0.00258 | 0.0292 | 0.53517 |
| DropViz_all_level2 | HC.Neuron_Slc17a7_Calb2-Adcyap1.6_6 | 14805 | -6.47E-04 | 0.0073 | 0.53532 |
| MouseCellAtlas_Mammary_Gland | Mammary_Gland_Lactation.B_cell_Jchain_high | 14459 | -0.0092 | 0.103 | 0.53547 |
| MouseCellAtlas_Lung | AT1_Cell | 14459 | -0.00675 | 0.0749 | 0.5359 |
| MouseCellAtlas_Mammary_Gland | Mammary_Gland_Lactation.Secretory_alveoli_cell_Cyb561_high | 14459 | -0.00665 | 0.0738 | 0.53592 |
| MouseCellAtlas_Mammary_Gland | Mammary_Gland_Lactation.Secretory_alveoli_cell_Sars_high | 14459 | -0.00844 | 0.0923 | 0.53643 |
| MouseCellAtlas_Mammary_Gland | Mammary_Gland_Lactation.Secretory_alveoli_cell_Iglc1_high | 14459 | -0.00834 | 0.0909 | 0.53656 |
| Linnarsson_MouseBrainAtlas_level5 | HBINH2 | 15140 | -0.00219 | 0.0238 | 0.53668 |
| MouseCellAtlas_all | Neonatal_Muscle.Erythroblast_Hbb.bs_high | 14459 | -0.00557 | 0.06 | 0.53699 |
| Linnarsson_MouseBrainAtlas_level5 | HBSER4 | 15140 | -0.00239 | 0.0257 | 0.53705 |
| DropViz_ENT_level2 | Astrocyte_Gja1_Myoc.7_1 | 14805 | -3.73E-04 | 0.00398 | 0.53735 |
| MouseCellAtlas_Mammary_Gland | Mammary_Gland_Pregnancy.Dendritic_cell_Fscn1_high | 14459 | -0.0044 | 0.0468 | 0.5375 |
| DropViz_all_level2 | SN.Neuron_Slc17a6.2_1 | 14805 | -6.03E-04 | 0.0064 | 0.53753 |
| MouseCellAtlas_all | Testis.Spermatids_Cst13_high | 14459 | -0.00335 | 0.0356 | 0.53757 |
| MouseCellAtlas_all | Lung.Monocyte_progenitor_cell | 14459 | -0.00458 | 0.0485 | 0.53762 |
| Linnarsson_GSE60361_Mouse_Cortex_Hippocampus_level2 | Int4 | 14010 | -0.00354 | 0.0375 | 0.53763 |
| DropViz_all_level2 | TH.Neuron_Slc17a6_Rora_Calca.2_3 | 14805 | -6.81E-04 | 0.0072 | 0.53769 |
| MouseCellAtlas_Embryo_all | Fetal_Liver.Hepatocyte_Afp_high | 14459 | -0.00467 | 0.0489 | 0.53798 |
| MouseCellAtlas_Adult_all | Lung.Monocyte_progenitor_cell | 14459 | -0.00451 | 0.0472 | 0.53808 |
| MouseCellAtlas_all | Mammary_Gland.Secretory_alveoli_cell_Cd63_high | 14459 | -0.00597 | 0.0625 | 0.53808 |
| Linnarsson_GSE60361_Mouse_Cortex_Hippocampus_level2 | S1PyrL5a | 14010 | -0.00335 | 0.0342 | 0.53902 |
| DropViz_all_level2 | HC.Neurogenesis_Sox4_Hist1h2al.13_2 | 14805 | -4.24E-04 | 0.00432 | 0.53907 |
| DropViz_all_level2 | ENT.Neuron_Slc17a7_Zbtb20.4_1 | 14805 | -3.83E-04 | 0.00384 | 0.53966 |
| Linnarsson_GSE101601_Human_Temporal_cortex | Glut_2 | 17177 | -0.0127 | 0.127 | 0.53969 |
| MouseCellAtlas_Mammary_Gland | Mammary_Gland_Lactation.Secretory_alveoli_cell_Gadd45b_high | 14459 | -0.0102 | 0.102 | 0.53976 |
| DropViz_ENT_level2 | Oligodendrocyte_Trf_Ndrg2.1_3 | 14805 | -6.95E-04 | 0.00692 | 0.53997 |
| MouseCellAtlas_Mammary_Gland | Mammary_Gland_Lactation.Secretory_alveoli_cell_Mrpl15_high | 14459 | -0.0096 | 0.0948 | 0.54035 |
| MouseCellAtlas_all | Fetal_Liver.Hepatocyte_Afp_high | 14459 | -0.00483 | 0.0474 | 0.54055 |
| MouseCellAtlas_all | Placenta.Basophil | 14459 | -0.00727 | 0.0711 | 0.54073 |
| MouseCellAtlas_Adult_all | Stomach.G_cell | 14459 | -0.00681 | 0.0666 | 0.54076 |
| MouseCellAtlas_all | Neonatal_Skin.Macrophage_Cd74_high | 14459 | -0.00492 | 0.048 | 0.54083 |
| MouseCellAtlas_all | Small_Intestine.T_cell_Icos_high | 14459 | -0.00874 | 0.0851 | 0.5409 |
| MouseCellAtlas_Adult_all | Mammary_Gland.Secretory_alveoli_cell_Ptpn18_high | 14459 | -0.00684 | 0.0662 | 0.54112 |
| DropViz_PC_level2 | Neuron_Slc17a7_Bcl6-C1ql2.3_3 | 14805 | -0.00103 | 0.00995 | 0.54128 |
| DropViz_FC_level2 | Neuron_Gad1Gad2_Synpr-Sncg.1_11 | 14805 | -7.90E-04 | 0.00762 | 0.54129 |
| DropViz_PC_level2 | Neuron_Sc17a7_Calb1-Fos.2_4 | 14805 | -0.00103 | 0.00987 | 0.5417 |
| DropViz_all_level2 | PC.Neuron_Gad1Gad2_Cplx3-Reln.4_1 | 14805 | -8.20E-04 | 0.00783 | 0.54173 |
| DropViz_all_level2 | SN.Oligodendrocyte_Tfr_Ndrg2.10_3 | 14805 | -5.80E-04 | 0.00552 | 0.54182 |
| MouseCellAtlas_Fetal_Brain | Purkinje_cell | 14459 | -0.0119 | 0.112 | 0.54217 |
| MouseCellAtlas_all | Small_Intestine.B_cell_Igkv12.46_high | 14459 | -0.00622 | 0.0586 | 0.5423 |
| DropViz_all_level2 | HC.Neuron_Slc17a7_Nxph3-Cplx3-Pappa2.3_9 | 14805 | -5.92E-04 | 0.00557 | 0.54235 |
| MouseCellAtlas_all | Mammary_Gland.B_cell_Jchain_high | 14459 | -0.00914 | 0.0856 | 0.5425 |
| Linnarsson_GSE104323_Mouse_Dentate_gyrus | MOL | 14909 | -0.00392 | 0.0367 | 0.54252 |
| DropViz_GP_level2 | Oligodendrocyte_Trf_Kif5b.10_6 | 14805 | -6.65E-04 | 0.00622 | 0.54253 |
| MouseCellAtlas_Neonatal_Skin | Macrophage_Pf4_high | 14459 | -0.00676 | 0.0632 | 0.54263 |
| MouseCellAtlas_Adult_all | Muscle.Granulocyte_monocyte_progenitor_cell | 14459 | -0.00633 | 0.0591 | 0.54269 |
| MouseCellAtlas_all | Stomach.Pit_cell_Gm26917_high | 14459 | -0.00473 | 0.0439 | 0.5429 |
| MouseCellAtlas_all | Ovary.Granulosa_cell_Inhba_high | 14459 | -0.00883 | 0.0813 | 0.54325 |
| MouseCellAtlas_Bone_Marrow | Bone_Marrow_c_kit.Multipotent_progenitor_Ctla2a_high | 14459 | -0.0131 | 0.12 | 0.54329 |
| GSE93374_Mouse_Arc_ME_level2 | Oligodendro4 | 13325 | -0.00516 | 0.0472 | 0.54352 |
| DropViz_FC_level2 | Neuron_Slc17a6-Slc17a7_Fezf2_3110035E14Rik.4_2 | 14805 | -8.00E-04 | 0.00731 | 0.54355 |
| Linnarsson_MouseBrainAtlas_level5 | TEGLU15 | 15140 | -0.0037 | 0.0338 | 0.54368 |
| DropViz_SN_level2 | Polydendrocyte_Tnr_Cspg5.6_1 | 14805 | -3.70E-04 | 0.00336 | 0.54389 |
| MouseCellAtlas_Embryo_all | Fetal_Stomache.Acinar_cell_Spp1_high | 14459 | -0.0137 | 0.124 | 0.54389 |
| DropViz_all_level2 | CB.Neuron_Gad1Gad2_Pvalb-Cadm1.3_3 | 14805 | -5.26E-04 | 0.00477 | 0.5439 |
| DropViz_FC_level2 | Neuron_Gad1Gad2_Synpr-Dpy19l1.1_7 | 14805 | -7.57E-04 | 0.00684 | 0.54409 |
| Linnarsson_GSE60361_Mouse_Cortex_Hippocampus_level2 | Int12 | 14010 | -0.0027 | 0.0244 | 0.54415 |
| MouseCellAtlas_Fetal_Stomache | Neutrophil | 14459 | -0.00892 | 0.0805 | 0.54415 |
| DropViz_TH_level2 | Neuron_Slc17a6_Rora_Fgf10.2_6 | 14805 | -8.54E-04 | 0.0077 | 0.54418 |
| DropViz_all_level2 | FC.Neuron_Slc17a7_Parm1_Nefm.7_6 | 14805 | -9.06E-04 | 0.00803 | 0.54489 |
| TabulaMuris_FACS_Heart | erythrocyte | 13888 | -0.00138 | 0.0122 | 0.54492 |
| MouseCellAtlas_all | Placenta.Macrophage_Spp1_high | 14459 | -0.00616 | 0.0544 | 0.54507 |
| MouseCellAtlas_all | Bone_Marrow.T_cell_Ms4a4b_high | 14459 | -0.0091 | 0.0799 | 0.54533 |
| MouseCellAtlas_Adult_all | Ovary.Cumulus_cell_Nupr1_high | 14459 | -0.0102 | 0.0891 | 0.54538 |
| Linnarsson_MouseBrainAtlas_level5 | MOL3 | 15140 | -0.00252 | 0.022 | 0.54559 |
| DropViz_CB_level1 | Mural | 14805 | -5.31E-04 | 0.00463 | 0.5457 |
| DropViz_all_level1 | TH.Mural | 14805 | -7.38E-04 | 0.0064 | 0.54588 |
| Linnarsson_GSE101601_Mouse_Somatosensory_cortex | Pyramidal_cells_layer_23_Npnt | 14550 | -0.00406 | 0.035 | 0.54613 |
| TabulaMuris_droplet_Bladder | basal_cell_of_urothelium | 13888 | -0.00424 | 0.0364 | 0.54639 |
| DropViz_all_level1 | GP.Oligodendrocyte | 14805 | -8.20E-04 | 0.00701 | 0.54654 |
| MouseCellAtlas_all | Muscle.Granulocyte_monocyte_progenitor_cell | 14459 | -0.00704 | 0.0602 | 0.54659 |
| GSE93374_Mouse_Arc_ME_neurons | Th_Slc6a3 | 13325 | -0.0364 | 0.309 | 0.54677 |
| DropViz_TH_level2 | Ependymal_Ccdc153.10_1 | 14805 | -5.21E-04 | 0.0044 | 0.54718 |
| TabulaMuris_FACS_Colon | enterocyte_of_epithelium_of_large_intestine | 13888 | -0.0011 | 0.00928 | 0.54728 |
| DropViz_all_level2 | PC.Neuron_Sc17a7_Calb1-Lpl-Cadm2.2_8 | 14805 | -6.25E-04 | 0.00521 | 0.54776 |
| MouseCellAtlas_Lung | Clara_Cell | 14459 | -0.00715 | 0.0596 | 0.54776 |
| MouseCellAtlas_all | Placenta.NKT_cell | 14459 | -0.00893 | 0.0743 | 0.54785 |
| MouseCellAtlas_Bladder | Umbrella_cell | 14459 | -0.0037 | 0.0306 | 0.54807 |
| DropViz_all_level2 | GP.Neuron_Gad1Gad2_Drd1-Pde1c.3_5 | 14805 | -8.36E-04 | 0.00689 | 0.54829 |
| MouseCellAtlas_Stomach | Gastric_mucosal_cell | 14459 | -0.0108 | 0.089 | 0.54835 |
| Allen_Mouse_ALM_level2 | Vip_Krt12 | 14744 | -0.00162 | 0.0133 | 0.54842 |
| MouseCellAtlas_Adult_all | Lung.Conventional_dendritic_cell_Tubb5_high | 14459 | -0.00771 | 0.063 | 0.54866 |
| GSE93374_Mouse_Arc_ME_level2 | Ependymocy2 | 13325 | -0.00617 | 0.0501 | 0.54902 |
| MouseCellAtlas_all | Stomach.G_cell | 14459 | -0.00816 | 0.0662 | 0.54904 |
| MouseCellAtlas_all | Brain.Microglia | 14459 | -0.00725 | 0.0585 | 0.54938 |
| DropViz_STR_level1 | Ependymal | 14805 | -6.51E-04 | 0.00521 | 0.5497 |
| Linnarsson_GSE60361_Mouse_Cortex_Hippocampus_level2 | S1PyrDL | 14010 | -0.00436 | 0.0348 | 0.54989 |
| MouseCellAtlas_Adult_all | Mammary_Gland.Macrophage_Cd74_high | 14459 | -0.015 | 0.119 | 0.55002 |
| MouseCellAtlas_Adult_all | Mammary_Gland.T.cells_Ctla4_high | 14459 | -0.0111 | 0.0881 | 0.55003 |
| Linnarsson_MouseBrainAtlas_level5 | CBINH2 | 15140 | -0.00434 | 0.0336 | 0.5513 |
| MouseCellAtlas_all | Small_Intestine.B_cell_Ms4a1_high | 14459 | -0.0102 | 0.0783 | 0.55192 |
| Linnarsson_MouseBrainAtlas_level5 | MBDOP1 | 15140 | -0.00718 | 0.055 | 0.55194 |
| MouseCellAtlas_Mammary_Gland | Mammary_Gland_Involution.Dendritic_cell_Il1b_high | 14459 | -0.00674 | 0.0516 | 0.55196 |
| MouseCellAtlas_Mammary_Gland | Mammary_Gland_Lactation.Secretory_alveoli_cell_Ccng1_high | 14459 | -0.0127 | 0.0966 | 0.55218 |
| Allen_Mouse_ALM_level2 | Vip_Sncg_3 | 14744 | -0.00107 | 0.00813 | 0.5525 |
| MouseCellAtlas_Mammary_Gland | Mammary_Gland_Lactation.Secretory_alveoli_cell_Igkv1.110_high | 14459 | -0.0108 | 0.0819 | 0.55264 |
| Allen_Mouse_VISp_level2 | Ndnf_Cxcl14 | 13868 | -0.00213 | 0.0161 | 0.55271 |
| GSE93374_Mouse_Arc_ME_neurons | Gpr50 | 13325 | -0.0325 | 0.245 | 0.55279 |
| DropViz_all_level1 | GP.Polydendrocyte | 14805 | -0.001 | 0.00747 | 0.55338 |
| DropViz_all_level2 | FC.Polydendrocyte_Tnr_Cspg5.10_1 | 14805 | -8.89E-04 | 0.00662 | 0.55343 |
| MouseCellAtlas_Adult_all | Mammary_Gland.Secretory_alveoli_cell_Sh3bgrl3_high | 14459 | -0.00851 | 0.0633 | 0.55345 |
| MouseCellAtlas_Adult_all | Kidney.Endothelial_cell | 14459 | -0.00709 | 0.0525 | 0.55366 |
| MouseCellAtlas_Adult_all | Ovary.Granulosa_cell_Inhba_high | 14459 | -0.0102 | 0.0754 | 0.55371 |
| DropViz_all_level1 | TH.Macrophage | 14805 | -5.64E-04 | 0.00412 | 0.55446 |
| Linnarsson_MouseBrainAtlas_level5 | VLMC2 | 15140 | -0.00434 | 0.0316 | 0.5545 |
| DropViz_TH_level2 | Neuron_Slc17a7_Tac2_Syt15.1_2 | 14805 | -8.32E-04 | 0.00606 | 0.55467 |
| MouseCellAtlas_Mammary_Gland | Mammary_Gland_Lactation.Secretory_alveoli_cell_Clu_high | 14459 | -0.0103 | 0.075 | 0.5547 |
| MouseCellAtlas_Adult_all | Lung.Eosinophil_granulocyte | 14459 | -0.00867 | 0.063 | 0.55475 |
| TabulaMuris_FACS_Marrow | monocyte | 13888 | -0.00163 | 0.0118 | 0.55507 |
| DropViz_all_level2 | GP.Neuron_Gad1Gad2_Cadm2.3_4 | 14805 | -8.11E-04 | 0.00585 | 0.55512 |
| MouseCellAtlas_Neonatal_all | Neonatal_Skin.Macrophage_Lyz2_high | 14459 | -0.00906 | 0.065 | 0.55541 |
| DropViz_all_level2 | PC.Neuron_Gad1Gad2_Synpr-Pcdh11x.4_9 | 14805 | -0.00104 | 0.0074 | 0.55589 |
| GSE93374_Mouse_Arc_ME_neurons | Pomc_Anxa2 | 13325 | -0.0315 | 0.224 | 0.55595 |
| DropViz_all_level1 | HC.Oligodendrocyte | 14805 | -9.52E-04 | 0.00676 | 0.55596 |
| Linnarsson_MouseBrainAtlas_level5 | SCINH6 | 15140 | -0.00546 | 0.0387 | 0.5561 |
| DropViz_all_level2 | TH.Polydendrocyte_Tnr_Bmp4.12_3 | 14805 | -7.45E-04 | 0.00528 | 0.55612 |
| MouseCellAtlas_Neonatal_all | Neonatal_Heart.Cardiac_muscle_cell | 14459 | -0.00875 | 0.062 | 0.55614 |
| MouseCellAtlas_Mammary_Gland | Mammary_Gland_Lactation.Secretory_alveoli_cell_Hsp90aa1_high | 14459 | -0.013 | 0.0915 | 0.5564 |
| MouseCellAtlas_Mammary_Gland | Mammary_Gland_Virgin.Luminal_cell_Krt19_high_ | 14459 | -0.00705 | 0.0495 | 0.55659 |
| GSE99235_Mouse_Lung_Vascular | cEC1 | 14559 | -0.00134 | 0.00942 | 0.55661 |
| DropViz_all_level2 | PC.Mural_Acta2Rgs5_Acta2.13_1 | 14805 | -6.74E-04 | 0.00472 | 0.55675 |
| GSE93374_Mouse_Arc_ME_level1 | Neurons3 | 13325 | -0.012 | 0.084 | 0.55681 |
| MouseCellAtlas_Adult_all | Small_Intestine.B_cell_Ms4a1_high | 14459 | -0.0105 | 0.0734 | 0.55684 |
| MouseCellAtlas_all | Mammary_Gland.T.cells_Ctla4_high | 14459 | -0.0128 | 0.0898 | 0.55687 |
| MouseCellAtlas_Adult_all | Bone_Marrow.Basophil | 14459 | -0.011 | 0.0766 | 0.557 |
| MouseCellAtlas_all | Neonatal_Heart.Macrophage | 14459 | -0.0116 | 0.0809 | 0.55718 |
| MouseCellAtlas_Adult_all | Mammary_Gland.Secretory_alveoli_cell_Pa2g4_high | 14459 | -0.0097 | 0.0673 | 0.55734 |
| Linnarsson_GSE101601_Mouse_Somatosensory_cortex | Pyramidal_cells_layer_4_Kcnab3 | 14550 | -0.00668 | 0.0462 | 0.55749 |
| Allen_Mouse_ALM_level2 | L4_Sparcl1 | 14744 | -9.00E-04 | 0.0062 | 0.55771 |
| GSE93374_Mouse_Arc_ME_level2 | Pars_Tuber1C | 13325 | -0.00929 | 0.0638 | 0.55785 |
| MouseCellAtlas_Neonatal_Skin | Lymphatic_vessel_endothelial_cell | 14459 | -0.0093 | 0.0637 | 0.55807 |
| DropViz_all_level2 | PC.Neuron_Slc17a7_Syt6-Slc24a2.1_6 | 14805 | -7.61E-04 | 0.00518 | 0.55835 |
| GSE93374_Mouse_Arc_ME_neurons | Sst_Pthlh | 13325 | -0.0556 | 0.378 | 0.55846 |
| Linnarsson_GSE101601_Mouse_Somatosensory_cortex | Microglia | 14550 | -0.0105 | 0.0716 | 0.55851 |
| MouseCellAtlas_Mammary_Gland | Mammary_Gland_Involution.Macrophage_Apoe_high | 14459 | -0.00729 | 0.0494 | 0.55865 |
| TabulaMuris_droplet_all | Spleen.B_cell | 13888 | -0.00706 | 0.0478 | 0.55878 |
| DropViz_all_level2 | SN.Neuron_Slc17a6_Tcf4.3_6 | 14805 | -9.92E-04 | 0.00662 | 0.5595 |
| MouseCellAtlas_all | Kidney.Endothelial_cell | 14459 | -0.0079 | 0.0525 | 0.55977 |
| MouseCellAtlas_Mammary_Gland | Mammary_Gland_Lactation.Secretory_alveoli_cell_Fth1_high | 14459 | -0.0128 | 0.0853 | 0.55987 |
| MouseCellAtlas_all | Lung.Conventional_dendritic_cell_Tubb5_high | 14459 | -0.0094 | 0.0624 | 0.5599 |
| DropViz_all_level2 | FC.Neuron_Slc17a7_Nptxr-Calb1.6_1 | 14805 | -0.00121 | 0.00795 | 0.5603 |
| DropViz_all_level2 | TH.Oligodendrocyte_Trf_Klk6.8_1 | 14805 | -8.06E-04 | 0.0053 | 0.56048 |
| MouseCellAtlas_Adult_all | Mammary_Gland.Secretory_alveoli_cell_Bckdha_high | 14459 | -0.0105 | 0.0686 | 0.56071 |
| DropViz_all_level2 | PC.Neuron_Sc17a7_Calb1-Lpl.2_5 | 14805 | -0.00122 | 0.00793 | 0.56095 |
| MouseCellAtlas_Neonatal_Skin | Adipocyte | 14459 | -0.0114 | 0.0741 | 0.56109 |
| MouseCellAtlas_all | Mammary_Gland.Secretory_alveoli_cell_Cd83_high | 14459 | -0.00941 | 0.0612 | 0.56113 |
| MouseCellAtlas_Adult_all | Mammary_Gland.Secretory_alveoli_cell_Naxd_high | 14459 | -0.0095 | 0.0617 | 0.56123 |
| MouseCellAtlas_Adult_all | Mammary_Gland.Secretory_alveoli_cell_Klf6_high | 14459 | -0.0105 | 0.0682 | 0.56133 |
| MouseCellAtlas_Lung | Stromal_cell_Acta2_high | 14459 | -0.0119 | 0.0767 | 0.5614 |
| Linnarsson_MouseBrainAtlas_level5 | PVM2 | 15140 | -0.00573 | 0.037 | 0.56141 |
| MouseCellAtlas_all | Lung.Eosinophil_granulocyte | 14459 | -0.00976 | 0.0628 | 0.56176 |
| DropViz_all_level2 | ENT.Neuron_Slc17a6_Pitx2.4_6 | 14805 | -0.00109 | 0.00702 | 0.56192 |
| MouseCellAtlas_all | Fetal_Stomache.Acinar_cell_Spp1_high | 14459 | -0.0142 | 0.0905 | 0.56222 |
| GSE99235_Mouse_Lung_Vascular | LEC | 14559 | -0.00176 | 0.0112 | 0.56253 |
| MouseCellAtlas_Adult_all | Thymus.gdT_cell_ | 14459 | -0.0115 | 0.0729 | 0.56253 |
| MouseCellAtlas_Small_Intestine | B_cell_Igkv12.46_high | 14459 | -0.0105 | 0.0662 | 0.5628 |
| MouseCellAtlas_all | Neonatal_Heart.Atrial_cardiomyocyte | 14459 | -0.00728 | 0.0459 | 0.56298 |
| MouseCellAtlas_all | Thymus.gdT_cell_ | 14459 | -0.0119 | 0.0747 | 0.56308 |
| MouseCellAtlas_all | Fetal_Brain.Radial_glia_Fabp7_high | 14459 | -0.0126 | 0.0793 | 0.56316 |
| MouseCellAtlas_all | Fetal_Stomache.Neutrophil | 14459 | -0.0119 | 0.0748 | 0.56323 |
| DropViz_SN_level1 | Astrocyte | 14805 | -9.98E-04 | 0.00619 | 0.56404 |
| DropViz_all_level2 | GP.Polydendrocyte_Tnr_Cspg5.4_5 | 14805 | -0.00101 | 0.00625 | 0.56431 |
| MouseCellAtlas_all | Bone_Marrow.Basophil | 14459 | -0.0125 | 0.0773 | 0.56443 |
| DropViz_all_level2 | TH.Neuron_Slc17a6_Rora_Nptxr.2_2 | 14805 | -0.00121 | 0.0074 | 0.56513 |
| DropViz_HC_level2 | Neuron_Gad1Gad2_Sst_Spon1.1_5 | 14805 | -0.00117 | 0.00714 | 0.56527 |
| Linnarsson_GSE101601_Mouse_Somatosensory_cortex | Mature_oligodendrocyte | 14550 | -0.00952 | 0.0579 | 0.56535 |
| DropViz_all_level2 | TH.Neuron_Slc17a7_Tac2_Wif1.1_3 | 14805 | -0.00103 | 0.00626 | 0.5654 |
| TabulaMuris_droplet_all | Muscle.macrophage | 13888 | -0.00849 | 0.0513 | 0.56569 |
| DropViz_PC_level2 | Neuron_Slc17a7_Syt6-Fos.1_9 | 14805 | -0.0017 | 0.0102 | 0.56589 |
| MouseCellAtlas_Mammary_Gland | Mammary_Gland_Lactation.Secretory_alveoli_cell_Dnajb1_high | 14459 | -0.016 | 0.0964 | 0.566 |
| MouseCellAtlas_Adult_all | Mammary_Gland.Secretory_alveoli_cell_Ifi35_high | 14459 | -0.011 | 0.0664 | 0.56609 |
| DropViz_all_level2 | HC.Neuron_Gad1Gad2_Sst_Atp2b4.1_3 | 14805 | -7.62E-04 | 0.00454 | 0.56658 |
| MouseCellAtlas_Adult_all | Mammary_Gland.Secretory_alveoli_cell_Gm1673_high | 14459 | -0.0114 | 0.0678 | 0.5669 |
| MouseCellAtlas_all | Ovary.luteal_cells | 14459 | -0.00701 | 0.0416 | 0.5669 |
| MouseCellAtlas_Bone_Marrow | Bone_Marrow_c_kit.Eosinophil_progenitor_cell | 14459 | -0.0116 | 0.0689 | 0.56692 |
| Linnarsson_GSE60361_Mouse_Cortex_Hippocampus_level2 | S1PyrL4 | 14010 | -0.00424 | 0.0251 | 0.56704 |
| DroNc_Mouse_Hippocampus | exPFC4 | 12425 | -0.0424 | 0.25 | 0.56744 |
| DropViz_ENT_level2 | Oligodendrocyte_Trf_Serpinb1a.1_1 | 14805 | -0.00113 | 0.00665 | 0.56749 |
| DropViz_FC_level2 | Oligodendrocyte_Tnf_Klk6.9_5 | 14805 | -9.74E-04 | 0.0057 | 0.56778 |
| GSE93374_Mouse_Arc_ME_neurons | Gm8773_Tac1 | 13325 | -0.039 | 0.227 | 0.56839 |
| MouseCellAtlas_Adult_all | Spleen.T_cell | 14459 | -0.0162 | 0.093 | 0.56921 |
| GSE93374_Mouse_Arc_ME_level2 | Ependymocy1 | 13325 | -0.00514 | 0.0293 | 0.56959 |
| MouseCellAtlas_Neonatal_Skin | Neutrophil | 14459 | -0.00988 | 0.056 | 0.57 |
| TabulaMuris_FACS_all | Aorta.hematopoietic_cell | 13888 | -0.00138 | 0.00775 | 0.57045 |
| DropViz_all_level2 | TH.Neuron_Slc17a6_Rora_Cacng4.2_10 | 14805 | -0.00123 | 0.00688 | 0.57068 |
| GSE87544_Mouse_Hypothalamus | Glu2 | 13878 | -0.00737 | 0.0413 | 0.57077 |
| MouseCellAtlas_Adult_all | Ovary.luteal_cells | 14459 | -0.00705 | 0.0394 | 0.57109 |
| MouseCellAtlas_all | Placenta.Spongiotrophoblast_Hsd11b2_high | 14459 | -0.00922 | 0.0515 | 0.5711 |
| MouseCellAtlas_Embryo_all | Fetal_Intestine.Acinar_cell | 14459 | -0.0142 | 0.079 | 0.57138 |
| DropViz_HC_level2 | Astrocyte_Gja1_Nnat.7_1 | 14805 | -9.93E-04 | 0.00549 | 0.57173 |
| MouseCellAtlas_all | Fetal_Brain.Purkinje_cell | 14459 | -0.0108 | 0.06 | 0.57177 |
| MouseCellAtlas_Testis | Spermatogonia_1700001P01Rik_high | 14459 | -0.0273 | 0.151 | 0.57183 |
| DropViz_all_level2 | PC.Neuron_Slc17a7_Bcl6-Npnt.3_10 | 14805 | -0.00134 | 0.00738 | 0.57196 |
| MouseCellAtlas_Testis | Spermatocyte_1700001F09Rik_high | 14459 | -0.00926 | 0.051 | 0.57196 |
| GSE92332_Mouse_Epithelium_droplet | TA_G2 | 11993 | -0.00987 | 0.0542 | 0.57224 |
| Allen_Mouse_ALM_level1 | L2_3 | 14744 | -0.00184 | 0.0101 | 0.57229 |
| Linnarsson_MouseBrainAtlas_level5 | VSMCA | 15140 | -0.00749 | 0.0411 | 0.57235 |
| GSE106678_Mouse_Cortex | Ex10 | 14573 | -0.0219 | 0.12 | 0.57244 |
| DropViz_all_level2 | FC.Neuron_Gad1Gad2_Synpr-Sncg.1_11 | 14805 | -0.00132 | 0.0072 | 0.5725 |
| MouseCellAtlas_all | Mammary_Gland.Secretory_alveoli_cell_Ptpn18_high | 14459 | -0.0117 | 0.064 | 0.57253 |
| Linnarsson_GSE95752_Mouse_Dentate_gyrus | PVM | 12517 | -0.00838 | 0.0458 | 0.57256 |
| MouseCellAtlas_Mammary_Gland | Mammary_Gland_Lactation.Secretory_alveoli_cell_Cgrrf1_high | 14459 | -0.0188 | 0.102 | 0.57277 |
| DropViz_all_level2 | FC.Neuron_Gad1Gad2_Synpr-Dpy19l1.1_7 | 14805 | -0.00119 | 0.00647 | 0.57317 |
| MouseCellAtlas_Adult_all | Mammary_Gland.Secretory_alveoli_cell_Ly6a_high | 14459 | -0.0113 | 0.0611 | 0.57346 |
| Linnarsson_GSE95752_Mouse_Dentate_gyrus | Radial_Glia_like | 12517 | -0.00893 | 0.048 | 0.57379 |
| DropViz_FC_level1 | Mural | 14805 | -0.00131 | 0.007 | 0.57446 |
| DropViz_all_level2 | FC.Neuron_Slc17a6-Slc17a7_Fezf2_3110035E14Rik.4_2 | 14805 | -0.00122 | 0.00645 | 0.57479 |
| MouseCellAtlas_Bone_Marrow | Bone_Marrow.Neutrophil_Cebpe_high | 14459 | -0.00754 | 0.0399 | 0.57483 |
| MouseCellAtlas_Neonatal_Rib | Neutrophil_Elane_high | 14459 | -0.0112 | 0.0593 | 0.57484 |
| DropViz_all_level2 | HC.Mural_Rgs5Acta2_Kcnj8.16_3 | 14805 | -7.54E-04 | 0.00399 | 0.5749 |
| MouseCellAtlas_Mammary_Gland | Mammary_Gland_Lactation.Secretory_alveoli_cell_Actb_high | 14459 | -0.0173 | 0.0916 | 0.57499 |
| TabulaMuris_FACS_Marrow | B_cell | 13888 | -0.00235 | 0.0124 | 0.57523 |
| MouseCellAtlas_Embryo_all | Fetal_Stomache.Neutrophil | 14459 | -0.015 | 0.0784 | 0.57591 |
| GSE99235_Mouse_Lung_Vascular | aEC | 14559 | -0.00156 | 0.00809 | 0.57629 |
| MouseCellAtlas_Pancreas | Endothelial_cell_Lrg1_high | 14459 | -0.0137 | 0.0708 | 0.57654 |
| DropViz_all_level2 | GP.Neuron_Gad1Gad2_Trdn.2_16 | 14805 | -0.00132 | 0.00682 | 0.57661 |
| DropViz_all_level2 | GP.Neuron_Gad1Gad2_Drd1-Pdyn.3_2 | 14805 | -0.00132 | 0.00682 | 0.57676 |
| Linnarsson_GSE74672_Mouse_Hypothalamus_Neurons_level2 | GABA_15_Npymedium | 14550 | -0.00765 | 0.0395 | 0.57679 |
| TabulaMuris_FACS_Marrow | granulocyte | 13888 | -0.00158 | 0.00813 | 0.57715 |
| Linnarsson_GSE60361_Mouse_Cortex_Hippocampus_level2 | CA2Pyr2 | 14010 | -0.00432 | 0.0221 | 0.57732 |
| TabulaMuris_FACS_all | Skin.epidermal_cell | 13888 | -0.0014 | 0.00716 | 0.57738 |
| MouseCellAtlas_all | Uterus.Muscle_cell_Pcp4_high | 14459 | -0.0114 | 0.0579 | 0.57799 |
| MouseCellAtlas_Mammary_Gland | Mammary_Gland_Lactation.Secretory_alveoli_cell_Cfd_high | 14459 | -0.0187 | 0.095 | 0.57811 |
| DropViz_CB_level2 | Neuron_Gad1Gad2_Pvalb-Cadm1.3_3 | 14805 | -8.88E-04 | 0.00451 | 0.57813 |
| MouseCellAtlas_Adult_all | Mammary_Gland.Secretory_alveoli_cell_Tmem123_high | 14459 | -0.0124 | 0.0624 | 0.57853 |
| MouseCellAtlas_Neonatal_all | Neonatal_Rib.Neuron_Stmn2_high | 14459 | -0.0114 | 0.0572 | 0.57873 |
| DropViz_all_level2 | TH.Neuron_Slc17a6_Rora_Nrp2.2_4 | 14805 | -0.00143 | 0.00715 | 0.57901 |
| MouseCellAtlas_Embryo_all | Placenta.Spongiotrophoblast_Hsd11b2_high | 14459 | -0.011 | 0.0548 | 0.57957 |
| DropViz_GP_level1 | Macrophage | 14805 | -9.47E-04 | 0.00471 | 0.57967 |
| Linnarsson_MouseBrainAtlas_level5 | OPC | 15140 | -0.00972 | 0.0483 | 0.57978 |
| MouseCellAtlas_all | Spleen.T_cell | 14459 | -0.0187 | 0.0928 | 0.57978 |
| DropViz_ENT_level2 | Astrocyte_Gja1_Htra1.7_2 | 14805 | -0.00108 | 0.00535 | 0.57981 |
| MouseCellAtlas_all | Placenta.Invasive_spongiotrophoblast | 14459 | -0.0132 | 0.0655 | 0.57999 |
| TabulaMuris_droplet_Trachea | epithelial_cell | 13888 | -0.0132 | 0.0653 | 0.5803 |
| MouseCellAtlas_Adult_all | Testis.Spermatocyte_Cabs1_high | 14459 | -0.00936 | 0.046 | 0.58057 |
| DropViz_ENT_level2 | Mural_Rgs5Acta2_Ccnd1.3_4 | 14805 | -8.48E-04 | 0.00416 | 0.58078 |
| Allen_Mouse_VISp_level1 | Gluta_L6b | 13868 | -0.00244 | 0.012 | 0.58088 |
| Linnarsson_MouseBrainAtlas_level6_rank2 | PNS_neurons | 15140 | -0.00514 | 0.0252 | 0.58088 |
| Linnarsson_MouseBrainAtlas_level5 | MGL3 | 15140 | -0.00675 | 0.033 | 0.58108 |
| DropViz_all_level2 | TH.Neuron_Slc17a6_Rora_Cbln2.2_8 | 14805 | -0.0014 | 0.00684 | 0.5813 |
| MouseCellAtlas_Mammary_Gland | Mammary_Gland_Pregnancy.Dendritic_cell_Cd209a_high | 14459 | -0.00986 | 0.0478 | 0.58165 |
| Linnarsson_MouseBrainAtlas_level5 | DEINH7 | 15140 | -0.0133 | 0.0646 | 0.58174 |
| MouseCellAtlas_Muscle | Dendritic_cell | 14459 | -0.0128 | 0.0619 | 0.58175 |
| DropViz_FC_level2 | Neuron_Slc17a7_Syt6-Slc30a3.3_5 | 14805 | -0.0019 | 0.00922 | 0.5818 |
| DropViz_all_level2 | TH.Neuron_Slc17a6_Rora_Pvalb.2_11 | 14805 | -0.00139 | 0.00673 | 0.58183 |
| GSE106678_Mouse_Cortex | Ex22 | 14573 | -0.034 | 0.164 | 0.58201 |
| Linnarsson_MouseBrainAtlas_level6_rank4 | Gopc | 15140 | -0.0113 | 0.0542 | 0.58216 |
| MouseCellAtlas_Bone_Marrow | Bone_Marrow_c_kit.Macrophage_S100a4_high | 14459 | -0.0134 | 0.0644 | 0.58243 |
| TabulaMuris_FACS_all | Tongue.basal_cell_of_epidermis | 13888 | -0.00136 | 0.00654 | 0.58254 |
| DropViz_ENT_level2 | Mural_Rgs5Acta2_Pdgfa.3_3 | 14805 | -6.62E-04 | 0.00316 | 0.58302 |
| DropViz_all_level1 | HC.Macrophage | 14805 | -8.19E-04 | 0.00389 | 0.58334 |
| MouseCellAtlas_all | Mammary_Gland.Secretory_alveoli_cell_Sh3bgrl3_high | 14459 | -0.013 | 0.0615 | 0.58354 |
| MouseCellAtlas_all | Fetal_Intestine.Acinar_cell | 14459 | -0.0132 | 0.0627 | 0.58356 |
| Linnarsson_GSE59739_Mouse_Dorsal_root_ganglion_level3 | Peptidergic_nociceptor1 | 13848 | -0.00299 | 0.0141 | 0.58404 |
| MouseCellAtlas_Adult_all | Mammary_Gland.Secretory_alveoli_cell_Wfdc17_high | 14459 | -0.0151 | 0.071 | 0.58427 |
| MouseCellAtlas_Lung | Alveolar_macrophage_Ear2_high | 14459 | -0.0193 | 0.0905 | 0.58432 |
| MouseCellAtlas_all | Mammary_Gland.Macrophage_Cd74_high | 14459 | -0.024 | 0.112 | 0.58447 |
| GSE87544_Mouse_Hypothalamus | Glu4 | 13878 | -0.011 | 0.0514 | 0.58492 |
| MouseCellAtlas_Adult_all | Mammary_Gland.Secretory_alveoli_cell_Lgals1_high | 14459 | -0.0136 | 0.0633 | 0.58498 |
| MouseCellAtlas_Adult_all | Bladder.Macrophage_Pf4_high | 14459 | -0.0111 | 0.0519 | 0.585 |
| MouseCellAtlas_all | Fetal_Lung.Neutrophil_S100a8_high | 14459 | -0.0188 | 0.0876 | 0.58501 |
| Linnarsson_GSE104323_Mouse_Dentate_gyrus | Immature_GABA | 14909 | -0.00912 | 0.0424 | 0.58507 |
| MouseCellAtlas_all | Testis.Spermatocyte_Cabs1_high | 14459 | -0.00988 | 0.0459 | 0.58514 |
| MouseCellAtlas_Adult_all | Uterus.Muscle_cell_Pcp4_high | 14459 | -0.0121 | 0.0564 | 0.58524 |
| MouseCellAtlas_Trophoblast_Stem_Cell | TS_Rps28_high | 14459 | -0.0279 | 0.13 | 0.58525 |
| DropViz_GP_level2 | Neuron_Gad1Gad2_Tac2.2_3 | 14805 | -0.00173 | 0.00801 | 0.58564 |
| MouseCellAtlas_Mammary_Gland | Mammary_Gland_Lactation.Secretory_alveoli_cell_Srsf11_high | 14459 | -0.021 | 0.0971 | 0.58579 |
| GSE93374_Mouse_Arc_ME_level1 | ParsTuber2 | 13325 | -0.019 | 0.0874 | 0.58584 |
| TabulaMuris_FACS_all | Pancreas.type_B_pancreatic_cell | 13888 | -0.00118 | 0.00543 | 0.58598 |
| MouseCellAtlas_all | Bone_Marrow.Megakaryocyte | 14459 | -0.0107 | 0.049 | 0.58611 |
| GSE104276_Human_Prefrontal_cortex_all_ages | Stem_cells | 17199 | -0.00278 | 0.0128 | 0.58639 |
| Allen_Mouse_VISp_level2 | L4_Scnn1a | 13868 | -0.00318 | 0.0146 | 0.58655 |
| DropViz_HC_level2 | Neuron_Gad1Gad2_Id2_Sfrp2.1_2 | 14805 | -0.00128 | 0.00583 | 0.58692 |
| MouseCellAtlas_all | Mammary_Gland.Secretory_alveoli_cell_Pa2g4_high | 14459 | -0.0144 | 0.065 | 0.58779 |
| Linnarsson_GSE76381_Mouse_Midbrain | NbML3 | 14550 | -0.0119 | 0.0532 | 0.58821 |
| DropViz_all_level2 | FC.Oligodendrocyte_Tnf_Klk6.9_5 | 14805 | -0.00138 | 0.00617 | 0.58842 |
| DropViz_all_level2 | GP.Neuron_Gad1Gad2_Scn4b.2_14 | 14805 | -0.0015 | 0.00671 | 0.58863 |
| MouseCellAtlas_Embryonic_Mesenchyme | Muscle | 14459 | -0.0311 | 0.138 | 0.58909 |
| MouseCellAtlas_Adult_all | Testis.Spermatids_Tnp1_high | 14459 | -0.00986 | 0.0436 | 0.5895 |
| Linnarsson_MouseBrainAtlas_level5 | HBSER1 | 15140 | -0.00704 | 0.0311 | 0.58959 |
| MouseCellAtlas_all | Mammary_Gland.Secretory_alveoli_cell_Naxd_high | 14459 | -0.0136 | 0.0601 | 0.58965 |
| MouseCellAtlas_Kidney | Distal_collecting_duct_principal_cell_Hsd11b2_high | 14459 | -0.0203 | 0.0895 | 0.58989 |
| TabulaMuris_droplet_all | Tongue.basal_cell_of_epidermis | 13888 | -0.00587 | 0.0258 | 0.58998 |
| TabulaMuris_FACS_all | Fat.epithelial_cell | 13888 | -0.00184 | 0.00805 | 0.59039 |
| MouseCellAtlas_Embryonic_Mesenchyme | Dendritic_Cell | 14459 | -0.0234 | 0.102 | 0.59046 |
| MouseCellAtlas_Adult_all | Bone_Marrow.Megakaryocyte | 14459 | -0.0107 | 0.0468 | 0.5905 |
| MouseCellAtlas_Mammary_Gland | Mammary_Gland_Lactation.Adiocyte_cell | 14459 | -0.0328 | 0.143 | 0.59075 |
| DropViz_all_level2 | PC.Neuron_Slc17a7-Slc17a6_Calb1-Rorb-Il1rapl2.2_13 | 14805 | -0.00194 | 0.00844 | 0.59085 |
| DropViz_FC_level2 | Neuron_Gad1Gad2_Cplx3.1_1 | 14805 | -0.00202 | 0.00878 | 0.59092 |
| MouseCellAtlas_Mammary_Gland | Mammary_Gland_Lactation.Secretory_alveoli_cell_Igkv1.117_high | 14459 | -0.0201 | 0.0871 | 0.59123 |
| MouseCellAtlas_all | Mammary_Gland.Secretory_alveoli_cell_Klf6_high | 14459 | -0.0153 | 0.0659 | 0.59187 |
| MouseCellAtlas_all | Mammary_Gland.Secretory_alveoli_cell_Bckdha_high | 14459 | -0.0154 | 0.0662 | 0.59193 |
| Linnarsson_GSE60361_Mouse_Cortex_Hippocampus_level2 | Mgl2 | 14010 | -0.00776 | 0.0332 | 0.59233 |
| DropViz_TH_level2 | Neuron_Slc17a7_Tac2_Chat.1_1 | 14805 | -0.00136 | 0.0058 | 0.59243 |
| MouseCellAtlas_Spleen | Macrophage | 14459 | -0.0155 | 0.0664 | 0.59251 |
| MouseCellAtlas_Bone_Marrow | Bone_Marrow.Erythroblast | 14459 | -0.00855 | 0.0365 | 0.59259 |
| MouseCellAtlas_Stomach | Pit_cell_Ifrd1_high | 14459 | -0.0229 | 0.0975 | 0.59281 |
| DropViz_ENT_level2 | Neuron_Gad1Gad2-Chat.4_12 | 14805 | -7.09E-04 | 0.00302 | 0.5929 |
| TabulaMuris_droplet_all | Mammary.B_cell | 13888 | -0.0109 | 0.0463 | 0.59319 |
| MouseCellAtlas_Embryo_all | Fetal_Liver.Megakaryocyte | 14459 | -0.0169 | 0.0712 | 0.59368 |
| MouseCellAtlas_all | Testis.Spermatids_Tnp1_high | 14459 | -0.0103 | 0.0435 | 0.59369 |
| DropViz_FC_level2 | Neuron_Gad1Gad2-Chat_Synpr-Slc5a7.1_5 | 14805 | -0.00202 | 0.00853 | 0.59377 |
| MouseCellAtlas_Prostate | Epithelial_cell | 14459 | -0.0515 | 0.217 | 0.594 |
| DropViz_all_level2 | STR.Neurogenesis_Sox4_Stmn2-Csrp2.2_4 | 14805 | -8.74E-04 | 0.00367 | 0.59405 |
| DropViz_SN_level2 | Neuron_Th_Aldh1a1.4_8 | 14805 | -0.00159 | 0.00668 | 0.59416 |
| MouseCellAtlas_Adult_all | Mammary_Gland.Secretory_alveoli_cell_Mat2a_high | 14459 | -0.0161 | 0.0673 | 0.59443 |
| DropViz_FC_level1 | Oligodendrocyte | 14805 | -0.00171 | 0.00715 | 0.5946 |
| TabulaMuris_droplet_Lung | natural_killer_cell | 13888 | -0.0129 | 0.0541 | 0.5946 |
| Allen_Mouse_ALM_level2 | Meis2 | 14744 | -0.00123 | 0.00511 | 0.59479 |
| Linnarsson_MouseBrainAtlas_level5 | HBINH4 | 15140 | -0.0112 | 0.0466 | 0.59481 |
| MouseCellAtlas_all | Mammary_Gland.Secretory_alveoli_cell_Ifi35_high | 14459 | -0.0155 | 0.0642 | 0.59513 |
| DropViz_all_level2 | GP.Mural_Rgs5Acta2_Acta2.8_1 | 14805 | -0.00111 | 0.00458 | 0.59552 |
| DropViz_ENT_level1 | Polydendrocyte | 14805 | -0.00178 | 0.00734 | 0.59561 |
| MouseCellAtlas_all | Mammary_Gland.Secretory_alveoli_cell_Gm1673_high | 14459 | -0.0159 | 0.0657 | 0.59577 |
| DropViz_SN_level2 | Neuron_Gad1Gad2_Rln3.3_5 | 14805 | -0.00104 | 0.00427 | 0.59597 |
| DropViz_all_level2 | PC.Neuron_Sc17a7_Crym.2_16 | 14805 | -0.00195 | 0.00801 | 0.59611 |
| MouseCellAtlas_Adult_all | Mammary_Gland.Secretory_alveoli_cell_Cidea_high | 14459 | -0.0167 | 0.0685 | 0.5962 |
| DropViz_SN_level2 | Neuron_Th_Grin2c.4_9 | 14805 | -0.00166 | 0.0068 | 0.59625 |
| MouseCellAtlas_Embryo_all | Placenta.Basophil | 14459 | -0.0159 | 0.0652 | 0.59629 |
| MouseCellAtlas_Neonatal_Calvaria | Mast_cell | 14459 | -0.0222 | 0.091 | 0.59649 |
| DropViz_HC_level1 | Mural | 14805 | -0.00147 | 0.00602 | 0.59662 |
| MouseCellAtlas_Mammary_Gland | Mammary_Gland_Virgin.T_cell_Cd8b1_high | 14459 | -0.0154 | 0.0628 | 0.59666 |
| Linnarsson_GSE74672_Mouse_Hypothalamus_Neurons_level2 | Trh_3_high | 14550 | -0.00829 | 0.0338 | 0.5969 |
| MouseCellAtlas_all | Embryonic_Mesenchyme.Lymphoid_progenitor_cell | 14459 | -0.0189 | 0.0769 | 0.59704 |
| MouseCellAtlas_Neonatal_all | Neonatal_Muscle.Neutrophil_Hmox1_high | 14459 | -0.013 | 0.0527 | 0.59706 |
| DropViz_all_level2 | TH.Neuron_Gad1Gad2_Ttn.3_11 | 14805 | -0.00136 | 0.00552 | 0.59737 |
| MouseCellAtlas_Small_Intestine | Erythroblast | 14459 | -0.0151 | 0.0608 | 0.59793 |
| MouseCellAtlas_all | Fetal_Intestine.Erythroblast_Hbb.bs_high | 14459 | -0.0249 | 0.1 | 0.59801 |
| MouseCellAtlas_Embryo_all | Fetal_Intestine.Erythroblast_Hbb.bs_high | 14459 | -0.0352 | 0.141 | 0.59824 |
| GSE104276_Human_Prefrontal_cortex_all_ages | OPC | 17199 | -0.00447 | 0.018 | 0.59838 |
| GSE93374_Mouse_Arc_ME_neurons | Ghrh | 13325 | -0.0622 | 0.249 | 0.59862 |
| DropViz_all_level2 | HC.Polydendrocyte_Tnr_Bmp4.9_2 | 14805 | -0.00132 | 0.00529 | 0.59876 |
| DropViz_FC_level2 | Polydendrocyte_Tnr_Bmp4-Gpr17.10_6 | 14805 | -0.00135 | 0.00539 | 0.59884 |
| DropViz_all_level2 | PC.Neuron_Sc17a7_Calb1-Fos.2_4 | 14805 | -0.00184 | 0.00734 | 0.59887 |
| Linnarsson_MouseBrainAtlas_level5 | TEGLU24 | 15140 | -0.01 | 0.0398 | 0.59957 |
| DropViz_STR_level1 | Neurogenesis | 14805 | -0.00146 | 0.00578 | 0.5996 |
| DropViz_all_level2 | PC.Neuron_Slc17a7_Bcl6-C1ql2.3_3 | 14805 | -0.00192 | 0.00759 | 0.59968 |
| MouseCellAtlas_all | Neonatal_Rib.Muscle_cell | 14459 | -0.0179 | 0.0708 | 0.59988 |
| MouseCellAtlas_Lung | Conventional_dendritic_cell_Mgl2_high | 14459 | -0.0219 | 0.0864 | 0.60006 |
| MouseCellAtlas_all | Mammary_Gland.Secretory_alveoli_cell_Ly6a_high | 14459 | -0.0151 | 0.0596 | 0.60021 |
| MouseCellAtlas_all | Fetal_Liver.Megakaryocyte | 14459 | -0.0158 | 0.0624 | 0.60026 |
| MouseCellAtlas_all | Neonatal_Muscle.Brown_adipose_tissue | 14459 | -0.00999 | 0.0393 | 0.6004 |
| MouseCellAtlas_Mammary_Gland | Mammary_Gland_Lactation.Secretory_alveoli_cell_Taf1d_high | 14459 | -0.021 | 0.0822 | 0.60063 |
| MouseCellAtlas_Mammary_Gland | Mammary_Gland_Lactation.Secretory_alveoli_cell_Tubb5_high | 14459 | -0.0225 | 0.0879 | 0.60076 |
| MouseCellAtlas_Adult_all | Mammary_Gland.Secretory_alveoli_cell_Cyb561_high | 14459 | -0.0146 | 0.0571 | 0.6009 |
| GSE104276_Human_Prefrontal_cortex_per_ages | GW23_Stem_cells | 17199 | -0.0011 | 0.00427 | 0.60153 |
| DropViz_PC_level2 | Neuron_Gad1Gad2-Chat_Synpr-Slc5a7.4_8 | 14805 | -0.00209 | 0.00812 | 0.60159 |
| DropViz_HC_level1 | Polydendrocyte | 14805 | -0.00189 | 0.00732 | 0.60174 |
| GSE93374_Mouse_Arc_ME_neurons | Agrp_Gm8773 | 13325 | -0.0627 | 0.243 | 0.60184 |
| TabulaMuris_FACS_Fat | epithelial_cell | 13888 | -0.00186 | 0.00716 | 0.60229 |
| MouseCellAtlas_Muscle | Neutrophil_Camp_high | 14459 | -0.0149 | 0.0571 | 0.60277 |
| TabulaMuris_FACS_Diaphragm | skeletal_muscle_satellite_stem_cell | 13888 | -0.0033 | 0.0126 | 0.60303 |
| MouseCellAtlas_Placenta | Basophil | 14459 | -0.0198 | 0.0757 | 0.60324 |
| DropViz_FC_level2 | Neuron_Slc17a7_Parm1_Fos.7_4 | 14805 | -0.00114 | 0.00435 | 0.60335 |
| DropViz_all_level2 | GP.Neuron_Gad1Gad2-Th_Adora2a-Th.3_9 | 14805 | -0.00195 | 0.00742 | 0.60341 |
| MouseCellAtlas_all | Bladder.Macrophage_Pf4_high | 14459 | -0.0133 | 0.0505 | 0.60353 |
| MouseCellAtlas_Embryo_all | Placenta.Macrophage_Spp1_high | 14459 | -0.0129 | 0.0492 | 0.60371 |
| MouseCellAtlas_Mammary_Gland | Mammary_Gland_Lactation.Macrophage_Cd74_high | 14459 | -0.0383 | 0.145 | 0.60383 |
| MouseCellAtlas_Adult_all | Mammary_Gland.Secretory_alveoli_cell_Sars_high | 14459 | -0.0171 | 0.0647 | 0.60428 |
| MouseCellAtlas_all | Mammary_Gland.Secretory_alveoli_cell_Tmem123_high | 14459 | -0.0162 | 0.061 | 0.60455 |
| MouseCellAtlas_Adult_all | Small_Intestine.Erythroblast | 14459 | -0.0147 | 0.0551 | 0.60502 |
| Linnarsson_GSE67602_Mouse_Skin_Epidermis | IB | 14528 | -0.0152 | 0.0569 | 0.60513 |
| MouseCellAtlas_all | Placenta.Spongiotrophoblast_Phlda2_high | 14459 | -0.0175 | 0.0654 | 0.60552 |
| MouseCellAtlas_Pancreas | Macrophage | 14459 | -0.0242 | 0.0904 | 0.60562 |
| MouseCellAtlas_Adult_all | Mammary_Gland.Secretory_alveoli_cell_Ccng1_high | 14459 | -0.0169 | 0.0631 | 0.60587 |
| Allen_Mouse_ALM_level2 | L5_ALM_Pld5_1 | 14744 | -0.00306 | 0.0113 | 0.60645 |
| MouseCellAtlas_Embryo_all | Placenta.Invasive_spongiotrophoblast | 14459 | -0.0174 | 0.0644 | 0.60659 |
| Linnarsson_GSE95315_Mouse_Dentate_gyrus | PVM | 11815 | -0.0108 | 0.0401 | 0.60675 |
| MouseCellAtlas_all | Ovary.Small_luteal_cell | 14459 | -0.0265 | 0.0977 | 0.60683 |
| DroNc_Human_Hippocampus | ASC1 | 16914 | -0.0529 | 0.194 | 0.60725 |
| MouseCellAtlas_Mammary_Gland | Mammary_Gland_Lactation.Secretory_alveoli_cell_Kif5b_high | 14459 | -0.0272 | 0.0999 | 0.60749 |
| DropViz_all_level2 | GP.Neuron_Gad1Gad2_Sphkap-Cpne4.3_8 | 14805 | -0.00169 | 0.0062 | 0.6076 |
| DropViz_all_level2 | HC.Gad1Gad2_Htr3a_Efba5.1_19 | 14805 | -0.00158 | 0.0058 | 0.60763 |
| MouseCellAtlas_Embryo_all | Fetal_Lung.Neutrophil_S100a8_high | 14459 | -0.0296 | 0.108 | 0.60769 |
| DropViz_FC_level2 | Neuron_Slc17a7_Syt6-Slc24a2.3_6 | 14805 | -0.00185 | 0.00678 | 0.60776 |
| MouseCellAtlas_all | Embryonic_Mesenchyme.Progenitor_cell_Ptprcap_high | 14459 | -0.0179 | 0.0655 | 0.60782 |
| MouseCellAtlas_Embryo_all | Placenta.Spongiotrophoblast_Phlda2_high | 14459 | -0.0208 | 0.0761 | 0.60787 |
| MouseCellAtlas_Neonatal_Rib | Macrophage_C1qc_high | 14459 | -0.0179 | 0.0651 | 0.60806 |
| MouseCellAtlas_Mammary_Gland | Mammary_Gland_Virgin.B_cell_Cd79a.Iglc2_high | 14459 | -0.0185 | 0.0673 | 0.60808 |
| Allen_Mouse_ALM_level2 | Vip_Gpc3_1 | 14744 | -0.00144 | 0.00524 | 0.60818 |
| MouseCellAtlas_Placenta | Macrophage_Spp1_high | 14459 | -0.0167 | 0.0609 | 0.60825 |
| TabulaMuris_FACS_all | Brain_Neurons.oligodendrocyte_precursor_cell | 13888 | -0.00158 | 0.00573 | 0.60838 |
| Linnarsson_GSE101601_Mouse_Somatosensory_cortex | Mature_oligodendrocyte_apod | 14550 | -0.0116 | 0.0422 | 0.60855 |
| Linnarsson_MouseBrainAtlas_level5 | HBGLU7 | 15140 | -0.00713 | 0.0258 | 0.60882 |
| Linnarsson_GSE74672_Mouse_Hypothalamus_Neurons_level2 | Avp_1_high | 14550 | -0.00935 | 0.0338 | 0.60886 |
| TabulaMuris_FACS_all | Lung.Clara_cell | 13888 | -0.00224 | 0.00811 | 0.60897 |
| MouseCellAtlas_all | Small_Intestine.Erythroblast | 14459 | -0.0153 | 0.055 | 0.60917 |
| MouseCellAtlas_Neonatal_all | Neonatal_Calvaria.Muscle_cell_Actc1_high | 14459 | -0.0216 | 0.078 | 0.60922 |
| MouseCellAtlas_Embryo_all | Embryonic_Mesenchyme.Progenitor_cell_Ptprcap_high | 14459 | -0.0213 | 0.0767 | 0.60933 |
| Linnarsson_MouseBrainAtlas_level5 | OBINH1 | 15140 | -0.018 | 0.0647 | 0.60972 |
| MouseCellAtlas_Adult_all | Mammary_Gland.Secretory_alveoli_cell_Gadd45b_high | 14459 | -0.0193 | 0.0691 | 0.60972 |
| TabulaMuris_droplet_Mammary | luminal_cell_of_lactiferous_duct | 13888 | -0.00977 | 0.035 | 0.60988 |
| DropViz_all_level2 | FC.Neuron_Slc17a7_Syt6-Slc30a3.3_5 | 14805 | -0.00211 | 0.00755 | 0.61004 |
| Linnarsson_GSE60361_Mouse_Cortex_Hippocampus_level2 | Int14 | 14010 | -0.00735 | 0.0263 | 0.6101 |
| MouseCellAtlas_all | Mammary_Gland.Secretory_alveoli_cell_Lgals1_high | 14459 | -0.0173 | 0.0619 | 0.61032 |
| MouseCellAtlas_Embryo_all | Placenta.NKT_cell | 14459 | -0.0182 | 0.0648 | 0.61048 |
| Allen_Mouse_ALM_level1 | L4_5 | 14744 | -0.00236 | 0.00839 | 0.61068 |
| Linnarsson_GSE67602_Mouse_Skin_Epidermis | IFE_DII | 14528 | -0.0108 | 0.0383 | 0.61086 |
| DropViz_HC_level2 | Oligodendrocyte_Trf_Ndrg2.8_2 | 14805 | -0.00145 | 0.00516 | 0.61098 |
| MouseCellAtlas_Adult_all | Prostate.Prostate_gland_cell | 14459 | -0.0336 | 0.119 | 0.6113 |
| MouseCellAtlas_all | Ovary.Cumulus_cell_Ube2c_high | 14459 | -0.0193 | 0.0682 | 0.61149 |
| MouseCellAtlas_Mammary_Gland | Mammary_Gland_Lactation.Secretory_alveoli_cell_AW112010_high | 14459 | -0.0253 | 0.0892 | 0.61159 |
| MouseCellAtlas_Neonatal_Rib | B_cell | 14459 | -0.0278 | 0.0982 | 0.61166 |
| MouseCellAtlas_Adult_all | Ovary.Small_luteal_cell | 14459 | -0.0256 | 0.09 | 0.61198 |
| Linnarsson_MouseBrainAtlas_level5 | HYPEP1 | 15140 | -0.0173 | 0.0607 | 0.61248 |
| MouseCellAtlas_Adult_all | Mammary_Gland.Secretory_alveoli_cell_Mrpl15_high | 14459 | -0.0195 | 0.0681 | 0.61249 |
| MouseCellAtlas_Mammary_Gland | Mammary_Gland_Involution.NK_cell_Gzma_high | 14459 | -0.0168 | 0.0586 | 0.61256 |
| Linnarsson_MouseBrainAtlas_level5 | SYNOR3 | 15140 | -0.00487 | 0.017 | 0.61271 |
| MouseCellAtlas_Adult_all | Kidney.Macrophage_Ccl4_high_ | 14459 | -0.0234 | 0.0814 | 0.61317 |
| DropViz_all_level2 | HC.Gad1Gad2_Cplx3_Tox.1_12 | 14805 | -0.00189 | 0.00653 | 0.61365 |
| Allen_Mouse_VISp_level2 | Smad3 | 13868 | -0.00316 | 0.0109 | 0.6137 |
| MouseCellAtlas_Embryo_all | Embryonic_Mesenchyme.Lymphoid_progenitor_cell | 14459 | -0.0256 | 0.0886 | 0.61372 |
| Linnarsson_MouseBrainAtlas_level5 | TEGLU7 | 15140 | -0.0101 | 0.0348 | 0.6138 |
| MouseCellAtlas_all | Mammary_Gland.Secretory_alveoli_cell_Wfdc17_high | 14459 | -0.0198 | 0.0686 | 0.6138 |
| GSE81547_Human_Pancreas | acinar | 16741 | -0.00263 | 0.00907 | 0.61395 |
| TabulaMuris_droplet_Marrow | Fraction_A_pre.pro_B_cell | 13888 | -0.0136 | 0.0465 | 0.61468 |
| MouseCellAtlas_Neonatal_all | Neonatal_Muscle.Erythroblast_Hbb.bs_high | 14459 | -0.02 | 0.0684 | 0.61482 |
| TabulaMuris_droplet_all | Lung.natural_killer_cell | 13888 | -0.015 | 0.0512 | 0.61484 |
| MouseCellAtlas_Adult_all | Mammary_Gland.Secretory_alveoli_cell_Iglc1_high | 14459 | -0.019 | 0.065 | 0.61506 |
| MouseCellAtlas_Neonatal_all | Neonatal_Muscle.Macrophage_Lyz2_high | 14459 | -0.0251 | 0.0856 | 0.61541 |
| Linnarsson_GSE101601_Mouse_Somatosensory_cortex | Pyramidal_cells_layer_5 | 14550 | -0.0135 | 0.0461 | 0.61559 |
| MouseCellAtlas_all | Neonatal_Calvaria.Muscle_cell_Myl9_high | 14459 | -0.0228 | 0.0772 | 0.61612 |
| DropViz_SN_level2 | Astrocyte_Gja1_Cst3.7_2 | 14805 | -0.0018 | 0.00608 | 0.61622 |
| DropViz_HC_level2 | Mural_Rgs5Acta2_Col3a1.16_4 | 14805 | -8.54E-04 | 0.00289 | 0.61628 |
| MouseCellAtlas_Adult_all | Mammary_Gland.Secretory_alveoli_cell_Clu_high | 14459 | -0.0174 | 0.0588 | 0.61666 |
| MouseCellAtlas_Neonatal_Calvaria | Granulocyte_monocyte_progenitor_cell | 14459 | -0.0186 | 0.0627 | 0.6167 |
| DropViz_all_level1 | ENT.Astrocyte | 14805 | -0.00166 | 0.00557 | 0.61685 |
| MouseCellAtlas_Adult_all | Mammary_Gland.Luminal_cell | 14459 | -0.0241 | 0.0811 | 0.61695 |
| DropViz_GP_level1 | Mural | 14805 | -0.00173 | 0.0058 | 0.6173 |
| MouseCellAtlas_Adult_all | Brain.Astroglial_cell | 14459 | -0.0136 | 0.0454 | 0.61748 |
| DropViz_PC_level2 | Neuron_Slc17a7-Slc17a8_Syt6-Lancl3.1_2 | 14805 | -0.00163 | 0.00543 | 0.61778 |
| Allen_Mouse_LGd_level2 | Dlx5_Cbln2 | 14545 | -0.00416 | 0.0139 | 0.61788 |
| DropViz_all_level1 | CB.Mural | 14805 | -0.00127 | 0.00423 | 0.61814 |
| DropViz_ENT_level2 | Neuron_Gad1Gad2_Pmch.4_10 | 14805 | -8.97E-04 | 0.00298 | 0.6184 |
| DropViz_GP_level2 | Oligodendrocyte_Trf_Klk6.10_2 | 14805 | -0.00174 | 0.00577 | 0.61869 |
| DropViz_TH_level1 | Astrocyte | 14805 | -0.00197 | 0.00651 | 0.6187 |
| DropViz_all_level2 | TH.Neuron_Gad1Gad2_Gata3.3_6 | 14805 | -0.00228 | 0.00751 | 0.61913 |
| Linnarsson_GSE67602_Mouse_Skin_Epidermis | uHF_II | 14528 | -0.0218 | 0.072 | 0.61913 |
| DropViz_all_level2 | PC.Neuron_Slc17a7_Syt6-Fos.1_9 | 14805 | -0.00233 | 0.00768 | 0.61918 |
| DropViz_all_level2 | GP.Neuron_Slc17a7_Neurod2-Rorb.2_11 | 14805 | -0.00182 | 0.00598 | 0.61924 |
| MouseCellAtlas_all | Prostate.Prostate_gland_cell | 14459 | -0.0359 | 0.118 | 0.61939 |
| MouseCellAtlas_Adult_all | Mammary_Gland.Secretory_alveoli_cell_Igkv1.110_high | 14459 | -0.0192 | 0.0631 | 0.61944 |
| MouseCellAtlas_Adult_all | Uterus.Glandular_epithelium_Sprr2f_high | 14459 | -0.0152 | 0.0499 | 0.61948 |
| Linnarsson_MouseBrainAtlas_level5 | MEGLU14 | 15140 | -0.00999 | 0.0328 | 0.61957 |
| MouseCellAtlas_Adult_all | Ovary.Cumulus_cell_Ube2c_high | 14459 | -0.0189 | 0.0621 | 0.61972 |
| MouseCellAtlas_Adult_all | Mammary_Gland.Secretory_alveoli_cell_Hsp90aa1_high | 14459 | -0.0195 | 0.0641 | 0.61973 |
| Linnarsson_GSE74672_Mouse_Hypothalamus_Neurons_level2 | Vglut2_15_Hcn16430411K18Rik | 14550 | -0.00705 | 0.0231 | 0.61974 |
| MouseCellAtlas_Mammary_Gland | Mammary_Gland_Lactation.Muscle__cell | 14459 | -0.0288 | 0.0943 | 0.61982 |
| GSE87544_Mouse_Hypothalamus | GABA10 | 13878 | -0.0107 | 0.0351 | 0.61999 |
| DropViz_all_level2 | FC.Neuron_Gad1Gad2_Cplx3.1_1 | 14805 | -0.00243 | 0.00794 | 0.62025 |
| DropViz_all_level2 | FC.Polydendrocyte_Tnr_Bmp4-Gpr17.10_6 | 14805 | -0.00175 | 0.00568 | 0.6211 |
| MouseCellAtlas_all | Mammary_Gland.Secretory_alveoli_cell_Mat2a_high | 14459 | -0.0202 | 0.0655 | 0.62112 |
| MouseCellAtlas_all | Brain.Astroglial_cell | 14459 | -0.014 | 0.0453 | 0.62114 |
| DropViz_GP_level2 | Neuron_Gad1Gad2_Drd1-Nefm.3_1 | 14805 | -0.00229 | 0.00742 | 0.62122 |
| MouseCellAtlas_Adult_all | Mammary_Gland.Adiocyte_cell | 14459 | -0.0295 | 0.0954 | 0.62134 |
| MouseCellAtlas_Adult_all | Mammary_Gland.Secretory_alveoli_cell_Dnajb1_high | 14459 | -0.0199 | 0.0645 | 0.62137 |
| GSE98816_Mouse_Brain_Vascular | PC | 14130 | -0.00332 | 0.0107 | 0.62154 |
| Linnarsson_GSE101601_Mouse_Somatosensory_cortex | Perivascular_macrophages | 14550 | -0.0221 | 0.0712 | 0.62169 |
| DropViz_all_level2 | FC.Neuron_Slc17a7_Parm1_Fos.7_4 | 14805 | -0.00128 | 0.00412 | 0.62179 |
| MouseCellAtlas_Bone_Marrow | Bone_Marrow.Neutrophil_Fcnb_high | 14459 | -0.013 | 0.0417 | 0.62208 |
| MouseCellAtlas_Mammary_Gland | Mammary_Gland_Virgin.Macrophage_Lyz1_high | 14459 | -0.018 | 0.0578 | 0.62209 |
| Allen_Mouse_ALM_level1 | Vip_Sncg | 14744 | -0.00358 | 0.0115 | 0.62222 |
| Linnarsson_GSE74672_Mouse_Hypothalamus_Neurons_level2 | Oxytocin_4 | 14550 | -0.00961 | 0.0308 | 0.62253 |
| DropViz_PC_level2 | Neuron_Sc17a7_Calb1-Lpl-Penk.2_7 | 14805 | -0.00306 | 0.00982 | 0.62255 |
| TabulaMuris_FACS_all | Diaphragm.skeletal_muscle_satellite_stem_cell | 13888 | -0.00287 | 0.00917 | 0.62289 |
| Linnarsson_GSE101601_Human_Temporal_cortex | Glut_1 | 17177 | -0.0579 | 0.185 | 0.62293 |
| MouseCellAtlas_Adult_all | Liver.Dendritic_cell_Cst3_high | 14459 | -0.0253 | 0.0809 | 0.62298 |
| DropViz_all_level2 | FC.Neuron_Gad1Gad2-Chat_Synpr-Slc5a7.1_5 | 14805 | -0.00249 | 0.00792 | 0.62329 |
| GSE67835_Human_Cortex_woFetal | oligodendrocytes | 16585 | -0.00271 | 0.00862 | 0.62352 |
| MouseCellAtlas_all | Mammary_Gland.Secretory_alveoli_cell_Cyb561_high | 14459 | -0.0176 | 0.0559 | 0.6236 |
| DropViz_all_level1 | FC.Macrophage | 14805 | -0.00151 | 0.00479 | 0.62415 |
| DroNc_Human_Hippocampus | exCA1 | 16914 | -0.0536 | 0.169 | 0.62419 |
| MouseCellAtlas_all | Mammary_Gland.Secretory_alveoli_cell_Cidea_high | 14459 | -0.0211 | 0.0665 | 0.62425 |
| Linnarsson_MouseBrainAtlas_level6_rank4 | Ghyp | 15140 | -0.0142 | 0.0447 | 0.62434 |
| DropViz_CB_level1 | Oligodendrocyte | 14805 | -0.00172 | 0.00543 | 0.62449 |
| Linnarsson_GSE74672_Mouse_Hypothalamus_Neurons_level2 | Vglut2_8 | 14550 | -0.0133 | 0.0419 | 0.62449 |
| DropViz_all_level2 | STR.Mural_Rgs5Acta2_Ccnd1.8_3 | 14805 | -0.00125 | 0.00394 | 0.62502 |
| Linnarsson_GSE59739_Mouse_Dorsal_root_ganglion_level2 | Peptidergic_nociceptor1 | 13848 | -0.00471 | 0.0148 | 0.6251 |
| MouseCellAtlas_Neonatal_all | Neonatal_Heart.Atrial_cardiomyocyte_Acta2_high | 14459 | -0.0305 | 0.0955 | 0.6252 |
| MouseCellAtlas_Adult_all | Bone_Marrow.Neutrophil_Ngp_high | 14459 | -0.0172 | 0.0537 | 0.62551 |
| Linnarsson_GSE74672_Mouse_Hypothalamus_Neurons_level2 | GABA_14_NpyAgrp | 14550 | -0.0124 | 0.0387 | 0.62561 |
| MouseCellAtlas_all | Uterus.Glandular_epithelium_Sprr2f_high | 14459 | -0.0163 | 0.0508 | 0.62563 |
| MouseCellAtlas_Peripheral_Blood | Neutrophil_Camp_high | 14459 | -0.0184 | 0.0572 | 0.62624 |
| MouseCellAtlas_Adult_all | Mammary_Gland.Secretory_alveoli_cell_Fth1_high | 14459 | -0.021 | 0.0652 | 0.62642 |
| MouseCellAtlas_Adult_all | Mammary_Gland.Secretory_alveoli_cell_Cgrrf1_high | 14459 | -0.0218 | 0.0676 | 0.62644 |
| GSE98816_Mouse_Brain_Vascular | vSMC | 14130 | -0.00302 | 0.00935 | 0.62659 |
| MouseCellAtlas_Embryo_all | Fetal_Intestine.Neuronal_cell_Fabp7_high | 14459 | -0.0466 | 0.144 | 0.6267 |
| TabulaMuris_droplet_all | Trachea.epithelial_cell | 13888 | -0.0147 | 0.0453 | 0.6269 |
| MouseCellAtlas_Mammary_Gland | Mammary_Gland_Pregnancy.Myoepithelial_cell | 14459 | -0.0125 | 0.0384 | 0.627 |
| MouseCellAtlas_all | Neonatal_Heart.Neutrophil_Ngp_high | 14459 | -0.0194 | 0.0598 | 0.6271 |
| GSE93374_Mouse_Arc_ME_level2 | Neurons3 | 13325 | -0.02 | 0.0616 | 0.62729 |
| Linnarsson_MouseBrainAtlas_level5 | HYPEN | 15140 | -0.0132 | 0.0406 | 0.62772 |
| GSE92332_Mouse_Epithelium_droplet | Enterocyte_Immature_Distal | 11993 | -0.0226 | 0.0692 | 0.62785 |
| MouseCellAtlas_all | Fetal_Intestine.Neuronal_cell_Fabp7_high | 14459 | -0.0307 | 0.0941 | 0.62805 |
| Linnarsson_GSE76381_Mouse_Midbrain | Rgl3 | 14550 | -0.0115 | 0.0352 | 0.62812 |
| MouseCellAtlas_Placenta | Invasive_spongiotrophoblast | 14459 | -0.0245 | 0.0749 | 0.62833 |
| TabulaMuris_FACS_all | Pancreas.pancreatic_PP_cell | 13888 | -0.00217 | 0.00662 | 0.62847 |
| GSE93374_Mouse_Arc_ME_neurons | Oxt | 13325 | -0.027 | 0.0823 | 0.62849 |
| Linnarsson_GSE74672_Mouse_Hypothalamus_Neurons_level2 | GABA_9 | 14550 | -0.0181 | 0.0553 | 0.62857 |
| MouseCellAtlas_all | Mammary_Gland.Luminal_cell | 14459 | -0.0263 | 0.08 | 0.629 |
| DropViz_all_level2 | FC.Neuron_Slc17a7_Syt6-Slc24a2.3_6 | 14805 | -0.00196 | 0.00595 | 0.62907 |
| MouseCellAtlas_Embryo_all | Fetal_Liver.B_cell | 14459 | -0.0216 | 0.0652 | 0.62976 |
| MouseCellAtlas_all | Kidney.Macrophage_Ccl4_high_ | 14459 | -0.026 | 0.0784 | 0.63007 |
| MouseCellAtlas_all | Bone_Marrow.Myoblast | 14459 | -0.0228 | 0.0685 | 0.63022 |
| GSE93374_Mouse_Arc_ME_level2 | a1_tanycytes2 | 13325 | -0.0365 | 0.11 | 0.63027 |
| MouseCellAtlas_Adult_all | Bone_Marrow.Dendritic_cell_Siglech_high | 14459 | -0.0159 | 0.0479 | 0.63032 |
| Allen_Mouse_VISp_level2 | Vip_Sncg | 13868 | -0.00384 | 0.0115 | 0.63047 |
| MouseCellAtlas_Small_Intestine | T_cell_Icos_high | 14459 | -0.0302 | 0.0905 | 0.63069 |
| DropViz_PC_level2 | Neuron_Slc17a7_Syt6-Nefm.1_8 | 14805 | -0.00368 | 0.011 | 0.63087 |
| MouseCellAtlas_all | Neonatal_Skin.Brown_adipose_tissue_Cox8b_high | 14459 | -0.0177 | 0.0528 | 0.6311 |
| DropViz_all_level2 | STR.Neuron_Gad1Gad2_Pnoc.14_2 | 14805 | -0.00139 | 0.00414 | 0.63113 |
| MouseCellAtlas_all | Mammary_Gland.Secretory_alveoli_cell_Sars_high | 14459 | -0.0211 | 0.0627 | 0.63162 |
| Linnarsson_GSE76381_Human_Midbrain | Rgl3 | 14835 | -0.0137 | 0.0406 | 0.63164 |
| TabulaMuris_droplet_all | Heart.smooth_muscle_cell | 13888 | -0.00989 | 0.0294 | 0.63167 |
| MouseCellAtlas_Mammary_Gland | Mammary_Gland_Pregnancy.Secretory_alveoli_cell | 14459 | -0.0124 | 0.0369 | 0.63171 |
| TabulaMuris_FACS_Bladder | basal_cell_of_urothelium | 13888 | -0.00398 | 0.0118 | 0.63177 |
| DropViz_SN_level2 | Mural_Rgs5Acta2_Cd9.13_3 | 14805 | -0.00159 | 0.00472 | 0.6318 |
| MouseCellAtlas_Mammary_Gland | Mammary_Gland_Involution.Macrophage_Retnla_high | 14459 | -0.0205 | 0.0608 | 0.6319 |
| Linnarsson_GSE59739_Mouse_Dorsal_root_ganglion_level3 | Neurofilament_containing4 | 13848 | -0.00366 | 0.0109 | 0.63201 |
| GSE106678_Mouse_Cortex | Ex19 | 14573 | -0.0579 | 0.171 | 0.6327 |
| TabulaMuris_droplet_Heart | erythrocyte | 13888 | -0.0108 | 0.0319 | 0.63273 |
| GSE104276_Human_Prefrontal_cortex_per_ages | GW23_Microglia | 17199 | -0.00208 | 0.00614 | 0.63281 |
| MouseCellAtlas_Adult_all | Mammary_Gland.Secretory_alveoli_cell_Cfd_high | 14459 | -0.0225 | 0.066 | 0.63318 |
| MouseCellAtlas_all | Mammary_Gland.Secretory_alveoli_cell_Ccng1_high | 14459 | -0.0207 | 0.0608 | 0.63337 |
| Linnarsson_GSE101601_Mouse_Somatosensory_cortex | Vascular_smooth_muscle | 14550 | -0.0234 | 0.0686 | 0.6335 |
| MouseCellAtlas_all | Bone_Marrow.Eosinophils | 14459 | -0.0234 | 0.0683 | 0.63385 |
| MouseCellAtlas_Neonatal_all | Neonatal_Heart.Atrial_cardiomyocyte | 14459 | -0.0159 | 0.0462 | 0.63427 |
| Linnarsson_MouseBrainAtlas_level5 | SCINH9 | 15140 | -0.0182 | 0.0529 | 0.63463 |
| Allen_Mouse_ALM_level2 | L2.3_ALM_Cbln4 | 14744 | -0.00273 | 0.00793 | 0.63468 |
| DropViz_all_level1 | TH.Ependymal | 14805 | -0.00165 | 0.0048 | 0.63471 |
| MouseCellAtlas_Adult_all | Bone_Marrow.Eosinophils | 14459 | -0.0227 | 0.0659 | 0.63479 |
| DropViz_PC_level2 | Neuron_Sc17a7_Tshz2-Smoc1.2_3 | 14805 | -0.00319 | 0.00925 | 0.63502 |
| DropViz_HC_level2 | Neuron_Slc17a7_Fibcd1-Fos.5_8 | 14805 | -0.00299 | 0.00864 | 0.63512 |
| DropViz_STR_level2 | Neuron_Gad1Gad2_Drd1-Fos.10_1 | 14805 | -0.00272 | 0.00784 | 0.63571 |
| DropViz_all_level2 | TH.Neuron_Gad1Gad2_Pou6f2-Pax6.3_3 | 14805 | -0.00281 | 0.00809 | 0.63585 |
| MouseCellAtlas_Bladder | Vascular_endothelial_cell | 14459 | -0.0266 | 0.0764 | 0.6359 |
| DropViz_HC_level2 | Gad1Gad2_Htr3a_Sema3c.1_27 | 14805 | -0.0022 | 0.00633 | 0.63593 |
| DropViz_STR_level1 | Polydendrocyte | 14805 | -0.00239 | 0.00687 | 0.63628 |
| MouseCellAtlas_Fetal_Intestine | Acinar_cell | 14459 | -0.0295 | 0.0845 | 0.63636 |
| MouseCellAtlas_Adult_all | Peripheral_Blood.Neutrophil_Ltf_high | 14459 | -0.0167 | 0.048 | 0.6364 |
| GSE104276_Human_Prefrontal_cortex_per_ages | GW12_Microglia | 17199 | -0.0011 | 0.00316 | 0.63641 |
| Linnarsson_MouseBrainAtlas_level5 | TECHO | 15140 | -0.0131 | 0.0375 | 0.63706 |
| MouseCellAtlas_Neonatal_all | Neonatal_Muscle.Neutrophil_Stfa3_high | 14459 | -0.028 | 0.0799 | 0.63715 |
| Linnarsson_MouseBrainAtlas_level5 | TEINH15 | 15140 | -0.0183 | 0.0521 | 0.63721 |
| MouseCellAtlas_Adult_all | Spleen.Macrophage | 14459 | -0.0203 | 0.0579 | 0.63729 |
| GSE93374_Mouse_Arc_ME_neurons | Nfix_Htr2c | 13325 | -0.0466 | 0.133 | 0.63737 |
| MouseCellAtlas_Adult_all | Bone_Marrow.Myoblast | 14459 | -0.0225 | 0.0641 | 0.63742 |
| GSE93374_Mouse_Arc_ME_level2 | Parstuber2A | 13325 | -0.0295 | 0.0838 | 0.63747 |
| MouseCellAtlas_all | Mammary_Gland.Secretory_alveoli_cell_Gadd45b_high | 14459 | -0.0235 | 0.0668 | 0.6376 |
| MouseCellAtlas_Adult_all | Mammary_Gland.Secretory_alveoli_cell_Srsf11_high | 14459 | -0.0233 | 0.066 | 0.63785 |
| MouseCellAtlas_Adult_all | Mammary_Gland.Secretory_alveoli_cell_Actb_high | 14459 | -0.0233 | 0.0659 | 0.63801 |
| TabulaMuris_droplet_all | Marrow.monocyte | 13888 | -0.0122 | 0.0345 | 0.63835 |
| MouseCellAtlas_all | Mammary_Gland.Secretory_alveoli_cell_Mrpl15_high | 14459 | -0.0235 | 0.0662 | 0.63851 |
| MouseCellAtlas_all | Fetal_Liver.B_cell | 14459 | -0.0197 | 0.0555 | 0.6387 |
| MouseCellAtlas_Fetal_Liver | Macrophage | 14459 | -0.0255 | 0.0719 | 0.63875 |
| MouseCellAtlas_all | Bone_Marrow.Neutrophil_Ngp_high | 14459 | -0.0188 | 0.053 | 0.63881 |
| DropViz_HC_level2 | Neuron_Slc17a7_Cbln1-Trps1.5_12 | 14805 | -0.00262 | 0.00737 | 0.63895 |
| TabulaMuris_FACS_all | Lung.T_cell | 13888 | -0.00332 | 0.00933 | 0.63917 |
| Allen_Mouse_ALM_level2 | Vip_Chat_3 | 14744 | -0.00397 | 0.0111 | 0.63961 |
| Allen_Mouse_ALM_level2 | L6a_Col12a1_1 | 14744 | -0.00233 | 0.00651 | 0.63993 |
| Allen_Mouse_ALM_level2 | L5_ALM_Aldh3b1_1 | 14744 | -0.00412 | 0.0115 | 0.64007 |
| MouseCellAtlas_all | Mammary_Gland.Secretory_alveoli_cell_Clu_high | 14459 | -0.0206 | 0.0574 | 0.64026 |
| DropViz_all_level2 | GP.Polydendrocyte_Tnr_Cyth3.4_7 | 14805 | -9.18E-04 | 0.00255 | 0.64035 |
| GSE89164_Mouse_Hindbrain | Microglia | 12262 | -0.0301 | 0.0838 | 0.64053 |
| MouseCellAtlas_Bone_Marrow | Bone_Marrow.Monocyte_Prtn3_high | 14459 | -0.0102 | 0.0282 | 0.64097 |
| MouseCellAtlas_all | Liver.Dendritic_cell_Cst3_high | 14459 | -0.028 | 0.0776 | 0.64097 |
| MouseCellAtlas_Prostate | Stromal_cell | 14459 | -0.0831 | 0.23 | 0.641 |
| MouseCellAtlas_all | Mammary_Gland.Secretory_alveoli_cell_Iglc1_high | 14459 | -0.0228 | 0.0632 | 0.6411 |
| MouseCellAtlas_Mammary_Gland | Mammary_Gland_Lactation.Secretory_alveoli_cell_Malat1_high | 14459 | -0.0341 | 0.0941 | 0.64128 |
| TabulaMuris_droplet_all | Kidney.macrophage | 13888 | -0.015 | 0.0414 | 0.64133 |
| MouseCellAtlas_all | Bone_Marrow.Dendritic_cell_Siglech_high | 14459 | -0.0171 | 0.0472 | 0.64185 |
| DropViz_PC_level2 | Oligodendrocyte_Tfr_Sez6.9_5 | 14805 | -0.00181 | 0.00499 | 0.64198 |
| MouseCellAtlas_Neonatal_Skin | Keratinocyte | 14459 | -0.0307 | 0.0844 | 0.64214 |
| MouseCellAtlas_Bone_Marrow | Bone_Marrow_c_kit.Neutrophil_Ighg1_high | 14459 | -0.0408 | 0.112 | 0.64223 |
| Linnarsson_MouseBrainAtlas_level5 | SCINH5 | 15140 | -0.0135 | 0.037 | 0.64245 |
| MouseCellAtlas_Adult_all | Muscle.Dendritic_cell | 14459 | -0.0218 | 0.0596 | 0.64251 |
| MouseCellAtlas_Adult_all | Mammary_Gland.Secretory_alveoli_cell_Igkv1.117_high | 14459 | -0.0241 | 0.066 | 0.64254 |
| MouseCellAtlas_Adult_all | Ovary.Macrophage_Lyz2_high | 14459 | -0.0235 | 0.0642 | 0.64308 |
| MouseCellAtlas_Adult_all | Lung.Dendritic_cell_Naaa_high | 14459 | -0.0275 | 0.0748 | 0.64324 |
| Linnarsson_GSE60361_Mouse_Cortex_Hippocampus_level2 | Oligo5 | 14010 | -0.00869 | 0.0237 | 0.64338 |
| Allen_Mouse_VISp_level2 | L6a_Sla | 13868 | -0.0057 | 0.0155 | 0.64343 |
| MouseCellAtlas_Adult_all | Bone_Marrow.Hematopoietic_stem_progenitor_cell | 14459 | -0.0175 | 0.0476 | 0.64346 |
| MouseCellAtlas_all | Mammary_Gland.Secretory_alveoli_cell_Igkv1.110_high | 14459 | -0.0227 | 0.0617 | 0.64347 |
| DropViz_PC_level2 | Neuron_Slc17a7_Bcl6-Tshz2-Syt2.3_8 | 14805 | -0.00317 | 0.00861 | 0.64355 |
| MouseCellAtlas_Adult_all | Lung.Dividing_dendritic_cells | 14459 | -0.0222 | 0.06 | 0.64428 |
| DropViz_all_level2 | GP.Neuron_Gad1Gad2_Vipr2.3_10 | 14805 | -0.0016 | 0.00433 | 0.64439 |
| TabulaMuris_droplet_Thymus | T_cell | 13888 | -0.0363 | 0.0981 | 0.64443 |
| MouseCellAtlas_Neonatal_all | Neonatal_Skin.Macrophage_Cd74_high | 14459 | -0.0177 | 0.0476 | 0.645 |
| Allen_Mouse_VISp_level2 | L5b_Chrna6 | 13868 | -0.00401 | 0.0108 | 0.6453 |
| MouseCellAtlas_Mammary_Gland | Mammary_Gland_Lactation.Secretory_alveoli_cell_Lyz2_high | 14459 | -0.0342 | 0.0917 | 0.64534 |
| Linnarsson_MouseBrainAtlas_level5 | OBNBL1 | 15140 | -0.0167 | 0.0447 | 0.64538 |
| GSE99235_Mouse_Lung_Vascular | EC2 | 14559 | -0.00426 | 0.0114 | 0.64613 |
| DropViz_PC_level1 | Mural | 14805 | -0.00224 | 0.00596 | 0.64622 |
| MouseCellAtlas_Pancreas | Macrophage_Ly6c2_high | 14459 | -0.0261 | 0.0696 | 0.64649 |
| MouseCellAtlas_all | Bone_Marrow.Hematopoietic_stem_progenitor_cell | 14459 | -0.0183 | 0.0486 | 0.64649 |
| DropViz_all_level1 | TH.Polydendrocyte | 14805 | -0.00268 | 0.00712 | 0.64654 |
| MouseCellAtlas_Adult_all | Mammary_Gland.Secretory_alveoli_cell_Taf1d_high | 14459 | -0.0227 | 0.0604 | 0.64658 |
| MouseCellAtlas_all | Mammary_Gland.Secretory_alveoli_cell_Hsp90aa1_high | 14459 | -0.0233 | 0.0621 | 0.64659 |
| DropViz_all_level2 | HC.Neuron_Slc17a6_Lhx1.14_1 | 14805 | -0.00256 | 0.00679 | 0.64669 |
| MouseCellAtlas_Placenta | Spongiotrophoblast_Hsd11b2_high | 14459 | -0.022 | 0.0584 | 0.64692 |
| MouseCellAtlas_Fetal_Lung | Erythroblast_Mt2.Mt1_high | 14459 | -0.0408 | 0.108 | 0.64708 |
| Linnarsson_GSE76381_Human_Midbrain | NProg | 14835 | -0.0283 | 0.0749 | 0.64734 |
| DropViz_all_level2 | SN.Neuron_Slc17a6_Cacna2d1.3_10 | 14805 | -0.00316 | 0.00832 | 0.6477 |
| MouseCellAtlas_all | Mammary_Gland.Secretory_alveoli_cell_Dnajb1_high | 14459 | -0.0237 | 0.0624 | 0.64783 |
| GSE92332_Mouse_Epithelium_droplet | Enterocyte_Immature_Proximal | 11993 | -0.0211 | 0.0554 | 0.64822 |
| Linnarsson_GSE104323_Mouse_Dentate_gyrus | Immature_Astro | 14909 | -0.0191 | 0.0501 | 0.64828 |
| Allen_Mouse_ALM_level1 | Meis2 | 14744 | -0.00203 | 0.00532 | 0.64838 |
| TabulaMuris_droplet_all | Spleen.T_cell | 13888 | -0.0182 | 0.0478 | 0.6484 |
| MouseCellAtlas_Adult_all | Mammary_Gland.Secretory_alveoli_cell_Tubb5_high | 14459 | -0.0237 | 0.0622 | 0.64841 |
| GSE93374_Mouse_Arc_ME_neurons | Agrp_Sst | 13325 | -0.13 | 0.34 | 0.64854 |
| MouseCellAtlas_all | Mammary_Gland.Adiocyte_cell | 14459 | -0.035 | 0.0918 | 0.64874 |
| Linnarsson_MouseBrainAtlas_level5 | SCGLU7 | 15140 | -0.0193 | 0.0503 | 0.64928 |
| MouseCellAtlas_all | Embryonic_Mesenchyme.Reproductive_tissues | 14459 | -0.0351 | 0.0913 | 0.64961 |
| Allen_Mouse_ALM_level2 | L5_ALM_Pld5_2 | 14744 | -0.004 | 0.0104 | 0.64966 |
| DropViz_all_level2 | PC.Neuron_Gad1Gad2-Chat_Synpr-Slc5a7.4_8 | 14805 | -0.00291 | 0.00754 | 0.65005 |
| MouseCellAtlas_Mammary_Gland | Mammary_Gland_Lactation.Secretory_alveoli_cell_Yif1b1_high | 14459 | -0.0382 | 0.0986 | 0.65058 |
| MouseCellAtlas_Neonatal_Skin | Mast_cell | 14459 | -0.0261 | 0.0673 | 0.65068 |
| MouseCellAtlas_all | Mammary_Gland.Secretory_alveoli_cell_Fth1_high | 14459 | -0.0246 | 0.0636 | 0.65076 |
| DropViz_FC_level2 | Neuron_Gad1Gad2_Cplx3-Reln.1_2 | 14805 | -0.00351 | 0.00905 | 0.65084 |
| TabulaMuris_FACS_all | Marrow.T_cell | 13888 | -0.00324 | 0.00836 | 0.65084 |
| Linnarsson_MouseBrainAtlas_level5 | MEINH14 | 15140 | -0.0179 | 0.0461 | 0.651 |
| MouseCellAtlas_Adult_all | Bone_Marrow.Macrophage_Ms4a6c_high | 14459 | -0.0162 | 0.0418 | 0.65139 |
| MouseCellAtlas_all | Peripheral_Blood.Neutrophil_Ltf_high | 14459 | -0.0184 | 0.0473 | 0.6515 |
| MouseCellAtlas_Brain | Granulocyte_Il33_high | 14459 | -0.0184 | 0.047 | 0.65207 |
| DropViz_all_level2 | PC.Neuron_Slc17a7-Slc17a8_Syt6-Lancl3.1_2 | 14805 | -0.00197 | 0.00504 | 0.65211 |
| MouseCellAtlas_all | Muscle.Dendritic_cell | 14459 | -0.023 | 0.0589 | 0.65215 |
| MouseCellAtlas_Adult_all | Mammary_Gland.Secretory_alveoli_cell_Kif5b_high | 14459 | -0.0265 | 0.0676 | 0.65227 |
| DropViz_all_level1 | ENT.Oligodendrocyte | 14805 | -0.00266 | 0.00678 | 0.65251 |
| MouseCellAtlas_all | Lung.Dividing_dendritic_cells | 14459 | -0.0235 | 0.0597 | 0.65285 |
| MouseCellAtlas_Adult_all | Bone_Marrow.Monocyte_progenitor_cell_Prtn3_high | 14459 | -0.0269 | 0.0683 | 0.65292 |
| MouseCellAtlas_all | Mammary_Gland.Secretory_alveoli_cell_Cgrrf1_high | 14459 | -0.0257 | 0.0652 | 0.65294 |
| MouseCellAtlas_Adult_all | Mammary_Gland.Endothelial_cell_Fabp4.Aqp1_high | 14459 | -0.0219 | 0.0555 | 0.6531 |
| Linnarsson_MouseBrainAtlas_level5 | TEGLU10 | 15140 | -0.0122 | 0.031 | 0.65317 |
| MouseCellAtlas_Fetal_Brain | Radial_glia_Fabp7_high | 14459 | -0.0627 | 0.159 | 0.65329 |
| Linnarsson_GSE104323_Mouse_Dentate_gyrus | MiCajal_Retziusoglia | 14909 | -0.0178 | 0.0451 | 0.65342 |
| MouseCellAtlas_Neonatal_Rib | Osteoclast | 14459 | -0.021 | 0.0532 | 0.65359 |
| DropViz_HC_level2 | Neuron_Gad1Gad2_Sst_Grm1.1_9 | 14805 | -0.00289 | 0.0073 | 0.6538 |
| MouseCellAtlas_Adult_all | Bone_Marrow.Monocyte_progenitor_cell_Ctsg_high | 14459 | -0.0202 | 0.0509 | 0.65438 |
| MouseCellAtlas_Mammary_Gland | Mammary_Gland_Virgin.T_cell_Ly6c2_high | 14459 | -0.0233 | 0.0587 | 0.65442 |
| MouseCellAtlas_all | Lung.Dendritic_cell_Naaa_high | 14459 | -0.0293 | 0.0735 | 0.65466 |
| DropViz_all_level1 | PC.Mural | 14805 | -0.00234 | 0.00587 | 0.65467 |
| MouseCellAtlas_Mammary_Gland | Mammary_Gland_Lactation.Secretory_alveoli_cell_Apod_high | 14459 | -0.0379 | 0.0952 | 0.6547 |
| MouseCellAtlas_Lung | Ciliated_cell | 14459 | -0.0256 | 0.0641 | 0.65497 |
| GSE87544_Mouse_Hypothalamus | Hista | 13878 | -0.0162 | 0.0405 | 0.65514 |
| MouseCellAtlas_Fetal_Stomache | Mast_cell | 14459 | -0.058 | 0.145 | 0.65532 |
| GSE93374_Mouse_Arc_ME_level1 | Tanycyte2 | 13325 | -0.0462 | 0.115 | 0.65556 |
| Linnarsson_MouseBrainAtlas_level5 | SYNOR1 | 15140 | -0.00722 | 0.018 | 0.65558 |
| MouseCellAtlas_all | Fetal_Brain.Macrophage | 14459 | -0.0276 | 0.0689 | 0.65558 |
| MouseCellAtlas_all | Spleen.Macrophage | 14459 | -0.0224 | 0.0558 | 0.65573 |
| DropViz_all_level2 | ENT.Neuron_Gad1Gad2_Six3-Pvalb.4_2 | 14805 | -0.00248 | 0.00619 | 0.65579 |
| MouseCellAtlas_Adult_all | Prostate.Epithelial_cell | 14459 | -0.0491 | 0.122 | 0.65637 |
| MouseCellAtlas_Ovary | Macrophage_Lyz2_high | 14459 | -0.0229 | 0.0569 | 0.65654 |
| Linnarsson_MouseBrainAtlas_level5 | MGL2 | 15140 | -0.0146 | 0.0361 | 0.65678 |
| MouseCellAtlas_all | Bone_Marrow.Monocyte_progenitor_cell_Prtn3_high | 14459 | -0.0283 | 0.0698 | 0.65751 |
| MouseCellAtlas_Adult_all | Stomach.Epithelial_cell_Gkn3_high | 14459 | -0.0197 | 0.0486 | 0.65757 |
| MouseCellAtlas_Adult_all | Bone_Marrow.Macrophage_S100a4_high | 14459 | -0.0261 | 0.0644 | 0.6576 |
| Allen_Mouse_VISp_level2 | L5a_Pde1c | 13868 | -0.00563 | 0.0139 | 0.65764 |
| DropViz_FC_level2 | Polydendrocyte_Tnr_Dlx1.10_2 | 14805 | -0.0018 | 0.00442 | 0.65776 |
| MouseCellAtlas_Adult_all | Mammary_Gland.T_cell_Icos_high | 14459 | -0.0319 | 0.0786 | 0.65788 |
| Linnarsson_GSE59739_Mouse_Dorsal_root_ganglion_level1 | Peptidergic_nociceptor | 13848 | -0.00797 | 0.0196 | 0.6579 |
| MouseCellAtlas_all | Mammary_Gland.Endothelial_cell_Fabp4.Aqp1_high | 14459 | -0.0227 | 0.0557 | 0.65809 |
| TabulaMuris_droplet_Marrow | hematopoietic_stem_cell | 13888 | -0.0181 | 0.0444 | 0.65817 |
| MouseCellAtlas_Placenta | NKT_cell | 14459 | -0.0328 | 0.0805 | 0.65821 |
| MouseCellAtlas_Bone_Marrow | Bone_Marrow_c_kit.Neutrophil_Ltf_high | 14459 | -0.0229 | 0.0561 | 0.65857 |
| MouseCellAtlas_Adult_all | Testis.Erythroblast_Hbb.bs_high | 14459 | -0.0251 | 0.0613 | 0.65862 |
| MouseCellAtlas_Mammary_Gland | Mammary_Gland_Lactation.Secretory_alveoli_cell_Retnla_high | 14459 | -0.0369 | 0.09 | 0.65889 |
| GSE104276_Human_Prefrontal_cortex_per_ages | GW12_GABAergic_neurons | 17199 | -0.00313 | 0.00765 | 0.65898 |
| DropViz_all_level1 | HC.Ependymal | 14805 | -0.002 | 0.00488 | 0.65901 |
| Linnarsson_MouseBrainAtlas_level5 | ACBG | 15140 | -0.0185 | 0.0452 | 0.65911 |
| MouseCellAtlas_all | Bone_Marrow.Monocyte_progenitor_cell_Ctsg_high | 14459 | -0.0215 | 0.0524 | 0.65914 |
| MouseCellAtlas_all | Ovary.Macrophage_Lyz2_high | 14459 | -0.0252 | 0.0614 | 0.65921 |
| GSE67835_Human_Cortex | hybrid | 16585 | -0.00481 | 0.0117 | 0.65924 |
| MouseCellAtlas_all | Mammary_Gland.Secretory_alveoli_cell_Cfd_high | 14459 | -0.0263 | 0.0639 | 0.65942 |
| DropViz_all_level2 | PC.Neuron_Sc17a7_Calb1-Lpl-Penk.2_7 | 14805 | -0.00305 | 0.00741 | 0.65956 |
| DropViz_GP_level2 | Neuron_Gad1Gad2_Sphkap-Tmem255a.3_7 | 14805 | -0.00321 | 0.00779 | 0.65969 |
| MouseCellAtlas_Bone_Marrow | Bone_Marrow_c_kit.Macrophage_Cd74_high | 14459 | -0.0325 | 0.0787 | 0.66005 |
| MouseCellAtlas_Bone_Marrow | Bone_Marrow_Mesenchyme.Proerythrocytes | 14459 | -0.0254 | 0.0615 | 0.66005 |
| MouseCellAtlas_Adult_all | Mammary_Gland.Secretory_alveoli_cell_AW112010_high | 14459 | -0.0271 | 0.0656 | 0.6602 |
| Linnarsson_MouseBrainAtlas_level5 | TEGLU22 | 15140 | -0.0184 | 0.0446 | 0.66025 |
| MouseCellAtlas_all | Testis.Erythroblast_Hbb.bs_high | 14459 | -0.0254 | 0.0613 | 0.66095 |
| DroNc_Human_Hippocampus | GABA1 | 16914 | -0.0829 | 0.2 | 0.66119 |
| MouseCellAtlas_all | Bone_Marrow.Macrophage_Ms4a6c_high | 14459 | -0.0172 | 0.0412 | 0.66175 |
| DropViz_STR_level2 | Neurogenesis_Sox4_Notum.2_6 | 14805 | -0.00164 | 0.00391 | 0.66222 |
| MouseCellAtlas_Adult_all | Mammary_Gland.Muscle__cell | 14459 | -0.0275 | 0.0657 | 0.66231 |
| MouseCellAtlas_Adult_all | Mammary_Gland.NK_cell | 14459 | -0.0359 | 0.0857 | 0.6624 |
| MouseCellAtlas_all | Mammary_Gland.Secretory_alveoli_cell_Srsf11_high | 14459 | -0.0268 | 0.0639 | 0.66246 |
| MouseCellAtlas_Adult_all | Thymus.B_cell | 14459 | -0.0384 | 0.0916 | 0.66251 |
| MouseCellAtlas_all | Stomach.Epithelial_cell_Gkn3_high | 14459 | -0.0206 | 0.0492 | 0.66254 |
| DropViz_HC_level2 | Gad1Gad2_Htr3a_Krt73.1_23 | 14805 | -0.00362 | 0.00862 | 0.66259 |
| MouseCellAtlas_Mammary_Gland | Mammary_Gland_Virgin.Dendritic_cell_Fscn1_high | 14459 | -0.0215 | 0.0511 | 0.66305 |
| MouseCellAtlas_Small_Intestine | B_cell_Ms4a1_high | 14459 | -0.0303 | 0.0719 | 0.66315 |
| MouseCellAtlas_all | Fetal_Lung.Erythroblast_Hbb.bs_high | 14459 | -0.0399 | 0.0947 | 0.6632 |
| MouseCellAtlas_all | Mammary_Gland.T_cell_Icos_high | 14459 | -0.0335 | 0.0794 | 0.66327 |
| MouseCellAtlas_Embryo_all | Embryonic_Mesenchyme.Reproductive_tissues | 14459 | -0.0434 | 0.103 | 0.66332 |
| Linnarsson_GSE76381_Mouse_Midbrain | DA2 | 14550 | -0.0243 | 0.0575 | 0.66362 |
| MouseCellAtlas_Adult_all | Peripheral_Blood.Basophil_Prss34_high | 14459 | -0.0357 | 0.0844 | 0.66408 |
| MouseCellAtlas_all | Mammary_Gland.Secretory_alveoli_cell_Actb_high | 14459 | -0.0271 | 0.064 | 0.66419 |
| MouseCellAtlas_all | Fetal_Stomache.Mast_cell | 14459 | -0.0447 | 0.105 | 0.66427 |
| MouseCellAtlas_all | Thymus.B_cell | 14459 | -0.0402 | 0.0947 | 0.6645 |
| MouseCellAtlas_all | Prostate.Epithelial_cell | 14459 | -0.0517 | 0.121 | 0.6652 |
| MouseCellAtlas_Pancreas | B_cell | 14459 | -0.0249 | 0.0582 | 0.66523 |
| DropViz_all_level2 | HC.Mural_Rgs5Acta2_Col3a1.16_4 | 14805 | -0.00127 | 0.00297 | 0.66541 |
| MouseCellAtlas_all | Mammary_Gland.Secretory_alveoli_cell_Igkv1.117_high | 14459 | -0.0275 | 0.0643 | 0.66565 |
| Allen_Mouse_ALM_level2 | Sst_Mrap2_3 | 14744 | -0.00573 | 0.0134 | 0.66587 |
| MouseCellAtlas_Adult_all | Mammary_Gland.Muscle_cell | 14459 | -0.0354 | 0.0824 | 0.66638 |
| DropViz_all_level2 | STR.Ependyma_Ccdc153_Ccdc153.1_1 | 14805 | -0.00191 | 0.00444 | 0.66692 |
| MouseCellAtlas_Embryonic_Stem_Cell | ES_2C_like | 14459 | -0.0305 | 0.0708 | 0.66698 |
| MouseCellAtlas_Mammary_Gland | Mammary_Gland_Lactation.Secretory_alveoli_cell_Cide.Wfdc3.Wap_high | 14459 | -0.0344 | 0.0797 | 0.66727 |
| Linnarsson_GSE60361_Mouse_Cortex_Hippocampus_level1 | oligodendrocytes | 14010 | -0.0111 | 0.0256 | 0.66755 |
| DropViz_all_level2 | PC.Neuron_Slc17a7_Syt6-Nefm.1_8 | 14805 | -0.00348 | 0.00802 | 0.66771 |
| Linnarsson_GSE60361_Mouse_Cortex_Hippocampus_level2 | Int13 | 14010 | -0.0136 | 0.0313 | 0.66794 |
| MouseCellAtlas_all | Peripheral_Blood.Basophil_Prss34_high | 14459 | -0.0385 | 0.0885 | 0.66809 |
| MouseCellAtlas_Adult_all | Mammary_Gland.Secretory_alveoli_cell_Lyz2_high | 14459 | -0.0273 | 0.0626 | 0.6685 |
| DropViz_PC_level2 | Polydendrocyte_Tnr_Dlx1.10_2 | 14805 | -0.00163 | 0.00373 | 0.66858 |
| MouseCellAtlas_Stomach | Antral_mucous_cell_ | 14459 | -0.0277 | 0.0634 | 0.66867 |
| MouseCellAtlas_all | Fetal_Intestine.Epithelial_cell_Lgals2_high | 14459 | -0.039 | 0.0893 | 0.66869 |
| GSE99235_Mouse_Lung_Vascular | VSMC | 14559 | -0.00332 | 0.00759 | 0.66884 |
| Allen_Mouse_VISp_level2 | Igtp | 13868 | -0.00483 | 0.011 | 0.66917 |
| Linnarsson_GSE74672_Mouse_Hypothalamus_Neurons_level2 | GABA_2_Gucy1a3 | 14550 | -0.0205 | 0.0469 | 0.66919 |
| Linnarsson_GSE76381_Mouse_Midbrain | Rgl1 | 14550 | -0.0175 | 0.0399 | 0.66938 |
| TabulaMuris_FACS_all | Muscle.skeletal_muscle_satellite_cell | 13888 | -0.0037 | 0.00843 | 0.66941 |
| MouseCellAtlas_all | Bone_Marrow.Macrophage_S100a4_high | 14459 | -0.0277 | 0.0631 | 0.66946 |
| MouseCellAtlas_Neonatal_Heart | Neutrophil_Retnlg_high | 14459 | -0.032 | 0.0729 | 0.66954 |
| MouseCellAtlas_Neonatal_Muscle | Stromal_cell_Spp1_high | 14459 | -0.0222 | 0.0506 | 0.66954 |
| Linnarsson_MouseBrainAtlas_level6_rank4 | Scex | 15140 | -0.0253 | 0.0575 | 0.66965 |
| Linnarsson_GSE95315_Mouse_Dentate_gyrus | Mossy_Cyp26b | 11815 | -0.0106 | 0.024 | 0.6701 |
| DropViz_PC_level2 | Neuron_Slc17a7_Bcl6-Cbln1.3_1 | 14805 | -0.0042 | 0.00954 | 0.67017 |
| DropViz_all_level2 | PC.Neuron_Sc17a7_Tshz2-Smoc1.2_3 | 14805 | -0.00319 | 0.00724 | 0.67053 |
| DropViz_GP_level2 | Neuron_Gad1Gad2_Adora2a.3_3 | 14805 | -0.00318 | 0.00719 | 0.67055 |
| MouseCellAtlas_Embryo_all | Fetal_Brain.Macrophage | 14459 | -0.0313 | 0.071 | 0.67055 |
| Linnarsson_GSE74672_Mouse_Hypothalamus_Neurons_level2 | Avp_3_medium | 14550 | -0.0146 | 0.033 | 0.67057 |
| TabulaMuris_droplet_Trachea | neuroendocrine_cell | 13888 | -0.04 | 0.0901 | 0.67148 |
| TabulaMuris_FACS_all | Pancreas.pancreatic_A_cell | 13888 | -0.00292 | 0.00656 | 0.67207 |
| MouseCellAtlas_all | Mammary_Gland.NK_cell | 14459 | -0.038 | 0.0851 | 0.67222 |
| MouseCellAtlas_all | Mammary_Gland.Secretory_alveoli_cell_Taf1d_high | 14459 | -0.0261 | 0.0586 | 0.67236 |
| DropViz_SN_level2 | Neuron_Gad1Gad2_Gad2-Fos.3_8 | 14805 | -0.00218 | 0.00488 | 0.67244 |
| Linnarsson_GSE74672_Mouse_Hypothalamus_Neurons_level2 | Ghrh | 14550 | -0.0105 | 0.0234 | 0.67245 |
| MouseCellAtlas_all | Neonatal_Skin.Melanocyte | 14459 | -0.0281 | 0.0626 | 0.67302 |
| Allen_Mouse_VISp_level2 | Astro_Aqp4 | 13868 | -0.0033 | 0.00735 | 0.67304 |
| MouseCellAtlas_Neonatal_all | Neonatal_Muscle.Neutrophil_Elane_high | 14459 | -0.0261 | 0.0581 | 0.67343 |
| Linnarsson_MouseBrainAtlas_level5 | MEINH1 | 15140 | -0.0298 | 0.0663 | 0.67358 |
| MouseCellAtlas_all | Mammary_Gland.Secretory_alveoli_cell_Tubb5_high | 14459 | -0.0271 | 0.0602 | 0.67376 |
| Linnarsson_MouseBrainAtlas_level5 | SCINH10 | 15140 | -0.0251 | 0.0557 | 0.67385 |
| TabulaMuris_droplet_Bladder | bladder_cell | 13888 | -0.0163 | 0.0362 | 0.67395 |
| DropViz_all_level2 | FC.Neuron_Gad1Gad2_Cplx3-Reln.1_2 | 14805 | -0.00368 | 0.00815 | 0.67426 |
| Linnarsson_MouseBrainAtlas_level5 | HBINH5 | 15140 | -0.0145 | 0.0321 | 0.67427 |
| MouseCellAtlas_Mammary_Gland | Mammary_Gland_Lactation.Secretory_alveoli_cell_Igsf8_high | 14459 | -0.0405 | 0.0897 | 0.67428 |
| Linnarsson_GSE95752_Mouse_Dentate_gyrus | Mossy_Calb2 | 12517 | -0.0146 | 0.0322 | 0.67441 |
| MouseCellAtlas_Adult_all | Mammary_Gland.Secretory_alveoli_cell_Apod_high | 14459 | -0.029 | 0.064 | 0.6746 |
| DropViz_TH_level2 | Neuron_Slc17a6_Rora_Lrrtm2.2_9 | 14805 | -0.003 | 0.00663 | 0.67467 |
| MouseCellAtlas_Mammary_Gland | Mammary_Gland_Lactation.Muscle_cell | 14459 | -0.0432 | 0.0953 | 0.6748 |
| MouseCellAtlas_Bone_Marrow | Bone_Marrow.Pre.pro_B_cell | 14459 | -0.0348 | 0.0766 | 0.67494 |
| DropViz_all_level2 | FC.Polydendrocyte_Tnr_Dlx1.10_2 | 14805 | -0.00206 | 0.00455 | 0.67496 |
| Linnarsson_MouseBrainAtlas_level6_rank4 | Imgl | 15140 | -0.0178 | 0.0392 | 0.67532 |
| DropViz_all_level2 | CB.Astrocyte_Gja_Slc1a3.7_1 | 14805 | -0.00226 | 0.00495 | 0.67562 |
| Allen_Mouse_VISp_level2 | L5a_Hsd11b1 | 13868 | -0.00661 | 0.0145 | 0.67616 |
| DroNc_Human_Hippocampus | exPFC1 | 16914 | -0.0928 | 0.202 | 0.67687 |
| DropViz_all_level2 | STR.Mural_Rgs5Acta2_Cnn1.8_2 | 14805 | -0.00157 | 0.00341 | 0.67689 |
| DropViz_all_level2 | PC.Neuron_Slc17a7_Bcl6-Tshz2-Syt2.3_8 | 14805 | -0.0032 | 0.00696 | 0.677 |
| Linnarsson_GSE95315_Mouse_Dentate_gyrus | Cck_Tox | 11815 | -0.0277 | 0.06 | 0.6778 |
| DropViz_all_level2 | PC.Oligodendrocyte_Tfr_Sez6.9_5 | 14805 | -0.00252 | 0.00547 | 0.67781 |
| DropViz_all_level2 | HC.Astrocyte_Gja1_Nnat.7_1 | 14805 | -0.00271 | 0.00587 | 0.67783 |
| MouseCellAtlas_all | Mammary_Gland.Secretory_alveoli_cell_Kif5b_high | 14459 | -0.0302 | 0.0654 | 0.67799 |
| MouseCellAtlas_Bone_Marrow | Bone_Marrow_c_kit.Erythrocyte_progenitor_Car1_high | 14459 | -0.03 | 0.065 | 0.67805 |
| Linnarsson_MouseBrainAtlas_level5 | HBGLU6 | 15140 | -0.0119 | 0.0257 | 0.67829 |
| MouseCellAtlas_Adult_all | Mammary_Gland.T_cell_Pclaf_high | 14459 | -0.0375 | 0.0811 | 0.67829 |
| Linnarsson_GSE76381_Mouse_Midbrain | Mgl | 14550 | -0.0121 | 0.026 | 0.67867 |
| Linnarsson_MouseBrainAtlas_level5 | DEINH4 | 15140 | -0.0242 | 0.0521 | 0.67881 |
| MouseCellAtlas_Brain | Astrocyte_Pla2g7_high | 14459 | -0.0254 | 0.0546 | 0.67915 |
| MouseCellAtlas_Thymus | DPT_cell | 14459 | -0.0921 | 0.198 | 0.67919 |
| MouseCellAtlas_all | Mammary_Gland.Muscle_cell | 14459 | -0.0378 | 0.0812 | 0.67919 |
| MouseCellAtlas_Adult_all | Mammary_Gland.Macrophage_Plbd1_high | 14459 | -0.0338 | 0.0724 | 0.67947 |
| Linnarsson_MouseBrainAtlas_level5 | SYCHO1 | 15140 | -0.00799 | 0.0171 | 0.67986 |
| MouseCellAtlas_Adult_all | Thymus.Proliferating_thymocyte | 14459 | -0.0293 | 0.0626 | 0.67994 |
| MouseCellAtlas_Adult_all | Mammary_Gland.Secretory_alveoli_cell_Yif1b1_high | 14459 | -0.031 | 0.0663 | 0.68005 |
| MouseCellAtlas_all | Thymus.Proliferating_thymocyte | 14459 | -0.0299 | 0.0639 | 0.68013 |
| Allen_Mouse_ALM_level2 | L6b_ALM_Trh | 14744 | -0.00297 | 0.00634 | 0.68018 |
| Linnarsson_GSE76381_Mouse_Midbrain | NbML4 | 14550 | -0.0224 | 0.0478 | 0.68029 |
| Linnarsson_GSE103840_Mouse_Dorsal_horn | GABA_Calb2_Krt17 | 14550 | -0.0278 | 0.0594 | 0.68035 |
| TabulaMuris_FACS_all | Thymus.T_cell | 13888 | -0.00452 | 0.00963 | 0.6804 |
| Linnarsson_GSE74672_Mouse_Hypothalamus_Neurons_level2 | Vglut2_3_Crh_low | 14550 | -0.036 | 0.0768 | 0.68049 |
| Linnarsson_GSE95752_Mouse_Dentate_gyrus | Microglia | 12517 | -0.0206 | 0.0438 | 0.68056 |
| DropViz_all_level2 | HC.Neuron_Slc17a6_Htr1a.2_2 | 14805 | -0.00315 | 0.00672 | 0.68072 |
| DropViz_all_level2 | STR.Neurogenesis_Sox4_Tuba1c.2_3 | 14805 | -0.00198 | 0.00422 | 0.6808 |
| DropViz_HC_level2 | Neurogenesis_Sox4_Gabra5.13_5 | 14805 | -0.00282 | 0.00599 | 0.68093 |
| MouseCellAtlas_all | Fetal_Intestine.Macrophage_Pf4_high | 14459 | -0.0452 | 0.096 | 0.68104 |
| MouseCellAtlas_Adult_all | Mammary_Gland.Secretory_alveoli_cell_Malat1_high | 14459 | -0.0313 | 0.0665 | 0.68139 |
| MouseCellAtlas_Mammary_Gland | Mammary_Gland_Lactation.Secretory_alveoli_cell_Gpx3_high | 14459 | -0.0472 | 0.0998 | 0.68182 |
| GSE106678_Mouse_Cortex | Ex12 | 14573 | -0.059 | 0.125 | 0.68198 |
| TabulaMuris_droplet_Mammary | T_cell | 13888 | -0.0222 | 0.0468 | 0.68202 |
| TabulaMuris_FACS_Lung | Clara_cell | 13888 | -0.00397 | 0.00838 | 0.68204 |
| Linnarsson_GSE74672_Mouse_Hypothalamus_Neurons_level2 | Sst_1_low | 14550 | -0.023 | 0.0485 | 0.68215 |
| Linnarsson_GSE59739_Mouse_Dorsal_root_ganglion_level3 | Neurofilament_containing2 | 13848 | -0.00633 | 0.0134 | 0.68216 |
| MouseCellAtlas_Lung | Nuocyte | 14459 | -0.046 | 0.0972 | 0.68218 |
| MouseCellAtlas_Neonatal_Muscle | Mast_cell | 14459 | -0.0303 | 0.0637 | 0.68267 |
| TabulaMuris_droplet_Lung | mast_cell | 13888 | -0.0211 | 0.0444 | 0.68289 |
| TabulaMuris_droplet_all | Mammary.luminal_cell_of_lactiferous_duct | 13888 | -0.0142 | 0.0299 | 0.68291 |
| GSE98816_Mouse_Brain_Vascular | OL | 14130 | -0.00246 | 0.00516 | 0.68292 |
| MouseCellAtlas_all | Mammary_Gland.T_cell_Pclaf_high | 14459 | -0.0419 | 0.0878 | 0.68332 |
| DropViz_GP_level2 | Polydendrocyte_Tnr_Cyth3.4_7 | 14805 | -0.00123 | 0.00258 | 0.68336 |
| MouseCellAtlas_Neonatal_all | Neonatal_Calvaria.Muscle_cell_Myl9_high | 14459 | -0.0422 | 0.0885 | 0.68337 |
| Linnarsson_MouseBrainAtlas_level5 | CBNBL1 | 15140 | -0.0276 | 0.0577 | 0.68339 |
| DropViz_STR_level2 | Neuron_Gad1Gad2_Adora2a-Nefm.11_1 | 14805 | -0.00391 | 0.00819 | 0.68349 |
| MouseCellAtlas_all | Mammary_Gland.Secretory_alveoli_cell_AW112010_high | 14459 | -0.0305 | 0.0637 | 0.68413 |
| DropViz_FC_level2 | Neuron_Slc17a7_Syt6-Efna5.3_3 | 14805 | -0.00479 | 0.00999 | 0.68417 |
| DropViz_PC_level2 | Neuron_Sc17a7_Calb1-Ddit4l.2_1 | 14805 | -0.00529 | 0.011 | 0.6851 |
| TabulaMuris_FACS_all | Fat.B_cell | 13888 | -0.00468 | 0.00971 | 0.68519 |
| Allen_Mouse_VISp_level2 | L6a_Mgp | 13868 | -0.0066 | 0.0137 | 0.6853 |
| Linnarsson_GSE60361_Mouse_Cortex_Hippocampus_level2 | Oligo3 | 14010 | -0.00817 | 0.0169 | 0.6854 |
| DropViz_GP_level2 | Neuron_Gad1Gad2_Adora2a-Pde1c.3_6 | 14805 | -0.00342 | 0.00705 | 0.68596 |
| MouseCellAtlas_Uterus | B_cell | 14459 | -0.0232 | 0.0477 | 0.68634 |
| MouseCellAtlas_all | Mammary_Gland.Muscle__cell | 14459 | -0.0309 | 0.0637 | 0.68643 |
| MouseCellAtlas_Fetal_Stomache | Acinar_cell_Ctrb1_high | 14459 | -0.0551 | 0.113 | 0.68664 |
| MouseCellAtlas_Mammary_Gland | Mammary_Gland_Lactation.Secretory_alveoli_cell_Hspa1a_high | 14459 | -0.0477 | 0.098 | 0.68675 |
| MouseCellAtlas_all | Fetal_Lung.Neutrophil_Gm5483_high | 14459 | -0.0463 | 0.0949 | 0.68697 |
| MouseCellAtlas_Lung | Eosinophil_granulocyte | 14459 | -0.0339 | 0.0694 | 0.68731 |
| MouseCellAtlas_all | Mesenchymal_Stem_Cell_Cultured.Mesenchymal_stem_cell_Cxcl1_high | 14459 | -0.033 | 0.0676 | 0.68731 |
| DropViz_HC_level2 | Astrocyte_Gja1_Fabp7.7_3 | 14805 | -0.00244 | 0.00499 | 0.68747 |
| DropViz_FC_level2 | Neuron_Gad1Gad2-Slc17a8_Synpr-Sncg-Yjefn3.1_10 | 14805 | -0.00383 | 0.00783 | 0.6875 |
| MouseCellAtlas_all | Mammary_Gland.Macrophage_Plbd1_high | 14459 | -0.0356 | 0.0729 | 0.68757 |
| DropViz_all_level2 | STR.Polydendrocye_Tnr_Ctps.5_4 | 14805 | -0.00156 | 0.00317 | 0.68793 |
| DropViz_HC_level2 | Choroid_Plexus_Ttr.12_1 | 14805 | -0.00198 | 0.00404 | 0.68795 |
| GSE104276_Human_Prefrontal_cortex_per_ages | GW13_Microglia | 17199 | -0.0015 | 0.00305 | 0.68799 |
| Linnarsson_GSE74672_Mouse_Hypothalamus_Neurons_level2 | Hmit | 14550 | -0.0308 | 0.0627 | 0.68819 |
| MouseCellAtlas_Mammary_Gland | Mammary_Gland_Lactation.Secretory_alveoli_cell_Spp1_high | 14459 | -0.0523 | 0.106 | 0.6885 |
| TabulaMuris_FACS_all | Skin.stem_cell_of_epidermis | 13888 | -0.00273 | 0.00555 | 0.68864 |
| MouseCellAtlas_Adult_all | Brain.Myelinating_oligodendrocyte | 14459 | -0.0279 | 0.0564 | 0.68977 |
| DropViz_PC_level2 | Polydendrocyte_Tnr_Lims2-Tmsb4x.10_4 | 14805 | -0.00219 | 0.00441 | 0.69033 |
| DropViz_ENT_level2 | Neuron_Slc17a7_Zbtb20.4_1 | 14805 | -0.00174 | 0.0035 | 0.69049 |
| Linnarsson_GSE95315_Mouse_Dentate_gyrus | Microglia | 11815 | -0.0224 | 0.045 | 0.6905 |
| GSE93374_Mouse_Arc_ME_neurons | Sst_Nts | 13325 | -0.0749 | 0.151 | 0.69068 |
| MouseCellAtlas_all | Mammary_Gland.Secretory_alveoli_cell_Lyz2_high | 14459 | -0.0303 | 0.0606 | 0.69122 |
| MouseCellAtlas_Brain | Astrocyte_Atp1b2_high | 14459 | -0.0415 | 0.083 | 0.69134 |
| TabulaMuris_FACS_Pancreas | pancreatic_D_cell | 13888 | -0.00497 | 0.00992 | 0.69173 |
| DropViz_all_level2 | PC.Polydendrocyte_Tnr_Dlx1.10_2 | 14805 | -0.00193 | 0.00385 | 0.69179 |
| TabulaMuris_FACS_Thymus | T_cell | 13888 | -0.00911 | 0.0181 | 0.69228 |
| MouseCellAtlas_Adult_all | Muscle.Neutrophil_Camp_high | 14459 | -0.0257 | 0.0511 | 0.69263 |
| GSE93374_Mouse_Arc_ME_level2 | b1_tanycytes | 13325 | -0.063 | 0.125 | 0.69281 |
| DropViz_all_level2 | FC.Neuron_Slc17a7_Syt6-Efna5.3_3 | 14805 | -0.00404 | 0.00799 | 0.69322 |
| MouseCellAtlas_Bone_Marrow | Bone_Marrow.Neutrophil_Mmp8_high | 14459 | -0.0233 | 0.0462 | 0.69333 |
| DropViz_PC_level2 | Astrocyte_Gja1_Igfbp2.8_1 | 14805 | -0.00283 | 0.0056 | 0.69343 |
| TabulaMuris_droplet_all | Lung.mast_cell | 13888 | -0.0209 | 0.0412 | 0.69362 |
| MouseCellAtlas_Adult_all | Liver.B_cell_Jchain_high | 14459 | -0.0295 | 0.0582 | 0.69386 |
| MouseCellAtlas_all | Brain.Myelinating_oligodendrocyte | 14459 | -0.0286 | 0.0563 | 0.69393 |
| Linnarsson_GSE101601_Mouse_Somatosensory_cortex | Interneuron_kit | 14550 | -0.023 | 0.0454 | 0.69413 |
| MouseCellAtlas_Neonatal_all | Neonatal_Muscle.Brown_adipose_tissue | 14459 | -0.0197 | 0.0388 | 0.69413 |
| MouseCellAtlas_Adult_all | Prostate.Stromal_cell | 14459 | -0.0659 | 0.13 | 0.69424 |
| Linnarsson_GSE74672_Mouse_Hypothalamus_Neurons_level2 | Trh_1_low | 14550 | -0.0345 | 0.0678 | 0.69479 |
| MouseCellAtlas_Embryo_all | Fetal_Lung.Erythroblast_Hbb.bs_high | 14459 | -0.0671 | 0.132 | 0.69483 |
| MouseCellAtlas_Fetal_Intestine | Erythroblast_Hbb.bs_high | 14459 | -0.086 | 0.169 | 0.69495 |
| MouseCellAtlas_Neonatal_all | Neonatal_Heart.Macrophage | 14459 | -0.0385 | 0.0754 | 0.69508 |
| DropViz_FC_level2 | Astrocyte_Gja1.8_1 | 14805 | -0.00309 | 0.00603 | 0.69554 |
| MouseCellAtlas_Adult_all | Stomach.Epithelial_cell_Pla2g1b_high | 14459 | -0.0204 | 0.0398 | 0.69562 |
| DropViz_STR_level2 | Neuron_Gad1Gad2_Drd1-Tpbg.13_1 | 14805 | -0.00395 | 0.00771 | 0.69571 |
| DropViz_PC_level2 | Neuron_Sc17a7_Calb1-Tshz2.2_2 | 14805 | -0.0059 | 0.0115 | 0.69584 |
| MouseCellAtlas_all | Neonatal_Muscle.B_cell | 14459 | -0.0411 | 0.08 | 0.69635 |
| DroNc_Human_Hippocampus | exDG | 16914 | -0.0858 | 0.167 | 0.6968 |
| DropViz_STR_level2 | Neuron_Gad1Gad2_Drd1-Pde1a.10_3 | 14805 | -0.00422 | 0.00818 | 0.69686 |
| TabulaMuris_FACS_Lung | T_cell | 13888 | -0.00472 | 0.00915 | 0.69709 |
| MouseCellAtlas_Embryo_all | Fetal_Intestine.Epithelial_cell_Lgals2_high | 14459 | -0.0649 | 0.125 | 0.69751 |
| GSE106678_Mouse_Cortex | Ex9 | 14573 | -0.0505 | 0.0975 | 0.69756 |
| Linnarsson_GSE101601_Human_Temporal_cortex | GABA_1 | 17177 | -0.0642 | 0.124 | 0.69758 |
| MouseCellAtlas_all | Mammary_Gland.Secretory_alveoli_cell_Apod_high | 14459 | -0.032 | 0.0619 | 0.69769 |
| Allen_Mouse_VISp_level1 | Gluta_L6a | 13868 | -0.00803 | 0.0155 | 0.69774 |
| DropViz_GP_level2 | Neuron_Gad1Gad2_Six3-Pvalb.2_20 | 14805 | -0.00338 | 0.00653 | 0.69774 |
| DropViz_all_level2 | PC.Neuron_Slc17a7_Bcl6-Cbln1.3_1 | 14805 | -0.00386 | 0.00744 | 0.69798 |
| Linnarsson_GSE60361_Mouse_Cortex_Hippocampus_level1 | astrocytes_ependymal | 14010 | -0.0194 | 0.0374 | 0.69803 |
| MouseCellAtlas_Adult_all | Mammary_Gland.Secretory_alveoli_cell_Spp1_high | 14459 | -0.0367 | 0.0708 | 0.69809 |
| Linnarsson_GSE74672_Mouse_Hypothalamus_Neurons_level2 | Sst_3_medium | 14550 | -0.0186 | 0.0356 | 0.69877 |
| MouseCellAtlas_Embryo_all | Fetal_Stomache.Mast_cell | 14459 | -0.0725 | 0.139 | 0.69884 |
| MouseCellAtlas_Adult_all | Mammary_Gland.Secretory_alveoli_cell_Retnla_high | 14459 | -0.0345 | 0.0661 | 0.69933 |
| DropViz_PC_level1 | Oligodendrocyte | 14805 | -0.00356 | 0.00682 | 0.69947 |
| MouseCellAtlas_Mammary_Gland | Mammary_Gland_Lactation.Secretory_alveoli_cell_Klf2_high | 14459 | -0.0495 | 0.0945 | 0.69956 |
| DropViz_SN_level2 | Neuron_Slc17a6-Gad2-Th_Crhbp.3_7 | 14805 | -0.00346 | 0.00661 | 0.70005 |
| GSE93374_Mouse_Arc_ME_level2 | a1_tanycytes1 | 13325 | -0.0517 | 0.0986 | 0.70012 |
| MouseCellAtlas_all | Prostate.Stromal_cell | 14459 | -0.0681 | 0.13 | 0.70042 |
| MouseCellAtlas_all | Stomach.Epithelial_cell_Pla2g1b_high | 14459 | -0.0209 | 0.0397 | 0.70057 |
| Linnarsson_GSE76381_Human_Midbrain | Rgl1 | 14835 | -0.0289 | 0.0549 | 0.7006 |
| MouseCellAtlas_Adult_all | Mammary_Gland.Secretory_alveoli_cell_Igsf8_high | 14459 | -0.0335 | 0.0637 | 0.70061 |
| MouseCellAtlas_Bone_Marrow | Bone_Marrow_Mesenchyme.Erythroblast | 14459 | -0.0409 | 0.0778 | 0.70063 |
| MouseCellAtlas_Testis | Macrophage_Lyz2_high | 14459 | -0.0517 | 0.0981 | 0.70071 |
| MouseCellAtlas_Neonatal_Rib | Neutrophil | 14459 | -0.0326 | 0.0618 | 0.70093 |
| MouseCellAtlas_all | Muscle.Neutrophil_Camp_high | 14459 | -0.0267 | 0.0506 | 0.70141 |
| MouseCellAtlas_Pancreas | Erythroblast_Hbb.bt_high | 14459 | -0.034 | 0.0643 | 0.70159 |
| MouseCellAtlas_all | Neonatal_Rib.Granulocyte | 14459 | -0.0473 | 0.0895 | 0.70165 |
| GSE106678_Mouse_Cortex | Inh1 | 14573 | -0.0388 | 0.0733 | 0.70174 |
| Linnarsson_GSE78845_Mouse_Ganglia | Noradregergic_neurons_3 | 12817 | -0.0257 | 0.0485 | 0.70176 |
| Linnarsson_MouseBrainAtlas_level6_rank4 | Chmn | 15140 | -0.0145 | 0.0273 | 0.70198 |
| Linnarsson_MouseBrainAtlas_level5 | SYNOR2 | 15140 | -0.00976 | 0.0184 | 0.70232 |
| Linnarsson_GSE76381_Human_Midbrain | Peric | 14835 | -0.0184 | 0.0346 | 0.70249 |
| MouseCellAtlas_Placenta | Spongiotrophoblast_Phlda2_high | 14459 | -0.0387 | 0.0727 | 0.7027 |
| DropViz_GP_level2 | Neuron_Slc17a6_Rspo3.2_9 | 14805 | -0.00394 | 0.00739 | 0.70305 |
| DropViz_all_level2 | PC.Neuron_Sc17a7_Calb1-Ddit4l.2_1 | 14805 | -0.00417 | 0.0078 | 0.70363 |
| MouseCellAtlas_all | Mammary_Gland.Secretory_alveoli_cell_Yif1b1_high | 14459 | -0.0343 | 0.064 | 0.70398 |
| MouseCellAtlas_all | Fetal_Stomache.Acinar_cell_Ctrb1_high | 14459 | -0.0416 | 0.0775 | 0.70403 |
| Linnarsson_MouseBrainAtlas_level6_rank4 | Ipvm | 15140 | -0.0219 | 0.0408 | 0.70419 |
| MouseCellAtlas_all | Mammary_Gland.Secretory_alveoli_cell_Malat1_high | 14459 | -0.0346 | 0.0645 | 0.70434 |
| MouseCellAtlas_all | Liver.B_cell_Jchain_high | 14459 | -0.0306 | 0.0571 | 0.70435 |
| Linnarsson_MouseBrainAtlas_level6_rank4 | Syno | 15140 | -0.00996 | 0.0186 | 0.70437 |
| Linnarsson_GSE76381_Mouse_Midbrain | Sert | 14550 | -0.025 | 0.0466 | 0.70441 |
| MouseCellAtlas_Adult_all | Kidney.Distal_collecting_duct_principal_cell_Hsd11b2_high | 14459 | -0.0365 | 0.0679 | 0.70451 |
| TabulaMuris_droplet_all | Muscle.B_cell | 13888 | -0.0269 | 0.0501 | 0.70453 |
| MouseCellAtlas_Adult_all | Uterus.Monocyte | 14459 | -0.0381 | 0.0708 | 0.7049 |
| DropViz_all_level2 | HC.Neuron_Gad1Gad2_Sst_Spon1.1_5 | 14805 | -0.00355 | 0.00659 | 0.70501 |
| MouseCellAtlas_Adult_all | Mammary_Gland.Secretory_alveoli_cell_Cide.Wfdc3.Wap_high | 14459 | -0.0331 | 0.0612 | 0.70545 |
| GSE87544_Mouse_Hypothalamus | Glu7 | 13878 | -0.0393 | 0.0728 | 0.70546 |
| MouseCellAtlas_Mammary_Gland | Mammary_Gland_Lactation.Secretory_alveoli_cell_Tyrobp_high | 14459 | -0.0511 | 0.0943 | 0.70597 |
| Allen_Mouse_ALM_level2 | L5_ALM_Aldh3b1_2 | 14744 | -0.00573 | 0.0106 | 0.7061 |
| Allen_Mouse_ALM_level2 | Sst_Th | 14744 | -0.00482 | 0.00889 | 0.70616 |
| Linnarsson_MouseBrainAtlas_level6_rank4 | Hind | 15140 | -0.0143 | 0.0264 | 0.70629 |
| Allen_Mouse_VISp_level1 | GABA_Sncg | 13868 | -0.00574 | 0.0106 | 0.70634 |
| DropViz_all_level2 | HC.Neuron_Gad1Gad2_Id2_Sfrp2.1_2 | 14805 | -0.00296 | 0.00545 | 0.70635 |
| MouseCellAtlas_Fetal_Lung | Macrophage_Pf4high | 14459 | -0.0506 | 0.0933 | 0.70639 |
| MouseCellAtlas_Mammary_Gland | Mammary_Gland_Lactation.Dendritic_cell | 14459 | -0.0528 | 0.0972 | 0.70649 |
| Linnarsson_GSE78845_Mouse_Ganglia | Noradregergic_neurons_5 | 12817 | -0.0235 | 0.0432 | 0.70663 |
| MouseCellAtlas_Peripheral_Blood | Monocyte_F13a1_high | 14459 | -0.0521 | 0.0958 | 0.70666 |
| MouseCellAtlas_Muscle | Neutrophil_Prg2_high | 14459 | -0.0191 | 0.0351 | 0.7067 |
| Allen_Mouse_ALM_level2 | Sst_Mrap2_2 | 14744 | -0.00795 | 0.0146 | 0.70671 |
| DropViz_all_level2 | CB.Neuron_Gad1Gad2_Pvalb-Nefh.3_1 | 14805 | -0.00319 | 0.00586 | 0.70695 |
| MouseCellAtlas_Adult_all | Mammary_Gland.Secretory_alveoli_cell_Gpx3_high | 14459 | -0.0375 | 0.0687 | 0.70721 |
| MouseCellAtlas_Adult_all | Small_Intestine.B_cell_Jchain_high | 14459 | -0.0334 | 0.0612 | 0.70733 |
| MouseCellAtlas_Neonatal_all | Neonatal_Heart.Neutrophil_Ngp_high | 14459 | -0.0331 | 0.0605 | 0.70755 |
| MouseCellAtlas_all | Embryonic_Stem_Cell.ES_Nedd4_high | 14459 | -0.0335 | 0.0612 | 0.70774 |
| MouseCellAtlas_Mammary_Gland | Mammary_Gland_Lactation.B_cell_Igkv6.15_high | 14459 | -0.051 | 0.0931 | 0.70801 |
| DropViz_all_level2 | FC.Neuron_Gad1Gad2-Slc17a8_Synpr-Sncg-Yjefn3.1_10 | 14805 | -0.00403 | 0.00736 | 0.7081 |
| MouseCellAtlas_Adult_all | Testis.Macrophage_Lyz2_high | 14459 | -0.0318 | 0.0581 | 0.70814 |
| TabulaMuris_FACS_Skin | epidermal_cell | 13888 | -0.0109 | 0.0199 | 0.70838 |
| DropViz_STR_level1 | Endothelial_tip | 14805 | -0.003 | 0.00546 | 0.70844 |
| GSE104276_Human_Prefrontal_cortex_per_ages | GW19_GABAergic_neurons | 17199 | -0.00604 | 0.011 | 0.70851 |
| MouseCellAtlas_Kidney | Stromal_cell_Cxcl10_high | 14459 | -0.0383 | 0.0698 | 0.70851 |
| Linnarsson_GSE103840_Mouse_Dorsal_horn | Glut_Reln_Npff | 14550 | -0.0372 | 0.0676 | 0.70873 |
| MouseCellAtlas_Embryo_all | Fetal_Intestine.Macrophage_Pf4_high | 14459 | -0.054 | 0.0981 | 0.70879 |
| MouseCellAtlas_Adult_all | Mammary_Gland.Secretory_alveoli_cell_Hspa1a_high | 14459 | -0.0369 | 0.067 | 0.7089 |
| Allen_Mouse_VISp_level2 | Vip_Mybpc1 | 13868 | -0.0067 | 0.0122 | 0.709 |
| Allen_Mouse_VISp_level1 | Gluta_L4 | 13868 | -0.00786 | 0.0143 | 0.70902 |
| DropViz_all_level2 | TH.Neuron_Slc17a6_Nrgn.3_9 | 14805 | -0.00386 | 0.00701 | 0.7091 |
| MouseCellAtlas_all | Placenta.Granulocyte_monocyte_progenitors | 14459 | -0.0411 | 0.0747 | 0.70914 |
| Linnarsson_MouseBrainAtlas_level5 | SCGLU10 | 15140 | -0.0307 | 0.0557 | 0.70926 |
| DropViz_ENT_level2 | Neuron_Gad1Gad2-Slc17a6_Cbln1.4_3 | 14805 | -0.00266 | 0.00481 | 0.70947 |
| MouseCellAtlas_all | Neonatal_Skin.Brown_adipose_tissue_Cidea_high | 14459 | -0.0211 | 0.0383 | 0.70947 |
| DropViz_all_level2 | ENT.Mural_Rgs5Acta2_Cnn1.3_5 | 14805 | -0.00192 | 0.00347 | 0.70979 |
| DropViz_all_level2 | STR.Neuron_Slc17a6_Oprk1.13_6 | 14805 | -0.00175 | 0.00317 | 0.70998 |
| DropViz_STR_level2 | Polydendrocye_Tnr_Bmp4.5_2 | 14805 | -0.00269 | 0.00485 | 0.71038 |
| Linnarsson_MouseBrainAtlas_level5 | RGDG | 15140 | -0.029 | 0.0523 | 0.71041 |
| Linnarsson_GSE60361_Mouse_Cortex_Hippocampus_level2 | Int9 | 14010 | -0.0159 | 0.0286 | 0.71045 |
| DropViz_HC_level1 | Neurogenesis | 14805 | -0.00349 | 0.00629 | 0.71059 |
| Allen_Mouse_ALM_level2 | Lamp5_Rgs12_3 | 14744 | -0.00395 | 0.00711 | 0.71074 |
| DropViz_all_level1 | CB.Oligodendrocyte | 14805 | -0.00293 | 0.00527 | 0.71091 |
| Linnarsson_MouseBrainAtlas_level5 | HYPEP5 | 15140 | -0.024 | 0.043 | 0.71108 |
| MouseCellAtlas_Mammary_Gland | Mammary_Gland_Lactation.Secretory_alveoli_cell_Hes1_high | 14459 | -0.056 | 0.101 | 0.71112 |
| DropViz_all_level2 | STR.Neuron_Slc17a7_Cplx3.13_7 | 14805 | -0.00269 | 0.00483 | 0.71128 |
| MouseCellAtlas_all | Kidney.Distal_collecting_duct_principal_cell_Hsd11b2_high | 14459 | -0.0376 | 0.0674 | 0.71158 |
| DropViz_all_level2 | SN.Polydendrocyte_Tnr_Cspg5-Dad1.6_2 | 14805 | -0.00269 | 0.00481 | 0.71188 |
| MouseCellAtlas_Embryonic_Mesenchyme | Progenitor_cell_Ptprcap_high | 14459 | -0.0426 | 0.0761 | 0.71199 |
| DropViz_HC_level2 | Gad1Gad2_Htr3a_Phlda1.1_18 | 14805 | -0.00402 | 0.00717 | 0.71218 |
| MouseCellAtlas_Embryo_all | Fetal_Lung.Neutrophil_Gm5483_high | 14459 | -0.069 | 0.123 | 0.7122 |
| DropViz_all_level2 | PC.Neuron_Sc17a7_Calb1-Tshz2.2_2 | 14805 | -0.00454 | 0.00809 | 0.71255 |
| DropViz_FC_level1 | Polydendrocyte | 14805 | -0.00464 | 0.00827 | 0.71275 |
| MouseCellAtlas_all | Neonatal_Calvaria.Endothelial_cell | 14459 | -0.0406 | 0.0723 | 0.7128 |
| DropViz_TH_level2 | Oligodendrocyte_Trf_Klk6.8_1 | 14805 | -0.00314 | 0.00558 | 0.71295 |
| Allen_Mouse_VISp_level1 | NonNeu_Astro | 13868 | -0.00434 | 0.00773 | 0.71296 |
| TabulaMuris_droplet_Lung | T_cell | 13888 | -0.0252 | 0.0448 | 0.71325 |
| MouseCellAtlas_all | Fetal_Liver.Stem_and_progenitor_cell | 14459 | -0.0426 | 0.0757 | 0.7133 |
| DropViz_GP_level2 | Mural_Rgs5Acta2_Acta2.8_1 | 14805 | -0.00262 | 0.00465 | 0.71372 |
| DropViz_TH_level2 | Neuron_Slc17a6_Rora_Atp2b4.2_1 | 14805 | -0.00449 | 0.00795 | 0.71377 |
| GSE87544_Mouse_Hypothalamus | Glu14 | 13878 | -0.0304 | 0.0538 | 0.71398 |
| TabulaMuris_FACS_all | Bladder.bladder_cell | 13888 | -0.00325 | 0.00575 | 0.71427 |
| DropViz_HC_level2 | Gad1Gad2Chat_Htr3a_Chat.1_17 | 14805 | -0.00478 | 0.00843 | 0.71479 |
| DropViz_STR_level2 | Oligodendrocyte_Trf_Klk6.3_1 | 14805 | -0.00311 | 0.00547 | 0.71517 |
| DropViz_TH_level2 | Polydendrocyte_Tnr_Bmp4.12_3 | 14805 | -0.00314 | 0.00552 | 0.71521 |
| DropViz_GP_level2 | Neuron_Gad1Gad2_Cadm2.3_4 | 14805 | -0.00342 | 0.00601 | 0.71535 |
| MouseCellAtlas_all | Testis.Macrophage_Lyz2_high | 14459 | -0.0328 | 0.0576 | 0.7157 |
| Linnarsson_GSE101601_Mouse_Somatosensory_cortex | Interneuron_vip2 | 14550 | -0.0385 | 0.0674 | 0.71629 |
| DropViz_PC_level2 | Neuron_Slc17a7_Syt6-Tox.1_7 | 14805 | -0.00608 | 0.0106 | 0.71639 |
| MouseCellAtlas_Fetal_Stomache | Hepatocyte.Like_Cell_ | 14459 | -0.0264 | 0.0462 | 0.7166 |
| MouseCellAtlas_all | Mesenchymal_Stem_Cell_Cultured.Mesenchymal_stem_cell_Tmsb10_high | 14459 | -0.0397 | 0.0692 | 0.71663 |
| DropViz_CB_level2 | Astrocyte_Gja_Slc1a3.7_1 | 14805 | -0.00329 | 0.00573 | 0.7168 |
| TabulaMuris_droplet_all | Trachea.neuroendocrine_cell | 13888 | -0.0488 | 0.0851 | 0.7168 |
| DropViz_all_level2 | HC.Oligodendrocyte_Trf_Ndrg2.8_2 | 14805 | -0.00323 | 0.00562 | 0.71696 |
| Linnarsson_MouseBrainAtlas_level6_rank4 | Gdgrg | 15140 | -0.0342 | 0.0595 | 0.71716 |
| MouseCellAtlas_Muscle | Neutrophil_Retnlg_high | 14459 | -0.0267 | 0.0463 | 0.71796 |
| MouseCellAtlas_all | Neonatal_Calvaria.Neuron | 14459 | -0.066 | 0.114 | 0.71804 |
| Linnarsson_MouseBrainAtlas_level6_rank4 | Cbnn | 15140 | -0.0562 | 0.0972 | 0.71828 |
| GSE104276_Human_Prefrontal_cortex_per_ages | GW09_Stem_cells | 17199 | -0.00569 | 0.00984 | 0.71841 |
| MouseCellAtlas_Adult_all | Bone_Marrow.Neutrophil_Fcnb_high | 14459 | -0.0315 | 0.0543 | 0.71867 |
| MouseCellAtlas_all | Uterus.Monocyte | 14459 | -0.0395 | 0.0682 | 0.71874 |
| MouseCellAtlas_all | Small_Intestine.B_cell_Jchain_high | 14459 | -0.0346 | 0.0598 | 0.71888 |
| DropViz_TH_level1 | Macrophage | 14805 | -0.00252 | 0.00435 | 0.71931 |
| GSE106678_Mouse_Cortex | Ex15 | 14573 | -0.0665 | 0.114 | 0.71948 |
| MouseCellAtlas_Adult_all | Mammary_Gland.Secretory_alveoli_cell_Trf_high | 14459 | -0.0434 | 0.0747 | 0.71951 |
| DropViz_all_level1 | TH.Oligodendrocyte | 14805 | -0.00435 | 0.00747 | 0.71968 |
| Allen_Mouse_ALM_level2 | Sst_Myh8_5 | 14744 | -0.00524 | 0.009 | 0.71974 |
| Linnarsson_MouseBrainAtlas_level6_rank3 | Hindbrain_neurons | 15140 | -0.0167 | 0.0288 | 0.71974 |
| MouseCellAtlas_all | Mammary_Gland.Secretory_alveoli_cell_Spp1_high | 14459 | -0.0398 | 0.0684 | 0.71982 |
| Linnarsson_GSE74672_Mouse_Hypothalamus_Neurons_level2 | GABA_7_Pomc | 14550 | -0.039 | 0.067 | 0.71989 |
| DropViz_all_level1 | PC.Oligodendrocyte | 14805 | -0.0042 | 0.00721 | 0.72003 |
| DropViz_STR_level2 | Neuron_Gad1Gad2_Drd1-Nefm.10_4 | 14805 | -0.00474 | 0.00812 | 0.72042 |
| DropViz_all_level2 | FC.Astrocyte_Gja1.8_1 | 14805 | -0.00373 | 0.00639 | 0.72047 |
| MouseCellAtlas_Uterus | Smooth_muscle_cell_Rgs5_high | 14459 | -0.0474 | 0.0811 | 0.72066 |
| MouseCellAtlas_Adult_all | Mammary_Gland.Secretory_alveoli_cell_Hes1_high | 14459 | -0.0398 | 0.068 | 0.72073 |
| DropViz_all_level1 | FC.Mural | 14805 | -0.00401 | 0.00686 | 0.72084 |
| MouseCellAtlas_Mammary_Gland | Mammary_Gland_Lactation.Secretory_alveoli_cell_Snord118_high | 14459 | -0.0563 | 0.0961 | 0.72122 |
| TabulaMuris_FACS_all | Colon.enteroendocrine_cell | 13888 | -0.00382 | 0.00651 | 0.72127 |
| MouseCellAtlas_Embryo_all | Fetal_Stomache.Acinar_cell_Ctrb1_high | 14459 | -0.0557 | 0.0949 | 0.7214 |
| TabulaMuris_FACS_all | Liver.natural_killer_cell | 13888 | -0.00462 | 0.00788 | 0.7214 |
| MouseCellAtlas_Adult_all | Bone_Marrow.Basophils | 14459 | -0.0507 | 0.0862 | 0.72154 |
| MouseCellAtlas_Neonatal_Heart | Cardiac_muscle_cell | 14459 | -0.0426 | 0.0725 | 0.72156 |
| TabulaMuris_FACS_all | Liver.Kupffer_cell | 13888 | -0.00489 | 0.00832 | 0.72161 |
| MouseCellAtlas_all | Mammary_Gland.Secretory_alveoli_cell_Retnla_high | 14459 | -0.0378 | 0.0642 | 0.72165 |
| TabulaMuris_FACS_all | Lung.natural_killer_cell | 13888 | -0.00517 | 0.00879 | 0.72177 |
| GSE98816_Mouse_Brain_Vascular | EC1 | 14130 | -0.00719 | 0.0122 | 0.72191 |
| MouseCellAtlas_all | Mammary_Gland.Secretory_alveoli_cell_Igsf8_high | 14459 | -0.0364 | 0.0618 | 0.72207 |
| MouseCellAtlas_Adult_all | Mammary_Gland.Secretory_alveoli_cell_Klf2_high | 14459 | -0.0395 | 0.0671 | 0.72212 |
| DropViz_all_level2 | PC.Polydendrocyte_Tnr_Lims2-Tmsb4x.10_4 | 14805 | -0.00277 | 0.00469 | 0.72272 |
| DropViz_STR_level2 | Astrocyte_Gja1_Sparc-Car2.4_2 | 14805 | -0.00345 | 0.00585 | 0.72274 |
| MouseCellAtlas_Adult_all | Mammary_Gland.Secretory_alveoli_cell_Snord118_high | 14459 | -0.0386 | 0.0653 | 0.72276 |
| MouseCellAtlas_all | Neonatal_Heart.Left_ventricle_cardiomyocyte_Myl2_high | 14459 | -0.0332 | 0.0558 | 0.72402 |
| MouseCellAtlas_all | Mammary_Gland.Secretory_alveoli_cell_Cide.Wfdc3.Wap_high | 14459 | -0.0356 | 0.0599 | 0.72411 |
| MouseCellAtlas_Stomach | Macrophage | 14459 | -0.0316 | 0.0531 | 0.72421 |
| MouseCellAtlas_Fetal_Brain | Microglia | 14459 | -0.0474 | 0.0795 | 0.72441 |
| MouseCellAtlas_Mammary_Gland | Mammary_Gland_Lactation.Secretory_alveoli_cell_Cnp_high | 14459 | -0.0529 | 0.0885 | 0.72502 |
| Linnarsson_MouseBrainAtlas_level5 | SYCHO2 | 15140 | -0.0105 | 0.0175 | 0.72525 |
| DropViz_HC_level2 | Gad1Gad2_Cplx3_Rxfp1.1_11 | 14805 | -0.00485 | 0.0081 | 0.72547 |
| MouseCellAtlas_Pancreas | Smooth_muscle_cell_Acta2_high | 14459 | -0.0465 | 0.0775 | 0.72571 |
| TabulaMuris_droplet_all | Marrow.T_cell | 13888 | -0.025 | 0.0416 | 0.72598 |
| DropViz_SN_level2 | Oligodendrocyte_Tfr_Plin3.10_2 | 14805 | -0.00344 | 0.00572 | 0.72599 |
| Linnarsson_GSE60361_Mouse_Cortex_Hippocampus_level2 | Astro2 | 14010 | -0.0169 | 0.0281 | 0.72613 |
| MouseCellAtlas_Adult_all | Mammary_Gland.Secretory_alveoli_cell_Tyrobp_high | 14459 | -0.0403 | 0.067 | 0.72618 |
| MouseCellAtlas_Adult_all | Uterus.Glandular_epithelium_Ltf_high | 14459 | -0.0459 | 0.0763 | 0.72631 |
[truncated: 145,192 more chars]
